# A decline in molluscan carbonate production driven by the loss of vegetated habitats encoded in the Holocene sedimentary record of the Gulf of Trieste

**DOI:** 10.1111/sed.12516

**Published:** 2018-08-25

**Authors:** Adam Tomašových, Ivo Gallmetzer, Alexandra Haselmair, Darrell S. Kaufman, Borut Mavrič, Martin Zuschin

**Affiliations:** ^1^ Earth Science Institute Slovak Academy of Sciences Dúbravska cesta 9 Bratislava 84005 Slovakia; ^2^ Department of Palaeontology University of Vienna Althanstrasse 14 Vienna 1090 Austria; ^3^ School of Earth Sciences & Environmental Sustainability Northern Arizona University Campus Box 4099 Flagstaff AZ 86011 USA; ^4^ Marine Biology Station National Institute of Biology Fornače 41 Piran SI‐6330 Slovenia

**Keywords:** Bioturbation, carbonate production, mollusca, northern Adriatic Sea, taphonomy, time averaging

## Abstract

Carbonate sediments in non‐vegetated habitats on the north‐east Adriatic shelf are dominated by shells of molluscs. However, the rate of carbonate molluscan production prior to the 20th century eutrophication and overfishing on this and other shelves remains unknown because: (i) monitoring of ecosystems prior to the 20th century was scarce; and (ii) ecosystem history inferred from cores is masked by condensation and mixing. Here, based on geochronological dating of four bivalve species, carbonate production during the Holocene is assessed in the Gulf of Trieste, where algal and seagrass habitats underwent a major decline during the 20th century. Assemblages of sand‐dwelling *Gouldia minima* and opportunistic *Corbula gibba* are time‐averaged to >1000 years and *Corbula gibba* shells are older by >2000 years than shells of co‐occurring *Gouldia minima*. This age difference is driven by temporally disjunct production of two species coupled with decimetre‐scale mixing. Stratigraphic unmixing shows that *Corbula gibba* declined in abundance during the highstand phase and increased again during the 20th century. In contrast, one of the major contributors to carbonate sands – *Gouldia minima* – increased in abundance during the highstand phase, but declined to almost zero abundance over the past two centuries. *Gouldia minima* and herbivorous gastropods associated with macroalgae or seagrasses are abundant in the top‐core increments but are rarely alive. Although *Gouldia minima* is not limited to vegetated habitats, it is abundant in such habitats elsewhere in the Mediterranean Sea. This live–dead mismatch reflects the difference between highstand baseline communities (with soft‐bottom vegetated zones and hard‐bottom *Arca* beds) and present‐day oligophotic communities with organic‐loving species. Therefore, the decline in light penetration and the loss of vegetated habitats with high molluscan production traces back to the 19th century. More than 50% of the shells on the sea floor in the Gulf of Trieste reflect inactive production that was sourced by heterozoan carbonate factory in algal or seagrass habitats.

## Introduction

Depositional models of temperate carbonate ramps with heterozoan skeletal assemblages in semi‐enclosed water bodies are primarily based on environments in the Mediterranean Sea (Betzler *et al*., 1997, 2011; Pomar *et al*., 2004; Wilson & Vecsei, 2005; Tropeano & Spalluto, 2006). In the northern Adriatic Sea, these carbonate‐rich deposits formed by mollusc‐dominated sands are presently located in zones of limited light penetration; they occur mainly in the eastern part of the northern Adriatic Sea at locations far from river‐borne sediment input (in contrast to the north‐west Adriatic Sea with high accumulation of siliciclastic muds) and belong to the so‐called coastal detritic or muddy detritic communities without macroalgae or seagrasses (Péres, 1967; Lipej *et al*., 2016). However, benthic habitats in the north‐east Adriatic Sea, especially off Istria and in the Gulf of Trieste, were negatively affected by eutrophication, pollution, trawling, mucilages, seasonal hypoxia and overfishing of top consumers in the late 20th century (Coll *et al*., 2009; Lotze *et al*., 2011; Giani *et al*., 2012; Kowalewski *et al*., 2015; Mautner *et al*., 2018). Local extinctions and onshore retreats of vegetation in the northern Adriatic Sea were documented since the 1960s, including the loss of *Posidonia* seagrass (Simonetti, 1966; Ghirardelli *et al*., 1973; Zavodnik, 1977; Orel *et al*., 1982; Zavodnik & Jaklin, 1990; Caressa *et al*., 1995) and macroalgae (Vukovic, 1982; Munda, 1993). These changes parallel anthropogenic disturbances that affected marine ecosystems in the 20th century all over the planet and led to benthic mass mortalities (Boesch & Rabalais, 1991), extinction of ecosystem engineers (Zu Ermgassen *et al*., 2012) and trophic downgrading (Estes *et al*., 2011).

In light of the shifting baselines and given that the monitoring of benthic ecosystems in the northern Adriatic Sea and in other shelf regions was rare prior to the 20th century (Lotze *et al*., 2006), rates and modes of net carbonate production measured during the 20th or 21st century might not be representative of natural highstand conditions. Carbonate‐rich sediments presently located in non‐vegetated habitats with high turbidity and pollution can either reflect ghosts of well‐lit vegetated habitats in the past and/or transport from shallower, infralittoral zones (James *et al*., 2009). In the first case, they can mark a temporal loss in molluscan production due to changes in water quality and light penetration. Major losses in the extent of oyster reefs and scallop grounds, and reductions in the diversity of molluscan communities over the past decades and centuries were documented worldwide in marine siliciclastic environments (Edgar & Samson, 2004; Poirier *et al*., 2010; Wilberg *et al*., 2011; Thurstan *et al*., 2013; Alleway & Connell, 2015; Martinelli *et al*., 2017; Rick *et al*., 2017). However, changes in carbonate production before and after major anthropogenic impacts in temperate carbonate environments remain less explored than in the tropics (Perry *et al*., 2008; Cramer *et al*., 2015; Albano *et al*., 2016; Smith *et al*., 2016; Gilad *et al*., 2018). Ecological and palaeoecological analyses performed in the northern Adriatic Sea detected that the composition of benthic communities inhabiting siliciclastic muds changed towards the dominance of opportunistic species during the 20th century (Crema *et al*., 1991; Barmawidjaja *et al*., 1995; Gallmetzer *et al*., 2017; Tomašových *et al*., 2018). It remains unclear whether communities inhabiting mixed or carbonate sediments were affected by similar dynamics and whether these shifts were associated with reduced carbonate production, for example, owing to high mortality of large bivalves (Hrs‐Brenko, 1980; Peharda *et al*., 2006; Mautner *et al*., 2018), loss of macroalgal and seagrass habitats (Falace *et al*., 2010; Orlando‐Bonaca & Mavrič, 2014; Iveša *et al*., 2016) and/or reduced carbonate preservation owing to eutrophication‐driven degradation of carbonate shells (Hallock, 1988; Lescinsky *et al*., 2002). The evidence of past carbonate production can be detected in palimpsest deposits on the basis of shell staining and/or large mismatch between species habitat affinities and present‐day habitat conditions(Corselli *et al*., 1994; Toscano & Sorgente, 2002; Brandano & Civitelli, 2007; Rivers *et al*., 2007). However, such criteria are insufficient when a significant decline in production occurred over the past decades or few centuries.

Skeletal assemblages in sediment cores provide a long‐term perspective on ecosystem changes (Kidwell & Tomašových, 2013); they allow distinction between short‐term (seasonal or inter‐annual) and reversible changes in community composition on one hand and major collapses that lead to irreversible changes or hysteresis on the other hand (Hsieh *et al*., 2005; Perry & Smithers, 2011). However, this approach also has drawbacks because low sedimentation rates and bioturbational mixing typically generate time‐averaged assemblages (Anderson *et al*., 1998; Scarponi & Kowalewski, 2007; Tomašových & Kidwell, 2010; Scarponi *et al*., 2013) that can be inert to the most recent changes in ecosystem composition (Kidwell, 2007, 2008). This inertia effect can be especially pronounced in carbonate environments with inherently low sedimentation rates and deep bioturbation (Kosnik *et al*., 2007, 2013; Bentley & Nittrouer, 2012). In this study, the effect of low sedimentation rate and bioturbational mixing on the temporal resolution of death assemblages is considered through dating a large number of shells (Tomašových & Kidwell, 2017; Tomašových *et al*., 2017) and by using living assemblages as indicators of the most recent conditions unaffected by mixing (Kidwell, 2013; Tomašových *et al*., 2016). With these two approaches, changes in molluscan carbonate production over the Holocene in the north‐east Adriatic Sea (Gulf of Trieste) and the effects of anthropogenic impacts on molluscan production can be reconstructed.

Age unmixing is based on down‐core changes in frequency distribution of post‐mortem ages of the infaunal bivalves *Gouldia minima* and *Corbula gibba* (*Varicorbula gibba* according to Anderson & Roopnarine, 2003) in 140 to 150 cm long cores collected at two sites (Piran 1 and Piran 2) in the southern Gulf of Trieste. The composition of living assemblages is assessed using samples from 14 stations collected at similar water depths. Mautner *et al*. (2018) analyzed temporal changes in the composition of assemblages on the basis of relative abundances of molluscs and described geochemical attributes of a sediment core at Piran 2, using amino acid data calibrated by ^14^C of two bivalve species (*Corbula gibba* and *Gouldia minima*). Mautner *et al*. (2018) showed that shell beds formed by epifaunal bivalves (*Arca noae*) were eradicated by overfishing in the 20th century. This study first evaluates the effects of sedimentation and bioturbational mixing on down‐core distribution of molluscan assemblages at both sites and, second, assesses temporal changes in carbonate production on the basis of age unmixing of *C. gibba* and *G. minima*, and on the basis of comparison of time‐averaged core assemblages with non‐averaged living assemblages. The current study then expands the analyses to the second site (Piran 1), including new abundance data and new amino acid data of *G. minima*. Further, it assesses down‐core changes in ^210^Pb and constrains the age of the major shell bed by new ^14^C data of shell‐bed forming species (*Arca* and *Ostrea*) at both sites. The age model used in Mautner *et al*. (2018) is revised based on new *Arca* and *Ostrea* radiocarbon ages.

## Setting

**Figure 1:**
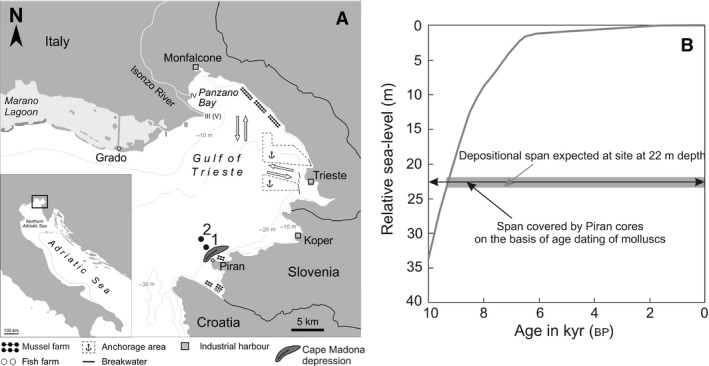
(A) Location of the two stations off Piran in the southern Gulf of Trieste (black points). The dark grey structure south of station 1 corresponds to the Cape Madona depression. (B) Temporal changes in relative sea‐level (m) in the Gulf of Trieste over the past 10 000 years (Antonioli *et al*., 2007). The maximum age of shells recovered in the two cores sampled at 22 m water depth (*ca* 10 000 years bp) approximately coincides with the time span over which these stations were flooded (*ca* 9500 years bp).

In the Gulf of Trieste, high sedimentation rates and rates of organic matter burial are limited to the northern (2 to 6 mm year^−1^ around the outflow of the Isonzo River) and the south‐west margin (*ca* 1 to 5 mm year^−1^ in bays of Piran, Koper and Muggia) (Ogorelec *et al*., 1991; Covelli *et al*., 2006). Sedimentation rates tend to be much lower (0·1 to 0·5 mm year^−1^) at offshore sites in the Gulf of Trieste, with the total thickness of Holocene sediments varying between 1 m and 5 m (Trobec *et al*., 2018). Two stations off Piran were sampled in the southern Gulf; Piran 1 is at 22 m water depth and located within the perimeter of the oceanographic buoy operated by the Marine Biology Station at Piran (45·548867°N, 13·550900°E). It is situated close to the northern margin of the Cape Madona depression elongated in a south‐west/north‐east direction, attaining 38 m water depth in the centre (Trobec *et al*., 2017). Piran 2 is *ca* 1 km north of Piran 1, at 22·7 m water depth (45·563200°N, 13·537033°E) (Fig. 1A). Both stations are characterized by carbonate‐rich skeletal muddy sand (muddy detritic bottom in the terminology of Peres, 1967), with total organic carbon (TOC) content below 1·0% and total nitrogen below 0·1% (Faganeli *et al*., 1991; Ogorelec *et al*., 1991). Habitats now at 22 m water depths were flooded *ca* 9500 years ago in the Gulf of Trieste (Fig. 1B; Antonioli *et al*., 2007; Vacchi *et al*., 2016), maximum ingression took place 6000 to 7000 years ago, and sea‐level stabilized at its present‐day position 2000 to 3000 years ago (Fig. 1B; Amorosi *et al*., 2008).

**Figure 2:**
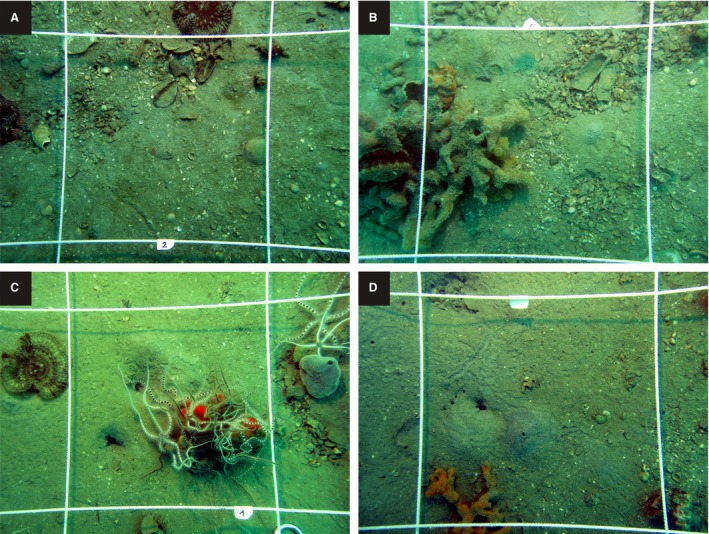
Sea floor photographs from Piran 1 (A) and (B) and Piran 2 (C) and (D) document the relicts of past community states and the former presence of abundant large carbonate producers, now visible as patches with concentrations of large shells that are covered by muddy sand – for example, *Arca noae* in the upper right corner in (B). Epifaunal clumps are formed mainly by sponges and ophiuroids. The size of the square is 25 cm^2^.

Circalittoral habitats offshore from Piran are presently characterized by low light conditions, with epifaunal clumps dominated by ascidians, sponges and ophiuroids (Fedra *et al*., 1976), and sciaphilic algae (Lipej *et al*., 2016), with sediments represented by molluscan sands and gravels (Fig. 2). In contrast, a fishing map from 1927 (Mancini, 1929) shows that the two stations investigated here were located within a *ca* 6 km^2^ patch of *Arca* biostrome in the earliest 20th century and close to a sector formed by sponge‐algal biostromes in the centre of the Gulf. Infralittoral depths with sea‐floor vegetation presently extend down to 8 m, rarely to 10 m, in this region (Orlando‐Bonaca & Mavrič, 2014).

## Methods

### Sampling

In 2013, three cores at each station were collected with an UWITEC piston corer (UWITEC, Mondsee, Austria) with hammer action (Gallmetzer *et al*., 2016), yielding cores with a length of 140 to 150 cm and a diameter of 16 cm and 9 cm. The two 9 cm diameter cores were used for grain size, ^210^Pb and geochemical analyses, and the 16 cm diameter cores for the analysis of molluscan assemblages and for shell dating. The top 20 cm of the cores was divided into 2 cm thick increments; the remainder was divided into 5 cm thick increments. To compare the upper 20 cm with the rest, the 2 cm thick increments were pooled into 4 cm thick increments. Complete valves or fragments with umbo preserved were selected from the >1 mm sieve fraction of each increment, identified to species level and counted. For each species, the higher number of single valves (either right or left) was added to the number of double‐valved specimens to get the final count in each increment. Due to the high shell abundance, the increments from 10 to 35 cm at Piran 2 were split into quarters, and only one quarter was counted. In analyses of absolute abundances, these data were multiplied by four. Similarly, the increments from 35 to 45 cm at Piran 2 were divided in half and then multiplied by two (Table S1).

### Geochronology, time averaging and disorder

**Figure 3:**
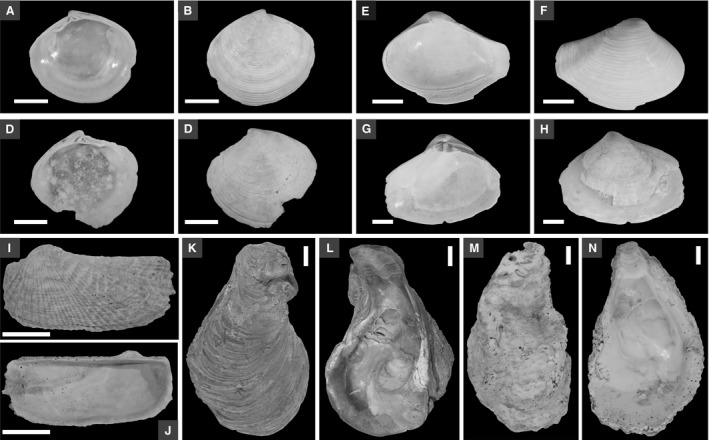
Internal and external sides of right and left valves of *Gouldia minima* (A) to (D) and *Corbula gibba* (E) to (H) from Piran 2, and internal and external sides of large shells of *Arca* and *Ostrea* used in dating of the shell bed. (A) and (B) Right valve, UAL10782T (0 to 2 cm at Piran 2). (C) and (D) Left valve, UAL10782Z (0 to 2 cm at Piran 2). (E) and (F) Right valve, UAL 12737 (145 to 150 cm at Piran 2). (G) and (H) Left valve, UAL 12735 (145 to 150 cm at Piran 2). Scale in (A) to (H) = 1 mm. (I) and (J) *Arca noae*. (10 to 12 cm at Piran 1). (K) and (L) *Ostrea* sp. (20 to 25 cm at Piran 1). (M) and (N) *Ostrea* sp. (55 to 60 cm at Piran 1). Scale in (I) to (N) = 1 cm.

Core chronology is based: (i) on the radiocarbon‐calibrated amino acid racemization (AAR) dating of 575 shells of *Gouldia minima* (Piran 1 and 2) and 247 shells of *Corbula gibba* (Piran 2) that are similar in size (up to 11 mm in length; Fig. 3), collected at centimetre‐scale stratigraphic resolution throughout the whole core; (ii) on radiocarbon ages of ten large shells (up to 8 cm in length) of *Arca noae* and *Ostrea* sp. collected in the shell bed in the upper part of cores at both stations; and (iii) on the ^210^Pb activity profile of near‐surface sediment. Thirty specimens (complete valves or fragments with umbo preserved) of *G. minima* were randomly selected from each of nine, more or less evenly spaced core increments at Piran 1 and from each of eleven increments at Piran 2 from the >1 mm sieve fraction. Similarly, 20 to 30 specimens of *C. gibba* were selected from each of nine increments at Piran 2. Five hundred and sixty‐four dead shells of *G. minima* and 232 dead shells of *C. gibba* passed the AAR screening criteria of Kosnik & Kaufman (2008) (Tables S2 and S3).

Thirteen shells of *G. minima*, five shells of *C. gibba*, five shells of *A. noae* and five shells of *Ostrea* sp. were ^14^C‐dated at the Poznan Radiocarbon Laboratory (Poznan, Poland) (Table 1). Radiocarbon ages were calibrated to calendar years using Calib7·1 (Stuiver & Reimer, 1993), Marine13 data (Reimer *et al*., 2013) and a correction from a marine reservoir (ΔR) age in the north‐east Adriatic Sea of −61 years (Siani *et al*., 2001). The extent of AAR in shells of *G. minima* and *C. gibba* was analyzed with reverse phase high pressure liquid chromatography (RP‐HPLC) (Kaufman & Manley, 1998). The rate of AAR was calibrated using the Bayesian model fitting procedures according to Allen *et al*. (2013). Tomašových *et al*. (2017) presented calibration of AAR in *C. gibba* shells by ^14^C using the time‐dependent reaction kinetic model (TDK1; Allen *et al*., 2013) for aspartic acid (Asp) D/L (ratio of dextrorotatory and levorotatory enantiomers), with the initial D/L value estimated from data. The calibration equation for *C. gibba* is; *a*(arctanh([DL − DL_0_]/[(1 − DL)DL_0_])^*b*^), where DL is Asp D/L, DL_0_ is Asp D/L at 0 years (here, 0·02799), and *a* = 87855·52 and *b *=* *2·531196.

**Table 1:** Numerical ages of *Gouldia minima, Arca noae* and *Ostrea sp*. used in AMS‐AAR (accelerator mass spectrometry – amino acid racemization) calibration with D/L of the aspartic acid (Asp) and glutamic acid (Glu)

Species	Specimen ID	Poznan ID	Conventional ^14^C age (bp)	Conventional ^14^C age error (2 SD)	Calibrated age (to 2013 ad)	Lower 95% conf. bound on calibrated age	Upper 95% conf. bound on calibrated age	Asp D/L	Glu D/L
*Gouldia minima*	Piran 1‐0‐2 cm‐26	Poz‐69883	565	61	332	487	164	0·102	0·054
*Gouldia minima*	Piran 1‐8‐10 cm‐02	Poz‐69884	490	58	239	350	104	0·137	0·053
*Gouldia minima*	Piran 1‐20‐25 cm‐03	Poz‐69885	4225	61	4467	4640	4281	0·297	0·095
*Gouldia minima*	Piran 1‐20‐25 cm‐05	Poz‐69886	4285	61	4548	4753	4362	0·313	0·100
*Gouldia minima*	Piran 1‐20‐25 cm‐11	Poz‐69887	2945	58	2846	2986	2746	0·273	0·086
*Gouldia minima*	Piran 1‐40‐45 cm‐01	Poz‐69888	4365	61	4663	4848	4493	0·298	0·103
*Gouldia minima*	Piran 1‐40‐45 cm‐08	Poz‐69894	5510	71	6031	6230	5857	0·330	0·107
*Gouldia minima*	Piran 1‐60‐65 cm‐02	Poz‐69895	4325	61	4604	4799	4456	0·293	0·092
*Gouldia minima*	Piran 1‐60‐65 cm‐05	Poz‐69896	3200	58	3147	3319	2970	0·258	0·063
*Gouldia minima*	Piran 2‐8‐10 cm‐01	Poz‐69952	995	58	685	779	587	0·172	0·043
*Gouldia minima*	Piran 2‐8‐10 cm‐07	Poz‐69953	3035	58	2934	3089	2805	0·270	0·091
*Gouldia minima*	Piran 2‐8‐10 cm‐10	Poz‐69954	2040	61	1742	1892	1593	0·220	0·096
*Gouldia minima*	Piran 2‐25‐30 cm‐11	Poz‐69955	3360	61	3351	3504	3195	0·247	0·107
*Gouldia minima*	Piran 2‐alive (G5‐2)	NA	NA	NA	1	2	0·1	0·039	0·019
*Gouldia minima*	Piran 2‐alive (5‐7)	NA	NA	NA	1	2	0·1	0·038	0·018
*Arca noae*	Piran 1‐10‐12 cm	Poz‐96309	2505	61	2296	2434	2125	NA	NA
*Ostrea sp*.	Piran 1‐12‐14 cm	Poz‐96310	6200	64	6777	6944	6613	NA	NA
*Arca noae*	Piran 1‐16‐18 cm	Poz‐96311	5810	64	6353	6494	6229	NA	NA
*Ostrea sp*.	Piran 1‐20‐25 cm	Poz‐96312	6230	64	6813	6984	6661	NA	NA
*Ostrea sp*.	Piran 1‐55‐60 cm	Poz‐96313	6970	71	7582	7710	7472	NA	NA
*Ostrea sp*.	Piran 1‐18‐20 cm	Poz‐98391	5775	61	6317	6454	6183	NA	NA
*Ostrea sp*.	Piran 1‐16‐18 cm	Poz‐98393	5420	61	5918	6058	5758	NA	NA
*Arca noae*	Piran 2‐25‐30 cm	Poz‐98394	4530	61	4869	5028	4673	NA	NA
*Arca noae*	Piran 2‐12‐14 cm	Poz‐98395	4725	61	5126	5318	4936	NA	NA
*Arca noae*	Piran 2‐2‐4 cm	Poz‐99101	3525	61	3545	3695	3405	NA	NA

The specimen ID corresponds to the unique specimen identification number, Poznan ID is the unique identification number for the radiocarbon analyses from the Poznan Radiocarbon Laboratory. Calibrated age (years) is relative to 2013 ad (the year of sampling). *Corbula gibba* ages are listed in Tomašových *et al*. (2017). Radiocarbon ages were calibrated to calendar years with the Marine13 data and a correction from a marine reservoir (ΔR) age in the north‐east Adriatic Sea of ‐61 years (SD = 50 years). NA, not available.

**Figure 4:**
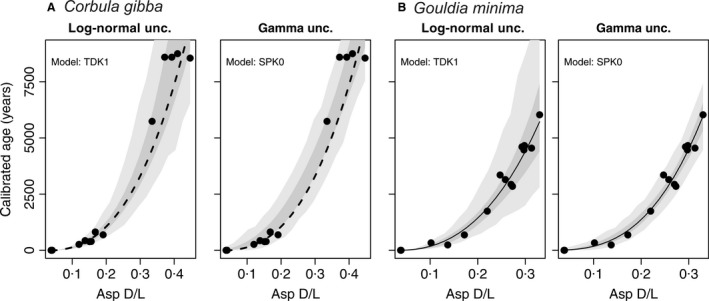
Relations between Asp D/L and ^14^C calibrated shell ages (before ad 2013) for *Corbula gibba* (A) and *Gouldia minima* (B), assuming log‐normal and gamma distributions for the residuals, including data from live‐collected specimens as calibration data points. Light‐grey envelopes correspond to 95% prediction intervals for the age of a given specimen; dark‐grey envelopes correspond to 95% confidence intervals for mean age.

The uncertainty of shell age estimates is defined by the log‐normal distribution, with the mean equal to age estimate and the variance equal to 0·056. The calibration of AAR in *G. minima* shells, based on 13 shells dated by ^14^C and on two live collected shells, is presented here for the first time (Table 1). It is based on the simple power‐law kinetics model (SPK0) for Asp D/L, with the initial D/L value set to zero (Fig. 4). The uncertainty of shell age estimates is defined by the gamma distribution, with the shape parameter equal to 51·16437. The calibration equation for *G. minima* is *a*DL^*b*^, where *a *=* *156 680·5 and *b *=* *2·946717. The AAR‐calibrated calendar ages are set relative to the year of sampling (2013 ad = year zero).

Activities of ^210^Pb and ^226^Ra were analyzed in 2 cm thick intervals in the upper 20 cm, and in 5 cm thick intervals between 20 cm and 40 cm by gamma spectrometry with a High Purity Germanium detector system (Canberra Industries) at the low level counting laboratory Arsenal in Vienna, Austria. Apparent sediment‐accumulation rates were computed from the slope of the decay in excess ^210^Pb according to the constant flux–constant sedimentation model (CFCS; Sanchez‐Cabeza & Ruiz‐Fernández, 2012). The surface fully‐mixed layer that is *ca* 4 cm thick at both stations was excluded. The profiles were terminated at 10 or 14 cm depth at Piran 1 and 2 where the excess ^210^Pb activity falls to the supported values. Due to these effects of mixing, the ^210^Pb excess profiles are primarily used here to determine the maximum depth to which sediment particles deposited during the 20th century were buried. The concentrations of TOC and total nitrogen were measured with an elemental analyzer (CHN 2400; Perkin Elmer, Waltham, MA, USA). The estimation of concentrations of organic pollutants (PCB) was described by Vidović *et al*. (2016).

Time averaging was estimated with inter‐quartile range (IQR) of *G. minima* and *C. gibba* ages. The component of time averaging expected purely due to the calibration error was computed by estimating an IQR for each shell expected under a repeated sampling of shell ages from log‐normal distribution (*C. gibba*) and gamma distribution (*G. minima*). The mean IQR of all shells in a given increment represents the component of time averaging expected purely under calibration error. The time averaging corrected for this error refers to the difference between the raw IQR on one and the error component on the other hand (Dominguez *et al*., 2016; Ritter *et al*., 2017). Stratigraphic disorder was estimated as a Spearman rank correlation between shell ages and their stratigraphic position (Cutler & Flessa, 1990).

**Figure 5:**
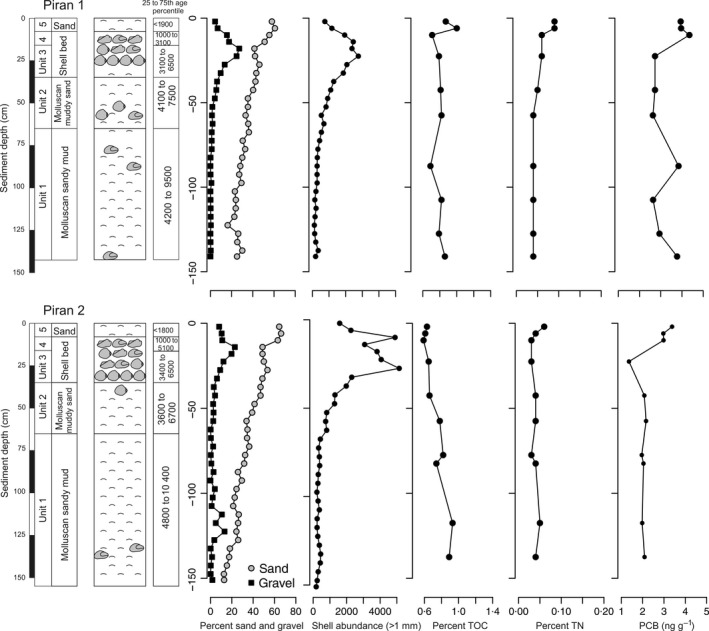
Lithological sections at the Piran stations show an upward increase in the proportion of sand and gravel, peaking in a marked shell‐gravel bed at both stations in the uppermost part (at Piran 1, an additional shell‐rich interval with large bivalves occurs between 45 cm and 90 cm), as detected by volumetric percentages of sand and gravel fractions and by total abundance of molluscan specimens in the fraction >1 mm. The top of the core is formed by molluscan sand with dispersed coralline algae. The percentage of total organic carbon (TOC) increases up‐core at Piran 1 in the upper 8 cm. The percentages of total nitrogen (TN) and organic pollutants (polychlorinated biphenyls, PCB) increase up‐core in the uppermost 5 to 10 cm at both stations. Ages refer to years before ad 2013.

**Figure 6:**
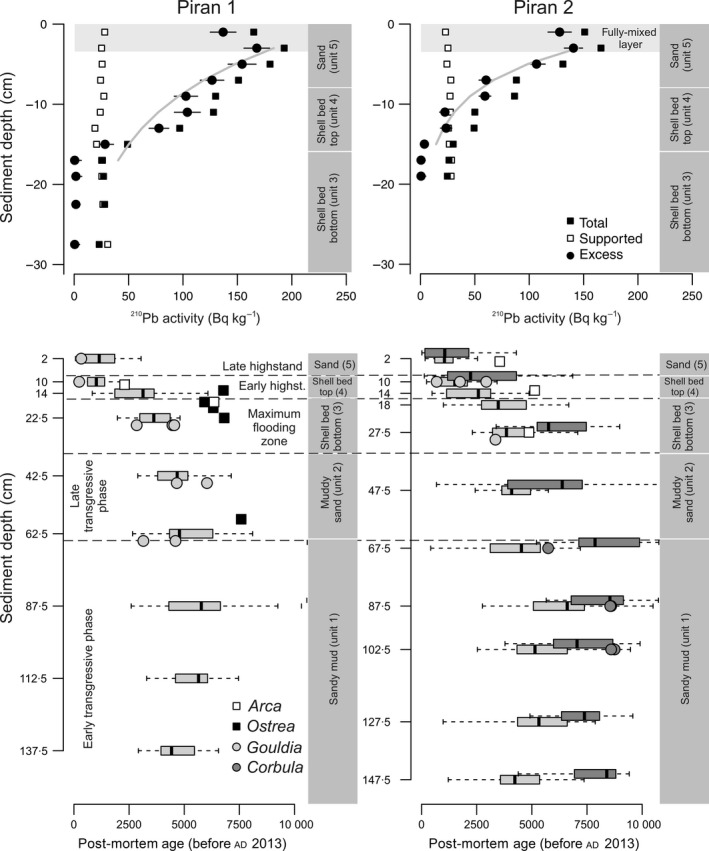
Summary of ^210^Pb activity and radiocarbon ages (top row) and radiocarbon‐calibrated amino acid racemization (AAR) ages (bottom row) of *Gouldia minima* (light grey boxplots) and *Corbula gibba* (dark grey boxplots) in sediment cores collected at Piran 1 and Piran 2. Radiocarbon ages of individual shells are shown separately in light‐grey circles (*G. minima*), dark‐grey circles (*C. gibba*), black squares (*Arca noae*) and white squares (*Ostrea* sp.). Boxplots show median ages and 25th and 75th percentiles, whiskers denote age minima and maxima.

**Figure 7:**
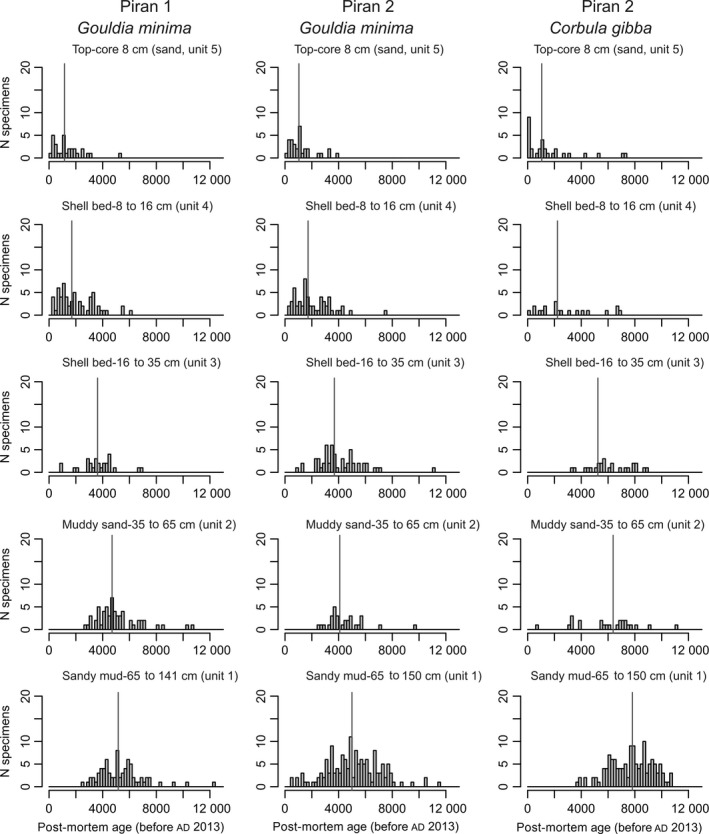
Down‐core age‐frequency distributions of *Gouldia minima* at the two Piran stations and of *Corbula gibba* at Piran 2 in 200 year cohorts and in five stratigraphic units. They shift from right‐skewed distributions in the upper 8 cm to less skewed, normal‐shaped distributions in the middle and lower parts of cores. Vertical lines represent median ages.

**Figure 8:**
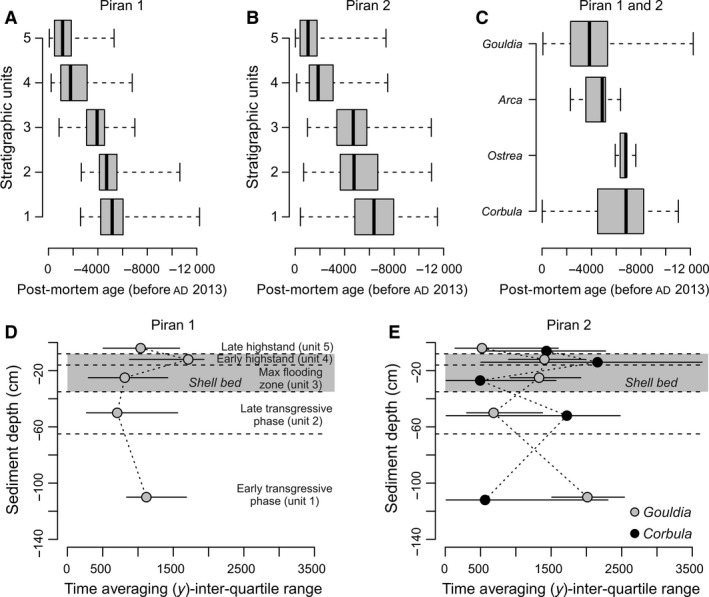
(A) and (B) Down‐core changes in age distributions in five stratigraphic units (based on all shell ages of *G. minima, A.noae* and *Ostrea* sp. at Piran 1, and *C. gibba, G. minima* and *A.noae* at Piran 2) show that median age monotonically declines but the units overlap in their 25th and 75th age percentiles. (C) Boxplots summarizing age distributions of four species in both cores. (D) and (E) Down‐core changes in time averaging of *Gouldia minima* and *Corbula gibba* at the two Piran stations. Inter‐quartile ranges of shell ages are corrected for calibration error and are thus smaller than the differences between the 25th and 75th age percentiles in (A) and (B). Time averaging of *G. minima* remains rather constant, whereas time averaging of *C. gibba* tends to increase towards the upper parts of the core at Piran 2.

The cores show similar stratigraphic distribution of sediment types (Fig. 5), with the same position of the shell bed at 8 to 35 cm, and similar down‐core changes in age of *G. minima* (Fig. 6). These cores can be subdivided into five units on the basis of sediment and geochemical composition (Figs 5 and 6). The overall stratigraphic subdivision of both cores to five stratigraphic units is based on the pooled distributions of all shell ages. These units are characterized by substantial overlaps in age and by millennial‐scale time averaging of molluscan shells (Figs 7 and 8). Therefore, rather than using discrete estimates of average age, this study uses the 25th and 75th age percentiles to summarize their age range (Fig. 8A and B).

### Age unmixing based on dated shells

This approach consists of three steps (Tomašových *et al*., 2017). First, the shape of age‐frequency distributions (AFD) of undated increments was interpolated by pooling distributions of dated increments below and above into a single distribution. The AFDs in individual increments below the upper part of the shell bed are normal‐shaped, allowing approximation of their shape with a normal distribution (truncated on zero years so that negative ages are not allowed; Jackson, 2011). Second, shell ages were resampled to the total number of shells of *C. gibba* and *G. minima* in each increment, either from increment‐specific or from interpolated AFDs (using the mean and standard deviation estimated on the basis of the pooled distributions from the underlying and overlying increments) and the number of resampled shells in 50 or 200 year cohorts was counted. Abundance of shells of a given age predicted by these two steps does not account for shells that were lost by disintegration in the taphonomic active zone (TAZ) or by burial below the core. Sediment increments at the base of cores are enriched in brackish species (*Cerastoderma edule* and *Hydrobia acuta*) and it was assumed that the burial of marine shells to depths below the core is negligible. Therefore, in the third step, loss rate of shells from the TAZ was estimated by fitting AFDs of shells from the topmost 8 cm to two preservation models presented in Tomašových *et al*. (2014), and the predicted number of preserved shells (at the resolution of 50 and 200 year cohorts per 0.02 m^2^) was divided by the survival function of a best model (Tomašových *et al*., 2016). The one‐phase exponential model assumes that disintegration rate does not vary with shell age in the TAZ whereas the two‐phase exponential model allows for an age‐dependent decline in shell loss rate (from *λ*_*1*_ to *λ*_*2*_). The decline in the probability of loss with increasing shell age can be caused by temporary burial below the TAZ and/or by diagenetic stabilization (parameter *τ*) (Tomašových *et al*., 2014).

The two‐phase exponential model fitted to the AFD of *C. gibba* from the top 8 cm outperforms the one‐phase exponential model [corrected Akaike information criterion (AIC) of one‐phase model = 1008·5; two‐phase model = 997·5]. The shell loss rate from the TAZ corresponds to a half‐life of *ca* 110 years (*λ*_1_ = 0·008) and the shift to higher shell durability (*λ*_2_ = 0·0005) occurs over *ca* 320 years (*τ* = 0·0022). These estimates assume that the shell production was approximately constant over the past *ca* 100 years (Tomašových *et al*., 2016). The order of magnitude estimate of disintegration is in accord with AFDs observed in the northern Gulf of Trieste where the loss of shells from the TAZ is primarily governed by shell burial below the TAZ that occurs over 20 to 30 years and disintegration of *C. gibba* is thus slower than burial (Tomašových *et al*., 2017). Cohorts younger than *ca* 100 years are infrequent in the AFD of *G. minima*, and the AFDs for this species are thus not informative about disintegration rates in the TAZ. Therefore, estimates of disintegration rates based on *C. gibba* in the upper 8 cm were also used in reconstructing the production of *G. minima*. Due to the uncertainties in the estimates of shell disintegration rates, the unmixed trajectories in production for both scenarios are shown here, including: (i) no or negligible shell loss in the TAZ (i.e. avoiding the third step in age unmixing); and (ii) shell loss in the TAZ.

### Compositional differences between subsurface and living assemblages

The AAR data demonstrate that molluscan assemblages in the topmost increments show millennial‐scale time averaging and thus can be inert to the most recent changes in ecosystem composition. Therefore, the composition of molluscan communities found in the topmost intervals of sediment cores (upper 12 cm) was compared with two living assemblages sampled at Piran 1 and Piran 2 in 2014, each with eight Van Veen grabs (0·125 m^2^). Pairwise Bray‐Curtis dissimilarities were computed among all core assemblages and living assemblages using square‐root‐transformed proportional species abundances, and principal coordinate analyses (PCO) were performed separately for the two stations. Non‐parametric permutational multivariate analysis of variance (permanova; Anderson, 2001) was used to test for differences in composition between stratigraphic units. To increase the spatial coverage of communities collected alive, the core assemblages and the two living assemblages from 2014 were finally compared with additional living assemblages collected in 2011 off Piran at 12 stations at 20 m water depth. At each station, three Van Veen grabs (0·1 m^2^) were sampled in June and September and sieved with a mesh size of 1 mm. Abundances of all species were pooled across grabs and months (Table S4).

## Results

### Stratigraphic subdivision of cores

Although separated by *ca* 1 km, sediment cores at both stations show very similar trends in grain size and shelliness (Fig. 5); they show a coarsening‐upward trend, with decreasing proportion of siliciclastic mud, increasing proportion of bioclastic sands and shell gravel, and increasing abundance of shells larger than 1 mm (Fig. 4). At both stations, the sediment is primarily formed by skeletal (mainly molluscs, echinoderms and foraminifera) sandy mud in the lower core part (65 to 141 cm at Piran 1 and 65 to 155 cm at Piran 2), with a gradually decreasing proportion of mud, shifting to 30 cm thick muddy mollusc‐rich sands (35 to 65 cm at both sites). A densely packed and poorly sorted shell bed formed by randomly oriented shells of large epifaunal bivalves (*Ostrea* in the lower part and *Arca* and *Modiolus* in the upper part) occurs at 8 to 35 cm at both stations. In addition, at Piran 1, a second shell‐rich interval with abundant dispersed shells of *Ostrea* and *Modiolus* occurs between 45 cm and 95 cm. The upper shell beds are buried below an 8 cm thick mollusc‐rich sand with dispersed coralline red algae (*Litothamnion*), although sea‐floor photographs show that they are locally directly exposed on the surface, and patchily intergrade with mollusc‐rich sandy sediments (Fig. 5). Piran 1 shows higher TOC in the upper 8 cm whereas Piran 2 shows an overall decline of TOC towards the top. At both stations, total nitrogen increases in the uppermost 5 to 10 cm (Fig. 4).

### Down‐core changes in age distributions

The ages of bivalve shells reveal that the core captures most of the marine ecosystem history since the sea floor at *ca* 20 m was flooded *ca* 9500 years ago (Fig. 6). Five stratigraphic units that differ in sediment geochemical composition correspond to different phases in sea‐level history: (i) the early transgressive unit 1 at 65 to 150 cm formed by sandy muds (25th and 75th age percentiles span 4800 to 8000 years at Piran 2, with the oldest *C. gibba* ages equal to 10 400 years); (ii) the late transgressive unit 2 at 35 to 65 cm formed by muddy sands with dispersed oysters (25th and 75th age percentiles span 3600 to 6700 years at Piran 2); and (iii) the transitional maximum‐flooding zone (unit 3) represented by a lower part of the gravelly shell bed at 16 to 35 cm dominated by *Ostrea* sp. (25th and 75th age percentiles span 3400 to 5800 years, and maximum ages of oysters to 6800 years); (iv) the upper part of the gravelly shell bed (unit 4) with abundant *Arca noae* and *Modiolus barbatus* (1100 to 3100 years); and (v) the top 8 cm (unit 5) formed by molluscan sands (<1800 to 1900 years).

The median age of *G. minima* in the uppermost 0 to 2 cm is *ca* 1000 to 1200 years at both stations, and the median age of *C. gibba* is 1000 years at Piran 2. *Corbula gibba* sampled in the upper 8 cm shows a strongly right‐skewed AFD, with the mode at 14 years and the median at 1000 years (Fig. 7). The AFDs of *G. minima* in the uppermost 8 cm are right‐skewed but the most frequent cohorts are older than 250 years, with modes at 340 years (Piran 1) and 1060 years (Piran 2), and median ages at 1200 years (Piran 1) and 1100 years (Piran 2) (Fig. 7). Uniform values of the ^210^Pb excess occur in the upper 4 cm at both stations (Fig. 6). The background values appear within the shell bed at 10 to 14 cm. In the absence of mixing, the profiles would correspond to sedimentation rates equal to 0·29 cm year^−1^ (Piran 1) and 0·16 cm year^−1^ (Piran 2). However, down‐core changes in median shell ages indicate that long‐term sedimentation rates are at least one order of magnitude lower on the basis of the whole 150 cm deposited over 10 000 years (*ca* 0·015 cm year^−1^) or based on the upper 15 to 20 cm (*ca* 0·005 cm year^−1^).

The *Ostrea*–*Arca* shell bed shows a monotonic downward increase in median age of *G. minima* (with 1000 to 1600 years at 8 to10 cm; 2600 to 3000 years at 12 to 14 cm; 3500 years at 16 to 18 cm; to *ca* 4000 to 4700 years at 20 to 30 cm) and *C. gibba* (with 2200 years at 8 to 10 cm; to *ca* 5800 years at 25 to 30 cm), spanning the transition from the maximum‐flooding zone to the late highstand unit (Fig. 6). The AFDs of both species shift from right‐skewed distributions in the upper 8 cm to more symmetrical, normal‐shaped distributions in the rest of the core (Fig. 7). The ages of large *Ostrea* (6000 to 6800 years) and *Arca* (4900 to 6400 years) specimens in the lower part of the shell bed are similar to the ages of *Corbula* (5200 to 7500 years at 25 to 30 cm at Piran 2), in contrast to younger *Gouldia* in the same increment (3000 to 4400 at 25 to 30 cm at Piran 1 and 3200 to 5000 at 20 to 25 cm at Piran 2). The cluster of oyster ages at the base of the shell bed approximately coincides with maximum flooding (6000 to 6500 years ago). Median ages of *G. minima* (4500 to 6600 years) are younger by >2000 years compared to median ages of co‐occurring *C. gibba* (7100 to 8300 years) between 65 cm and 150 cm at Piran 2. Pooling all shells into whole‐core distributions, shells of *G. minima* are on average markedly younger than shells of *C. gibba* (Fig. 8C).

### Down‐core changes in time averaging and in stratigraphic disorder

Time averaging (IQR) of both species does not vary systematically with sediment depth and between transgressive and highstand sediments at Piran 1 (Fig. 8D and E). Down‐core trends in time averaging of *G. minima* and *C. gibba* are also variable at Piran 2. Time averaging of *G. minima* in 4 to 5 cm thick increments peaks in the lowermost part of the cores in the early transgressive interval (*ca* 1000 to 2000 years) and in the shell bed (*ca* 1500 years) and reaches *ca* 500 to 1000 years in the rest of the core. Time averaging of *C. gibba* attains *ca* 500 years in the early transgressive unit and in the lower part of the shell bed, and *ca* 2000 years in the upper part of the shell bed.

Median ages of *G. minima* show a reversal at 115 to 120 cm and 135 to 140 cm at both stations, where they are younger than at 90 cm. In unit 1 (65 to 150 cm), shell ages of *G. minima* do not correlate with sediment depth at both stations [Piran 1 (Spearman *r* = 0·14, *P* = 0·17); Piran 2 (Spearman *r* = 0·08, *P* = 0·36)] and in *C. gibba* at Piran 2 (Spearman *r* = 0·14, *P* = 0·11), demonstrating extensive age homogenization and vertical displacement of some shells by *ca* 1 m in transgressive sediments. In the upper part of the cores (0 to 65 cm), however, post‐mortem ages significantly correlate with sediment depth in *G. minima* at both stations [Piran 1 (Spearman *r* = −0·72, *P* < 0·001), Piran 2 (Spearman *r* = 0·08, *P* < 0·001)] and in *C. gibba* at Piran 2 (Spearman *r* = −0·75, *P* < 0·001).

### Stratigraphic trends in abundance

**Figure 9:**
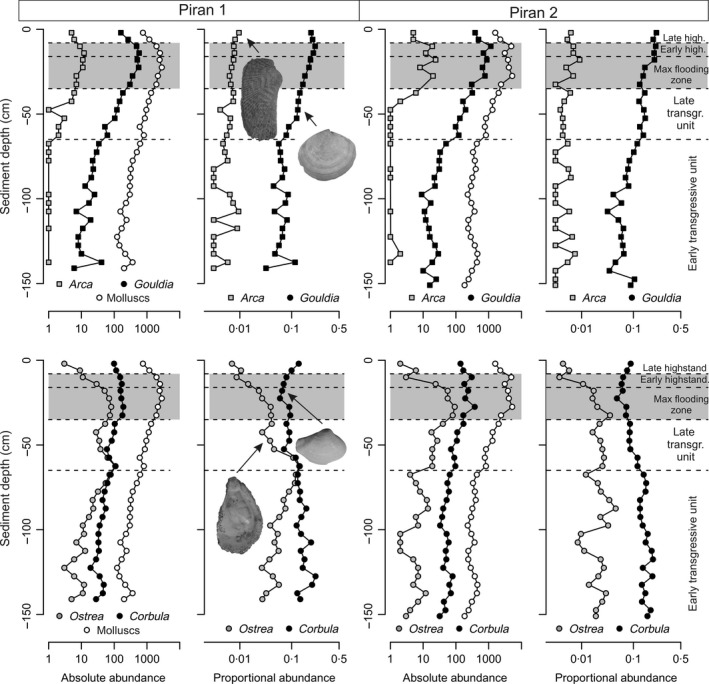
Proportional abundances of *C. gibba* generally decrease towards the top of the core (with a slight reversal in the upper 8 cm) whereas proportional abundances of *G. minima* increase towards the top of the core at Piran stations. In contrast, absolute abundances of both species increase towards the top of the core, although abundances of *G. minima* are smaller in transgressive sediments and increase at much higher rates. Absolute abundances of *G. minima* show a slight decline in the uppermost two increments.

**Figure 10:**
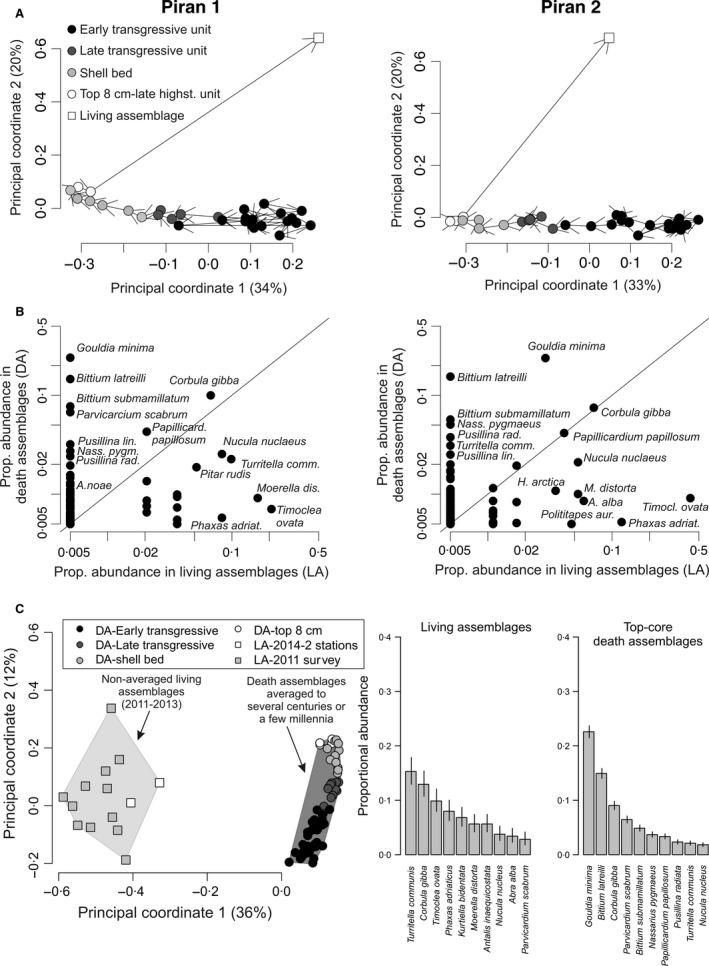
(A) Principal coordinate analyses based on square‐root transformed proportional abundances show significant differences between core assemblages and a living assemblage (LA) at the two Piran stations. (B) Proportional abundances of *G. minima* and herbivorous gastropods (*Bittium* and *Pusillina*) are significantly higher in top‐core death assemblages (DA) than in living assemblages at the two Piran stations. (C) Principal coordinate analysis showing a major difference in composition between assemblages from the two cores and 14 living assemblages sampled by Van Veen grabs in 2011 and 2014 (including two living assemblages collected at Piran 1 and Piran 2). Two barplots on the right show proportional abundances of ten most common molluscan species in the pooled living assemblages and top‐core death assemblages, with 95% confidence intervals.

The total abundance of molluscs (shells >1 mm) increases markedly towards the top of both cores, peaking in the shell bed at 8 to 25 cm at Piran 1 and at 8 to 12 cm at Piran 2 (Fig. 9). The transition at 65 cm marks a change from sandy muds to muddy sands and coincides with major changes in community composition, including the appearance of large *Ostrea* sp. (Fig. 9), a shift along the PCO axis 1 (Fig. 10), and differences between units 1 and 2 in permanova [*F* (Piran 1) = 6·6, *P* < 0·001; *F* (Piran 2) = 8·4, *P* < 0·001].

In *G. minima* and *A. noae*, stratigraphic trends in absolute abundances are similar to trends in proportions. Both species generally show an upward increase and achieve maximum abundance in the upper parts of the shell bed. Assemblages of *G. minima* consist of about five to ten individuals in the lowermost increments, rising to 576 individuals at 12 to 16 cm at Piran 1 and to 1158 individuals at 8 to 12 cm at Piran 2. The uppermost 8 cm are characterized by a decline in abundance, with 160 individuals at 0 to 4 cm at Piran 1 and 385 individuals at 0 to 4 cm at Piran 2 (Fig. 9). Proportional abundances of *G. minima* show a similar increase, rising from 5% in the lower parts to 20 to 25% in the upper parts. Proportional abundances of *A. noae* increase from 0·1 to 1·0%, and absolute abundances increase from zero in the lower parts of cores to about ten individuals in the upper shell bed.

In *C. gibba* and *Ostrea* sp., absolute abundances increase upward and peak in the shell bed whereas proportional abundances peak in the lower and middle parts of cores. Proportional abundances of *C. gibba* decrease at 65 cm from 20% to 5 to 10%, and again increase in the uppermost 10 cm to 10 to 15%. Absolute abundances of *Ostrea* sp. increase from about ten individuals in the lower parts of cores, peak in the lower part of the shell bed (50 to 100 individuals), and decline in the uppermost increments (less than five individuals). Proportional abundances of *Ostrea* sp. fluctuate between 5% and 10% in the lower and middle parts. A major decline in *Ostrea* sp. in the uppermost 16 cm is detected by both absolute and proportional abundances.

### Unmixed trends in abundance

**Figure 11:**
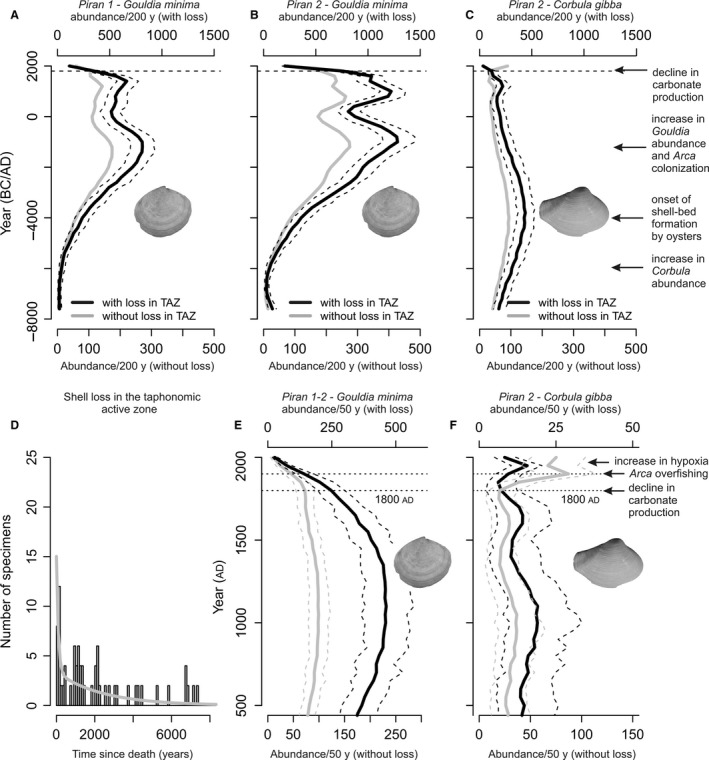
(A) to (C) Unmixed abundance trends of *G. minima* at both Piran stations (A) and (B) and of *C. gibba* at Piran 2 in 200 year cohorts, with (black) and without (grey) accounting for shell loss in the taphonomic active zone (TAZ). (D) Age‐frequency of distribution of *C. gibba* used for modelling shell loss in the TAZ. (E) and (F) Unmixed abundance trends of *G. minima* and *C. gibba* at the resolution of 50 year cohorts (both Piran stations pooled). *Corbula gibba* attained maximum abundance during the late transgressive phase (6000 to 7000 years ago) and increased again in the 20th century. *Gouldia minima* attained maximum abundance during the highstand phase (<5500 years ago) and abruptly declined in abundance since the 19th century. The main events in the history of molluscan communities are shown on the left with arrows.

The age unmixing procedure shows that abundances of *G. minima* increased *ca* 5500 years ago and peaked during the highstand phase while those of *C. gibba* peaked *ca* 6000 to 7000 years ago and gradually declined over the past 5000 years (Fig. 11). Thus, the unmixed trends of *G. minima* generally capture unmixed stratigraphic trends in absolute and proportional abundances, with the exception of the strong most recent decline that is not detected by raw stratigraphic patterns. The unmixed trends of *C. gibba* are more similar to proportional trends than to absolute trends. Increasing the resolution to 50 year cohorts and focusing on the most recent decline in production of *G. minima*, the decline in abundance of this species occurred over the past two centuries (Fig. 11). *Corbula gibba* shows an increase in the 20th century, although this increase is subdued in the model accounting for loss in TAZ.

Age unmixing predicts 500 to 1000 individuals of *G. minima* per 200 years per 0·02 m^2^ during the highstand phase (standing abundance of *ca* 125 to 250 individuals per m^2^ assuming a one‐year lifespan, or 250 to 500 individuals per m^2^ assuming that mean per‐individual lifespan is two years), and *ca* 400 individuals of *C. gibba* per 200 years per 0·02 m^2^ during the late transgressive phase (100 individuals per m^2^ assuming a one‐year lifespan, or 200 individuals per m^2^ assuming a two‐year mean lifespan). The estimated standing abundance of *G. minima* during the highstand phase alone is larger than total standing abundances of all molluscs collected in living assemblages at 14 stations sampled in 2011 and 2014. Present‐day standing densities of all molluscs are invariably smaller than 200 individuals per m^2^ at all stations.

### Differences between living and sea floor death assemblages

At both stations, living molluscs collected in the earliest 21st century and death assemblages collected from topmost 12 cm of cores are separated in the PCO space (Fig. 10). Living assemblages (LA) collected at Piran 1 and Piran 2 are dominated by five species that are less frequent in death assemblages (DA) at both stations, including *Tellina distorta* (5 to 6% in LA at Piran 1 and 2 versus <1% in DA at both stations)*, Phaxas adriaticus* (8 to 11% versus <1%)*, Nucula nucleus* (5 to 8% versus 2%), and *Timoclea ovata* (20 to 42% versus <1%). Death assemblages in the topmost core sediment are dominated by *G. minima* (23 to 24%) whereas this species does not occur alive at Piran 1 (*n* = 62) and makes up only 2·5% of the LA (4 out of 162) at Piran 2 (Fig. 10). Death assemblages are further dominated by the herbivorous gastropods *Bittium latreilli, Bittium submamillatum, Pusillina lineolata* and *Pusillina radiata. *These four gastropod species together account for 26% and 25% of total abundance in top‐core death assemblages at Piran 1 and Piran 2, respectively, and are completely absent in LA*. *In contrast to *G. minima*,* C. gibba* is less under‐represented in LA, constituting 6 to 7% of living individuals (4 out of 62, and 11 out of 162) at both Piran stations, and 15% of dead individuals at Piran 1 (965 out of 6651) and 12% at Piran 2 (400 out of 3342). Principal coordinate analyses based on 12 additional living assemblages show a significant segregation among all living and late‐highstand core assemblages (Fig. 10; permanova *F* = 12·1, *P* < 0·001), with *Turritella communis, Corbula gibba, Kurtiella bidentata, Antalis inaequicostata, Phaxas adriaticus* and *Tellina distorta* being the most common species alive (Fig. 11). Therefore, living assemblages collected in 2014 at the two Piran stations are not outliers.

## Discussion

### Slow sedimentation rates

The ^210^Pb‐based sedimentation rates are two orders of magnitude higher than sedimentation rates estimated on the basis of down‐core changes in median shell ages in the top 10 to 15 cm (*ca* 0·005 cm year^−1^ for both species). Although estimates of sedimentation rates on the basis of shells of species that declined in abundance towards the present can be biased downward (Peng & Broecker, 1984), the presence of large oysters dated to *ca* 6000 years at the base of the shell bed indicate that long‐term sedimentation rates correspond to AAR‐based estimates and not to the ^210^Pb estimates. Therefore, the ^210^Pb‐based sedimentation rates are strongly biased upward by bioturbation (Johannessen & Macdonald, 2012; Kuzyk *et al*., 2015). The majority of sedimentary particles with ^210^Pb excess do not extend below 10 cm sediment depth, indicating that the 20th century sedimentary record is mixed with older shells in the uppermost parts, just above the shell bed. The top 10 cm are formed by a mixture of shells several centuries old and sediment particles carrying ^210^Pb that were largely deposited in the 20th century because: (i) under very low sedimentation rates, the excess ^210^Pb profiles should be rather rapidly smeared vertically by bioturbation and thus should be uniform rather than monotonically declining; (ii) the topmost 10 cm of cores are characterized by high concentrations of organic pollutants and total nitrogen (both stations) and TOC (Piran 1) characteristic of 20th century pollution and eutrophication in the Gulf of Trieste (Turk *et al*., 2007); and (iii) higher durability of molluscan shells (with a half‐life exceeding a few decades in the TAZ; see below) relative to ^210^Pb (with half‐life = 22 years) can be expected to generate differential mixing of shells and ^210^Pb tracers. More durable shells can be prone to stronger displacement because they have longer residence time (Barker *et al*., 2007), leading to apparent age differences between shells and ^210^Pb tracers. The upper parts of the cores with the shell bed are strongly condensed. Although the uppermost parts of cores contain some particles reflecting the 20th century deposition, the bulk of surface carbonate sediments is dominated by old shells and is thus not representative of 20th century carbonate deposition.

### Effects of mixing

Although condensation reduces temporal resolution of molluscan assemblages to some degree, the effect of mixing further reduces resolution and affects stratigraphic trends in species abundances. Stratigraphic mixing is documented by: (i) AFD that show millennial‐scale time averaging; (ii) down‐core constancy and reversals in shell median age over more than 80 cm; and (iii) differences in abundance patterns based on raw and unmixed records. First, the scales of time averaging cannot be explained by low sedimentation rate. Assuming that residence time of shells in 5 cm thick increments is exponentially distributed, IQR of shell ages can be predicted as log(3)/inverse of shell burial time. In this case, shell burial time from a 5 cm thick increment to underlying increments would be *ca* 330 years (burial rate = 0·015 cm year^−1^ when integrated over the whole core). Such burial time would generate assemblages with IQR equal to *ca* 370 years, whereas IQRs are typically larger by a factor of 2 to 5. Second, the constancy or even reversal of shell median age of *G. minima* over 80 cm (between 45 cm and 150 cm at Piran 1 and 70 cm and 150 cm at Piran 2) and of *C. gibba* over 80 cm (between 70 cm and 150 cm at Piran 2) shows that the vertical extent of shell mixing exceeded 80 cm and was non‐local to some degree (Meysman *et al*., 2003). Deep bioturbation exceeding 50 cm is produced by shrimps in the Gulf of Trieste (Pervesler & Hohenegger, 2006).

Third, the difference between raw stratigraphic and unmixed trends is informative about the nature of mixing processes and about the joint effects of bioturbation and temporally‐variable production on stratigraphic distributions. Mixing in stratigraphic sections does not only smear off stratigraphic patterns and attenuate their magnitude, but also induces stratigraphic shifts when species abundances increase or decrease markedly down‐core (Bard *et al*., 1987; Manighetti *et al*., 1995; Broecker *et al*., 1999; Löwemark *et al*., 2008). Given that *G. minima* underwent a significant increase and *C. gibba* a decrease in abundance towards the present, such changes in abundance explain within‐species differences between raw stratigraphic and unmixed trends and between‐species age differences between *G. minima* on one hand and *C. gibba* (and *Ostrea* sp. and *Arca noae*) on the other hand, exceeding 1000 to 2000 years within individual increments in most of the core. First, downward burial of numerically abundant young shells of *G. minima* from the upper parts of cores was more likely than upward reworking of less frequent old shells of *G. minima* from the lower parts of cores. Second, upward reworking of numerically abundant old shells of *C. gibba* from the lower core parts was more likely than downward burial of less frequent and young shells of *C. gibba* from the upper core parts. Therefore, abundance of *G. minima* in the lower part is exaggerated by downward mixing from highstand sediments, generating highly averaged *G. minima* assemblages in transgressive sediments. In contrast, the abundance of *C. gibba* in the upper part is exaggerated by upward mixing from transgressive sediments, generating a highly averaged *C. gibba* assemblage in highstand sediments. However, the extent of upward‐reworking of *C. gibba* is probably smaller than the extent of downward‐reworking of *G. minima* because *C. gibba* is age‐congruent with large, shell bed forming *Ostrea* sp. that appears for the first time at high abundance consistently at 30 to 35 cm. Other mechanisms can generate age offsets, such as differences in durability or differential mixing susceptibility of skeletal particles differing in size (Bard, 2001), but *G. minima* and *C. gibba* are similar in size, mineralogy and shell thickness.

### Long‐term changes in molluscan production

The long‐term change in the depositional regime between the transgressive and the highstand phase at both stations was mainly determined by: (i) a decline in the proportion of fine‐grained siliciclastic sediment; and (ii) an increase in the volumetric abundance of molluscan sand and shell gravel (see also Mautner *et al*., 2018). The higher proportion of mud in transgressive sediments and the smaller rate of change in median age imply higher accumulation of siliciclastic mud during the transgressive phase, with molluscan assemblages dominated by *C. gibba* inhabiting protected, low‐energy habitats. Progradation of the Isonzo prodelta commenced *ca* 5000 years ago in the northern Gulf of Trieste (Amorosi *et al*., 2008) but it did not affect deposition in the southern Gulf of Trieste. Mud sedimentation was probably hindered since the onset of recent circulation patterns *ca* 5000 to 6000 years ago that intensified currents flowing towards the west and south‐west and triggered mud winnowing along the eastern side (Mautner *et al*., 2018). The Isonzo plume is deflected towards the west and thus does not contribute to deposition in the southern Gulf of Trieste. This onset of circulation temporally coincides with the switch from the transgressive to the highstand phase (Correggiari *et al*., 1996). It was most likely caused by the broadening of the northern Adriatic basin due to maximum ingression, allowing a larger fetch for winds and a stronger thermohaline circulation (Trincardi *et al*., 1994).

*Corbula gibba* shows an upward increase in absolute abundance but the reconstructions show that its production was highest during the late transgressive phase. The up‐core increase in absolute abundance of *C. gibba* was probably caused by a two‐fold decrease in shell dilution by pelitic siliciclastic sediments, generating the mismatch between raw (increasing) and unmixed (decreasing) absolute abundances. However, the increase in raw abundance of all molluscs is not just caused by reduced sediment dilution. The total molluscan production truly increased towards the highstand phase because: (i) unmixed trajectories show that the production of *G. minima* increased through time and strongly exceeded the production by *C. gibba*; and (ii) large epifaunal bivalves increased in abundance during the late transgressive phase and at times of maximum flooding (*Ostrea*) or later during the highstand phase (*Arca* and *Modiolus*).

Therefore, the temporal decline in rate of siliciclastic sedimentation was associated with a higher molluscan production (probably in response to reduced turbidity and sediment input), with shell bed clumps and with the facilitation of highly productive habitats associated with algal or seagrass vegetation. Carbonate particles associated with vegetated habitats were either transported from shallower vegetation that was probably more extensive in the past than now and/or were produced more closely to shell beds at similar water depths. This scenario indicates that both slow sedimentation and the resulting higher production contribute to the formation of molluscan shell beds in temperate habitats (Kidwell, 1986).

### Timing of the most recent decline in molluscan production

Although the unmixing procedure predicting temporal changes in molluscan production accounts for higher disintegration rate of shells in the TAZ, a temporal increase in shell disintegration rate of *G. minima* towards the present can also generate an apparent decline in abundance of young cohorts. However, this effect is not responsible for the recent decline in abundance of *G. minima*. First, in contrast to *G. minima*, the decline in production is not detected in *C. gibba*. *Corbula gibba* increases in proportional abundance in the uppermost increments, and unmixing indicates that this increase took place in the 20th century. Second, proportions of poorly preserved specimens of *G. minima* do not differ between the top 8 cm and the top of the shell bed (45 to 57% specimens with signs of dissolution in the top core versus 47 to 62% in the top of the shell bed, and 61 to 63% specimens with microbioerosion in the top core versus 38 to 76% in the top of the shell bed).

Age unmixing shows that the production of *G. minima* rapidly declined over the past 200 years (Fig. 11), and that the decline to very low levels during the 19th and 20th century did not have any precedents in its population history over the previous *ca* 5000 years. The effects of natural environmental variability, affecting populations and ecosystems at multiple timescales, can be difficult to disentangle from the effects of anthropogenic impacts. The northern Adriatic Sea was affected by climatic fluctuations at millennial, centennial and decadal scales (Piva *et al*., 2008; Tomašových *et al*., 2017), affecting circulation patterns, nutrient regimes and ecosystem composition. First, however, the magnitude of decline in abundance of *G. minima* relative to its millennial‐scale persistence and abundance indicates that the forcing mechanisms generating the decline differ from natural forcing. Second, the signatures of anthropogenic pollution and eutrophication, i.e. the increase in concentrations of total nitrogen and organic pollutants in the upper 8 to 12 cm (Fig. 5), coincide with the proportional decrease in *A. noae* and *G. minima*, and with the proportional increase in *C. gibba* (Fig. 9). The massive increase in the frequency of hypoxia and mucilages (Deserti *et al*., 2005) and changes in the composition of algal, seagrass and zoobenthic communities were observed in living communities since the 1960s (Simonetti, 1966; Orel *et al*., 1982) and in late 20th century increments of sediment cores (Tomašových *et al*., 2018). However, the timing of decline in *G. minima* and the early 20th century monitoring data indicate that *G. minima* was already rare in the early 20th century.

The rarity of *G. minima* in the northern Adriatic Sea prior to major eutrophication in the late 20th century is confirmed by surveys performed in the early 20th century (Vatova, 1928, 1935) and in the 1960s (Gamulin‐Brida, 1967; Gamulin‐Brida *et al*., 1968). The top‐core assemblages are dominated by species that are rare or absent in living assemblages, and standing abundances of molluscs per m^2^ collected alive are smaller than reconstructed highstand abundances of *G. minima* based on age unmixing. Therefore, trajectories in abundance reconstructed from age unmixing and differences in the species pool composition based on living and core assemblages in ordination analyses indicate that compositional and production changes are unprecedented relative to abundance fluctuations occurring during most of the Holocene highstand phase (Fig. 11).

The decline in abundance of *G. minima* in the southern Gulf of Trieste is rather surprising because this species has a long stratigraphic duration and achieved high abundance in the Paratethys and Mediterranean since the Miocene (Mandic & Harzhauser, 2003; Zuschin *et al*., 2007; Gianolla *et al*., 2010), and possesses a broad present‐day geographic range between 30°N and 60°N in the north‐east Atlantic and in the Mediterranean. Frequent shells of dead *G. minima* were also found in surface death assemblages in the northern Gulf of Trieste (Sawyer & Zuschin, 2010), off Rovinj (Vatova, 1928) and in the Gulf of Venice (Trincardi *et al*., 2011), indicating that the past distributional extent of this species was not limited to Piran stations.

### The loss of vegetated habitats

*Gouldia minima* is a suspension feeder with short and fused siphons, living close to the sediment surface (Ansell, 1961). Although it occurs frequently on non‐vegetated sandy and gravelly habitats in the North Sea and Black Sea, it is highly abundant in macroalgal or seagrass habitats in the Mediterranean Sea (Seneš, 1988; Di Geronimo *et al*., 1990; Zenetos, 1996; Basso & Brusoni, 2004; Albano & Sabelli, 2011), and is closely associated with seagrass remains in Pliocene sediments (Moissette *et al*., 2007). It is abundant in infralittoral algal habitats with *Cystoseira* in the Gulf of Trieste (Pitacco *et al*., 2014) and in seagrass, algal and *Cladocora* habitats in the eastern Adriatic Sea (Šiletić, 2006). Gastropods over‐represented in death assemblages are of the genera *Bittium* (Basso & Corselli, 2002; Russo *et al*., 2002; Albano & Sabelli, 2011) and *Pusillina* (Basso *et al*., 2008), which also occur frequently in seagrass and algal assemblages in the Mediterranean Sea. Large, shell‐bed‐forming species are numerically less frequent than small and numerous bivalves (*G. minima*) and gastropods, but they also do not occur in living assemblages. In contrast to dead‐only species, species that are abundant (*C. gibba*) or over‐represented in living assemblages (*Tellina distorta, Nucula nucleus* and *Phaxas adriaticus*) are infaunal species that tend to prefer organic‐rich sediments (Holmes *et al*., 2002; Nerlović *et al*., 2011).

The Gulf of Trieste is presently characterized by low light conditions, with seagrasses not exceeding to habitats deeper than 6·5 m (Ghirardelli *et al*., 1973). *Posidonia* meadows in the Gulf of Trieste are presently limited to very few and small occurrences in Koper Bay and off Grado (Turk & Bonaca, 2002) and *Zostera marina* meadows are also limited in distribution. Benacchio (1938) showed that seagrass meadows extensively colonized many shallow habitats of the Gulf of Trieste still in the earliest 20th century. However, the dead‐only species (*G. minima* and herbivorous gastropods) indicate that the compositional changes in the southern Gulf of Trieste related to the loss of vegetated habitats took place in the 19th century. This timing is similar to the timing of decline in abundance of epiphytic foraminifera at the Po prodelta that took place also during late 19th century (Barmawidjaja *et al*., 1995). The presence of dead *Posidonia* debris in surface or subsurface sediments on now non‐vegetated sea floor in the Gulf of Venice (Taviani, 1980) and off Grado (Newton & Stefanon, 1982; Orel *et al*., 1982) generally attests to the formerly extensive seagrass meadows that formed in the northern Adriatic Sea since the end of Holocene sea‐level rise (Correggiari *et al*., 1996).

A similar environmental history, with the loss of seagrass meadows and the increase in water turbidity, associated with massive increase in abundance of *C. gibba* and expansion of dark muddy bottoms, occurred in semi‐enclosed basins of the Taranto Sea in the late 20th century (Mastrototaro *et al*., 2008). These basins were largely vegetated and characterized by clean waters in the early 20th century, and the shift to non‐vegetated habitats occurred in the course of just a few decades. It is suggested that a similar loss of water clarity occurred in the Gulf of Trieste due to increasing nutrient loads and industrialization during the 19th century (Ghirardelli *et al*., 1973). The trawling and fishery activities probably contributed to the demise of vegetated ecosystems during the 20th century. However, intense fish exploitation traces back at least to the early 19th century in the northern Adriatic Sea, and the intensity of fisheries increased considerably in the late 19th and early 20th century, with increasing numbers of boats and with replacement of traditional, selective types of nets by unselective and destructive equipment (Botter *et al*., 2006; Fortibuoni *et al*., 2010). Large epifaunal bivalves such as *Arca noae* still co‐occurred with brown algae and seagrasses (*Zostera*) at 10 to 20 m water depths during the mid‐20th century off Rovinj (Gamulin‐Brida, 1967; Seneš, 1988), indicating that similar community types persisted for longer on the western side of the Istrian peninsula.

## Conclusions

Actualistic analyses assume that sedimentological and palaeoecological mechanisms can be inferred based on present‐day observations of carbonate production and sedimentation. However, age unmixing and comparison of sea floor and living assemblages reveal that the rate of molluscan production in the southern Gulf of Trieste declined over the past two centuries due to the loss of species associated with vegetation and loss of epifaunal suspension‐feeders, with present‐day molluscan production being largely dominated by deposit and detritus feeders. The shift from a transgressive to a highstand phase was associated with decreased siliciclastic sedimentation, leading to a natural late‐highstand baseline characterized by biostromes with epifaunal bivalves and by molluscs associated with vegetated habitats. The approach in this study reveals a major change in composition that is not seen in assemblages of the topmost sediment layers due to condensation and mixing. The most recent decline in abundance of *Gouldia minima* has no past equivalents in the Holocene highstand record of carbonate sediments off Piran, as demonstrated by down‐core trends in abundance. This decline becomes apparent in both cores from: (i) age–frequency distributions, showing a complete disjunction in production timing of *Gouldia minima* and *Corbula gibba*; and from (ii) mismatch in composition of living and late‐highstand death assemblages, demonstrating an under‐representation of most carbonate producers associated with vegetated sea floors. Such a decline in production probably reflects a decrease in light penetration due to eutrophication, and an increase in sediment disturbance and higher sediment resuspension due to elevated bottom trawling rates. Therefore, although molluscan‐dominated sands are presently located in zones of limited light penetration in the northern Adriatic Sea, it seems that more sunlight penetrated to these habitats in the early 20th century. Molluscan production generating bioclastic deposits in the southern Gulf of Trieste was thus naturally higher during the highstand phase prior to the major anthropogenic impacts that took place over the past few centuries.

## Supporting information


**Table S1.** Absolute abundances of molluscs in two cores at Piran 1 and Piran 2, including two surface living assemblages collected by Van Veen grabs at the same sites in 2014.

**Table S2.** Amino acid racemization data and calibrated estimates of post‐mortem age of *Gouldia minima* collected from the shell bed at Piran 1 and Piran 2.

**Table S3.** Amino acid racemization data and calibrated estimates of post‐mortem age of *Corbula gibba* and Piran 2.

**Table S4.** Absolute abundances of molluscs in living assemblages collected by Van Veen grabs at 11 stations in 2011.
