# Supplementary material for: A decline in molluscan carbonate production driven by the loss of vegetated habitats encoded in the Holocene sedimentary record of the Gulf of Trieste
Source: Sedimentology. 2018 Aug 25;66(3):781–807. doi: 10.1111/sed.12516 (PMC6446828; doi:10.1111/sed.12516)
Supplement: Supplementary file 1 — Table S1. Absolute abundances of molluscs in two cores at Piran 1 and Piran 2, including two surface living assemblages collected by Van Veen grabs at the same sites in 2014. [file SED-66-781-s001.pdf]

class	genus	species	determination_	Piran 1-LA	Piran 2-LA
Bivalvia	Nuculana	Nuculana pella	1	0	0
Bivalvia	Saccella	Saccella commutata	1	2	1
Bivalvia	Nucula	Nucula cf_nuclaeus	1	5	8
Bivalvia	Nucula	Nucula sulcata	1	0	0
Bivalvia	Arca	Arca noae	1	0	0
Bivalvia	Arca	Arca tetragona	1	0	0
Bivalvia	Barbatia	Barbatia barbata	1	0	0
Bivalvia	Anadara	Anadara transversa	1	0	0
Bivalvia	Anadara	Anadara inaequivalvis	1	0	0
Bivalvia	Anadara	Anadara gibbosa	1	0	0
Bivalvia	Striarca	Striarca lactea	1	0	0
Bivalvia	Glycymeris	Glycymeris glycymeris	1	0	0
Bivalvia	Glycymeris	Glycymeris nummaria	1	0	0
Bivalvia	Mytilus	Mytilus cf_edulis	1	0	0
Bivalvia	Gibbomodiola	Gibbomodiola adriatica	1	0	0
Bivalvia	Modiolus	Modiolus barbatus	1	0	1
Bivalvia	Musculus	Musculus subpictus	1	0	1
Bivalvia	NA	Mytilidae indet	0	0	0
Bivalvia	Aequipecten	Aequipecten opercularis	1	0	0
Bivalvia	Pecten	Pecten jacobaeus	1	0	0
Bivalvia	Mimachlamys	Mimachlamys varia	1	1	0
Bivalvia	Talochlamys	Talochlamys multistriata	1	0	0
Bivalvia	Flexopecten	Flexopecten glaber	1	0	0
Bivalvia	Palliolum	Palliolum incomparabile	1	0	0
Bivalvia	NA	Pectinidae indet	0	0	0
Bivalvia	Anomia	Anomia ephippium	1	0	1
Bivalvia	Heteranomia	Heteranomia squamula	1	0	0
Bivalvia	Monia	Monia patelliformes	1	0	0
Bivalvia	Lima	Lima lima juv	1	0	0
Bivalvia	Lima	Lima cf_hians	1	0	0
Bivalvia	Limaria	Limaria loscombi	1	0	0
Bivalvia	Limaria	Limaria tuberculata	1	0	0
Bivalvia	Limaria	Limaria spp	0	0	0
Bivalvia	Limatula	Limatula gwyni	1	0	0
Bivalvia	Limatula	Limatula subauriculata	1	0	0
Bivalvia	NA	Limidae indet	0	0	0
Bivalvia	Ostrea	Ostrea spp	0	0	0
Bivalvia	Ostrea	Ostrea cf_stentina	1	0	0
Bivalvia	Ostrea	Ostrea edulis	1	0	0
Bivalvia	Loripinus	Loripinus fragilis	1	2	2
Bivalvia	Ctena	Ctena decussata	1	0	0
Bivalvia	Lucinella	Lucinella divaricata	1	0	0
Bivalvia	Lucinoma	Lucinoma borealis	1	0	0
Bivalvia	Loripinus	Loripes lucinalis	1	0	0
Bivalvia	Myrtea	Myrtea spinifera	1	0	0
Bivalvia	NA	Lucinidae indet	0	0	0
Bivalvia	Thyasira	Thyasira biplicata	1	0	0
Bivalvia	Thyasira	Thyasira spp	0	0	0
Bivalvia	Coracuta	Coracuta obliquata	1	0	0
Bivalvia	Kurtiella	Kurtiella bidentata	1	0	0
Bivalvia	Tellimya	Tellimya ferruginosa	1	0	0
Bivalvia	Litigiella	Litigiella glabra	1	0	0
Bivalvia	Montacuta	Montacuta goudi	1	0	0
Bivalvia	Montacuta	Montacuta phascolionis	1	0	0
Bivalvia	Scacchia	Scacchia oblonga	1	0	0
Bivalvia	Lepton	Lepton squamosum	1	0	0
Bivalvia	Lepton	Lepton subtrigonum	1	0	0
Bivalvia	Arculus	Arculus cf_sykesii	1	0	0
Bivalvia	Hemilepton	Hemilepton nitidum	1	0	0
Bivalvia	Kellia	Kellia suborbicularis	1	0	0
Bivalvia	Bornia	Bornia geoffroyi	1	0	0
Bivalvia	Centrocardita	Centrocardita aculeata	1	0	0
Bivalvia	Chama	Chama gryphoides	1	0	0
Bivalvia	Pseudochama	Pseudochama gryphina	1	0	0
Bivalvia	Saxicavella	Saxicavella jeffreysi	1	0	0
Bivalvia	Acanthocardia	Acanthocardia paucicostata	1	2	1

Bivalvia	Acanthocardia	Acanthocardia deshayesii	1	0	0
Bivalvia	Acanthocardia	Acanthocardia aculeata	1	0	0
Bivalvia	Parvicardium	Parvicardium scriptum	1	0	0
Bivalvia	Parvicardium	Parvicardium exiguum	1	0	0
Bivalvia	Parvicardium	Parvicardium scabrum	1	0	0
Bivalvia	Parvicardium	Parvicardium minimum	1	0	1
Bivalvia	Papillicardium	Papillicardium papillosum	1	1	6
Bivalvia	NA	Fraginae indet Papillicardium_Parvicardium	0	0	0
Bivalvia	Cerastoderma	Cerastoderma glaucum	1	0	0
Bivalvia	Cerastoderma	Cerastoderma edule	1	0	0
Bivalvia	Cerastoderma	Cerastoderma spp	0	0	0
Bivalvia	Laevicardium	Laevicardium crassum	1	0	2
Bivalvia	Spisula	Spisula subtruncata	1	0	2
Bivalvia	Pisidium	Pisidium sp_1	1	0	0
Bivalvia	Abra	Abra alba	1	2	9
Bivalvia	Abra	Abra nitida	1	0	0
Bivalvia	Abra	Abra prismatica	1	0	0
Bivalvia	Abra	Abra tenuis	1	0	0
Bivalvia	Abra	Abra spp	0	0	0
Bivalvia	Moerella	Moerella cf_distorta	1	10	8
Bivalvia	Tellina	Tellina serrata	1	0	1
Bivalvia	Tellina	Tellina pulchella	1	0	0
Bivalvia	Tellina	Tellina donacina	1	0	0
Bivalvia	Tellina	Tellina fabula	1	0	0
Bivalvia	Tellina	Tellina pygmaea	1	0	0
Bivalvia	Tellina	Tellina incarnata	1	0	0
Bivalvia	NA	Tellininae indet Tellina_Moerella	0	0	0
Bivalvia	Gastrana	Gastrana fragilis	1	0	0
Bivalvia	Arcopagia	Arcopagia balaustina	1	0	0
Bivalvia	Gari	Gari costulata	1	0	0
Bivalvia	Gari	Gari fervensis	1	0	0
Bivalvia	Gari	Gari depressa	1	0	0
Bivalvia	Gari	Gari spp	0	0	0
Bivalvia	Azorinus	Azorinus chamasolen	1	0	1
Bivalvia	Solecurtus	Solecurtus candidus	1	0	0
Bivalvia	Solecurtus	Solecurtus spp	0	0	0
Bivalvia	Donax	Donax venustus	1	0	0
Bivalvia	Mysia	Mysia undata	1	0	0
Bivalvia	Gouldia	Gouldia minima	1	0	4
Bivalvia	Pitar	Pitar rudis	1	3	0
Bivalvia	Pitar	Pitar mediterraneus	1	0	0
Bivalvia	Callista	Callista chione	1	0	0
Bivalvia	Polititapes	Polititapes cf_rhomboides	1	0	0
Bivalvia	Polititapes	Polititapes aurea	1	0	7
Bivalvia	Polititapes	Polititapes spp	0	0	0
Bivalvia	NA	Tapetinae indet	0	0	0
Bivalvia	Clausinella	Clausinella fasciata	1	0	0
Bivalvia	Dosinia	Dosinia lupinis	1	0	0
Bivalvia	Venus	Venus verrucosa	1	0	0
Bivalvia	Venus	Venus casina	1	0	0
Bivalvia	Timoclea	Timoclea ovata	1	13	70
Bivalvia	NA	Veneridae indet	0	0	0
Bivalvia	Coralliophaga	Coralliophaga sp_1	1	0	0
Bivalvia	Diplodonta	Diplodonta brocchii	1	0	0
Bivalvia	Diplodonta	Diplodonta trigona	1	0	0
Bivalvia	Corbula	Corbula gibba	1	4	11
Bivalvia	Lentidium	Lentidium mediterraneum	1	0	0
Bivalvia	Barnea	Barnea candida	1	0	0
Bivalvia	Phaxas	Phaxas adriaticus	1	5	19
Bivalvia	Ensis	Ensis ensis	1	0	0
Bivalvia	Rocellaria	Rocellaria dubia	1	1	0
Bivalvia	Hiatella	Hiatella arctica	1	1	5
Bivalvia	Pandora	Pandora pinna	1	0	0
Bivalvia	Pandora	Pandora inaequivalvis	1	0	0
Bivalvia	Cuspidaria	Cuspidaria cuspidata	1	0	0
Bivalvia	Cardiomya	Cardiomya costellata	1	0	0
Bivalvia	Thracia	Thracia phaseolina	1	0	0

Bivalvia	Thracia	Thracia corbuloides	1	0	0
Bivalvia	Thracia	Thracia cf_distorta	1	0	0
Bivalvia	Cochlodesma	Cochlodesma praetenue	1	0	0
Bivalvia	NA	Bivalvia indet	0	0	0
Gastropoda	Tectura	Tectura virginea	1	0	0
Gastropoda	Diodora	Diodora graeca	1	0	0
Gastropoda	Diodora	Diodora italica	1	0	0
Gastropoda	Diodora	Diodora gibberula	1	0	0
Gastropoda	Emarginula	Emarginula rosea	1	0	0
Gastropoda	Emarginula	Emarginula huzardii	1	0	0
Gastropoda	Scissurella	Scissurella costata	1	0	0
Gastropoda	Tricolia	Tricolia pullus	1	0	0
Gastropoda	Haliotis	Haliotis tuberculata	1	0	0
Gastropoda	Calliostoma	Calliostoma cf_conulus	1	0	0
Gastropoda	Calliostoma	Calliostoma cf_zizyphinum	1	0	0
Gastropoda	Calliostoma	Calliostoma laugieri ev auch conulus	1	0	0
Gastropoda	Gibbula	Gibbula fanulum	1	0	0
Gastropoda	Gibbula	Gibbula cf ardens	1	0	0
Gastropoda	Gibbula	Gibbula cf_guttadauri	1	0	0
Gastropoda	Gibbula	Gibbula cf_varia	1	0	0
Gastropoda	Gibbula	Gibbula cf_leucophaea	1	0	0
Gastropoda	Jujubinus	Jujubinus montagui	1	0	0
Gastropoda	Jujubinus	Jujubinus exasperatus	1	0	0
Gastropoda	Jujubinus	Jujubinus cf_striatus	1	0	0
Gastropoda	Jujubinus	Jujubinus spp	0	0	0
Gastropoda	Clelandella	Clelandella miliaris	1	0	0
Gastropoda	Clanculus	Clanculus cruciatus	1	0	0
Gastropoda	Phorcus	Phorcus richardi	1	0	0
Gastropoda	Bolma	Bolma rugosa	1	0	0
Gastropoda	Bittium	Bittium latreilli	1	0	0
Gastropoda	Bittium	Bittium reticulatum cf_scabrum	1	0	0
Gastropoda	Bittium	Bittium submamillatum	1	0	0
Gastropoda	Cerithium	Cerithium cf_vulgatum	1	0	0
Gastropoda	Cerithium	Cerithium sp_1 ev_auch vulgatum	1	0	0
Gastropoda	Turritella	Turritella communis	1	6	0
Gastropoda	Turritella	Turritella turbona	1	0	0
Gastropoda	Cerithiopsis	Cerithiopsis tubercularis	1	0	0
Gastropoda	Cerithiopsis	Cerithiopsis jeffreysi	1	0	0
Gastropoda	Cerithiopsis	Cerithiopsis barleei	1	0	0
Gastropoda	Cerithiopsis	Cerithiopsis nana	1	0	0
Gastropoda	Cerithiopsis	Cerithiopsis nofronii	1	0	0
Gastropoda	Cerithiopsis	Cerithiopsis cf_diadema	1	0	0
Gastropoda	Cerithiopsis	Cerithiopsis spp	0	0	0
Gastropoda	Dizoniopsis	Dizoniopsis cf_abylensis	1	0	0
Gastropoda	Monophorus	Monophorus thiriotae	1	0	0
Gastropoda	Monophorus	Monophorus perversus	1	0	0
Gastropoda	Marshallora	Marshallora adversa	1	0	0
Gastropoda	Metaxia	Metaxia metaxa	1	0	0
Gastropoda	Similiphora	Similiphora cf_similior	1	0	0
Gastropoda	NA	Triphoridae indet	0	0	0
Gastropoda	Epitonium	Epitonium muricatum	1	0	0
Gastropoda	Epitonium	Epitonium clathrus	1	0	0
Gastropoda	Epitonium	Epitonium cf_clathratulum	1	0	0
Gastropoda	Epitonium	Epitonium algerianum	1	0	0
Gastropoda	Epitonium	Epitonium turtonis	1	0	0
Gastropoda	Eulima	Eulima glabra	1	0	0
Gastropoda	Eulima	Eulima bilineata	1	0	0
Gastropoda	Melanella	Melanella alba	1	0	0
Gastropoda	Melanella	Melanella frielei	1	0	0
Gastropoda	Melanella	Melanella cf_polita	1	0	0
Gastropoda	Melanella	Melanella spp	0	0	0
Gastropoda	Vitreolina	Vitreolina curva	1	0	0
Gastropoda	Curveulima	Curveulima devians	1	0	0
Gastropoda	NA	Eulimidae indet	0	0	0
Gastropoda	Aclis	Aclis minor	1	0	0
Gastropoda	Alvania	Alvania beanii	1	0	0
Gastropoda	Alvania	Alvania cancellata	1	0	0

Gastropoda	Alvania	Alvania carinata	1	0	0
Gastropoda	Alvania	Alvania cimex	1	0	0
Gastropoda	Alvania	Alvania geryonia	1	0	0
Gastropoda	Alvania	Alvania hispidula	1	0	0
Gastropoda	Alvania	Alvania cf_lineata	1	0	0
Gastropoda	Alvania	Alvania punctura	1	0	0
Gastropoda	Pusillina	Pusillina inconspicua	1	0	0
Gastropoda	Pusillina	Pusillina lineolata	1	0	0
Gastropoda	Pusillina	Pusillina philippi	1	0	0
Gastropoda	Pusillina	Pusillina radiata	1	0	0
Gastropoda	Pusillina	Pusillina sp_1 cf_marginata	1	0	0
Gastropoda	Pusillina	Pusillina cf_sarsii	1	0	0
Gastropoda	Setia	Setia sp_1 cf_silikorum	1	0	0
Gastropoda	Crisilla	Crisilla semistriata	1	0	0
Gastropoda	Rissoina	Rissoina bruguieri	1	0	0
Gastropoda	Rissoa	Rissoa decorata	1	0	0
Gastropoda	Rissoa	Rissoa cf_guerinii	1	0	0
Gastropoda	Rissoa	Rissoa monodonta	1	0	0
Gastropoda	Rissoa	Rissoa ventricosa	1	0	0
Gastropoda	Rissoa	Rissoa splendida	1	0	0
Gastropoda	Rissoa	Rissoa membranacea	1	0	0
Gastropoda	Rissoa	Rissoa violacea	1	0	0
Gastropoda	Rissoa	Rissoa spp	0	0	0
Gastropoda	Manzonia	Manzonia crassa	1	0	0
Gastropoda	Aporrhais	Aporrhais pespelecani	1	2	0
Gastropoda	Megalomphalu	Megalomphalus azoneus	1	0	0
Gastropoda	Calyptraea	Calyptraea chinensis	1	1	1
Gastropoda	Crepidula	Crepidula moulinsii	1	0	0
Gastropoda	Hydrobia	Hydrobia sp_1 cf_acuta	1	0	0
Gastropoda	Ecrobia	Ecrobia sp_1 cf_ventrosa	1	0	0
Gastropoda	NA	Hydrobiidae indet	0	0	0
Gastropoda	Hyala	Hyala vitrea	1	0	0
Gastropoda	Ceratia	Ceratia proxima	1	0	0
Gastropoda	Circulus	Circulus striatus	1	0	0
Gastropoda	Caecum	Caecum trachea	1	0	0
Gastropoda	Lamellaria	Lamellaria perspicua	1	0	0
Gastropoda	Euspira	Euspira nitida	1	0	2
Gastropoda	Euspira	Euspira macilenta	1	0	0
Gastropoda	Natica	Natica stercusmuscarum	1	0	0
Gastropoda	NA	Naticidae juv_indet	0	0	0
Gastropoda	Typhinellus	Typhinellus labiatus juv	1	0	0
Gastropoda	Hexaplex	Hexaplex trunculus juv	1	0	0
Gastropoda	Bolinus	Bolinus brandaris	1	0	0
Gastropoda	NA	Muricidae indet	0	0	0
Gastropoda	Trophonopsis	Trophonopsis sp_1	1	0	0
Gastropoda	Ocenebra	Ocenebra erinaceus	1	0	0
Gastropoda	Ocinebrina	Ocinebrina helleri	1	0	0
Gastropoda	Gibberula	Gibberula philippii	1	0	0
Gastropoda	Gibberula	Gibberula turgidula	1	0	0
Gastropoda	Gibberula	Gibberula spp	0	0	0
Gastropoda	Granulina	Granulina marginata	1	0	0
Gastropoda		Vexillum ebenus	1	0	0
Gastropoda	Fusinus	Fusinus rostratus	1	1	0
Gastropoda	Nassarius	Nassarius pygmaeus	1	0	0
Gastropoda	Nassarius	Nassarius lima	1	0	0
Gastropoda	Nassarius	Nassarius nitidus	1	0	0
Gastropoda	Nassarius	Nassarius reticulatus	1	0	0
Gastropoda	Mitrella	Mitrella minor	1	0	0
Gastropoda	Comarmondia	Comarmondia gracilis	1	0	0
Gastropoda	Bela	Bela brachystoma	1	0	0
Gastropoda	Bela	Bela cf_menkhorsti	1	0	0
Gastropoda	Bela	Bela nebula	1	0	1
Gastropoda	Mangelia	Mangelia unifasciata	1	0	0
Gastropoda	Mangelia	Mangelia cf_costata	1	0	0
Gastropoda	Mangelia	Mangelia costulata	1	0	0
Gastropoda	Mangelia	Mangelia attenuata	1	0	0
Gastropoda	Mangelia	Mangelia stosiciana	1	0	0

Gastropoda	Mangelia	Mangelia sp_1	1	0	0
Gastropoda	Raphitoma	Raphitoma cf_leufroyi	1	0	0
Gastropoda	Raphitoma	Raphitoma cf_linearis	1	0	0
Gastropoda	Raphitoma	Raphitoma cf_echinata	1	0	0
Gastropoda	Raphitoma	Raphitoma cf_atropurpurea	1	0	0
Gastropoda	Raphitoma	Raphitoma pseudohystrix	1	0	0
Gastropoda	Raphitoma	Raphitoma cf_aequalis	1	0	0
Gastropoda	Raphitoma	Raphitoma cf_horrida	1	0	0
Gastropoda	Raphitoma	Raphitoma cf_hispida	1	0	0
Gastropoda	Raphitoma	Raphitoma cf_densa	1	0	0
Gastropoda	Raphitoma	Raphitoma cf_ pumila	1	0	0
Gastropoda	Raphitoma	Raphitoma spp	0	0	0
Gastropoda	Mitromorpha	Mitromorpha columbellaria	1	0	0
Gastropoda	Capulus	Capulus ungaricus	1	0	0
Gastropoda	Odostomia	Odostomia cf_eulimoides	1	0	0
Gastropoda	Odostomia	Odostomia acuta	1	0	0
Gastropoda	Odostomia	Odostomia cf_turriculata	1	0	0
Gastropoda	Odostomia	Odostomia spp	0	0	0
Gastropoda	Megastomia	Megastomia conoidea	1	0	0
Gastropoda	Megastomia	Megastomia conspicua	1	0	0
Gastropoda	Ondina	Ondina vitrea	1	0	0
Gastropoda	Ondina	Ondina cf_diaphana oder Odostomia	1	0	0
Gastropoda	Turbonilla	Turbolilla acutissima	1	0	0
Gastropoda	Turbonilla	Turbonilla rufa	1	0	0
Gastropoda	Turbonilla	Turbonilla sp_1 cf_lactea	1	0	0
Gastropoda	Turbonilla	Turbonilla jeffreysii	1	0	0
Gastropoda	Turbonilla	Turbonilla gradata	1	0	0
Gastropoda	Turbonilla	Turbonilla pusilla	1	0	0
Gastropoda	Turbonilla	Turbonilla spp	0	0	0
Gastropoda	Eulimella	Eulimella acicula	1	0	0
Gastropoda	Chrysallida	Chrysallida sp_1	1	0	0
Gastropoda	Chrysallida	Chrysallida sp_2	1	0	0
Gastropoda	Chrysallida	Chrysallida sp_3 ev_interstincta	1	0	0
Gastropoda	Chrysallida	Chrysallida sp_4 Parthenina suturalis	1	0	0
Gastropoda	Chrysallida	Chrysallida cf_clathrata	1	0	0
Gastropoda	Chrysallida	Chrysallida cf_indistincta	1	0	0
Gastropoda	Chrysallida	Chrysallida cf_interstincta	1	0	0
Gastropoda	Chrysallida	Chrysallida terebellum	1	0	0
Gastropoda	Ebala	Ebala nitidissima	1	0	0
Gastropoda	Folinella	Folinella excavata	1	0	0
Gastropoda	Euparthenia	Euparthenia bulinea	1	2	0
Gastropoda	Clathrella	Clathrella clathrata	1	0	0
Gastropoda	Acteon	Acteon tornatilis	1	0	0
Gastropoda	Ringicula	Ringicula conformis	1	0	0
Gastropoda	Akera	Akera bullata	1	0	0
Gastropoda	Weinkauffia	Weinkauffia turgidula	1	0	0
Gastropoda	Haminoea	Haminoea hydatis	1	0	0
Gastropoda	Atys	Atys jeffreysi	1	0	0
Gastropoda	Philine	Philine catena	1	0	0
Gastropoda	Philine	Philine scabra	1	0	0
Gastropoda	Philine	Philine quadripartita	1	0	0
Gastropoda	Philine	Philine pruinosa	1	0	0
Gastropoda	Philine	Philine retifera	1	0	0
Gastropoda	Retusa	Retusa truncatula	1	0	0
Gastropoda	Retusa	Retusa umbilicata	1	0	0
Gastropoda	Retusa	Retusa laevisculpta	1	0	0
Gastropoda	Retusa	Retusa minutissima	1	0	0
Gastropoda	Retusa	Retusa sp_1 cf_nitidula	1	0	0
Gastropoda	Cylichna	Cylichna cylindracea	1	0	0
Gastropoda	Volvulella	Volvulella acuminata	1	0	0
Gastropoda	Creseis	Creseis acicula	1	0	0
Gastropoda	Tylodina	Tylodina sp_1	1	0	0
Gastropoda	Smaragdia	Smaragdia sp	1	0	0
Gastropoda	NA	Gastropoda marine indet	0	0	0
Gastropoda	Oxyloma	Oxyloma elegans	1	0	0
Gastropoda	Succinella	Succinella oblonga	1	0	0
Gastropoda	Vallonia	Vallonia cf_pulchella	1	0	0

Gastropoda	Vallonia	Vallonia cf_costata	1	0	0
Gastropoda	Oxychilus	Oxychilus spp	1	0	0
Gastropoda	NA	Hygromiidae Helicella_Trochulus hispidus	1	0	0
Gastropoda	Pupilla	Pupilla triplicata	1	0	0
Gastropoda	Pupilla	Pupilla cf_muscorum	1	0	0
Gastropoda	Vertigo	Vertigo antivertigo	1	0	0
Gastropoda	Vertigo	Vertigo angustior	1	0	0
Gastropoda	Stagnicola	Stagnicola spp	1	0	0
Gastropoda	Radix	Sp_4 ev_Radix	1	0	0
Gastropoda	Segmentina	Segmentina nitida	1	0	0
Gastropoda	Anisus	Anisus cf_leucostoma	1	0	0
Gastropoda	Gyraulus	Gyraulus cf_laevis	1	0	0
Gastropoda	Gyraulus	Gyraulus cf_albus	1	0	0
Gastropoda	Gyraulus	Gyraulus crista	1	0	0
Gastropoda	Gyraulus	Gyraulus sp_1	1	0	0
Gastropoda	NA	Sp_3 big Planorbidae	0	0	0
Gastropoda	Ancylus	Ancylus lacustris	1	0	0
Gastropoda	Valvata	Valvata macrostoma	1	0	0
Gastropoda	Valvata	Valvata piscinalis	1	0	0
Gastropoda	Valvata	Valvata cristata	1	0	0
Gastropoda	Bithynia	Bithynia spp	1	0	0
Gastropoda	Bithynia	Bithynia tentaculata	1	0	0
Gastropoda	Viviparus	Sp_1 Viviparus sp_1	1	0	0
Gastropoda	NA	Sp_2 similar Viviparus but angulated	0	0	0
Gastropoda	NA	Gastropoda nonmarine indet	0	0	0
Scaphopoda	Antalis	Antalis inaequicostata	1	0	0
Scaphopoda	Antalis	Antalis vulgaris	1	0	0
Scaphopoda	Fustiaria	Fustiaria rubescens	1	0	0
Scaphopoda	Episiphon	Episiphon filum	1	0	0
Scaphopoda	Dischides	Dischides politus	1	0	0
Polyplacophora	Chiton	Chiton olivaceus	1	0	0
Polyplacophora	Chiton	Chiton cf_corallinus	1	0	0
Polyplacophora	Callochiton	Callochiton sp_1 cf_septemvalvis	1	0	0
Polyplacophora	Leptochiton	Leptochiton cf_ algesirensis	1	0	0
Polyplacophora	Leptochiton	Leptochiton sp_ 1 cf_cancellatus	1	0	0
Polyplacophora	Leptochiton	Leptochiton cf_bedullii	1	0	0
Polyplacophora	Parachiton	Parachiton cf_africanus	1	0	0
Polyplacophora	Lepidopleurus	Leptochiton sp_3	1	0	0
Polyplacophora	Acanthochitona	Acanthochitona fascicularis	1	0	1

Pir1_M1_0_2	Pir1_M1_2_4	Pir1_M1_4_6	Pir1_M1_6_8	Pir1_M1_8_10	Pir1_M1_10_1	Pir1_M1_12_1	Pir1_M1_14_1	Pir1_M1_16_1
0	1	0	3	1	1	5	1	4
1	1	0	0	0	0	0	0	0
4	11	13	16	14	21	19	13	21
0	0	0	0	0	0	0	0	0
2	3	4	2	3	6	7	5	7
0	0	0	0	0	0	0	1	0
0	0	0	0	0	0	0	0	0
2	2	0	0	0	0	0	1	0
0	0	0	0	0	0	0	0	0
0	0	0	0	0	0	0	0	0
1	2	1	0	3	8	3	2	4
0	0	0	0	0	0	0	0	0
0	0	0	0	0	1	0	0	1
0	1	0	0	0	0	0	0	0
0	0	0	0	0	0	0	0	0
1	4	5	2	2	4	9	7	9
0	1	3	1	1	3	2	4	3
0	0	0	0	0	0	0	0	0
0	0	2	0	1	0	4	2	0
0	0	0	0	0	0	0	0	0
2	1	2	1	1	3	9	2	7
1	0	0	0	0	1	0	0	0
0	0	0	0	2	4	7	12	10
0	0	0	0	0	0	0	0	0
0	0	0	0	0	0	1	0	0
4	11	5	11	2	8	7	6	3
0	1	2	0	0	1	1	1	4
3	1	2	3	2	3	3	3	9
0	0	0	0	0	0	0	0	0
0	0	0	0	0	0	0	0	0
0	0	0	0	0	0	0	0	0
0	0	0	0	0	0	0	0	0
0	1	0	1	1	0	1	0	2
0	0	0	0	0	0	0	0	0
0	0	0	0	0	0	0	0	0
0	0	0	0	0	0	0	0	0
1	2	4	5	4	7	17	12	19
0	0	0	0	0	0	0	0	0
0	0	0	0	0	0	0	0	0
1	3	0	0	1	4	1	1	4
0	0	0	0	0	0	0	0	0
0	0	1	0	0	0	0	1	0
0	0	0	0	0	0	0	0	0
0	0	0	0	0	0	0	0	0
0	0	0	0	0	3	1	2	0
0	0	0	0	0	0	0	0	0
0	0	1	1	1	1	2	0	1
0	0	0	0	0	0	0	0	0
0	0	0	0	0	0	0	0	0
1	5	4	7	6	5	5	1	1
0	0	0	0	0	0	0	0	0
0	1	0	1	1	0	0	1	0
0	0	0	0	0	0	0	0	0
0	0	0	0	0	0	0	0	0
0	0	0	0	0	0	0	0	0
1	0	0	0	0	0	0	1	0
0	0	0	0	0	0	0	0	0
0	0	0	0	0	0	0	0	0
0	1	5	4	4	8	3	2	3
0	0	0	0	0	0	1	0	0
0	0	0	0	0	0	0	0	0
3	0	2	1	3	2	2	2	2
1	0	2	2	1	2	8	5	4
0	0	0	0	0	0	1	1	0
0	0	0	0	0	0	0	0	0
0	0	0	0	0	0	0	0	0

0	0	0	0	0	0	0	0	0
0	2	2	1	3	2	1	2	5
0	0	0	0	2	1	0	0	0
1	2	3	5	3	2	7	8	8
21	28	53	38	41	60	70	46	57
1	0	0	1	1	0	0	2	0
8	20	24	16	26	52	58	52	53
3	0	0	2	4	5	10	0	0
0	0	0	0	0	0	0	0	0
0	0	0	0	0	0	0	0	0
0	0	0	0	0	0	0	0	0
1	0	1	1	1	0	1	0	0
1	2	0	1	0	0	0	0	0
0	0	0	0	0	0	0	0	0
2	3	2	4	2	6	6	7	13
0	0	0	0	0	0	0	1	0
0	0	0	0	0	0	0	0	0
0	0	0	0	0	0	0	0	0
0	0	0	0	0	0	0	0	0
1	2	4	2	3	4	14	10	15
0	0	0	0	0	0	0	0	0
0	0	0	0	0	0	0	0	0
0	0	0	0	0	0	0	0	0
0	0	0	0	0	0	0	0	0
0	0	0	0	0	0	0	0	0
0	0	0	0	0	0	0	0	0
0	1	0	0	0	0	0	0	0
0	0	0	0	0	0	0	0	1
0	0	0	0	1	0	2	2	1
0	0	0	0	0	0	0	0	0
0	0	0	0	0	0	0	0	0
0	0	0	0	0	0	0	0	0
0	0	0	0	0	0	0	0	0
0	0	0	0	1	1	0	0	4
0	0	0	0	0	0	0	0	0
0	0	0	0	0	0	0	0	0
0	0	0	0	0	0	0	0	0
0	0	2	1	0	0	0	0	0
44	116	126	140	156	325	336	240	294
3	6	4	9	13	18	23	21	21
0	0	0	0	0	0	0	0	0
0	0	0	0	0	0	0	0	0
1	0	1	1	1	0	0	0	0
0	0	0	0	0	0	0	0	0
0	0	0	0	0	0	0	0	0
0	0	0	0	0	0	0	0	0
0	0	0	0	0	0	1	0	0
0	0	0	0	0	0	0	0	0
0	3	1	2	2	2	3	0	3
0	0	0	0	0	1	0	1	1
1	2	0	1	1	3	2	1	2
0	0	0	0	0	0	0	0	0
0	0	0	0	0	0	0	0	0
1	0	1	0	0	0	0	0	0
0	0	0	0	0	0	0	0	0
29	70	63	52	65	87	107	64	89
0	0	0	0	0	0	0	0	0
0	0	0	0	0	0	0	0	0
0	2	1	0	0	0	1	0	0
0	0	0	0	0	0	0	0	0
1	1	2	1	7	2	4	3	2
3	2	6	8	5	9	8	7	5
0	0	0	0	0	0	0	0	0
0	0	0	0	0	0	0	0	0
0	0	0	0	0	0	0	0	0
0	0	0	0	0	0	0	0	0
1	0	1	0	0	1	0	0	0

0	0	0	0	0	0	0	0	0
0	0	0	0	0	0	0	0	0
0	0	0	0	0	0	0	0	0
1	0	1	0	0	0	0	0	0
0	0	0	0	0	0	0	1	0
0	2	7	0	3	2	6	3	1
0	0	0	0	0	0	0	0	0
0	0	0	0	0	0	0	0	0
0	0	0	0	0	0	0	0	0
0	0	0	0	0	0	0	0	0
0	0	0	0	0	0	1	1	2
0	0	0	0	0	0	0	0	0
0	0	0	0	0	0	1	0	0
0	0	0	0	0	0	0	0	0
0	0	0	1	1	0	1	1	2
0	0	0	0	0	1	0	0	0
0	0	0	0	0	0	0	0	0
0	0	0	0	0	0	0	0	0
1	0	0	0	1	0	0	0	1
0	0	0	0	0	0	0	0	0
0	0	0	0	0	0	0	0	0
0	0	1	3	1	1	2	0	2
1	1	0	2	2	6	1	1	6
0	0	0	0	0	0	0	0	0
0	0	0	0	0	0	0	0	0
0	0	0	0	0	0	0	0	0
0	0	0	0	0	0	0	0	0
0	0	0	0	0	0	0	0	0
1	0	0	0	0	1	0	0	0
25	87	81	82	60	209	143	137	159
2	3	7	5	7	13	20	12	12
4	27	31	38	52	127	124	75	69
1	3	5	0	2	11	13	10	15
0	0	0	0	0	0	0	0	0
2	9	11	9	8	29	22	11	19
0	0	0	0	2	3	4	2	2
0	0	0	1	0	1	1	0	0
0	0	0	0	0	0	0	0	0
0	0	0	0	0	0	0	0	0
0	0	0	0	0	0	0	0	0
0	0	0	0	0	0	0	0	0
0	0	0	0	0	0	0	0	0
0	0	0	0	0	1	0	0	0
0	0	0	0	0	0	0	0	0
0	0	0	0	0	0	0	0	0
0	0	0	0	0	0	0	0	1
0	6	0	1	0	2	6	3	7
0	0	0	0	0	0	0	0	0
0	0	0	0	0	0	0	0	0
2	2	9	3	5	8	13	8	8
0	1	0	1	0	3	0	2	2
0	1	1	0	1	0	3	0	2
0	0	0	0	0	0	0	0	0
0	0	0	0	0	0	0	0	0
0	0	0	0	0	0	0	0	0
1	0	1	2	1	1	0	1	0
0	0	0	0	0	0	0	0	0
0	0	0	0	0	0	0	0	0
0	0	0	0	0	0	0	0	0
0	0	0	0	0	1	0	0	0
0	0	0	0	0	0	0	0	0
0	0	0	0	0	0	0	0	0
0	0	0	0	0	0	0	0	0
0	0	0	0	0	0	0	0	0
1	0	0	0	0	0	1	0	0
0	0	0	0	0	0	0	0	0
0	0	0	0	0	0	0	1	0

0	0	0	0	0	0	1	0	0
1	1	4	1	3	9	5	5	7
2	3	1	1	5	19	13	17	13
0	0	0	0	0	0	0	1	0
0	0	0	0	1	0	0	0	0
0	0	0	0	0	0	0	0	0
1	0	4	1	0	7	9	9	6
2	15	12	18	32	25	43	27	45
0	0	1	3	2	0	4	2	5
2	7	11	7	13	46	74	34	47
0	1	0	0	1	0	0	0	0
0	0	0	0	0	1	0	0	0
0	0	0	0	0	0	0	0	0
0	0	0	0	0	0	0	0	0
0	0	0	0	0	0	0	0	0
0	0	0	0	0	0	0	0	0
0	0	0	0	0	0	1	0	0
0	0	0	0	0	0	0	0	0
0	0	0	0	0	0	0	0	0
0	0	0	0	0	0	0	0	0
0	0	0	0	0	0	0	0	0
0	0	2	0	0	0	0	0	1
0	0	0	0	0	0	0	0	0
0	2	0	0	1	1	4	3	1
2	2	3	4	4	2	4	6	4
0	0	0	0	0	0	0	0	0
0	1	6	2	0	1	2	3	3
0	0	0	0	0	0	0	0	1
0	0	0	0	0	0	1	0	0
0	0	0	0	0	0	0	0	0
0	0	0	0	0	0	0	0	0
0	0	0	0	0	0	0	0	0
0	0	0	0	0	0	0	0	0
0	0	0	0	0	0	1	0	0
0	0	0	0	0	1	0	1	1
0	0	0	0	0	0	0	0	0
2	3	9	9	14	19	25	11	25
0	0	3	2	0	3	2	2	4
0	0	0	0	0	0	0	0	0
0	0	0	0	0	0	0	0	0
0	0	0	0	0	0	0	0	0
0	2	0	2	1	3	6	1	3
1	0	0	0	0	0	1	0	2
0	0	0	0	0	0	0	0	0
0	0	0	0	0	0	0	0	0
0	0	0	0	0	0	0	2	0
0	0	0	0	0	0	0	0	0
0	0	0	0	0	0	0	0	0
0	0	0	0	0	0	0	0	0
0	0	0	0	1	2	0	1	3
0	0	0	0	1	0	4	3	0
0	0	0	0	0	0	0	0	0
0	0	0	2	3	1	0	1	0
5	9	11	22	15	12	33	35	43
0	0	0	0	0	0	0	0	0
0	0	0	0	0	0	0	0	0
0	0	0	0	0	0	0	0	0
0	0	0	0	0	0	0	0	0
0	0	0	0	0	1	1	0	1
0	4	0	3	1	7	9	0	8
0	0	0	0	0	0	1	0	0
0	1	0	1	0	0	0	0	0
2	3	0	0	4	5	8	10	0
0	0	0	0	0	0	0	0	0
0	5	0	4	1	0	11	3	0
1	0	2	0	0	0	3	0	0
0	0	0	0	1	3	0	0	0

0	0	0	0	0	0	0	0	0
0	1	0	0	0	0	1	1	0
0	0	1	0	0	0	0	0	0
0	0	0	0	1	2	3	0	2
0	1	0	1	0	2	0	1	1
0	0	0	0	0	0	0	0	0
0	0	0	0	0	0	0	0	0
0	0	0	0	1	1	0	0	0
0	1	1	0	0	1	1	1	0
0	0	0	0	0	0	0	0	0
1	0	0	0	0	2	1	0	2
0	0	0	0	0	0	0	0	0
0	0	0	0	0	0	0	0	0
0	0	0	0	0	0	1	0	0
1	0	1	0	2	1	1	1	4
0	0	0	0	0	0	0	0	0
0	0	0	0	0	0	0	0	0
0	0	0	0	0	0	1	1	0
1	3	2	6	1	6	10	7	11
0	0	1	0	0	0	1	0	2
0	2	0	0	0	0	1	1	1
0	0	0	0	0	0	0	0	0
1	3	3	0	0	4	5	6	3
0	0	0	0	0	1	0	1	0
0	0	0	0	0	0	0	0	0
0	0	0	0	0	0	0	0	0
0	0	0	0	0	2	0	0	0
0	0	0	0	0	1	0	0	0
0	0	0	0	0	0	0	0	0
0	0	0	0	0	0	0	0	0
0	0	0	0	0	0	0	0	0
0	0	0	0	0	0	0	0	0
0	0	0	0	0	0	0	0	0
0	0	0	0	1	0	2	0	0
0	0	0	0	0	0	0	0	0
0	0	0	0	0	0	0	0	0
0	0	0	0	1	3	1	1	4
0	0	0	0	0	0	0	0	0
0	0	0	0	0	0	0	0	0
0	0	0	0	0	0	0	1	0
0	0	0	0	0	0	0	0	0
0	0	0	0	0	0	0	0	0
0	0	0	1	1	1	0	2	2
0	0	0	0	0	0	0	0	0
0	0	0	0	0	0	0	0	0
0	0	0	0	0	1	0	0	1
0	0	0	0	0	1	1	0	2
0	0	1	0	0	0	0	1	0
0	0	0	0	0	0	0	0	1
0	0	0	0	0	0	0	0	0
0	0	0	0	0	0	0	0	0
0	0	0	0	0	0	0	0	0
0	0	0	0	0	0	0	0	0
0	0	0	0	0	0	1	0	0
0	0	0	0	0	0	1	0	0
0	0	1	0	0	0	2	0	0
0	1	0	0	1	0	0	0	1
0	0	0	0	0	0	0	0	0
0	0	0	0	0	0	0	1	0
0	0	0	0	0	0	0	0	0
0	0	0	0	0	0	0	0	0
0	0	0	0	0	0	0	0	0
0	0	0	0	0	0	0	0	0
0	0	2	0	0	0	0	0	0
0	0	0	0	0	0	0	0	0
0	0	0	0	0	0	0	0	0
0	0	0	0	0	0	0	0	0

0	0	0	0	0	0	0	0	0
0	0	0	0	0	0	0	0	0
0	0	0	0	0	0	0	0	0
0	0	0	0	0	0	0	0	0
0	0	0	0	0	0	0	0	0
0	0	0	0	0	0	0	0	0
0	0	0	0	0	0	0	0	0
0	0	0	0	0	0	0	0	0
0	0	0	0	0	0	0	0	0
0	0	0	0	0	0	0	0	0
0	0	0	0	0	0	0	0	0
0	0	0	0	0	0	0	0	0
0	0	0	0	0	0	0	0	0
0	0	0	0	0	0	0	0	0
0	0	0	0	0	0	0	0	0
0	0	0	0	0	0	0	0	0
0	0	0	0	0	0	0	0	0
0	0	0	0	0	0	0	0	0
0	0	0	0	0	0	0	0	0
0	0	0	0	0	0	0	0	0
0	0	0	0	0	0	0	0	0
0	0	0	0	0	0	0	0	0
0	0	0	0	0	0	0	0	0
0	0	0	0	0	0	0	0	0
0	0	0	0	0	0	0	0	0
2	3	2	4	3	16	27	12	19
0	0	0	0	0	0	0	0	0
0	0	1	0	0	0	0	0	0
0	0	0	0	0	0	0	0	0
0	0	0	0	0	0	0	0	0
3	3	5	5	3	5	7	6	7
0	0	0	0	0	0	0	0	0
0	0	1	0	0	2	0	0	1
0	0	0	0	0	0	0	0	0
0	0	0	1	0	0	0	0	0
0	0	1	0	0	0	0	0	1
0	0	0	0	0	0	0	0	0
0	0	0	0	0	0	0	0	0
2	3	2	1	2	3	4	5	2

Pir1_M1_18_2(	Pir1_M1_20_2!	Pir1_M1_25_3(	Pir1_M1_30_3!	Pir1_M1_35_4(	Pir1_M1_40_4!	Pir1_M1_45_5(	Pir1_M1_50_5!	Pir1_M1_55_6(
3	4	2	6	1	2	2	1	0
0	0	0	0	0	0	0	0	0
12	43	32	21	17	11	12	12	8
0	0	0	0	0	0	0	0	0
4	11	7	7	6	5	1	3	2
0	0	0	0	0	0	0	0	0
0	0	0	0	0	0	0	0	0
0	0	0	0	0	0	0	0	0
0	0	0	0	0	0	0	0	0
0	0	0	0	0	0	0	0	0
6	10	8	4	3	5	3	2	0
0	0	0	0	0	0	0	0	0
0	0	0	0	0	0	0	0	0
0	0	1	0	0	0	0	0	0
0	0	0	0	0	0	0	0	0
9	16	12	17	15	6	9	7	6
5	8	8	11	8	8	5	9	3
0	0	0	0	0	0	0	0	0
2	1	3	5	1	1	0	1	0
0	0	0	0	0	0	0	0	0
4	12	10	10	7	7	7	6	4
0	0	0	0	0	0	0	0	0
6	17	14	13	10	14	13	15	5
0	0	0	0	1	0	0	0	0
0	1	0	0	1	0	1	3	3
5	7	18	22	10	11	9	5	6
0	7	7	8	7	0	6	0	2
13	13	12	20	6	6	4	4	2
0	0	0	0	0	0	0	0	0
0	0	0	0	0	0	0	0	0
0	0	0	0	0	0	0	0	0
0	1	0	0	0	1	0	0	0
1	2	2	0	1	0	0	1	1
0	0	0	0	0	0	0	0	0
0	0	0	0	0	0	0	0	0
0	0	0	0	0	0	0	0	0
32	70	81	74	53	28	35	38	63
0	0	0	0	0	0	0	0	0
0	0	0	0	0	0	0	0	0
4	7	25	19	30	15	16	19	13
0	0	0	0	0	0	0	0	0
0	0	1	0	0	0	0	0	0
0	0	0	0	0	0	0	0	0
0	0	0	0	0	0	0	0	0
1	2	2	1	0	0	0	0	0
0	0	0	0	0	0	0	0	0
1	3	4	5	5	4	1	2	1
0	0	0	0	0	0	0	0	0
0	0	0	0	0	1	0	0	0
1	10	10	11	11	4	10	7	1
0	0	0	1	0	0	0	1	0
0	0	1	1	1	0	1	0	0
0	0	0	0	0	0	0	0	0
0	0	0	0	0	0	0	0	0
0	1	1	0	0	1	4	0	0
1	2	3	1	2	2	1	3	0
0	0	0	0	0	0	0	0	0
0	0	0	0	0	0	0	0	0
1	6	6	10	13	4	6	7	0
0	0	1	0	0	0	0	0	0
0	0	0	0	0	0	0	0	0
1	1	2	0	0	0	0	0	0
7	10	11	7	5	3	3	7	5
0	1	1	0	0	0	0	0	0
0	0	0	0	0	0	0	0	0
0	0	0	0	0	0	0	0	0

0	0	0	0	0	0	0	0	0
1	4	4	14	8	4	7	5	3
1	1	0	1	1	2	3	0	0
6	11	14	10	13	5	4	5	3
37	101	59	46	35	26	19	13	8
0	0	1	0	0	1	1	0	0
45	93	96	83	50	48	28	29	13
8	0	0	7	0	0	0	5	0
0	0	0	0	0	0	0	0	0
0	2	1	1	1	0	4	0	1
0	0	0	0	0	0	0	0	0
0	0	0	0	1	0	0	0	0
0	0	0	0	0	0	0	0	1
0	0	0	0	0	0	0	0	0
17	41	39	43	33	39	24	32	30
1	1	2	0	4	6	6	3	1
0	0	1	0	0	0	0	0	0
0	0	0	0	0	0	0	0	0
0	0	0	0	0	0	0	0	0
26	43	39	46	39	35	25	30	29
0	1	0	0	0	0	0	0	0
0	0	0	0	0	0	0	0	0
0	0	0	0	0	0	0	0	0
0	0	0	0	0	0	0	0	0
0	0	0	0	0	0	0	0	0
0	0	0	0	0	0	0	0	0
1	0	0	0	0	0	0	0	0
0	0	0	0	0	0	0	1	0
1	1	0	2	0	1	0	0	0
0	0	0	0	0	0	0	0	0
0	0	0	0	0	0	0	0	0
0	0	0	0	0	0	0	0	0
0	0	0	0	0	0	0	0	0
7	9	6	6	4	1	1	3	1
0	0	0	0	0	0	0	0	0
0	0	0	0	0	0	0	0	0
0	0	0	0	0	0	0	0	0
0	1	1	0	2	1	0	1	0
213	557	363	301	184	146	121	104	52
17	65	59	45	47	30	29	29	17
0	0	0	0	0	0	0	0	0
0	0	0	0	0	0	0	0	0
0	0	4	0	1	0	0	1	0
0	0	0	0	0	0	0	0	0
0	0	0	0	0	0	0	0	0
0	0	0	0	0	0	0	0	0
0	1	0	0	0	2	0	0	0
0	0	0	0	1	1	1	0	0
1	3	2	2	2	2	3	2	0
1	0	0	2	0	0	0	1	0
2	2	4	2	3	2	0	1	0
0	0	0	0	0	1	0	0	0
0	0	0	0	0	0	0	0	0
0	2	1	1	0	0	2	0	0
0	0	0	0	0	0	0	0	0
67	165	190	178	104	100	84	60	70
0	0	0	0	0	0	0	0	0
0	0	0	0	0	0	0	0	0
0	0	0	0	0	0	0	0	0
0	0	0	0	0	0	0	0	0
4	6	3	2	2	1	1	2	5
4	9	14	8	6	4	5	3	3
0	0	0	0	0	0	0	0	0
0	0	0	0	0	0	0	0	1
0	0	0	0	0	0	0	0	0
0	0	0	0	0	0	0	0	0
0	0	1	0	0	2	0	0	0

0	0	0	0	0	0	0	0	0
0	0	0	0	0	0	0	0	0
0	0	0	0	0	0	0	0	0
0	0	0	0	0	1	0	0	0
0	0	0	0	0	0	0	0	0
5	17	10	8	4	2	2	2	3
0	0	0	0	0	0	0	0	0
0	0	0	0	0	0	0	0	0
0	0	0	0	0	0	0	0	0
0	0	0	0	0	0	0	0	0
0	0	2	2	2	0	0	1	0
0	0	0	0	0	0	0	0	0
0	0	0	0	0	0	0	0	0
0	0	0	0	0	0	0	0	0
0	1	0	1	0	1	1	0	0
0	0	0	0	0	0	0	0	0
0	0	0	0	0	0	0	0	0
0	0	1	0	0	0	0	2	0
0	0	0	1	0	0	0	0	0
0	0	0	0	0	0	0	0	0
0	0	0	0	0	0	0	0	0
2	3	0	0	1	1	0	0	0
1	2	2	2	0	0	0	1	0
0	1	0	0	0	0	0	0	0
0	0	0	0	0	0	0	0	0
0	0	0	0	0	0	0	0	0
0	0	0	0	0	0	0	0	0
0	0	0	0	0	0	0	0	0
1	0	0	0	0	0	0	0	0
130	255	176	121	81	70	52	37	17
7	22	20	12	8	0	1	7	3
79	235	113	102	61	65	62	80	51
5	23	15	14	6	4	7	5	4
0	0	0	0	0	0	0	0	0
20	49	20	22	12	10	10	7	8
3	4	0	0	0	1	0	0	0
1	0	0	0	0	0	0	0	0
0	0	0	0	0	0	1	0	0
0	0	0	0	0	0	0	0	0
0	0	0	0	0	0	0	0	0
0	0	0	0	0	0	0	0	0
0	0	0	0	0	0	0	0	0
1	0	1	0	0	0	0	0	1
0	0	0	0	0	0	0	0	0
0	0	0	0	0	0	0	0	0
0	0	4	2	0	2	0	0	0
2	26	10	10	7	6	3	5	0
0	0	0	0	0	0	0	0	0
0	0	0	0	0	0	0	0	0
9	17	11	15	8	6	11	10	2
4	4	4	2	1	0	2	0	1
0	4	2	1	2	0	0	0	1
0	0	0	0	0	0	0	0	0
0	0	0	0	0	0	0	0	0
0	0	0	0	0	0	0	0	0
1	2	1	3	3	1	0	0	2
0	0	0	0	0	0	0	0	0
1	0	0	0	0	0	0	0	0
0	0	0	0	0	0	0	1	0
0	1	0	0	0	0	1	0	0
0	0	0	0	0	0	0	0	0
0	0	0	0	0	0	0	0	0
0	0	0	0	0	0	0	0	0
0	0	0	0	0	0	0	0	0
1	1	1	0	0	1	0	0	0
0	0	0	0	0	0	0	0	0
0	1	0	0	0	0	0	0	0

0	0	0	0	0	0	0	0	0
7	18	32	18	6	8	6	8	5
20	48	12	23	16	18	8	8	4
0	0	0	0	0	0	0	0	0
0	0	1	5	1	1	3	2	0
0	0	0	0	0	0	0	0	0
8	21	5	11	9	8	7	7	4
41	163	84	87	51	58	36	30	21
2	9	9	13	2	4	5	5	2
45	86	87	49	48	35	46	35	25
0	0	2	0	3	0	3	2	0
0	0	1	0	0	0	0	0	0
0	0	0	0	0	0	0	0	0
0	0	0	0	0	0	0	0	0
0	0	0	0	0	0	0	0	0
0	0	1	0	0	0	0	0	0
0	0	1	0	0	0	0	0	0
0	1	0	0	0	0	0	0	0
0	0	0	0	0	0	0	0	0
0	0	0	0	0	0	0	0	0
0	0	0	0	1	0	0	0	0
2	2	3	1	0	0	1	0	0
0	0	0	0	0	0	0	0	0
1	2	2	1	1	3	0	2	0
3	8	10	7	14	9	8	6	1
0	0	0	0	0	0	0	0	0
4	9	5	4	1	1	1	1	0
0	1	0	0	0	0	0	0	0
0	2	0	4	2	0	4	0	2
0	0	0	1	0	0	2	1	1
0	0	0	0	0	0	0	0	0
0	0	0	0	0	0	0	0	0
0	0	0	0	0	0	0	0	0
0	2	0	0	0	0	0	0	0
2	0	0	0	0	0	0	0	0
0	0	0	0	0	0	0	0	0
16	31	1	21	10	18	9	10	0
2	11	2	2	3	3	2	0	2
0	0	0	0	0	0	0	0	0
0	0	0	0	0	0	0	0	0
0	0	0	0	0	0	0	0	0
8	16	9	11	7	6	8	4	1
3	2	4	3	6	5	2	1	0
0	0	0	0	0	0	0	0	0
0	0	0	0	0	0	0	0	0
0	1	0	0	1	0	0	0	0
0	0	0	0	0	0	0	0	0
0	0	0	0	0	0	0	0	0
0	0	0	0	0	0	0	0	0
1	0	2	0	0	0	0	0	0
0	3	0	0	2	0	0	0	0
0	0	0	0	0	0	0	0	0
1	3	0	0	0	1	0	0	0
60	146	144	154	98	109	71	55	40
0	0	0	0	0	0	0	0	0
0	0	0	0	0	0	0	0	0
0	0	0	0	0	0	0	0	0
0	0	0	0	0	0	0	0	0
0	2	0	0	0	0	0	0	0
5	0	13	15	12	2	8	2	6
0	0	0	0	0	0	0	0	0
0	2	0	0	1	0	0	1	0
13	29	15	13	8	8	12	8	6
0	0	0	0	0	0	0	0	0
4	12	9	7	8	2	7	3	1
4	9	9	2	3	3	2	1	0
0	0	1	2	0	0	0	0	1

0	0	0	0	0	0	0	0	0
0	0	0	0	0	0	0	0	0
0	0	0	0	0	0	0	0	0
3	2	3	8	3	3	1	0	0
4	2	0	1	1	0	0	0	0
0	0	0	0	0	0	0	0	0
0	0	0	0	0	0	0	0	0
0	0	1	0	0	0	0	0	0
0	0	0	3	1	0	0	0	0
0	0	0	0	0	0	0	0	0
0	4	2	0	0	2	0	0	0
0	0	0	0	0	0	0	0	0
0	0	0	0	0	0	0	0	0
0	0	0	0	0	0	0	0	0
2	4	2	1	0	2	0	0	1
0	0	0	0	0	0	0	0	0
0	0	0	0	0	0	0	0	0
1	1	0	0	1	0	0	0	0
4	22	9	5	6	7	2	3	1
1	4	2	4	0	0	0	0	0
0	1	0	0	0	0	0	0	0
0	0	0	0	0	0	0	0	0
4	20	8	4	3	2	1	2	1
0	0	0	0	0	0	0	0	0
0	0	0	0	0	0	0	0	0
0	0	0	0	0	0	0	0	0
1	1	0	0	0	0	0	0	0
0	0	3	0	0	0	0	0	0
0	0	0	0	0	0	0	0	0
1	0	0	0	1	0	0	0	0
0	0	0	0	0	0	0	0	0
0	0	0	0	0	0	0	0	0
0	0	0	0	0	0	0	0	0
0	0	0	0	0	0	0	0	0
0	0	0	0	1	0	0	0	0
0	0	0	0	0	0	0	0	0
0	0	2	3	1	2	2	3	0
0	0	0	0	0	0	0	0	0
0	0	0	0	0	0	2	0	0
0	0	0	1	0	0	0	0	0
0	0	0	0	0	0	0	0	0
0	0	0	0	0	0	0	0	0
3	1	2	0	3	2	0	2	1
0	0	0	0	0	0	0	0	0
0	0	0	0	0	0	0	0	0
0	4	6	5	10	3	1	1	0
0	0	0	2	2	0	0	1	0
0	1	0	0	1	0	0	1	0
0	0	0	0	0	0	0	0	0
0	0	0	0	0	0	0	0	0
0	0	1	0	0	1	0	0	0
0	0	0	0	1	0	0	0	0
0	0	0	0	0	0	0	0	0
0	1	1	0	0	0	1	1	0
0	0	0	1	0	0	0	0	0
1	2	2	3	1	0	1	1	1
0	1	2	1	0	0	0	0	0
0	0	0	0	0	0	0	0	0
0	0	0	0	0	0	0	0	0
0	0	0	0	0	0	0	0	0
0	0	0	0	0	0	0	0	0
0	0	0	0	0	0	0	0	0
0	0	0	0	0	0	0	0	0
0	1	0	0	0	0	0	0	0
0	0	0	0	0	0	0	0	0
0	0	0	0	0	0	0	0	0
0	0	0	0	0	0	0	0	0

0	0	0	0	0	0	0	0	0
0	0	0	0	0	0	0	0	0
0	0	0	0	0	0	0	0	0
0	0	0	0	0	0	0	0	0
0	0	0	0	0	0	0	0	0
0	0	0	0	0	0	0	0	0
0	0	0	0	0	0	0	0	0
0	0	0	0	0	0	0	0	0
0	0	0	0	0	0	0	0	0
0	0	0	0	0	0	0	0	0
0	0	0	0	0	0	0	0	0
0	0	0	0	0	0	0	0	0
0	0	0	0	0	0	0	0	0
0	0	0	0	0	0	0	0	0
0	0	0	0	0	0	0	0	0
0	0	0	0	0	0	0	0	0
0	0	0	0	0	0	0	0	0
0	0	0	0	0	0	0	0	0
0	0	0	0	0	0	0	0	0
0	0	0	0	0	0	0	0	0
0	0	0	0	0	0	0	0	0
0	0	1	1	0	0	0	0	0
0	0	0	0	0	0	0	0	0
0	0	0	0	0	0	0	0	0
0	0	0	0	0	0	0	0	0
38	76	67	59	39	34	27	27	24
0	0	0	0	0	0	0	0	0
0	0	0	0	1	1	0	1	1
0	0	0	0	0	0	0	0	0
0	0	0	0	0	0	0	0	0
5	8	5	6	4	4	4	3	1
0	0	0	0	0	0	0	0	0
0	2	1	0	0	0	0	0	1
0	0	0	0	0	0	0	0	0
0	0	0	0	0	0	0	0	0
1	0	1	0	0	1	1	0	0
0	0	0	0	0	0	0	0	0
0	0	0	0	0	1	1	0	0
3	6	4	3	3	1	3	1	0

Pir1_M1_60_65	Pir1_M1_65_70	Pir1_M1_70_75	Pir1_M1_75_80	Pir1_M1_80_85	Pir1_M1_85_90	Pir1_M1_90_95	Pir1_M1_95_10	Pir1_M1_100_1
0	1	0	0	0	1	1	0	0
0	0	0	0	0	0	0	0	0
6	6	6	2	8	10	11	8	3
0	0	0	0	0	0	0	0	0
2	1	1	1	0	0	0	1	1
0	0	0	0	0	0	0	0	0
0	0	0	0	0	0	0	0	1
0	0	0	0	0	0	0	0	0
0	0	0	0	0	0	0	0	0
0	0	0	0	0	0	0	0	0
5	2	0	0	0	0	0	1	1
0	0	0	0	0	0	0	0	0
0	0	0	0	0	0	0	0	0
0	0	0	0	0	0	0	0	0
0	0	0	0	0	0	0	0	0
7	8	10	4	2	4	3	5	4
3	4	2	3	2	4	2	1	6
0	0	0	0	0	0	0	0	0
0	0	0	0	0	0	1	0	0
0	0	0	0	0	0	0	0	0
5	5	3	1	1	3	5	6	4
0	0	0	0	0	0	0	0	0
9	7	5	6	2	12	11	3	5
0	0	0	0	0	0	0	0	0
0	0	0	0	1	0	0	0	0
7	3	2	3	2	3	2	0	0
13	0	0	1	0	0	0	0	1
3	3	2	0	0	0	0	0	1
0	0	0	0	0	0	0	0	0
0	0	0	0	0	0	0	0	0
0	0	0	0	0	0	0	0	0
0	0	0	0	0	0	0	0	0
1	0	0	0	0	0	0	0	0
0	0	0	0	0	0	0	0	0
0	0	0	0	0	0	0	0	0
0	0	0	0	0	0	0	0	0
108	72	55	32	22	23	17	11	12
0	0	0	0	0	0	0	0	0
0	0	0	0	0	0	0	0	0
22	21	24	17	14	10	12	2	4
0	0	0	0	0	0	0	0	0
0	0	0	0	0	0	0	0	0
0	0	0	0	0	0	0	0	0
0	0	0	0	0	0	0	0	1
0	0	0	0	0	0	0	0	0
1	0	0	0	0	0	0	0	0
2	0	0	0	0	1	0	1	0
0	0	0	0	0	0	0	0	0
0	0	0	0	0	0	0	0	0
5	7	8	3	6	3	4	2	2
1	0	0	0	0	0	0	0	0
0	0	0	0	0	0	0	0	0
0	0	0	0	0	0	0	0	0
0	0	0	0	0	0	0	0	0
0	0	0	0	0	0	0	0	1
0	0	0	0	0	0	0	1	0
0	0	0	0	0	0	0	0	0
0	0	0	0	0	0	0	0	0
0	2	0	3	4	1	3	3	0
0	0	0	0	0	0	0	0	0
0	0	0	0	0	0	0	0	0
0	0	0	0	0	0	0	0	0
12	6	2	1	1	0	3	1	0
0	0	0	0	0	0	0	0	0
0	0	0	0	0	0	0	0	0
0	0	0	0	0	0	0	0	0

0	0	0	0	0	0	0	0	0
9	8	5	2	3	9	5	4	4
0	0	0	1	1	2	2	2	1
2	2	1	1	0	3	1	0	1
5	6	5	5	3	1	2	6	5
0	0	0	0	0	0	0	0	0
16	15	4	7	7	5	6	9	8
0	0	0	0	1	0	0	0	0
0	0	0	0	0	0	0	0	0
0	0	0	0	1	0	1	1	2
0	0	0	0	0	0	0	0	0
0	0	0	0	0	0	0	0	0
0	0	0	0	0	0	0	0	0
0	0	0	0	0	0	0	0	0
40	28	33	22	14	23	23	12	6
3	2	2	0	1	1	2	0	3
0	0	0	0	0	0	0	0	0
0	0	0	0	0	0	0	0	0
0	0	0	0	0	0	0	0	0
36	28	27	28	26	20	12	9	12
0	0	0	0	0	0	0	0	0
0	0	0	0	0	0	0	0	0
0	0	0	0	0	0	0	0	0
0	0	0	0	0	0	0	0	0
0	0	0	0	0	0	0	0	0
0	0	0	0	0	0	0	0	0
0	0	0	0	0	0	0	0	0
1	0	0	0	1	0	0	0	0
0	0	0	0	0	0	0	0	0
0	0	0	0	0	0	0	0	0
0	0	0	0	0	0	0	0	0
0	0	0	0	0	0	0	0	0
0	0	0	0	0	0	0	0	0
1	2	2	5	2	6	1	1	1
0	0	0	0	0	0	0	0	0
0	0	0	0	0	0	0	0	0
0	0	0	0	0	0	0	0	0
0	0	0	0	1	0	0	0	0
62	34	29	22	23	20	13	25	17
30	18	10	6	19	13	14	12	8
0	0	0	0	0	0	0	0	0
0	0	0	0	0	0	0	0	0
0	0	1	0	0	2	1	1	1
0	0	0	0	0	0	0	0	0
0	0	0	0	0	0	0	0	0
0	0	0	0	0	0	0	0	0
0	0	0	0	0	0	0	0	0
2	1	1	1	2	1	2	0	0
1	1	0	1	0	0	0	0	0
0	0	0	1	0	0	0	0	0
1	1	0	0	1	0	2	0	0
0	0	0	0	0	0	0	0	0
0	0	0	0	0	0	0	0	0
0	0	0	0	0	0	0	0	0
0	0	0	0	0	0	0	0	0
110	78	62	51	44	55	44	36	33
0	0	0	0	0	0	0	0	0
0	0	0	0	0	0	0	0	0
0	0	0	0	0	0	1	0	0
0	0	0	0	0	0	0	0	0
3	2	2	0	2	0	1	1	1
2	1	1	1	1	1	1	2	0
0	0	0	0	0	0	0	0	0
0	0	0	0	0	0	0	0	0
0	0	0	0	0	0	0	0	0
0	0	0	0	0	0	0	0	0
0	0	0	0	0	0	0	0	0

0	0	0	0	0	0	0	0	0
0	0	0	0	0	0	0	0	0
0	0	0	0	0	0	0	0	0
0	0	0	0	0	0	0	0	0
0	0	0	0	0	0	0	0	0
0	2	1	1	0	0	1	1	0
0	0	0	0	0	0	0	0	0
0	0	0	0	0	0	0	0	0
0	0	0	0	0	0	0	0	0
0	0	0	0	0	0	0	0	0
0	0	0	0	0	0	1	0	0
0	0	0	0	0	0	0	2	0
0	0	0	0	0	0	0	0	0
0	0	0	0	0	0	0	0	0
0	0	0	0	0	0	0	0	0
0	0	0	0	0	0	0	0	0
0	0	0	0	0	0	0	0	0
0	0	0	0	0	0	0	0	0
0	0	0	0	0	0	0	0	0
0	0	0	0	0	0	0	0	0
0	0	0	0	0	0	0	0	0
0	0	0	0	0	0	0	0	0
1	1	0	1	0	1	0	0	0
0	0	0	0	0	0	0	0	0
0	0	0	0	0	0	0	0	0
0	0	0	0	0	0	0	0	0
0	0	0	0	0	0	0	0	0
0	0	0	0	0	0	0	0	0
0	0	0	0	0	0	0	0	0
13	12	6	10	4	2	2	8	5
6	1	1	2	0	0	0	0	0
73	65	46	25	26	10	28	26	18
8	3	9	5	2	7	3	3	1
0	0	0	0	0	0	0	0	0
6	2	1	0	1	1	0	2	2
0	0	0	0	0	0	0	0	0
1	1	1	0	0	0	0	0	0
0	1	0	0	0	0	0	0	0
0	0	0	0	0	0	0	0	0
0	0	0	0	0	0	0	0	0
0	0	0	0	0	0	0	0	0
0	0	0	0	0	0	0	0	0
0	0	0	0	0	0	0	0	0
0	0	0	0	0	0	0	0	0
0	0	0	0	0	0	0	0	0
0	0	0	0	0	0	0	0	0
1	1	0	0	0	2	1	0	1
0	0	0	0	0	0	0	0	0
0	0	0	0	0	0	0	0	0
2	2	0	0	0	2	1	1	0
1	0	1	0	0	0	0	0	1
0	0	0	0	1	0	0	0	1
0	0	0	0	0	0	0	0	0
0	0	0	0	0	0	0	0	0
0	0	0	0	0	0	0	0	0
2	2	1	0	2	1	1	0	0
0	0	0	0	0	0	0	0	0
1	0	0	0	0	0	0	0	0
0	0	0	0	0	0	0	0	0
0	0	0	0	0	0	0	0	0
0	0	0	0	0	0	0	0	0
0	0	0	0	0	0	0	0	0
0	0	0	0	0	0	0	0	0
0	0	0	0	0	0	0	0	0
0	0	0	0	0	0	0	0	0
0	0	0	0	0	0	0	0	0
0	0	0	0	0	0	0	0	0

0	0	0	0	0	0	0	0	0
2	2	1	0	1	1	1	0	0
2	6	5	3	3	0	2	3	0
0	0	0	0	0	0	0	0	0
0	0	0	0	0	0	0	1	0
0	0	0	0	0	0	0	0	0
4	4	2	2	1	0	0	3	1
13	11	9	15	11	7	10	6	7
2	1	2	6	2	0	1	3	3
29	25	14	14	8	6	11	14	2
0	2	0	1	1	1	0	0	0
1	0	1	0	0	0	0	0	0
1	0	0	0	0	0	0	0	0
0	0	0	0	0	0	0	0	0
0	0	0	0	0	0	0	0	0
0	0	0	0	0	0	0	0	0
1	0	0	0	0	0	0	0	0
0	0	0	0	0	0	0	0	0
0	0	0	0	0	0	0	0	0
0	0	0	0	0	0	0	0	0
0	0	0	0	0	0	0	0	0
0	0	0	0	0	0	0	0	0
0	0	0	0	0	0	0	0	0
0	1	1	0	0	0	1	0	0
6	2	1	4	1	0	0	1	2
0	0	0	0	0	0	0	0	0
0	0	0	0	1	1	0	1	0
0	0	0	0	0	0	0	0	0
1	1	1	1	3	0	0	6	0
0	0	1	0	0	0	0	0	0
0	0	0	0	0	0	0	0	0
0	0	0	0	0	0	0	0	0
0	0	0	0	0	0	0	0	0
1	0	1	0	0	0	0	0	0
0	0	0	0	0	0	0	0	0
0	0	0	0	0	0	0	0	0
4	4	3	0	0	2	1	3	1
3	0	1	2	2	1	1	1	0
0	0	0	0	0	0	0	0	0
0	0	0	0	0	0	0	0	0
0	0	0	0	0	0	0	0	0
0	5	5	0	5	1	1	4	2
4	0	0	2	0	0	1	0	0
0	0	0	0	0	0	0	0	0
0	0	0	0	0	0	0	0	0
0	0	0	0	0	0	0	0	0
0	0	0	0	0	0	0	0	0
0	0	0	0	0	0	0	0	0
0	0	0	0	0	0	0	0	0
0	0	0	0	0	0	0	0	0
0	0	0	0	0	0	0	0	0
0	0	0	0	0	0	0	0	0
0	0	0	0	0	0	0	0	0
33	29	22	14	9	14	12	16	18
0	0	0	0	0	0	0	0	0
0	0	0	0	0	0	0	0	0
0	0	0	0	0	0	0	0	0
0	0	0	0	0	0	0	0	0
0	0	0	0	0	0	0	0	0
3	5	6	0	0	2	0	1	0
0	0	0	0	0	0	0	0	0
0	1	0	0	0	0	0	0	0
5	12	7	5	5	0	1	4	1
0	0	0	0	0	0	0	0	0
1	0	0	0	0	0	0	3	1
0	0	0	0	0	0	0	2	0
1	0	0	0	0	0	0	0	0

0	0	0	0	0	0	0	0	0
0	0	0	0	0	0	0	0	0
0	0	0	0	0	0	0	0	0
1	1	0	2	0	0	0	0	0
0	0	0	0	0	0	0	0	0
0	0	0	0	0	0	0	0	0
0	0	0	0	0	0	0	0	0
0	0	0	0	0	0	0	0	0
0	0	0	0	0	0	0	0	0
0	0	0	0	0	0	0	0	0
0	0	0	0	0	0	0	0	0
0	0	0	0	0	0	0	0	0
0	0	0	0	0	0	0	0	0
0	0	0	0	0	0	0	0	0
0	0	2	0	1	0	1	1	1
0	0	0	0	0	0	0	0	0
0	0	0	0	0	0	0	0	0
0	0	0	0	0	0	0	0	0
3	0	1	0	1	0	1	0	1
0	0	1	1	0	0	0	1	0
0	0	0	0	0	0	0	0	0
0	0	1	0	0	0	0	0	0
0	0	1	2	0	0	0	0	1
0	0	0	0	0	0	0	0	0
0	0	0	0	0	0	0	0	0
0	0	0	0	0	0	0	0	0
0	0	0	0	0	0	0	0	0
0	0	0	0	0	0	0	0	0
0	0	0	0	0	0	0	0	0
0	0	1	0	0	0	0	0	0
0	0	0	0	0	0	0	0	0
0	0	0	0	0	0	0	0	0
0	0	0	0	0	0	0	0	0
0	0	0	0	0	0	0	0	0
0	0	0	0	0	0	0	0	0
0	0	0	0	0	0	0	0	0
0	0	0	0	0	0	0	0	0
0	0	0	0	0	0	0	0	0
0	0	0	0	0	0	0	0	0
0	0	0	0	0	0	0	0	0
0	0	0	0	0	0	0	0	0
0	0	0	0	0	0	0	0	0
1	0	1	1	0	0	1	2	0
0	0	0	0	0	0	0	0	0
0	0	0	0	0	0	0	0	0
1	4	1	2	0	1	0	1	0
0	0	1	0	0	0	0	0	0
0	1	0	0	2	0	0	0	0
0	0	0	0	0	0	1	0	0
0	0	0	0	0	0	0	0	0
1	1	0	0	1	2	0	0	0
0	0	0	0	0	0	0	0	0
0	0	0	0	0	0	0	0	0
0	0	0	0	0	0	0	0	1
0	0	0	0	0	0	0	0	0
0	0	1	0	1	0	0	0	0
1	0	1	0	0	0	0	0	0
0	0	0	0	0	0	0	0	1
0	0	0	0	0	0	0	0	0
0	0	0	0	0	0	0	0	0
0	0	0	0	0	0	0	0	0
0	0	0	0	0	0	0	0	0
0	0	0	0	0	0	0	0	0
0	0	0	0	0	0	0	0	0
0	0	0	0	0	0	0	0	0
0	0	0	0	0	0	0	0	0
0	0	0	0	0	0	0	0	0

0	0	0	0	0	0	0	0	0
0	0	0	0	0	0	0	0	0
0	0	0	0	0	0	0	0	0
0	0	0	0	0	0	0	0	0
0	0	0	0	0	0	0	0	0
0	0	0	0	0	0	0	0	0
0	0	0	0	0	0	0	0	0
0	0	0	0	0	0	0	0	0
0	0	0	0	0	0	0	0	0
0	0	0	0	0	0	0	0	0
0	0	0	0	0	0	0	0	0
0	0	0	0	0	0	0	0	0
0	0	0	0	0	0	0	0	0
0	0	0	0	0	0	0	0	0
0	0	0	0	0	0	0	0	0
0	0	0	0	0	0	0	0	0
0	0	0	0	0	0	0	0	0
0	0	0	0	0	0	0	0	0
0	0	0	0	0	0	0	0	0
0	0	0	0	0	0	0	0	0
0	0	0	0	0	0	0	0	0
0	0	1	0	0	0	0	0	0
0	0	0	0	0	0	0	0	0
0	0	0	0	0	0	0	0	0
0	0	0	0	0	0	0	0	0
30	30	22	13	8	9	7	12	6
0	0	0	0	0	0	0	0	0
0	1	1	1	0	0	0	0	1
0	0	0	0	0	0	0	0	0
0	0	0	0	0	0	0	0	0
1	0	0	0	2	0	0	1	0
0	0	0	0	0	0	0	0	0
0	0	0	0	0	0	0	0	0
0	0	0	0	0	0	0	0	0
0	0	0	0	0	0	0	0	0
0	0	0	0	0	0	0	0	0
0	0	0	0	0	0	0	0	0
0	0	0	0	0	0	0	0	0
0	1	0	1	0	0	1	0	0

Pir1_M1_105_:	Pir1_M1_110_:	Pir1_M1_115_:	Pir1_M1_120_:	Pir1_M1_125_:	Pir1_M1_130_:	Pir1_M1_135_:	Pir1_M1_140_:	Pir2_M53_0_2
1	0	1	0	0	0	0	0	1
0	0	0	0	0	0	0	0	0
4	8	3	2	1	2	6	8	12
0	0	0	0	0	0	0	0	0
1	0	1	0	0	0	1	0	1
0	0	0	0	0	0	0	0	0
0	0	0	0	0	0	0	0	0
0	0	0	0	0	0	0	0	0
0	0	0	0	0	0	0	0	0
0	0	0	0	0	0	0	0	0
1	2	1	0	0	0	0	0	1
0	0	0	0	0	0	0	0	0
0	0	0	0	0	0	0	0	0
0	0	0	0	0	0	0	0	0
0	0	0	0	0	0	0	0	0
2	2	3	2	1	2	3	3	5
4	1	0	1	5	5	1	4	2
0	0	0	0	0	0	0	0	0
0	0	0	1	0	0	0	0	0
0	0	0	0	0	0	0	0	0
3	3	1	2	2	1	1	1	2
0	0	0	0	0	0	0	0	0
3	4	0	5	1	4	2	10	2
0	0	0	0	0	0	0	0	0
0	0	0	0	0	1	0	0	0
3	2	2	0	0	1	4	0	7
0	0	0	0	0	0	0	0	1
0	1	0	2	1	0	0	0	1
0	0	0	0	0	0	0	0	0
0	0	0	0	0	0	0	0	0
0	0	0	0	0	0	0	0	0
0	0	0	0	0	0	1	0	0
0	0	0	0	0	0	0	0	0
0	0	0	0	0	0	0	0	0
0	0	0	0	0	0	0	0	0
0	0	0	0	0	0	0	0	0
7	13	6	3	6	12	11	5	1
0	0	0	0	0	0	0	0	0
0	0	0	0	0	0	0	0	0
3	9	2	5	4	8	2	5	2
0	0	0	0	0	0	0	0	0
0	0	0	0	0	0	0	0	0
0	0	0	0	0	0	0	0	0
0	0	0	0	1	1	0	0	0
0	0	0	0	0	0	1	0	1
0	0	0	0	0	0	0	0	0
1	1	0	1	1	0	2	1	0
0	0	0	0	0	0	0	0	0
0	0	0	0	0	0	0	0	0
3	5	2	2	3	4	2	2	4
0	0	0	0	0	0	0	0	1
0	0	0	0	0	0	0	0	0
0	1	0	0	0	0	0	0	0
0	0	0	0	0	0	0	0	0
0	1	0	0	0	0	0	0	0
0	0	0	0	0	0	1	0	0
0	0	0	0	0	0	0	0	0
0	0	0	0	0	0	0	0	0
1	5	1	1	0	1	3	0	1
0	0	0	0	0	0	0	0	0
0	0	0	0	0	0	0	0	0
0	0	0	0	0	0	0	0	2
0	0	0	1	1	0	1	0	2
0	0	0	0	0	0	0	0	0
0	0	0	0	0	0	0	0	0
0	0	0	0	0	0	0	0	1

0	0	0	0	0	0	0	0	0
4	4	5	5	4	12	5	2	0
1	1	1	0	0	2	0	1	31
2	0	0	0	0	0	3	1	2
0	4	6	3	1	2	7	3	43
0	0	1	0	0	0	0	0	5
3	8	0	3	4	5	12	4	24
1	0	0	0	0	0	0	0	28
0	0	0	0	0	0	0	0	0
0	1	1	1	1	1	4	1	0
0	0	0	0	0	0	0	0	0
0	0	0	0	0	0	0	0	0
1	0	0	0	1	1	0	0	0
0	0	0	0	0	0	0	0	0
7	5	6	2	4	7	11	17	3
2	2	4	2	1	1	3	1	0
0	0	0	0	0	0	0	0	1
0	0	0	0	0	0	0	0	0
0	0	0	0	0	0	0	0	0
4	13	7	6	3	12	7	8	5
0	0	0	0	0	0	0	0	0
0	0	0	0	0	0	0	0	0
0	0	0	0	0	0	0	0	0
0	0	0	0	0	0	0	0	0
0	0	0	0	0	0	0	0	0
0	0	0	0	0	0	0	0	0
0	0	0	0	0	0	0	0	2
0	0	0	1	0	0	0	0	0
0	1	0	0	0	0	0	0	0
0	0	0	0	0	0	0	0	0
0	0	0	0	0	0	0	0	0
0	0	0	0	0	0	0	0	0
0	0	0	0	0	0	0	0	0
1	0	0	0	0	0	0	0	0
0	0	0	0	0	0	0	0	0
0	0	0	0	0	0	0	0	0
0	0	0	0	0	0	0	0	0
1	0	0	0	2	0	0	0	0
7	19	11	8	8	10	41	6	198
5	9	8	4	4	8	11	8	14
0	0	0	0	0	0	0	0	0
0	0	0	0	0	0	0	0	0
2	0	0	0	0	1	2	4	0
0	0	0	0	0	0	0	0	0
0	0	0	0	0	0	0	0	0
0	0	0	0	0	0	0	0	0
0	0	0	0	0	0	0	0	0
0	0	2	3	3	3	0	2	0
0	0	0	0	0	0	0	0	2
0	1	1	0	0	0	0	0	0
0	0	0	0	0	1	0	1	4
0	0	0	0	0	0	0	0	0
0	0	0	0	0	0	0	0	0
0	0	0	0	0	0	0	0	0
0	0	0	0	0	0	0	0	0
33	34	28	19	36	48	45	28	57
0	0	0	0	0	0	1	0	0
0	0	0	0	0	0	0	0	0
0	0	0	0	0	2	0	0	1
0	0	0	0	0	0	0	0	0
0	0	0	0	0	0	0	0	4
0	1	1	0	0	0	0	0	8
0	0	0	0	0	0	0	0	1
0	0	0	0	0	0	0	0	0
0	0	0	0	0	0	0	0	0
0	0	0	0	0	0	0	0	0
0	0	0	0	0	0	0	0	0

0	0	0	0	0	0	0	0	0
0	0	0	0	0	0	0	0	0
0	0	0	0	0	0	0	0	0
0	0	0	0	0	0	0	0	0
0	0	0	0	0	0	0	0	0
0	0	0	0	1	0	0	0	3
0	0	0	0	0	0	0	0	0
0	0	0	0	0	0	0	0	0
0	0	0	0	0	0	0	0	0
0	0	0	0	0	0	0	0	0
0	0	0	0	0	0	1	0	0
0	0	0	0	0	0	0	0	0
0	0	0	0	0	0	0	0	0
0	0	0	0	0	0	0	0	0
0	1	0	0	0	0	0	0	0
0	0	0	0	0	0	0	0	0
0	0	0	0	0	0	0	0	0
0	0	0	0	0	0	0	0	0
0	0	0	0	0	0	0	0	0
0	0	0	0	0	0	0	0	0
0	0	0	0	0	0	0	0	0
0	0	0	0	0	0	0	0	4
0	0	0	0	0	0	0	1	1
0	0	0	0	0	0	0	0	2
0	0	0	0	0	0	0	0	0
0	0	0	0	0	0	0	0	0
0	0	0	0	0	0	0	0	0
0	0	0	0	0	0	0	0	0
0	0	0	0	0	0	0	0	0
2	7	5	1	2	2	30	4	83
1	0	1	0	0	0	0	1	10
5	15	12	4	6	14	33	20	30
0	2	0	0	1	0	3	2	2
0	0	0	0	0	0	0	0	4
1	1	1	1	1	0	4	1	17
0	1	0	0	0	0	0	0	0
0	0	0	0	0	0	0	0	0
0	0	0	0	1	0	0	0	0
0	0	0	0	0	0	0	0	1
0	0	0	0	0	0	0	0	0
0	0	0	0	0	0	0	0	0
0	0	0	0	0	0	0	0	0
0	0	0	0	0	0	0	0	0
0	0	0	0	0	0	0	0	0
0	0	0	0	0	0	0	0	0
0	0	0	0	0	0	0	0	0
0	0	0	0	0	0	0	0	5
0	0	0	0	0	0	0	0	0
0	0	0	0	0	0	0	0	0
2	1	0	0	1	0	1	1	2
0	1	0	1	0	0	0	0	1
0	0	0	0	0	0	0	0	0
0	0	0	0	0	0	0	0	0
0	0	0	0	0	0	0	0	0
0	0	0	0	0	0	0	0	1
2	0	0	0	1	1	0	2	0
0	0	0	0	0	0	0	0	0
0	0	0	0	0	0	0	0	0
1	0	0	0	0	0	0	0	0
0	0	0	0	0	0	0	0	0
0	0	0	0	0	0	0	0	0
0	0	0	0	0	0	0	0	0
0	0	0	0	0	0	0	0	0
0	0	0	0	0	0	0	0	0
0	0	0	0	0	0	0	0	0
0	0	0	0	0	0	0	0	1
0	0	0	0	0	0	0	0	0

0	0	0	0	0	0	0	0	0
1	1	0	0	0	0	3	0	2
0	4	2	1	0	0	6	1	2
0	0	0	0	0	0	0	0	0
0	0	0	0	0	0	0	0	0
0	0	0	0	0	0	0	0	0
0	2	3	2	0	1	1	1	4
2	6	4	0	3	4	12	3	12
0	1	1	0	2	0	1	0	0
4	6	1	5	4	4	7	3	19
1	2	1	3	0	1	4	1	0
0	1	0	0	0	0	0	0	0
0	0	0	0	0	0	0	0	0
0	0	0	0	0	0	0	0	0
0	0	0	0	0	0	0	0	0
0	0	0	0	0	0	0	0	0
0	0	0	0	0	0	0	0	0
0	0	0	0	0	0	0	0	0
0	0	0	0	0	0	0	0	0
0	0	0	0	0	0	0	0	0
0	0	0	0	0	0	0	0	0
0	0	0	0	0	1	0	0	0
0	0	0	0	0	0	0	0	0
0	0	0	1	0	0	0	0	1
2	1	0	2	0	0	1	1	7
0	0	0	0	0	0	0	0	0
0	0	0	0	0	0	0	0	0
0	0	0	0	0	0	0	0	0
1	5	7	0	0	3	14	6	0
0	1	0	0	0	0	0	3	0
0	0	0	0	0	0	0	0	0
0	0	0	0	0	0	0	0	0
0	0	0	0	0	0	0	0	0
0	0	0	0	0	0	0	0	0
0	0	0	0	0	0	0	0	0
0	0	0	0	0	0	0	0	0
0	0	2	0	1	0	3	0	3
0	0	0	1	0	2	0	1	1
0	0	0	0	0	0	0	0	0
0	0	0	0	0	0	0	0	0
0	0	0	0	0	0	0	0	0
0	0	1	0	1	0	1	0	3
2	0	0	0	0	0	0	0	0
0	0	0	0	0	0	0	0	0
0	0	0	0	0	0	0	0	0
0	0	0	0	0	0	0	0	0
0	0	0	0	0	0	0	0	0
0	0	0	0	0	0	0	0	1
0	0	0	0	0	0	0	0	0
0	0	0	0	0	0	0	0	0
0	0	0	0	0	0	0	0	2
0	0	0	0	0	0	0	0	0
0	0	0	0	0	0	0	0	1
9	5	11	8	11	13	18	13	22
0	0	0	0	0	0	0	0	0
0	0	0	0	0	0	0	0	0
0	0	0	0	0	0	0	0	0
0	0	0	0	0	0	0	0	0
0	0	0	0	0	0	0	0	0
0	2	0	1	0	0	1	0	0
1	0	0	0	0	0	0	0	0
0	0	0	0	0	0	0	0	0
1	2	5	0	2	0	3	1	4
0	0	0	0	0	0	0	0	0
0	0	0	0	0	0	0	0	9
0	0	0	0	0	0	0	0	0
0	1	0	0	0	0	0	0	0

0	0	0	0	0	0	0	0	0
0	0	0	0	0	0	0	0	0
0	0	0	0	0	0	0	0	0
1	0	0	0	0	0	2	0	1
0	0	0	0	0	0	0	0	0
0	0	0	0	0	0	0	0	0
0	0	0	0	0	0	0	0	0
0	0	0	0	0	0	0	0	0
0	0	0	0	0	0	0	0	0
0	0	0	0	0	0	0	0	0
0	0	0	0	0	0	0	0	0
0	0	0	0	0	0	0	0	0
0	0	0	0	0	0	0	0	0
0	0	0	0	0	0	0	0	0
0	0	0	1	0	2	1	0	5
0	0	0	0	0	0	0	0	0
0	0	0	0	0	0	0	0	0
0	0	0	0	0	0	0	0	0
1	1	0	0	0	1	0	0	0
0	0	0	0	0	0	0	0	1
0	0	0	0	0	0	0	0	1
0	0	0	0	0	0	0	3	0
0	0	0	0	0	0	1	0	5
0	0	0	0	0	0	0	0	1
0	0	0	0	0	0	0	0	0
0	0	0	0	0	0	0	0	0
0	0	0	0	0	0	0	0	0
0	0	0	0	0	0	0	0	0
0	0	0	0	0	0	0	0	0
0	0	0	0	0	0	0	0	0
0	0	0	0	0	0	0	0	0
0	0	0	0	0	0	0	0	0
0	0	0	0	0	0	0	0	0
0	0	0	0	0	0	0	0	0
0	0	0	0	0	0	0	0	0
0	0	0	0	0	0	0	0	0
0	0	0	0	0	0	0	0	0
0	0	0	0	0	0	0	0	0
0	0	0	0	0	0	0	0	0
0	0	0	0	0	0	0	0	0
0	0	0	0	0	0	0	0	0
0	0	0	0	0	0	0	0	0
0	1	1	0	1	0	1	0	1
0	0	0	0	0	0	0	0	0
0	0	0	0	0	0	0	0	0
1	2	0	0	1	0	1	1	0
0	0	0	0	1	0	0	0	0
0	0	0	0	0	1	0	0	0
0	0	0	0	0	0	0	0	0
0	0	0	0	0	0	0	0	0
2	0	0	1	0	0	1	0	0
0	0	0	0	0	0	0	0	0
0	0	0	0	0	0	0	0	0
0	0	1	0	1	0	1	0	0
0	0	0	0	0	0	0	0	0
0	2	1	1	0	1	4	0	1
0	0	0	0	0	0	2	0	0
0	0	0	0	0	1	0	0	0
0	0	0	0	0	0	0	0	0
0	0	0	0	0	0	0	0	0
0	0	0	0	0	0	0	0	0
0	0	0	0	0	0	0	0	0
0	0	0	0	0	0	0	0	0
0	0	0	0	0	0	0	0	0
0	0	0	0	0	0	0	0	0
0	0	0	0	0	0	0	0	0
0	0	0	0	0	0	0	0	0

0	0	0	0	0	0	0	0	0
0	0	0	0	0	0	0	0	0
0	0	0	0	0	0	0	0	0
0	0	0	0	0	0	0	0	0
0	0	0	0	0	0	0	0	0
0	0	0	0	0	0	0	0	0
0	0	0	0	0	0	0	0	0
0	0	0	0	0	0	0	0	0
0	0	0	0	0	0	0	0	0
0	0	0	0	0	0	0	0	0
0	0	0	0	0	0	0	0	0
0	0	0	0	0	0	0	0	0
0	0	0	0	0	0	0	0	0
0	0	0	0	0	0	0	0	0
0	0	0	0	0	0	0	0	0
0	0	0	0	0	0	0	0	0
0	0	0	0	0	0	0	0	0
0	0	0	0	0	0	0	0	0
0	0	0	0	0	0	0	0	0
0	0	0	0	0	0	0	0	0
0	0	0	0	0	0	0	0	0
0	0	0	0	0	0	0	0	0
0	0	0	0	0	0	0	0	0
0	0	0	0	0	0	0	0	0
0	0	0	0	0	0	0	0	0
2	2	4	4	3	7	9	4	0
0	0	0	0	0	0	0	0	0
1	0	0	0	1	0	0	0	0
0	0	0	0	0	0	0	0	0
0	0	0	0	0	0	0	0	0
0	1	0	0	0	0	0	1	4
0	0	0	0	0	0	0	0	0
0	0	0	0	0	0	0	0	0
0	0	0	0	0	0	0	0	0
0	0	0	0	0	0	0	0	0
0	0	0	0	0	0	0	0	0
0	0	0	0	0	0	0	0	0
1	0	0	0	0	1	0	0	0
0	0	0	0	0	0	0	0	3

Pir2_M53_2_4	Pir2_M53_4_6	Pir2_M53_6_8	Pir2_M53_8_1(	Pir2_M53_10_1	Pir2_M53_12_1	Pir2_M53_14_1	Pir2_M53_16_1	Pir2_M53_18_2
8	1	2	4	4	4	0	4	4
0	0	0	0	0	0	0	0	0
10	9	30	39	40	20	16	32	44
0	0	0	0	0	0	0	0	0
4	0	5	7	12	4	8	20	4
0	1	0	1	0	4	0	0	0
0	0	0	0	0	0	0	0	0
1	1	0	0	0	0	0	0	0
0	0	0	0	0	0	0	0	0
0	0	0	0	0	0	0	0	0
2	0	6	0	4	8	0	4	8
0	0	0	0	0	0	0	0	0
0	0	0	0	0	0	0	0	0
0	0	0	0	0	0	0	0	0
0	0	0	0	0	0	0	0	0
5	0	6	6	28	8	8	20	8
1	0	3	2	8	4	0	4	8
0	0	0	0	0	0	0	0	0
2	0	2	1	8	0	4	4	4
0	0	0	0	0	0	0	0	0
2	1	5	4	4	8	4	8	8
0	0	0	0	0	0	0	0	0
0	2	3	5	12	8	8	4	12
0	0	0	0	0	0	0	0	0
0	0	0	0	0	0	0	0	0
14	5	5	10	16	4	16	0	12
0	2	0	1	0	0	0	0	4
1	0	0	2	0	8	0	0	4
0	0	0	0	0	0	0	0	0
0	0	0	0	0	0	0	0	0
0	0	0	0	0	0	0	0	0
0	0	0	0	0	0	0	0	0
0	0	1	1	0	0	0	0	0
0	0	0	0	0	0	0	0	0
0	0	0	0	0	0	0	0	0
0	0	0	0	0	0	0	0	0
1	3	3	3	0	12	12	32	24
0	0	0	0	0	0	0	0	0
0	0	0	0	0	0	0	0	0
1	3	4	3	16	4	4	16	16
0	0	0	0	0	0	0	0	0
0	0	0	0	0	0	0	0	0
0	0	0	0	0	0	0	0	0
0	0	0	0	0	0	0	0	0
0	1	0	0	0	0	0	0	0
0	0	0	0	0	0	0	0	0
2	2	1	2	0	0	0	0	8
0	0	0	0	0	0	0	0	0
0	0	0	0	0	0	0	0	0
5	5	13	2	8	0	4	8	4
0	0	0	0	0	0	0	0	0
0	0	0	0	0	0	0	0	0
0	0	2	0	0	0	0	0	0
0	0	0	0	0	0	0	0	0
0	0	1	0	0	0	0	0	0
0	0	1	1	0	4	0	0	0
0	0	0	1	0	0	0	0	0
0	0	0	0	0	0	0	0	0
4	1	2	8	4	8	4	12	0
0	0	0	0	0	4	0	0	0
0	0	0	0	0	0	0	0	0
1	3	2	3	8	0	0	4	0
4	0	2	7	8	8	12	8	4
0	0	0	1	0	4	0	4	0
1	0	0	0	0	0	0	0	0
3	2	2	1	4	12	0	0	12

0	0	0	0	0	0	0	0	0
0	0	0	0	0	0	0	0	0
2	0	0	3	8	0	0	0	0
1	3	11	9	16	12	4	12	8
47	49	92	84	136	112	72	108	60
4	4	0	0	0	0	8	0	0
29	19	52	87	108	56	52	84	92
34	31	26	23	28	12	8	16	8
0	0	0	0	0	4	0	0	0
0	0	0	0	0	0	0	0	0
0	0	0	0	0	0	0	0	0
0	1	0	0	0	0	4	4	0
1	0	0	0	0	0	0	0	0
0	0	0	0	0	0	0	0	0
3	5	7	5	8	8	12	12	40
1	0	0	0	0	0	4	0	0
0	0	0	0	0	8	0	0	0
0	0	0	0	0	0	0	0	0
0	0	0	0	0	0	0	0	0
5	3	7	12	12	16	24	28	44
0	0	0	0	0	0	0	0	0
0	0	0	0	0	0	0	0	0
0	0	0	0	0	0	0	0	0
0	0	0	0	0	0	0	0	0
0	0	0	0	0	0	0	0	0
0	0	0	0	0	0	0	0	0
1	1	2	2	0	0	0	0	0
0	0	0	0	0	0	0	0	0
0	0	0	2	0	0	0	0	0
0	0	0	0	0	0	0	0	0
0	0	0	0	0	0	0	0	0
0	0	0	0	0	0	0	0	0
0	0	0	0	0	0	0	0	0
0	0	1	0	8	8	0	0	4
0	0	0	0	0	0	0	0	0
0	0	0	0	0	0	0	0	0
0	0	0	0	0	0	0	0	0
2	0	0	0	0	0	0	0	4
187	160	329	398	760	400	292	452	424
13	17	24	23	40	36	36	40	84
0	0	0	0	0	0	0	0	0
0	0	0	0	0	0	0	0	0
2	1	2	0	0	0	0	0	0
0	0	0	0	0	0	0	0	0
0	0	0	0	0	0	0	0	0
0	0	0	0	0	0	0	0	0
0	0	0	0	0	0	0	0	0
0	0	0	0	0	0	0	0	0
1	2	2	3	4	4	0	4	12
0	0	1	0	0	0	0	4	4
6	7	4	11	4	4	16	0	0
0	0	0	0	0	0	0	0	0
0	0	0	0	0	0	0	0	0
1	0	2	0	0	0	0	0	0
0	0	0	0	0	0	0	0	0
81	55	113	124	180	112	68	96	140
0	0	0	0	0	0	0	0	0
0	0	0	0	0	0	0	0	0
0	1	0	0	0	0	0	0	0
0	0	0	0	0	0	0	0	0
2	1	3	4	4	4	4	4	4
9	7	8	7	12	16	4	16	8
0	0	0	0	0	0	0	0	0
0	0	0	0	0	0	0	0	0
0	0	0	0	0	0	0	0	0
0	0	0	0	0	0	0	0	0
0	0	0	0	0	0	0	0	0

0	0	0	0	0	0	0	0	0
0	0	0	0	0	0	0	0	0
0	0	0	0	0	0	0	0	0
0	0	0	0	0	0	0	4	0
0	0	0	1	0	0	0	0	0
2	5	9	7	0	4	0	4	4
0	0	0	0	4	0	0	0	0
0	0	0	0	0	0	0	0	0
0	0	0	0	0	0	0	0	0
0	0	0	0	0	0	0	0	0
1	0	0	0	0	0	0	0	4
0	0	1	1	4	0	0	4	0
0	0	0	0	0	0	0	0	0
0	0	0	0	0	0	0	0	0
0	0	0	0	0	4	0	0	0
0	0	0	0	0	0	0	0	0
0	0	0	0	0	0	0	0	0
2	2	2	4	8	0	0	0	0
1	1	1	0	0	0	0	0	0
0	0	0	0	0	0	0	0	0
0	0	0	0	0	0	0	0	0
5	5	9	4	8	0	0	4	0
5	2	7	5	4	0	0	0	0
0	0	0	3	8	0	0	0	0
0	0	0	0	0	0	0	0	0
0	0	0	0	0	0	0	0	0
0	0	0	1	0	0	0	0	0
0	0	0	0	0	0	0	0	0
0	0	2	0	4	8	4	0	0
151	115	235	317	404	232	164	224	216
10	6	19	22	36	24	4	8	16
36	33	81	97	176	88	52	52	64
1	3	10	9	8	4	12	4	4
0	5	9	7	20	4	20	4	12
15	17	42	48	88	44	24	60	32
0	0	0	0	0	0	0	0	0
0	0	0	0	0	0	0	0	4
0	0	0	0	0	0	0	0	0
0	0	0	0	0	0	0	0	0
0	0	0	0	0	0	0	0	0
0	0	0	0	0	0	0	0	0
0	0	0	0	0	0	0	0	0
0	0	0	1	0	0	0	0	0
0	0	0	0	0	0	0	0	0
0	0	0	0	0	0	0	0	0
0	0	0	0	0	0	0	0	0
2	3	8	10	24	12	0	20	8
0	1	0	0	0	0	0	0	0
0	0	0	2	0	0	0	0	0
4	3	13	11	12	16	12	20	12
1	1	4	5	0	0	4	4	4
1	0	1	2	0	0	0	0	4
0	0	0	0	0	0	0	0	0
0	0	0	0	0	0	0	0	0
1	0	1	0	4	0	0	0	0
0	0	1	0	4	0	0	4	4
0	0	0	0	0	0	0	0	0
0	0	0	0	0	0	0	0	0
0	0	2	0	0	0	0	0	0
1	0	1	0	0	0	0	0	0
0	0	0	0	0	0	0	0	0
0	1	0	0	0	0	0	0	0
0	0	0	0	0	0	0	0	0
0	0	2	0	0	0	0	0	0
0	2	1	0	4	0	0	0	0
1	0	0	2	8	0	0	0	0
1	0	1	1	0	0	0	0	4

0	0	0	0	0	0	0	0	0
4	3	10	10	16	4	4	8	0
9	9	16	24	48	28	16	24	28
0	0	0	0	0	0	0	0	0
0	0	1	0	0	0	0	0	0
0	0	1	0	0	0	0	0	0
7	4	4	6	16	0	0	8	12
6	9	20	33	96	16	20	40	12
0	0	3	4	4	8	4	4	0
17	26	45	62	116	68	36	56	52
0	0	0	0	0	0	0	4	0
0	0	0	0	4	0	0	0	0
0	0	0	0	0	0	0	0	0
0	0	0	0	4	0	0	0	0
0	0	0	0	0	0	0	0	0
0	0	0	0	0	0	0	0	0
0	0	0	0	0	0	0	0	0
0	0	0	0	0	0	0	0	0
0	0	0	0	0	0	0	0	0
0	0	0	0	0	0	0	0	0
0	0	0	0	0	0	0	0	0
0	0	0	0	0	0	4	0	0
0	0	1	0	0	0	0	0	0
2	0	3	0	0	4	0	4	0
6	8	13	17	36	8	16	0	12
0	0	0	0	0	0	0	0	0
0	0	0	2	0	0	4	4	0
0	0	0	0	0	0	0	0	0
0	0	0	0	0	0	0	0	0
0	0	0	0	0	0	0	0	0
0	0	0	0	0	0	0	0	0
0	0	0	0	0	0	0	0	0
0	0	0	0	0	0	0	0	0
0	0	0	0	8	0	0	0	0
0	0	0	0	0	0	0	0	0
0	0	0	0	0	0	0	0	0
17	9	27	30	40	20	16	32	20
3	1	2	4	4	0	8	12	4
0	0	0	0	0	0	0	0	0
0	0	0	0	0	0	0	0	0
0	0	0	0	0	0	0	0	0
3	1	2	16	16	16	4	8	16
0	0	0	0	0	0	0	0	0
0	0	0	0	0	0	0	0	0
0	0	0	0	0	0	0	0	0
0	0	0	0	0	0	0	0	0
0	0	0	0	0	0	0	0	0
0	0	0	0	4	4	0	0	0
1	0	0	0	0	0	0	0	0
0	0	0	0	0	0	0	0	0
0	1	7	4	4	0	4	4	0
0	0	0	0	0	0	0	0	0
0	1	5	4	4	4	0	0	4
33	31	79	85	148	80	72	76	156
0	0	0	0	0	0	0	0	0
0	0	0	0	0	0	0	0	0
0	0	0	0	0	0	0	0	0
0	0	0	0	0	0	0	0	0
1	0	1	0	0	4	0	0	0
5	4	4	10	12	4	0	8	4
1	0	0	1	0	0	0	0	0
0	0	0	1	0	0	4	0	0
7	6	20	16	28	8	20	8	32
0	0	0	0	0	0	0	0	0
3	4	10	17	12	12	12	20	12
4	0	3	2	0	12	0	16	8
0	2	0	1	0	0	0	0	0

0	0	0	0	0	0	0	0	0
2	0	0	0	0	0	0	0	0
0	2	0	0	0	0	0	0	0
1	3	8	6	16	8	4	0	4
0	1	0	0	0	0	4	4	0
0	0	0	0	0	0	0	0	0
0	0	0	0	0	0	0	0	0
0	1	0	0	0	4	4	0	0
0	0	1	1	4	0	0	4	0
0	0	0	0	0	0	4	0	0
0	0	0	0	0	0	0	0	0
0	0	0	0	0	0	0	0	0
0	0	0	0	0	0	0	0	0
0	0	0	0	0	0	0	0	0
0	2	6	2	0	0	0	0	4
0	0	0	0	0	0	0	0	4
0	0	0	0	0	0	0	0	0
0	1	0	0	0	0	0	0	0
3	1	3	6	16	12	12	12	0
0	0	3	0	0	0	0	0	0
0	0	0	1	4	0	0	0	0
0	0	0	0	0	0	0	0	0
3	1	5	3	8	16	0	16	8
0	1	0	1	0	0	0	0	0
0	0	0	0	0	0	0	0	0
0	0	0	0	0	0	0	0	0
0	0	0	0	0	0	0	0	0
0	0	0	1	0	0	0	0	0
0	0	0	0	0	0	0	0	0
0	0	0	0	0	4	0	0	0
0	0	0	0	0	0	0	0	0
0	0	0	0	0	0	0	0	0
0	0	0	0	0	0	0	0	0
0	0	0	0	0	0	0	0	4
0	0	0	0	0	0	0	0	0
0	0	0	0	0	0	0	0	0
0	0	1	0	4	0	0	0	0
0	0	0	0	0	0	0	0	0
0	0	0	0	0	0	0	0	0
0	0	1	0	0	0	0	0	0
0	0	1	0	0	0	0	0	0
0	0	0	0	0	0	0	0	0
0	1	3	1	4	8	4	0	8
0	0	0	0	0	0	0	0	0
0	0	0	0	0	0	0	0	0
0	0	2	6	8	4	4	0	8
0	0	1	3	4	0	0	0	0
0	0	0	1	0	0	0	0	8
0	0	0	0	0	0	0	0	0
0	0	1	1	0	0	0	0	0
0	0	0	0	0	0	0	0	0
0	0	0	0	0	0	0	0	0
0	0	0	0	0	0	0	0	0
1	0	0	1	8	0	0	0	0
0	0	0	0	0	4	0	0	0
0	0	2	0	0	0	0	0	0
0	0	0	0	0	0	0	0	0
0	0	0	0	0	0	0	0	0
0	1	0	0	0	0	0	0	0
0	0	0	0	0	0	0	0	0
0	0	0	0	0	0	0	0	0
0	0	0	0	0	0	0	0	0
0	0	0	0	0	0	0	0	0
0	0	0	0	0	4	0	0	0
0	0	0	0	0	0	0	0	0
0	0	0	0	0	0	0	0	0
0	0	0	0	0	0	0	0	0

0	0	0	0	0	0	0	0	0
0	0	0	0	0	0	0	0	0
0	0	0	0	0	0	0	0	0
0	0	0	0	0	0	0	0	0
0	0	0	0	0	0	0	0	0
0	0	0	0	0	0	0	0	0
0	0	0	0	0	0	0	0	0
0	0	0	0	0	0	0	0	0
0	0	0	0	0	0	0	0	0
0	0	0	0	0	0	0	0	0
0	0	0	0	0	0	0	0	0
0	0	0	0	0	0	0	0	0
0	0	0	0	0	0	0	0	0
0	0	0	0	0	0	0	0	0
0	0	0	0	0	0	0	0	0
0	0	0	0	0	0	0	0	0
0	0	0	0	0	0	0	0	0
0	0	0	0	0	0	0	0	0
0	0	0	0	0	0	0	0	0
0	0	0	0	0	0	0	0	0
0	0	0	0	0	0	0	0	0
0	0	0	0	0	0	0	0	0
0	0	0	0	0	0	0	0	0
0	0	0	0	0	0	0	0	0
0	0	0	0	0	0	0	0	0
0	0	11	19	24	56	24	44	48
0	0	0	0	0	0	0	0	0
0	0	0	0	0	0	0	0	0
0	0	0	0	0	0	0	0	0
0	0	0	0	0	0	0	0	0
4	4	7	5	8	12	8	12	16
0	1	0	2	0	0	0	4	0
0	0	0	0	0	0	0	0	0
0	0	0	0	0	0	0	0	0
0	0	0	0	0	0	0	0	0
1	0	1	1	4	4	0	0	0
0	0	0	0	0	0	0	0	0
0	0	0	0	0	0	0	0	0
2	3	4	7	12	8	4	12	8

Pir2_M53_20_2	Pir2_M53_25_3	Pir2_M53_30_3	Pir2_M53_35_4	Pir2_M53_40_4	Pir2_M53_45_5	Pir2_M53_50_5	Pir2_M53_55_6	Pir2_M53_60_6
16	8	8	4	4	2	3	1	2
0	0	0	0	0	0	0	0	0
40	52	28	16	12	11	8	6	9
0	0	0	0	0	0	0	0	0
8	20	0	6	2	1	1	1	1
0	0	0	0	0	0	0	0	0
0	0	0	0	0	0	0	0	0
0	0	0	0	0	0	0	0	0
0	0	0	0	0	0	0	0	0
0	0	0	0	0	0	0	0	0
0	24	4	8	2	3	2	1	2
0	0	0	0	0	0	0	0	0
0	0	0	0	0	0	0	0	0
0	0	0	0	0	0	0	0	0
0	0	0	0	0	0	0	0	0
36	36	24	14	10	11	10	7	7
24	40	24	12	10	15	6	8	10
0	0	0	0	0	0	0	0	0
4	28	4	6	8	9	3	2	2
0	0	0	0	0	0	0	0	0
20	8	4	6	2	4	1	0	1
0	0	0	0	0	0	0	0	0
20	16	24	24	10	16	6	7	9
0	0	0	0	0	0	0	0	0
0	0	0	0	0	0	0	0	0
24	36	28	26	6	13	4	4	11
0	4	4	2	2	1	0	1	0
4	28	4	4	14	2	1	1	4
0	0	0	0	0	0	0	0	0
0	0	0	0	0	0	0	0	0
0	0	0	0	0	0	0	0	0
0	0	0	0	0	0	0	0	0
4	12	0	2	2	0	3	0	1
0	0	0	0	0	0	0	0	0
0	0	0	0	0	0	0	0	0
0	0	0	0	0	0	0	0	0
56	92	80	40	18	27	19	19	19
0	0	0	0	0	0	0	0	0
0	0	0	0	0	0	0	0	0
16	48	68	38	24	38	20	19	20
0	0	0	0	0	0	0	0	0
0	0	0	0	0	0	1	0	0
0	0	0	0	0	0	0	1	1
0	0	0	2	0	0	0	0	0
0	4	8	2	0	0	1	0	0
0	0	0	0	0	0	0	0	0
12	28	16	4	4	3	2	1	3
0	0	0	0	0	0	0	0	0
0	4	0	0	0	0	1	0	0
24	36	16	18	12	8	5	8	13
0	0	0	0	0	0	0	0	2
0	0	0	0	0	0	0	0	0
0	0	0	0	0	0	0	1	0
0	0	0	0	0	0	0	0	0
0	0	4	2	0	0	1	0	0
8	12	4	4	2	1	1	1	1
0	0	0	0	0	0	0	0	0
0	0	0	0	0	0	0	0	0
8	52	12	6	4	5	6	8	6
0	0	0	0	0	0	0	1	0
0	0	0	0	0	0	0	0	0
0	0	0	0	0	0	0	0	0
24	20	4	14	4	5	5	0	0
0	0	0	0	0	2	0	0	0
0	0	0	0	0	0	0	0	0
12	24	8	12	12	7	10	7	9

0	0	0	0	0	0	0	0	0
0	0	0	0	0	0	0	0	0
0	0	0	0	4	2	0	0	2
12	24	12	16	10	8	6	4	5
160	176	60	64	50	33	23	17	22
0	0	0	0	0	0	0	0	0
124	160	68	74	36	46	32	22	27
52	28	12	16	2	0	7	0	5
0	4	4	4	0	1	0	0	0
0	0	0	0	0	0	0	0	0
0	0	0	0	0	0	0	0	0
0	0	0	0	0	0	0	0	0
0	0	4	2	2	2	3	1	5
0	0	0	0	0	0	0	0	0
52	72	52	46	44	31	21	19	25
0	0	0	4	4	0	2	7	7
0	0	0	0	0	0	1	0	0
0	0	0	0	0	1	0	0	0
0	0	0	0	0	0	0	0	0
96	152	108	72	58	53	30	20	30
0	0	0	0	0	0	0	0	0
0	0	0	0	0	0	0	0	0
0	0	0	0	0	0	0	0	0
0	0	0	0	0	0	0	0	0
0	0	0	0	0	0	0	0	0
0	0	0	0	0	0	0	0	0
24	8	16	12	4	5	1	4	8
8	8	0	2	0	2	0	1	0
0	0	0	0	0	2	0	0	0
0	0	0	0	0	0	0	0	0
0	0	0	0	0	0	0	0	0
0	0	0	0	0	0	0	0	0
0	0	0	0	0	0	0	0	0
4	8	4	6	2	2	1	2	1
0	0	0	0	0	0	0	0	0
0	0	0	0	0	0	0	0	0
0	0	0	0	0	0	1	0	0
4	4	8	0	6	0	0	0	1
648	768	304	316	164	197	132	98	122
104	160	112	68	48	46	24	28	26
0	0	0	0	0	0	0	0	0
0	0	0	0	0	0	0	0	0
0	0	0	4	2	2	1	0	0
0	0	0	0	0	0	0	0	0
0	0	0	0	0	0	0	0	0
0	0	0	0	0	0	0	0	0
0	0	0	0	0	0	0	0	0
0	0	0	0	2	3	2	0	1
4	4	0	2	2	0	0	0	3
0	4	4	0	0	1	0	0	0
4	8	4	2	4	2	2	0	1
0	0	0	0	0	0	0	0	0
0	0	0	0	0	0	0	0	0
0	0	4	2	2	3	0	0	0
0	0	0	0	0	0	0	0	0
192	376	168	170	110	107	71	89	96
0	0	0	0	0	0	0	0	0
0	0	0	0	0	0	0	0	0
0	0	0	0	2	2	0	0	1
0	0	0	0	0	0	0	0	0
8	4	0	0	2	3	1	1	1
20	16	4	14	8	9	6	6	5
0	0	0	0	0	0	0	0	0
0	0	0	0	0	0	0	0	0
0	0	0	2	0	0	0	0	0
0	0	0	0	0	0	0	0	0
0	0	4	0	0	1	1	0	0

0	0	0	0	0	0	0	0	0
0	0	0	0	0	0	0	0	0
0	0	0	0	0	0	0	0	0
0	0	0	0	0	0	0	0	0
0	0	0	0	0	0	0	0	0
12	8	8	8	8	3	5	2	2
0	0	0	0	0	0	0	0	0
0	0	0	0	0	0	0	0	0
0	0	0	0	0	0	0	0	0
0	0	0	0	0	0	0	0	0
4	4	4	0	2	1	1	1	0
0	0	0	0	0	0	1	0	1
0	0	0	0	0	0	0	0	0
0	0	0	0	0	0	0	0	0
4	4	0	0	0	1	0	2	0
0	0	0	0	0	0	0	0	0
0	0	4	0	0	0	0	0	0
0	0	0	0	0	0	0	0	0
0	0	0	0	0	0	0	1	0
0	0	0	0	0	0	0	0	0
0	0	0	0	0	0	0	0	0
0	0	0	0	0	0	1	1	0
4	0	0	0	2	0	0	1	1
0	0	4	0	2	0	1	0	0
0	0	0	0	0	0	0	0	0
0	0	0	0	0	0	0	0	0
0	4	0	0	0	0	0	0	0
0	0	0	0	0	1	0	0	0
0	0	0	6	0	0	0	0	0
388	336	140	126	76	77	45	45	15
60	72	20	14	6	9	1	6	7
552	528	132	104	38	70	34	39	29
36	20	4	6	4	4	7	2	4
16	16	8	6	2	5	5	4	2
68	124	28	28	10	17	7	9	9
0	0	0	0	0	0	0	0	0
8	4	0	0	0	0	1	0	1
0	0	0	0	0	0	0	0	0
0	0	0	0	0	0	0	0	0
0	0	0	0	0	0	0	0	0
0	0	0	2	0	0	0	0	0
0	0	0	0	0	0	0	0	0
0	0	0	2	0	1	1	0	1
0	0	0	0	0	0	0	0	0
0	0	0	0	0	0	0	0	0
0	0	0	0	0	0	0	0	0
8	12	8	6	6	6	1	1	7
0	0	0	0	0	0	0	0	0
4	4	0	4	0	1	0	1	0
24	32	8	12	6	6	6	5	4
0	0	4	0	6	3	0	0	2
4	4	0	0	2	0	0	0	0
0	0	0	0	0	0	0	0	0
0	0	0	0	0	0	0	0	0
0	4	0	0	0	1	1	0	0
4	0	8	4	2	1	4	2	1
0	0	0	0	0	0	0	0	0
0	0	0	0	0	0	0	0	0
0	0	0	0	0	0	0	0	0
0	0	0	4	0	1	1	0	0
0	0	0	0	0	0	0	0	0
0	0	0	0	0	0	0	0	0
0	0	0	0	0	0	0	0	0
0	0	0	0	0	0	0	0	0
0	0	0	2	0	0	0	0	0
0	0	0	0	0	0	0	0	0
4	0	4	0	0	0	1	0	0

0	0	0	0	0	0	0	0	0
24	12	12	14	12	6	8	6	3
60	56	40	36	44	21	12	7	17
0	0	0	0	0	0	0	0	0
0	0	0	0	0	1	0	0	0
4	0	0	0	0	0	0	0	0
36	24	24	10	8	9	5	4	7
52	132	12	42	16	9	4	9	25
8	36	8	2	8	4	3	4	4
144	184	108	54	22	40	25	23	20
0	0	8	2	0	0	2	0	4
0	4	0	0	2	0	0	0	0
0	0	0	0	0	0	0	0	0
0	0	0	0	0	0	0	0	1
0	0	0	0	0	0	0	0	0
0	0	0	0	0	0	0	0	0
0	0	0	0	0	0	0	0	0
0	0	0	0	0	0	0	0	0
0	0	0	0	0	0	0	0	0
0	0	0	0	0	0	0	0	0
0	0	0	0	0	0	0	0	0
0	8	0	0	0	0	0	0	1
0	0	0	2	0	0	0	0	0
8	0	4	0	4	0	0	0	1
44	28	12	16	14	15	6	5	2
0	0	0	0	0	0	0	0	0
4	8	4	4	6	3	2	4	1
0	0	0	0	0	0	0	0	0
0	4	4	0	2	1	0	1	0
0	0	0	0	0	0	0	0	0
4	4	0	0	0	1	0	0	1
0	0	0	0	0	0	0	0	0
0	0	0	0	0	0	0	0	0
0	0	0	0	0	0	0	0	0
0	0	0	0	0	0	0	0	0
0	0	0	0	0	0	0	0	0
52	80	48	28	18	17	12	10	2
12	20	4	6	14	1	2	1	3
0	0	0	0	0	0	0	0	0
0	0	0	0	0	0	0	0	0
0	0	0	0	0	0	0	0	0
36	52	8	6	4	9	7	5	4
4	0	0	0	0	0	1	2	0
0	0	0	0	0	0	0	0	1
0	0	0	0	0	0	0	0	0
0	0	0	0	0	0	1	0	0
0	0	0	0	0	0	0	0	0
0	0	0	0	0	0	0	0	0
0	0	0	0	0	0	0	0	0
0	0	0	0	0	0	0	0	0
0	8	0	0	0	0	0	0	0
0	0	0	0	0	0	0	0	0
4	8	8	2	0	1	1	0	3
316	464	280	186	152	120	88	84	69
0	0	0	0	0	0	0	0	0
4	0	0	0	0	0	0	0	0
0	0	0	0	0	0	0	0	0
0	0	0	0	0	0	0	0	0
0	0	0	0	0	1	0	0	1
24	32	8	12	6	9	3	6	9
0	4	0	0	0	0	0	0	0
0	0	0	0	0	0	0	0	0
32	56	12	22	10	17	9	5	2
0	0	0	0	0	0	1	0	0
28	12	4	6	4	5	4	1	4
8	4	0	0	2	2	1	3	2
0	0	0	2	0	0	1	0	0

0	0	0	0	0	0	0	0	0
4	0	0	0	0	0	0	0	1
0	0	0	0	0	0	0	0	0
20	20	12	8	4	3	1	1	1
8	4	0	0	0	0	2	0	0
0	0	0	0	0	0	0	0	0
0	0	0	0	0	0	0	0	0
0	0	0	0	0	0	0	0	0
0	0	0	0	0	0	1	0	0
12	4	0	0	2	0	0	0	0
0	0	0	0	0	0	0	0	0
0	0	0	0	0	0	0	0	0
0	0	0	0	0	0	0	0	0
0	0	0	0	0	0	0	0	0
4	20	4	4	2	1	0	1	0
4	4	0	0	0	0	0	0	0
0	0	0	0	0	0	0	0	0
0	0	0	0	0	0	0	0	0
16	24	0	4	2	2	0	2	2
0	0	0	0	0	0	0	0	0
0	0	0	0	0	0	0	0	0
0	0	0	0	0	0	0	0	0
24	24	4	4	2	4	0	4	5
0	0	0	0	0	0	0	1	0
0	0	0	0	0	0	0	0	0
0	0	0	0	0	0	0	0	0
0	0	0	0	0	0	0	0	0
0	0	0	0	0	0	0	1	0
0	0	0	0	0	0	0	0	0
0	0	0	0	0	0	0	0	0
0	0	0	0	0	0	0	0	0
0	0	0	0	0	0	0	0	0
0	0	0	0	0	0	0	0	0
0	0	0	0	0	0	0	0	0
0	0	0	0	0	0	0	0	0
0	0	0	0	0	0	0	0	0
16	8	0	2	0	0	0	0	1
0	0	0	0	0	0	0	0	0
0	0	0	0	0	0	0	0	0
0	0	0	0	0	0	0	0	0
0	0	0	0	0	0	0	0	0
0	0	0	0	0	0	0	0	0
8	4	12	4	0	7	3	5	2
0	0	0	0	0	0	0	0	0
0	0	0	2	2	1	1	0	0
4	20	4	6	4	4	1	5	5
4	4	4	0	2	0	0	0	0
0	0	4	2	2	0	2	0	0
0	0	0	0	0	0	0	0	0
0	0	0	0	0	1	0	0	0
0	0	0	0	0	4	3	0	1
0	0	0	0	0	0	0	0	0
4	0	0	0	0	0	0	0	0
0	8	4	0	0	0	0	1	0
4	4	0	0	0	2	1	1	0
8	4	0	2	4	2	1	2	2
0	8	0	0	0	0	0	0	0
0	0	0	0	0	0	0	0	0
0	0	0	0	0	0	0	0	0
0	0	0	0	0	0	0	0	1
0	0	0	0	0	0	0	0	0
0	0	0	0	0	0	0	0	0
0	0	0	0	0	0	0	0	0
0	4	0	0	0	0	0	0	0
0	0	0	0	0	0	0	0	0
0	0	0	0	0	0	0	0	0
0	0	0	0	0	0	0	0	0

0	0	0	0	0	0	0	0	0
0	0	0	0	0	0	0	0	0
0	0	0	0	0	0	0	0	0
0	0	0	0	0	0	0	0	0
0	0	0	0	0	0	0	0	0
0	0	0	0	0	0	0	0	0
0	0	0	0	0	0	0	0	0
0	0	0	0	0	0	0	0	0
0	0	0	0	0	0	0	0	0
0	0	0	0	0	0	0	0	0
0	0	0	0	0	0	0	0	0
0	0	0	0	0	0	0	0	0
0	0	0	0	0	0	0	0	0
0	0	0	0	0	0	0	0	0
0	0	0	0	0	0	0	0	0
0	0	0	0	0	0	0	0	0
0	4	0	0	0	0	0	0	0
0	0	0	0	0	0	0	0	0
0	0	0	0	0	0	0	0	0
0	0	0	0	0	0	0	0	0
0	0	0	0	0	0	0	0	0
0	0	0	0	0	0	0	0	0
0	0	0	0	0	0	0	0	0
0	0	0	0	0	0	0	0	0
0	4	0	0	0	0	0	0	0
84	80	40	52	46	35	16	19	20
0	0	0	0	0	0	0	0	0
0	4	0	4	0	0	2	1	0
0	0	0	0	0	0	0	0	0
0	0	0	0	0	0	0	0	0
12	12	8	6	6	2	2	2	2
0	0	0	0	0	1	0	0	1
0	0	0	0	0	0	0	0	0
0	0	0	0	0	0	0	0	0
0	0	0	0	0	0	0	0	0
0	4	4	0	0	0	0	0	0
0	0	0	0	0	0	0	0	0
0	4	0	2	0	0	0	0	1
8	16	0	4	4	2	0	1	1

Pir2_M53_65_7	Pir2_M53_70_7	Pir2_M53_75_8	Pir2_M53_80_8	Pir2_M53_85_9	Pir2_M53_90_9	Pir2_M53_95_1	Pir2_M53_100_	Pir2_M53_105_
2	1	1	0	0	0	0	0	0
0	0	0	0	0	0	0	0	0
5	4	5	5	7	3	3	2	2
0	0	0	0	0	0	0	0	0
1	0	0	1	1	0	0	0	1
0	0	0	0	0	0	0	0	0
0	0	0	0	0	0	0	0	0
0	0	0	0	0	0	0	0	0
0	0	0	0	0	0	0	0	0
0	0	0	0	0	0	0	0	0
0	0	0	1	0	0	1	1	0
0	0	0	0	0	0	0	0	0
0	0	0	0	0	0	0	0	0
0	0	0	0	0	0	0	0	0
0	0	0	0	0	0	0	0	0
5	2	1	4	3	3	6	4	6
10	9	10	4	2	5	5	12	11
0	0	0	0	0	0	0	0	0
0	0	6	2	1	0	0	1	0
0	0	0	0	0	0	0	0	0
1	1	0	1	0	0	0	0	0
0	0	0	0	0	0	0	0	0
3	4	8	21	6	4	3	16	19
0	0	0	0	0	0	0	0	0
0	0	0	0	0	0	0	0	0
1	1	4	3	3	3	0	0	1
2	0	0	0	0	0	0	0	0
1	1	0	1	2	0	0	0	0
0	0	0	0	0	0	0	0	0
0	0	0	0	0	0	0	0	0
0	0	0	0	0	0	0	0	0
0	0	0	0	0	0	0	0	0
1	0	1	1	0	1	0	1	1
0	0	0	0	0	0	0	0	0
0	0	0	0	0	0	0	0	0
0	0	0	0	0	0	0	0	0
4	6	8	13	14	6	7	2	2
0	0	0	0	0	0	0	0	0
0	0	0	0	0	0	0	0	0
17	8	8	11	9	12	5	12	19
0	0	0	0	0	0	0	0	0
0	0	0	0	0	0	0	0	0
0	0	0	0	1	0	0	0	0
0	0	0	0	0	0	0	0	0
0	0	0	0	0	1	0	0	0
0	0	0	0	0	0	0	0	0
0	1	2	2	0	0	1	1	0
0	0	0	0	0	0	0	0	0
1	0	0	0	0	0	0	0	0
9	3	10	8	8	2	6	13	28
0	0	0	1	0	0	0	0	0
0	0	0	1	0	1	0	0	0
0	0	0	0	0	0	0	0	0
0	0	0	0	0	0	0	0	0
1	0	1	1	0	1	1	1	1
1	1	1	0	1	0	0	0	0
0	0	0	0	0	0	0	0	0
0	0	0	0	0	0	0	0	0
4	1	1	4	3	2	2	1	2
0	0	0	0	0	0	0	0	0
0	0	0	0	0	0	0	0	0
0	0	0	0	0	0	0	0	0
0	0	1	0	3	0	1	0	1
0	0	0	0	0	0	0	0	0
0	0	0	0	0	0	0	0	0
14	8	16	8	4	2	3	4	4

0	0	0	0	0	0	0	0	0
0	0	0	0	0	0	0	0	0
0	0	1	0	0	1	0	0	8
2	1	1	2	4	4	2	3	9
5	10	8	11	7	11	5	5	3
0	0	0	0	0	0	0	0	0
18	6	9	11	9	13	4	4	7
2	3	3	0	1	1	1	0	0
0	2	0	0	2	2	0	0	0
0	0	0	0	0	0	0	0	0
0	0	0	0	0	0	0	0	0
0	0	0	0	0	0	0	0	0
4	17	5	1	1	0	0	1	0
0	0	0	0	0	0	0	0	0
9	12	19	34	18	14	24	24	25
4	4	5	12	2	1	8	7	12
0	0	0	0	0	0	0	0	0
0	0	0	0	0	0	0	0	0
0	0	0	0	0	0	0	0	0
15	9	9	11	9	10	6	19	19
0	0	0	0	0	0	0	0	0
0	0	0	0	0	0	0	0	0
0	0	0	0	0	0	0	0	0
0	0	0	0	0	0	0	0	0
0	0	0	0	0	0	0	0	0
0	0	0	0	0	0	0	0	0
2	2	1	3	2	1	1	1	2
0	0	0	2	2	0	0	0	0
0	0	0	0	0	0	0	0	0
0	0	0	0	0	0	0	0	0
0	0	0	0	0	0	0	0	0
0	0	0	0	0	0	0	0	0
0	0	0	0	0	0	0	0	0
0	0	2	1	0	1	0	0	0
0	0	0	0	0	0	0	0	0
0	0	0	0	0	0	0	0	0
0	0	0	0	0	0	0	0	0
1	2	1	0	0	0	0	0	0
50	33	31	31	22	23	9	17	11
16	13	16	12	9	20	12	8	19
0	0	0	0	0	0	0	0	0
0	0	0	0	0	0	0	0	0
1	2	0	2	2	0	1	0	2
0	0	0	0	0	0	0	0	0
0	0	0	0	0	0	0	0	0
0	0	0	0	0	0	0	0	0
0	0	0	0	0	0	0	0	0
2	2	1	0	0	1	1	1	1
0	2	1	1	0	0	0	0	0
0	0	1	0	0	0	0	0	0
0	1	2	2	0	0	0	0	0
0	0	0	0	0	0	0	0	0
0	0	0	0	0	0	0	0	0
0	0	0	0	0	0	0	0	0
0	0	0	0	0	0	0	0	0
65	55	61	52	41	38	33	52	60
0	0	0	0	2	1	1	0	0
0	0	1	0	0	0	0	0	0
0	0	0	1	0	0	0	0	0
0	0	0	0	0	0	0	0	0
0	1	0	3	1	0	0	0	1
2	2	1	1	1	2	1	1	0
0	0	0	0	0	0	0	0	0
0	0	0	0	0	0	0	0	0
0	0	0	0	0	0	0	0	0
0	0	0	0	0	0	0	0	0
1	1	0	1	0	0	0	0	1

0	0	0	0	0	0	0	0	0
0	0	0	0	0	0	0	0	0
0	0	0	0	0	0	0	0	0
0	0	0	0	0	0	0	0	0
0	0	0	0	0	0	0	0	0
0	1	1	1	0	0	0	0	1
0	0	0	0	0	0	0	0	0
0	0	0	0	0	0	0	0	0
0	0	0	0	0	0	0	0	0
0	0	0	0	0	0	0	0	0
0	0	0	1	0	0	0	0	0
0	1	0	0	1	0	0	0	0
0	0	0	0	0	0	0	0	0
0	0	0	0	0	0	0	0	0
0	0	0	0	0	0	0	0	0
0	0	0	0	0	0	0	0	0
0	0	0	0	0	0	0	0	0
0	0	0	0	0	0	0	0	0
0	0	0	0	0	0	0	0	0
0	0	0	0	0	0	0	0	0
0	0	0	0	0	0	0	0	0
1	0	0	0	0	0	1	0	0
0	0	0	1	0	2	0	0	0
0	0	0	0	0	0	0	0	0
0	0	0	0	0	0	0	0	0
0	0	0	0	0	0	0	0	0
0	0	0	0	0	0	0	0	0
0	0	0	0	0	0	0	0	0
0	0	0	0	0	0	0	0	0
19	0	16	11	4	1	6	0	3
2	1	0	2	4	2	0	0	2
14	12	12	16	14	7	2	9	11
2	0	1	1	3	1	1	0	0
0	0	0	0	1	1	1	1	0
5	7	3	3	1	2	3	0	0
0	0	0	0	0	0	0	0	0
1	0	0	0	0	0	0	0	0
0	0	0	0	0	0	0	0	0
0	0	0	0	0	0	0	0	0
0	0	0	0	0	0	0	0	0
0	0	0	0	0	0	0	0	0
0	0	0	0	0	0	0	0	0
0	0	0	0	1	0	1	0	0
0	0	0	0	0	0	0	0	0
0	0	0	0	0	0	0	0	0
0	0	0	0	0	0	0	0	0
0	0	0	0	0	2	0	1	0
0	0	0	0	0	0	0	0	0
0	0	0	0	0	0	0	0	0
1	3	0	2	0	0	0	0	0
0	1	1	0	0	1	0	0	0
0	0	0	1	1	0	0	0	0
0	0	0	0	0	0	0	0	0
0	0	0	0	0	0	0	0	0
1	0	1	0	0	1	1	0	1
0	0	2	0	1	1	0	1	1
0	0	0	0	0	0	0	0	0
0	0	0	0	0	0	0	0	0
0	0	0	0	0	0	0	0	0
0	0	0	0	0	0	0	0	0
0	0	0	0	0	0	0	0	0
0	0	0	0	0	0	0	0	0
0	0	0	0	0	0	0	0	0
0	0	0	0	0	0	0	0	0
0	0	0	0	0	0	0	0	0
0	0	0	0	0	0	0	0	0
0	1	0	0	0	0	0	0	0

0	0	0	0	0	0	0	0	0
2	3	0	1	0	1	1	0	2
6	1	4	2	2	1	2	5	2
0	0	0	0	0	0	0	0	0
0	0	0	0	0	0	0	0	0
0	0	0	0	0	0	0	0	0
3	7	1	1	1	4	2	3	5
3	5	3	3	0	3	4	3	0
1	0	2	0	1	0	1	0	0
5	9	8	7	5	5	8	8	10
0	1	2	2	2	1	1	1	2
0	0	0	0	1	0	0	0	0
0	0	0	0	0	0	0	0	0
0	0	0	0	0	0	0	0	0
0	0	0	0	0	0	0	0	0
0	0	0	0	0	0	0	0	0
0	0	0	0	0	0	0	0	0
0	0	0	0	0	0	0	0	0
0	0	0	0	0	0	0	0	0
0	0	0	0	0	0	0	0	0
0	0	0	0	0	0	0	0	0
0	0	0	0	0	0	0	0	0
0	0	0	0	0	0	0	0	0
0	0	0	0	1	1	0	0	0
8	2	2	0	5	0	2	1	3
0	0	0	0	0	0	0	0	0
1	1	1	1	1	0	0	0	0
0	0	0	0	0	0	0	0	0
0	1	0	0	1	3	1	1	2
0	0	0	0	0	0	0	0	0
0	1	0	0	0	0	0	0	0
0	0	0	0	0	0	0	0	0
0	0	0	0	0	0	0	0	0
0	0	0	0	0	0	0	0	0
0	0	0	0	0	0	0	0	0
0	0	0	0	0	0	0	0	0
7	3	3	0	3	2	1	2	0
1	2	0	1	2	1	1	1	3
0	0	0	0	0	0	0	0	0
0	0	0	0	0	0	0	0	0
0	0	0	0	0	0	0	0	0
3	1	1	1	1	3	2	1	3
0	0	0	0	0	1	0	1	1
0	0	0	0	0	0	0	0	0
0	0	0	0	0	0	0	0	0
0	0	0	0	0	0	0	0	0
0	0	0	0	0	0	0	0	0
0	0	0	0	0	0	0	0	0
0	0	0	0	0	0	0	0	0
0	0	0	0	0	0	0	0	0
0	1	0	0	0	0	0	0	0
0	0	0	0	0	0	0	0	0
0	0	0	1	0	0	0	1	0
33	22	29	32	39	25	24	16	14
0	0	0	0	0	0	0	0	0
0	0	0	0	0	0	0	3	0
0	0	0	0	0	0	0	1	0
0	0	0	0	0	0	0	0	0
0	0	0	0	0	0	0	0	0
4	0	0	0	1	1	0	0	3
0	0	0	0	0	0	0	0	0
0	0	0	0	0	0	0	0	0
9	1	2	7	1	1	3	3	5
0	0	0	0	0	0	0	0	0
1	0	1	0	0	0	0	1	1
0	0	0	0	0	0	0	0	0
0	0	0	0	0	0	0	0	0

0	0	0	0	0	0	0	0	0
0	0	0	0	0	0	0	0	0
0	0	0	0	0	0	0	0	0
0	1	0	0	2	0	0	0	0
0	0	0	0	0	0	0	0	0
0	0	0	0	0	0	0	0	0
0	0	0	0	0	0	0	0	0
0	0	0	0	0	0	0	0	0
0	0	0	0	0	0	0	0	0
0	0	0	2	0	0	0	1	0
0	0	0	0	0	0	0	0	0
0	0	0	0	0	0	0	0	0
0	0	0	0	0	0	0	0	0
0	0	0	0	0	0	0	0	0
1	0	1	2	0	0	2	1	3
0	0	0	0	0	0	0	0	0
0	0	0	0	0	0	0	0	0
0	0	0	1	0	0	0	0	0
1	1	1	0	0	0	0	1	0
0	0	0	1	0	1	0	1	0
0	0	0	0	0	0	0	0	0
0	0	0	0	0	0	0	0	0
1	2	2	1	0	0	0	0	0
0	0	0	0	1	0	1	1	0
0	0	0	0	0	0	0	0	0
0	0	0	0	0	0	0	0	0
0	0	0	0	0	0	0	0	0
0	0	0	0	0	0	0	0	0
0	0	0	0	0	0	0	0	0
0	0	0	0	0	0	0	0	0
0	0	0	0	0	0	0	0	0
0	0	0	0	0	0	0	0	0
0	0	0	0	0	0	0	0	0
0	0	0	0	0	0	0	0	0
0	0	0	0	0	0	0	0	0
0	0	0	0	0	0	0	0	0
1	0	0	0	0	0	0	0	0
0	0	0	0	0	0	0	0	0
0	0	0	0	0	0	0	0	0
0	0	0	0	0	0	0	0	0
0	0	0	0	0	0	0	0	0
0	0	0	0	0	0	0	0	0
1	1	0	0	1	3	0	1	1
0	0	0	0	0	0	0	0	0
0	1	0	0	0	0	0	0	0
2	3	2	1	2	6	1	4	2
0	0	1	0	0	1	0	0	0
0	0	0	1	0	0	0	0	1
0	0	0	0	0	0	0	0	0
0	0	0	0	0	0	0	0	0
3	0	3	2	3	0	1	0	2
0	0	0	0	0	0	0	0	0
0	0	1	0	1	0	0	0	0
0	0	0	0	0	0	0	1	0
0	1	0	0	0	1	2	1	0
0	0	0	0	0	1	0	0	1
0	0	0	0	0	0	0	0	0
0	0	0	0	0	0	0	0	0
0	0	0	0	0	0	0	0	0
0	0	0	0	0	0	0	0	0
0	0	0	0	0	0	0	0	0
0	0	0	0	0	0	0	0	0
0	0	0	0	0	0	0	0	0
0	0	0	0	0	0	0	0	0
0	0	0	0	0	0	0	0	0
0	0	0	0	0	0	0	0	0
0	0	0	0	0	0	0	0	0

0	0	0	0	0	0	0	0	0
0	0	0	1	0	0	0	0	0
0	0	0	0	0	0	0	0	0
0	0	0	0	0	0	0	0	0
0	0	0	0	0	0	0	0	0
0	0	0	0	0	0	0	0	0
0	0	0	0	0	0	0	0	0
0	0	0	0	0	0	0	0	0
0	0	0	0	0	0	0	0	0
0	0	0	0	0	0	0	0	0
0	0	0	0	0	0	0	0	0
0	0	0	0	0	0	0	0	0
0	0	0	0	0	0	0	0	0
0	0	0	0	0	0	0	0	0
0	0	0	0	0	0	0	0	0
0	0	0	0	0	0	0	0	0
0	0	0	0	0	0	0	0	0
0	0	0	0	0	0	0	0	0
0	0	0	0	0	0	0	0	0
0	0	0	0	0	0	0	0	0
0	0	0	0	0	0	0	0	0
0	0	0	0	0	0	0	0	0
0	0	0	0	0	0	0	0	0
0	0	0	0	0	0	0	0	0
0	0	0	0	0	0	0	0	0
11	10	10	10	7	13	3	6	9
0	0	0	0	0	0	0	0	0
0	0	0	1	0	0	0	0	0
0	0	0	0	0	0	0	0	0
0	0	0	0	0	0	0	0	0
2	1	2	0	2	2	0	1	1
0	0	0	0	0	0	0	1	0
0	0	0	0	0	0	0	0	0
0	0	0	0	0	0	0	0	0
0	0	0	0	0	0	0	0	0
0	0	0	0	0	0	0	0	0
0	0	0	0	0	0	0	0	0
1	0	0	0	1	0	0	0	0
1	1	1	1	1	1	0	0	0

Pir2_M53_110_	Pir2_M53_115_	Pir2_M53_120_	Pir2_M53_125_	Pir2_M53_130_	Pir2_M53_135_	Pir2_M53_140_	Pir2_M53_145_	Pir2_M53_150_
0	1	0	0	1	1	1	0	1
0	0	0	0	0	0	0	0	0
3	1	3	6	3	6	7	5	0
0	0	0	0	0	0	0	0	0
0	0	1	0	2	1	0	0	0
0	0	0	0	0	0	0	0	0
0	0	0	0	0	0	0	0	0
0	0	0	0	0	0	0	0	0
0	0	0	0	0	0	0	0	0
0	0	0	0	0	0	0	0	0
0	0	0	1	1	0	0	0	1
0	0	0	0	0	0	0	0	0
0	0	0	0	0	0	0	0	0
0	0	0	0	0	0	0	0	0
0	0	0	0	0	0	0	0	0
3	1	3	4	8	17	12	2	1
11	7	7	6	15	8	3	2	4
0	0	0	0	0	0	0	0	0
1	1	1	1	2	2	0	2	0
0	0	0	0	0	0	0	0	0
0	0	1	1	0	1	1	1	1
0	0	0	0	0	0	0	0	0
10	16	19	26	35	24	10	4	4
0	0	0	0	0	0	0	0	0
0	0	0	0	0	0	0	0	0
0	0	1	7	1	3	0	1	0
0	0	1	0	0	0	0	0	0
0	0	0	2	0	1	0	1	0
0	0	0	0	0	0	0	0	0
0	0	0	0	0	0	0	0	0
0	0	0	0	0	0	0	0	0
0	0	0	0	0	0	0	0	0
0	0	0	0	0	0	0	0	0
0	0	0	0	0	0	0	0	0
0	0	0	0	0	0	0	0	0
0	0	0	0	0	0	0	0	0
2	5	7	7	5	12	7	4	3
0	0	0	0	0	0	0	0	0
0	0	0	0	0	0	0	0	0
4	5	5	5	10	12	9	3	5
0	0	0	0	0	0	0	0	0
0	0	0	0	0	0	0	0	0
0	0	0	0	0	0	1	1	0
0	0	0	0	0	1	0	0	0
0	0	0	0	0	0	0	0	0
0	0	0	0	0	0	0	0	0
0	0	1	0	2	0	0	0	0
0	0	0	0	0	0	0	0	0
0	0	0	0	0	0	0	0	0
18	14	17	18	19	22	16	17	18
1	1	2	3	0	0	0	0	0
0	0	0	0	0	0	0	0	0
0	0	0	0	0	0	0	0	0
0	0	0	0	0	0	0	0	0
0	0	0	0	2	1	0	0	1
0	0	0	0	0	0	0	0	0
0	0	0	0	0	0	0	0	0
0	0	0	0	0	0	0	0	0
2	1	1	4	3	0	3	2	1
0	0	0	0	0	0	0	0	0
0	0	0	0	0	0	0	0	0
0	0	0	0	0	0	0	0	0
0	0	0	0	0	2	0	0	1
0	0	0	0	0	0	0	0	0
0	0	0	0	0	0	0	0	0
2	5	3	11	5	2	3	6	2

0	0	0	0	0	0	0	0	0
0	0	0	0	0	0	0	0	0
4	0	3	2	5	9	10	3	4
6	4	3	2	9	7	6	2	1
4	2	2	2	6	3	2	4	3
0	2	0	0	0	0	0	0	0
6	3	5	3	6	6	7	4	3
0	0	0	0	0	1	0	0	0
2	2	3	4	2	3	1	4	2
0	0	0	0	0	0	0	0	0
0	0	0	0	0	0	0	0	0
0	0	0	0	0	0	0	0	0
1	0	0	0	0	0	0	0	0
0	0	0	0	0	0	0	0	0
8	9	12	17	35	40	29	7	9
7	8	9	17	13	10	14	7	11
0	0	0	0	0	0	0	0	0
0	0	0	0	0	0	0	0	0
0	0	0	0	0	0	0	0	0
8	5	9	8	10	7	10	4	8
0	0	0	0	0	0	0	0	0
0	0	0	0	0	0	0	0	0
0	0	0	0	0	0	0	0	0
0	0	0	0	0	0	0	0	0
0	0	0	0	0	0	0	0	0
0	0	0	0	0	0	0	0	0
2	0	0	0	0	0	0	0	0
1	0	0	2	4	4	0	2	0
0	0	0	0	0	0	0	0	0
0	0	0	0	0	0	0	0	0
0	0	0	0	0	0	0	0	0
0	0	0	0	0	0	0	0	0
0	0	0	0	0	0	0	0	0
0	1	0	0	0	0	0	0	0
0	0	0	0	0	0	0	0	0
0	0	0	0	0	0	0	0	0
0	0	0	0	0	0	0	0	0
1	1	0	2	0	0	1	0	0
12	15	16	23	29	19	10	25	16
9	6	10	10	16	21	13	8	7
0	0	0	0	0	0	0	0	0
0	0	0	0	0	0	0	0	0
3	1	2	1	0	1	1	1	1
0	0	0	0	0	0	0	0	0
0	0	0	0	0	0	0	0	0
0	0	0	0	0	0	0	0	0
0	0	0	0	0	0	0	0	0
0	0	1	0	0	0	0	0	0
0	0	1	0	0	0	0	0	0
0	0	0	0	0	0	0	0	0
0	0	1	0	1	0	0	0	0
0	0	0	0	0	0	0	0	0
0	0	0	0	0	0	0	0	0
0	0	0	0	0	1	0	0	0
0	0	0	0	0	0	0	0	0
49	51	41	79	63	70	43	48	32
0	1	1	0	0	0	0	0	0
0	0	0	0	0	0	0	0	0
0	0	0	1	0	0	0	0	0
0	0	0	0	0	0	0	0	0
0	0	1	1	0	0	0	0	0
0	1	1	1	0	3	1	1	1
0	0	0	0	0	0	0	0	0
0	0	0	0	0	0	0	0	0
0	0	0	0	0	0	0	0	0
0	0	0	0	0	0	0	0	0
0	0	0	0	1	0	1	0	0

0	0	0	0	0	0	0	0	0
0	0	0	0	0	0	0	0	0
0	0	0	0	0	0	0	0	0
0	0	0	0	0	0	0	0	0
0	0	0	0	0	0	0	0	0
0	0	0	0	0	0	1	0	0
0	0	0	0	0	0	0	0	0
0	0	0	0	0	0	0	0	0
0	0	0	0	0	0	0	0	0
0	0	0	0	0	0	0	0	0
0	0	0	0	1	0	0	0	0
0	0	0	0	0	1	0	0	0
0	0	0	0	0	0	0	0	0
0	0	0	0	0	0	0	0	0
0	0	0	0	0	0	0	0	0
0	0	0	0	0	1	0	0	0
0	0	0	0	0	0	0	0	0
0	0	0	0	0	0	0	0	0
0	0	0	0	0	0	0	0	0
0	0	0	0	0	0	0	0	0
0	0	0	0	0	0	0	0	0
0	0	0	0	0	0	0	0	0
0	1	0	1	0	4	0	1	0
0	0	0	0	0	0	0	0	0
0	0	0	0	0	0	0	1	0
0	0	0	0	0	0	0	0	0
0	0	0	0	0	0	0	0	0
0	0	0	0	0	0	0	0	0
0	0	0	0	0	0	0	0	0
4	3	5	2	6	8	2	5	5
1	1	0	1	1	1	0	3	1
8	13	9	8	16	10	12	11	6
0	0	0	1	2	2	1	2	0
0	0	0	1	0	2	0	0	0
0	0	3	3	1	3	1	0	0
0	0	0	0	0	0	0	0	0
0	0	0	0	0	0	0	0	0
0	0	0	0	0	0	0	0	0
0	0	0	0	0	0	0	0	0
0	0	0	0	0	0	0	0	0
0	0	0	0	0	0	0	0	0
0	0	0	0	0	0	0	0	0
0	0	0	0	0	0	0	0	0
0	0	0	0	0	0	0	0	0
0	0	0	0	0	0	0	0	0
0	0	0	0	0	0	0	0	0
0	1	0	0	2	1	2	1	0
0	0	0	0	0	0	0	0	0
0	0	0	0	0	0	0	0	0
0	0	1	2	0	1	0	0	0
0	0	0	0	0	1	0	0	0
1	0	1	1	0	0	1	0	0
0	0	0	0	0	0	0	0	0
0	0	0	0	0	0	0	0	0
0	0	0	0	1	0	1	0	0
0	0	1	0	0	0	0	1	0
0	0	0	0	0	0	0	0	0
0	0	0	0	0	0	0	0	0
0	0	0	0	0	0	0	0	0
0	0	0	0	0	0	0	0	0
0	0	0	0	0	0	0	0	0
0	0	0	0	0	0	0	0	0
0	0	0	0	0	0	0	0	0
0	0	0	0	0	0	0	0	0
0	0	0	0	0	0	0	0	0
0	0	0	0	0	0	0	0	0
0	0	0	0	0	0	0	0	0

0	0	0	0	0	0	0	0	0
0	0	1	0	0	1	0	1	0
1	3	2	3	2	4	4	1	0
0	0	0	0	0	0	0	0	0
0	0	0	0	0	0	0	0	0
0	0	0	0	0	0	0	0	0
1	1	1	2	3	3	3	1	0
4	0	2	5	6	0	3	5	5
1	2	0	1	1	1	3	0	0
5	3	5	5	17	5	5	4	2
4	1	0	0	2	1	1	0	0
0	0	0	0	0	0	0	0	0
0	0	0	0	0	0	0	0	0
0	0	0	0	0	0	0	0	0
0	0	0	0	0	0	0	0	0
0	0	0	0	0	0	0	0	0
0	0	0	0	0	0	0	0	0
0	0	0	0	0	0	0	0	0
0	0	0	0	0	0	0	0	0
0	0	0	0	0	0	0	0	0
0	0	0	0	0	0	0	0	0
0	0	0	0	0	0	1	0	0
0	0	0	0	0	0	0	0	0
0	0	0	1	0	0	0	0	0
0	1	2	1	2	3	2	0	0
0	0	0	0	0	0	0	0	0
1	2	0	0	0	0	0	1	1
0	0	0	0	0	0	0	0	0
1	0	1	0	3	0	0	5	0
0	0	0	0	0	0	0	0	0
0	1	0	0	0	0	0	0	0
0	0	0	0	0	0	0	0	0
0	0	0	0	0	0	0	0	0
0	0	0	0	0	0	0	0	0
0	0	0	0	0	0	0	0	0
0	0	0	0	0	0	0	0	0
1	1	2	4	6	2	1	1	0
2	1	0	4	0	1	2	0	0
0	0	0	0	0	0	0	0	0
0	0	0	0	0	0	0	0	0
0	0	0	0	0	0	0	0	0
2	3	3	2	9	3	2	1	0
0	0	0	0	1	0	0	0	0
0	0	0	0	0	0	0	0	0
0	0	0	0	0	0	0	0	0
0	0	0	0	0	0	0	0	0
0	0	0	0	0	0	0	0	0
0	0	0	0	0	0	0	0	0
0	0	0	0	0	0	0	0	0
0	0	0	0	0	0	0	0	0
0	0	0	0	0	0	0	0	0
0	0	0	0	0	0	0	0	0
0	0	0	0	0	0	0	0	0
12	11	17	15	19	26	14	15	10
0	0	0	0	0	0	0	0	0
0	0	0	0	0	0	0	0	0
0	1	0	0	0	0	0	0	0
0	0	0	0	0	0	0	0	0
0	0	0	0	0	0	0	0	0
1	2	3	0	1	0	2	0	0
0	0	0	0	0	0	0	0	0
0	0	0	0	0	0	0	0	0
0	1	2	2	1	3	1	0	0
0	0	0	0	0	1	0	0	0
0	0	0	0	1	0	2	0	0
0	0	1	0	0	0	0	0	0
0	0	0	1	0	0	0	0	0

0	0	0	0	0	0	0	0	0
0	0	0	0	0	0	0	0	0
0	0	0	0	0	0	0	0	0
0	1	1	0	0	0	0	0	0
0	0	0	0	0	0	0	0	0
0	0	0	0	0	0	0	0	0
0	0	0	0	0	0	0	0	0
0	0	0	0	0	0	0	0	0
0	0	0	0	0	0	0	0	0
0	0	0	0	0	0	0	0	0
0	0	0	0	0	0	0	0	0
0	0	0	0	0	0	0	0	0
0	0	0	0	0	0	0	0	0
0	0	0	0	0	0	0	0	0
0	1	1	0	0	3	1	1	1
0	0	0	0	0	0	0	0	0
0	0	0	0	0	0	0	0	0
0	0	0	0	0	0	0	0	0
0	0	1	3	4	1	0	0	0
0	0	0	0	0	0	0	0	0
0	0	0	0	0	0	0	0	0
0	0	0	0	0	0	0	0	0
1	0	0	1	0	3	0	0	0
0	0	0	1	0	0	0	0	0
0	0	0	0	0	0	0	0	0
0	0	0	0	0	0	0	0	0
0	0	0	0	0	0	0	0	0
0	0	0	0	0	0	0	0	0
0	0	0	0	0	0	0	0	0
0	0	0	0	0	0	0	0	0
0	0	0	0	0	0	0	0	0
0	0	0	0	0	0	0	0	0
0	0	0	0	0	0	0	0	0
0	0	0	0	0	0	0	0	0
0	0	0	0	0	0	0	0	0
0	0	0	0	0	0	0	0	0
0	0	0	0	0	0	0	0	0
0	0	0	0	0	0	0	0	0
0	0	0	0	0	0	0	0	0
0	0	0	0	0	0	0	0	0
0	0	0	0	0	0	0	0	0
0	0	0	0	0	0	0	0	0
0	1	1	0	0	0	1	0	0
0	0	0	0	0	0	0	0	0
0	0	0	1	0	0	0	0	0
3	5	11	9	11	8	2	3	3
0	0	0	0	0	0	0	0	0
2	0	1	2	3	1	0	0	0
0	0	0	0	0	0	0	0	0
0	1	0	0	2	0	0	0	0
0	1	4	10	8	5	4	0	1
0	0	0	0	0	0	0	0	0
0	0	0	0	0	0	0	0	0
0	0	1	0	0	0	0	0	2
0	0	0	2	1	1	1	1	0
0	0	2	0	1	0	0	1	0
0	0	0	0	0	0	0	0	0
0	0	0	0	0	0	0	0	0
0	0	0	0	0	0	0	0	0
0	0	0	0	0	0	0	0	0
0	0	0	0	0	0	0	0	0
0	0	0	0	0	0	0	0	0
0	0	0	0	0	0	0	0	0
0	0	0	0	0	0	0	0	0
0	0	0	0	0	0	0	0	0
0	0	0	0	0	0	0	0	0
0	0	0	0	0	0	0	0	0

0	0	0	0	0	0	0	0	0
0	0	0	0	0	0	0	0	0
0	0	0	0	0	0	0	0	0
0	0	0	0	0	0	0	0	0
0	0	0	0	0	0	0	0	0
0	0	0	0	0	0	0	0	0
0	0	0	0	0	0	0	0	0
0	0	0	0	0	0	0	0	0
0	0	0	0	0	0	0	0	0
0	0	0	0	0	0	0	0	0
0	0	0	0	0	0	0	0	0
0	0	0	0	0	0	0	0	0
0	0	0	0	0	0	0	0	0
0	0	0	0	0	0	0	0	0
0	0	0	0	0	0	0	0	0
0	0	0	0	0	0	0	0	0
0	0	0	0	0	0	0	0	0
0	0	0	0	0	0	0	0	0
0	0	0	0	0	0	0	0	0
0	0	0	0	0	0	0	0	0
0	0	0	0	0	0	0	0	0
0	0	0	0	0	0	0	0	0
0	0	0	0	0	0	0	0	0
0	0	0	0	0	0	0	0	0
0	0	0	0	0	0	0	0	0
4	4	5	5	3	6	8	7	4
0	0	0	0	0	0	0	0	0
0	0	0	0	0	0	0	0	0
0	0	0	0	0	0	0	0	0
0	0	0	0	0	0	0	0	0
1	1	0	0	1	1	1	1	1
0	0	0	0	0	0	0	0	0
0	0	0	0	0	0	0	0	0
0	0	0	0	0	0	0	0	0
0	0	0	0	0	0	0	0	0
0	0	0	0	0	0	0	0	0
0	0	0	0	0	0	0	0	0
0	0	0	1	0	0	0	0	0
1	1	0	0	0	0	0	0	1
