# Supplementary material for: A decline in molluscan carbonate production driven by the loss of vegetated habitats encoded in the Holocene sedimentary record of the Gulf of Trieste
Source: Sedimentology. 2018 Aug 25;66(3):781–807. doi: 10.1111/sed.12516 (PMC6446828; doi:10.1111/sed.12516)
Supplement: Supplementary file 2 — Table S2. Amino acid racemization data and calibrated estimates of post‐mortem age of Gouldia minima collected from the shell bed at Piran 1 and Piran 2. [file SED-66-781-s002.pdf]

| Age | UAL_num | Injection | Hydrolysis | Notes | Station | specimen_no |
|---|---|---|---|---|---|---|
| 3044 | 11365 | 2ul10-4ul12 | 6 | | Piran I | PiranI M1 0-2-01 |
| 1054 | 11366 | 2ul10 | 6 | | Piran I | PiranI M1 0-2-02 |
| 1849 | 11367 | 2ul10 | 6 | | Piran I | PiranI M1 0-2-03 |
| 517 | 11368 | 2ul10 | 6 | | Piran I | PiranI M1 0-2-04 |
| 2415 | 11369 | 2ul10 | 6 | | Piran I | PiranI M1 0-2-05 |
| 1504 | 11370 | 2ul10 | 6 | | Piran I | PiranI M1 0-2-06 |
| 735 | 11371 | 2ul10 | 6 | | Piran I | PiranI M1 0-2-07 |
| 489 | 11372 | 2ul10 | 6 | | Piran I | PiranI M1 0-2-08 |
| 1795 | 11373 | 2ul10 | 6 | | Piran I | PiranI M1 0-2-09 |
| 1856 | 11374 | 2ul10 | 6 | | Piran I | PiranI M1 0-2-10 |
| 1147 | 11375 | 2ul10 | 6 | | Piran I | PiranI M1 0-2-11 |
| 1071 | 11376 | 2ul10 | 6 | | Piran I | PiranI M1 0-2-12 |
| 2539 | 11377 | 2ul10 | 6 | | Piran I | PiranI M1 0-2-13 |
| 2084 | 11378 | 2ul10 | 6 | | Piran I | PiranI M1 0-2-14 |
| 2848 | 11379 | 2ul10 | 6 | | Piran I | PiranI M1 0-2-15 |
| 1228 | 11380 | 2ul10 | 6 | | Piran I | PiranI M1 0-2-16 |
| 1612 | 11381 | 2ul10 | 6 | | Piran I | PiranI M1 0-2-17 |
| 292 | 11382 | 2ul10 | 6 | | Piran I | PiranI M1 0-2-18 |
| 847 | 11383 | 2ul10 | 6 | | Piran I | PiranI M1 0-2-19 |
| 1027 | 11384 | 2ul10 | 6 | | Piran I | PiranI M1 0-2-20 |
| 5311 | 11385 | 2ul10 | 6 | | Piran I | PiranI M1 0-2-21 |
| 320 | 11386 | 2ul10 | 6 | | Piran I | PiranI M1 0-2-22 |
| 486 | 11387 | 2ul10 | 6 | | Piran I | PiranI M1 0-2-23 |
| NA | 11388 | 2ul10 | 6 | | Piran I | PiranI M1 0-2-24 |
| 319 | 11389 | 2ul10 | 6 | | Piran I | PiranI M1 0-2-25 |
| 332 | 11390 | 4ul12 | 6 | | Piran I | PiranI M1 0-2-26 |
| 1176 | 11391 | 2ul10 | 6 | | Piran I | PiranI M1 0-2-27 |
| 72 | 11392 | 2ul10 | 6 | | Piran I | PiranI M1 0-2-28 |
| 287 | 11393 | 2ul10 | 6 | | Piran I | PiranI M1 0-2-29 |
| 1594 | 11394 | 2ul10 | 6 | | Piran I | PiranI M1 0-2-30 |
| 1385 | 11395 | 2ul12 | 6 | | Piran I | PiranI M1 8-10-01 |
| 239 | 11396 | 2ul12 | 6 | | Piran I | PiranI M1 8-10-02 |
| 613 | 11397 | 2ul10 | 6 | | Piran I | PiranI M1 8-10-03 |
| 276 | 11398 | 2ul10 | 6 | | Piran I | PiranI M1 8-10-04 |
| 2514 | 11399 | 2ul10 | 6 | | Piran I | PiranI M1 8-10-05 |
| 1086 | 11400 | 2ul10 | 6 | | Piran I | PiranI M1 8-10-06 |
| 1136 | 11401 | 2ul10 | 6 | | Piran I | PiranI M1 8-10-07 |
| 1887 | 11402 | 2ul10 | 6 | | Piran I | PiranI M1 8-10-08 |
| 2289 | 11403 | 2ul10 | 6 | | Piran I | PiranI M1 8-10-09 |
| 1988 | 11404 | 2ul10 | 6 | | Piran I | PiranI M1 8-10-10 |
| 781 | 11405 | 2ul10 | 6 | | Piran I | PiranI M1 8-10-11 |
| 2824 | 11406 | 2ul10 | 6 | | Piran I | PiranI M1 8-10-12 |
| 1374 | 11407 | 2ul10 | 6 | | Piran I | PiranI M1 8-10-13 |
| 286 | 11408 | 2ul10 | 6 | | Piran I | PiranI M1 8-10-14 |
| 989 | 11409 | 2ul10 | 6 | | Piran I | PiranI M1 8-10-15 |
| 222 | 11410 | 2ul10 | 6 | | Piran I | PiranI M1 8-10-16 |
| 1001 | 11411 | 2ul10 | 6 | | Piran I | PiranI M1 8-10-17 |
| 1062 | 11412 | 2ul10 | 6 | | Piran I | PiranI M1 8-10-18 |
| 2285 | 11413 | 2ul10 | 6 | | Piran I | PiranI M1 8-10-19 |
| 1128 | 11414 | 2ul10 | 6 | | Piran I | PiranI M1 8-10-20 |

| | | | | | |
|---|---|---|---|---|---|
| 1621 | 11415 | 2ul10 | 6 | Piran I | PiranI M1 8-10-21 |
| 409 | 11416 | 2ul10 | 6 | Piran I | PiranI M1 8-10-22 |
| 813 | 11417 | 2ul10 | 6 | Piran I | PiranI M1 8-10-23 |
| 618 | 11418 | 2ul10 | 6 | Piran I | PiranI M1 8-10-24 |
| 1394 | 11419 | 2ul10 | 6 | Piran I | PiranI M1 8-10-25 |
| 954 | 11420 | 2ul10 | 6 | Piran I | PiranI M1 8-10-26 |
| 697 | 11421 | 2ul10 | 6 | Piran I | PiranI M1 8-10-27 |
| 627 | 11422 | 2ul10 | 6 | Piran I | PiranI M1 8-10-28 |
| 615 | 11423 | 2ul10 | 6 | Piran I | PiranI M1 8-10-29 |
| 1029 | 11424 | 2ul10 | 6 | Piran I | PiranI M1 8-10-30 |
| 3034 | 11425 | 2ul12 | 6 | Piran I | PiranI M1 20-25-01 |
| 4467 | 11427 | 2ul12 | 6 | Piran I | PiranI M1 20-25-03 |
| 2937 | 11428 | 2ul12 | 6 | Piran I | PiranI M1 20-25-04 |
| 4548 | 11429 | 2ul12 | 6 | Piran I | PiranI M1 20-25-05 |
| NA | 11430 | 2ul12 | 6 | Piran I | PiranI M1 20-25-06 |
| 4041 | 11431 | 2ul10 | 6 | Piran I | PiranI M1 20-25-07 |
| 4477 | 11432 | 2ul10 | 6 | Piran I | PiranI M1 20-25-08 |
| 4085 | 11433 | 2ul10 | 6 | Piran I | PiranI M1 20-25-09 |
| 4519 | 11434 | 2ul12 | 6 | Piran I | PiranI M1 20-25-10 |
| 2846 | 11435 | 2ul12 | 6 | Piran I | PiranI M1 20-25-11 |
| 2823 | 11436 | 2ul12 | 6 | Piran I | PiranI M1 20-25-12 |
| 3408 | 11437 | 2ul12 | 6 | Piran I | PiranI M1 20-25-13 |
| 3515 | 11438 | 2ul12 | 6 | Piran I | PiranI M1 20-25-14 |
| 6992 | 11439 | 2ul12 | 6 | Piran I | PiranI M1 20-25-15 |
| 863 | 11440 | 2ul10 | 6 | Piran I | PiranI M1 20-25-16 |
| 4810 | 11441 | 2ul10 | 6 | Piran I | PiranI M1 20-25-17 |
| 6647 | 11442 | 2ul10 | 6 | Piran I | PiranI M1 20-25-18 |
| 3574 | 11443 | 2ul12 | 6 | Piran I | PiranI M1 20-25-19 |
| NA | 11444 | 2ul10 | 6 | Piran I | PiranI M1 20-25-20 |
| 3138 | 11445 | 2ul10 | 6 | Piran I | PiranI M1 20-25-21 |
| 906 | 11446 | 2ul10 | 6 | Piran I | PiranI M1 20-25-22 |
| 4210 | 11447 | 2ul10 | 6 | Piran I | PiranI M1 20-25-23 |
| 3621 | 11448 | 2ul10 | 6 | Piran I | PiranI M1 20-25-24 |
| 4256 | 11449 | 2ul10 | 6 | Piran I | PiranI M1 20-25-25 |
| 3354 | 11450 | 2ul10 | 6 | Piran I | PiranI M1 20-25-26 |
| 1973 | 11451 | 2ul10 | 6 | Piran I | PiranI M1 20-25-27 |
| 3708 | 11452 | 2ul10 | 6 | Piran I | PiranI M1 20-25-28 |
| 3938 | 11453 | 2ul10 | 6 | Piran I | PiranI M1 20-25-29 |
| 2151 | 11454 | 2ul10 | 6 | Piran I | PiranI M1 20-25-30 |
| 4663 | 11455 | 2ul12 | 6 | Piran I | PiranI M1 40-45-01 |
| 5164 | 11456 | 2ul10 | 6 | Piran I | PiranI M1 40-45-02 |
| 4090 | 11457 | 2ul10 | 6 | Piran I | PiranI M1 40-45-03 |
| 4981 | 11458 | 2ul10 | 6 | Piran I | PiranI M1 40-45-04 |
| 5454 | 11459 | 2ul10 | 6 | Piran I | PiranI M1 40-45-05 |
| 3798 | 11460 | 2ul10 | 6 | Piran I | PiranI M1 40-45-06 |
| 3509 | 11461 | 2ul10 | 6 | Piran I | PiranI M1 40-45-07 |
| 6031 | 11462 | 2ul12 | 6 | Piran I | PiranI M1 40-45-08 |
| 4914 | 11463 | 2ul10 | 6 | Piran I | PiranI M1 40-45-09 |
| 3627 | 11464 | 2ul10 | 6 | Piran I | PiranI M1 40-45-10 |
| 6753 | 11465 | 2ul10 | 6 | Piran I | PiranI M1 40-45-11 |
| 3571 | 11466 | 2ul10 | 6 | Piran I | PiranI M1 40-45-12 |
| 4528 | 11467 | 2ul10 | 6 | Piran I | PiranI M1 40-45-13 |
| 5007 | 11468 | 2ul10 | 6 | Piran I | PiranI M1 40-45-14 |
| 4998 | 11469 | 2ul10 | 6 | Piran I | PiranI M1 40-45-15 |
| 8409 | 11470 | 2ul10 | 6 | Piran I | PiranI M1 40-45-16 |
| 2894 | 11471 | 2ul10 | 6 | Piran I | PiranI M1 40-45-17 |

| | | | | | |
|---|---|---|---|---|---|
| 4714 | 11472 | 2ul10 | 6 | Piran I | PiranI M1 40-45-18 |
| 3865 | 11473 | 2ul10 | 6 | Piran I | PiranI M1 40-45-19 |
| 4152 | 11474 | 2ul10 | 6 | Piran I | PiranI M1 40-45-20 |
| 4196 | 11475 | 2ul12 | 6 | Piran I | PiranI M1 40-45-21 |
| 5563 | 11476 | 2ul10 | 6 | Piran I | PiranI M1 40-45-22 |
| 3648 | 11477 | 2ul10 | 6 | Piran I | PiranI M1 40-45-23 |
| 4284 | 11478 | 2ul10 | 6 | Piran I | PiranI M1 40-45-24 |
| 3601 | 11479 | 2ul10-4ul12 | 6 | Piran I | PiranI M1, 40-45, 25 |
| 5234 | 11480 | 2ul10 | 6 | Piran I | PiranI M1, 40-45, 26 |
| 7132 | 11481 | 2ul10 | 6 | Piran I | PiranI M1, 40-45, 27 |
| 5116 | 11482 | 2ul10 | 6 | Piran I | PiranI M1, 40-45, 28 |
| 4700 | 11483 | 2ul10 | 6 | Piran I | PiranI M1, 40-45, 29 |
| 3040 | 11484 | 2ul10 | 6 | Piran I | PiranI M1, 40-45, 30 |
| 3748 | 11485 | 2ul12 | 6 | Piran I | PiranI M1 60-65-01 |
| 4604 | 11486 | 2ul10 | 6 | Piran I | PiranI M1 60-65-02 |
| 6181 | 11487 | 2ul10 | 6 | Piran I | PiranI M1 60-65-03 |
| 4359 | 11488 | 2ul10 | 6 | Piran I | PiranI M1 60-65-04 |
| 3147 | 11489 | 2ul12 | 6 | Piran I | PiranI M1 60-65-05 |
| 5517 | 11490 | 2ul10 | 6 | Piran I | PiranI M1 60-65-06 |
| 4971 | 11491 | 2ul10 | 6 | Piran I | PiranI M1 60-65-07 |
| 4785 | 11492 | 2ul10 | 6 | Piran I | PiranI M1 60-65-08 |
| 4335 | 11493 | 2ul10 | 6 | Piran I | PiranI M1 60-65-09 |
| 6869 | 11494 | 2ul10 | 6 | Piran I | PiranI M1 60-65-10 |
| 4647 | 11495 | 2ul10 | 6 | Piran I | PiranI M1 60-65-11 |
| 4313 | 11496 | 2ul10 | 6 | Piran I | PiranI M1 60-65-12 |
| 4413 | 11497 | 2ul10 | 6 | Piran I | PiranI M1 60-65-13 |
| 7116 | 11498 | 2ul10 | 6 | Piran I | PiranI M1 60-65-14 |
| 6742 | 11499 | 2ul10 | 6 | Piran I | PiranI M1 60-65-15 |
| 4451 | 11500 | 2ul10 | 6 | Piran I | PiranI M1 60-65-16 |
| 4107 | 11501 | 2ul10 | 6 | Piran I | PiranI M1 60-65-17 |
| 6828 | 11502 | 2ul10 | 6 | Piran I | PiranI M1 60-65-18 |
| NA | 11503 | 2ul10 | 6 | Piran I | PiranI M1 60-65-19 |
| 8102 | 11504 | 2ul10 | 6 | Piran I | PiranI M1 60-65-20 |
| 5413 | 11505 | 2ul12 | 6 | Piran I | PiranI M1 60-65-21 |
| 6296 | 11506 | 2ul10 | 6 | Piran I | PiranI M1 60-65-22 |
| 4202 | 11507 | 2ul10 | 6 | Piran I | PiranI M1 60-65-23 |
| 5297 | 11508 | 2ul10 | 6 | Piran I | PiranI M1 60-65-24 |
| 4683 | 11509 | 2ul10 | 6 | Piran I | PiranI M1 60-65-25 |
| 10632 | 11510 | 2ul10 | 6 | Piran I | PiranI M1 60-65-26 |
| 5323 | 11511 | 2ul10 | 6 | Piran I | PiranI M1 60-65-27 |
| 3180 | 11512 | 2ul10 | 6 | Piran I | PiranI M1 60-65-28 |
| 2659 | 11513 | 2ul10 | 6 | Piran I | PiranI M1 60-65-29 |
| 10388 | 11514 | 2ul10 | 6 | Piran I | PiranI M1 60-65-30 |
| 3895 | 11515 | 2ul10 | 6 | Piran I | PiranI M1 85-90-01 |
| 5050 | 11516 | 2ul10 | 6 | Piran I | PiranI M1 85-90-02 |
| 5804 | 11517 | 2ul10 | 6 | Piran I | PiranI M1 85-90-03 |
| 6638 | 11518 | 2ul10 | 6 | Piran I | PiranI M1 85-90-04 |
| 7513 | 11519 | 2ul10 | 6 | Piran I | PiranI M1 85-90-05 |
| 6849 | 11520 | 2ul10 | 6 | Piran I | PiranI M1 85-90-06 |
| 2596 | 11521 | 2ul10 | 6 | Piran I | PiranI M1 85-90-07 |
| 5009 | 11522 | 2ul10 | 6 | Piran I | PiranI M1 85-90-08 |
| 5746 | 11523 | 2ul10 | 6 | Piran I | PiranI M1 85-90-09 |
| 3089 | 11524 | 2ul10 | 6 | Piran I | PiranI M1 85-90-10 |
| 6164 | 11525 | 2ul10 | 6 | Piran I | PiranI M1 85-90-11 |
| 5364 | 11526 | 2ul10 | 6 | Piran I | PiranI M1 85-90-12 |
| 6199 | 11527 | 2ul10 | 6 | Piran I | PiranI M1 85-90-13 |

| | | | | | |
|---|---|---|---|---|---|
| 3714 | 11528 | 2ul10 | 6 | Piran I | PiranI M1 85-90-14 |
| NA | 11529 | 2ul10 | 6 | Piran I | PiranI M1 85-90-15 |
| 5366 | 11530 | 2ul10 | 6 | Piran I | PiranI M1 85-90-16 |
| 9244 | 11531 | 2ul10 | 6 | Piran I | PiranI M1 85-90-17 |
| 6353 | 11532 | 2ul10 | 6 | Piran I | PiranI M1 85-90-18 |
| 12225 | 11534 | 2ul10 | 6 | Piran I | PiranI M1 85-90-20 |
| 4067 | 11535 | 2ul10 | 6 | Piran I | PiranI M1 85-90-21 |
| 6037 | 11536 | 2ul10 | 6 | Piran I | PiranI M1 85-90-22 |
| 4303 | 11537 | 2ul10 | 6 | Piran I | PiranI M1 85-90-23 |
| 8305 | 11538 | 2ul10 | 6 | Piran I | PiranI M1 85-90-24 |
| 4054 | 11539 | 2ul10 | 6 | Piran I | PiranI M1 110-115-01 |
| 4335 | 11540 | 2ul12 | 6 | Piran I | PiranI M1 110-115-02 |
| 5932 | 11541 | 2ul10 | 6 | Piran I | PiranI M1 110-115-03 |
| 4359 | 11542 | 2ul10 | 6 | Piran I | PiranI M1 110-115-04 |
| 4841 | 11543 | 2ul10 | 6 | Piran I | PiranI M1 110-115-05 |
| 5740 | 11544 | 2ul10 | 6 | Piran I | PiranI M1 110-115-06 |
| 5464 | 11545 | 2ul10 | 6 | Piran I | PiranI M1 110-115-07 |
| 7329 | 11546 | 2ul10 | 6 | Piran I | PiranI M1 110-115-08 |
| 5166 | 11547 | 2ul10 | 6 | Piran I | PiranI M1 110-115-09 |
| 5905 | 11548 | 2ul10 | 6 | Piran I | PiranI M1 110-115-10 |
| 5691 | 11549 | 2ul10 | 6 | Piran I | PiranI M1 110-115-11 |
| 5101 | 11550 | 2ul10 | 6 | Piran I | PiranI M1 110-115-12 |
| 4763 | 11551 | 2ul10 | 6 | Piran I | PiranI M1 110-115-13 |
| 6184 | 11552 | 2ul10 | 6 | Piran I | PiranI M1 110-115-14 |
| 7340 | 11553 | 2ul10 | 6 | Piran I | PiranI M1 110-115-15 |
| 3415 | 11554 | 2ul10 | 6 | Piran I | PiranI M1 110-115-16 |
| 6994 | 11555 | 2ul10 | 6 | Piran I | PiranI M1 110-115-17 |
| 7136 | 11556 | 2ul10 | 6 | Piran I | PiranI M1 110-115-18 |
| 3298 | 11557 | 2ul10 | 6 | Piran I | PiranI M1 110-115-19 |
| 7460 | 11558 | 2ul10 | 6 | Piran I | PiranI M1 110-115-20 |
| 4449 | 11559 | 2ul10 | 6 | Piran I | PiranI M1 110-115-21 |
| 5876 | 11560 | 2ul10 | 6 | Piran I | PiranI M1 110-115-22 |
| 5649 | 11562 | 2ul10 | 6 | Piran I | PiranI M1 110-115-24 |
| 4228 | 11563 | 2ul12 | 6 | Piran I | PiranI M1 135-140-01 |
| 4302 | 11564 | 2ul10 | 6 | Piran I | PiranI M1 135-140-02 |
| 3380 | 11565 | 2ul10 | 6 | Piran I | PiranI M1 135-140-03 |
| 5844 | 11566 | 2ul10 | 6 | Piran I | PiranI M1 135-140-04 |
| 4411 | 11567 | 2ul10 | 6 | Piran I | PiranI M1 135-140-05 |
| 4426 | 11568 | 2ul10 | 6 | Piran I | PiranI M1 135-140-06 |
| 3717 | 11569 | 2ul10 | 6 | Piran I | PiranI M1 135-140-07 |
| 6551 | 11570 | 2ul10 | 6 | Piran I | PiranI M1 135-140-08 |
| 4335 | 11571 | 2ul10 | 6 | Piran I | PiranI M1 135-140-09 |
| 4158 | 11572 | 2ul10 | 6 | Piran I | PiranI M1 135-140-10 |
| 3937 | 11573 | 2ul10 | 6 | Piran I | PiranI M1 135-140-11 |
| 4953 | 11574 | 2ul10 | 6 | Piran I | PiranI M1 135-140-12 |
| 3715 | 11575 | 2ul10 | 6 | Piran I | PiranI M1 135-140-13 |
| 5847 | 11576 | 2ul10 | 6 | Piran I | PiranI M1 135-140-14 |
| 4627 | 11577 | 2ul10 | 6 | Piran I | PiranI M1 135-140-15 |
| 5014 | 11578 | 2ul10 | 6 | Piran I | PiranI M1 135-140-16 |
| 3148 | 11579 | 2ul10 | 6 | Piran I | PiranI M1 135-140-17 |
| 6315 | 11580 | 2ul10 | 6 | Piran I | PiranI M1 135-140-18 |
| 3946 | 11581 | 2ul10 | 6 | Piran I | PiranI M1 135-140-19 |
| 5463 | 11582 | 2ul10 | 6 | Piran I | PiranI M1 135-140-20 |
| 5086 | 11583 | 2ul10 | 6 | Piran I | PiranI M1 135-140-21 |
| 6021 | 11584 | 2ul10 | 6 | Piran I | PiranI M1 135-140-22 |
| 5172 | 11585 | 2ul10 | 6 | Piran I | PiranI M1 135-140-23 |

| | | | | | |
|---|---|---|---|---|---|
| 3936 | 11586 | 2ul10 | 6 | Piran I | PiranI M1 135-140-24 |
| 10265 | 11587 | 2ul10 | 6 | Piran I | PiranI M1 135-140-25 |
| 2919 | 11588 | 2ul10 | 6 | Piran I | PiranI M1 135-140-26 |
| NA | 11589 | 2ul10 | 6 | Piran I | PiranI M1 135-140-27 |
| 5149 | 11590 | 2ul10 | 6 | Piran I | PiranI M1 135-140-28 |
| 4088 | 11591 | 2ul10 | 6 | Piran I | PiranI M1 135-140-29 |
| 5483 | 11592 | 2ul10 | 6 | Piran I | PiranI M1 135-140-30 |
| 3064 | 12989 | 2ul10 | 6 | Piran I | PiranI M1, 14-16, 01 |
| 5402 | 12990 | 2ul10 | 6 | Piran I | PiranI M1, 14-16, 02 |
| 1377 | 12991 | 2ul10 | 6 | Piran I | PiranI M1, 14-16, 03 |
| 3794 | 12992 | 2ul10 | 6 | Piran I | PiranI M1, 14-16, 04 |
| 1495 | 12993 | 2ul10 | 6 | Piran I | PiranI M1, 14-16, 05 |
| 3887 | 12994 | 2ul10 | 6 | Piran I | PiranI M1, 14-16, 06 |
| 1697 | 12995 | 2ul10 | 6 | Piran I | PiranI M1, 14-16, 07 |
| 5407 | 12996 | 2ul10 | 6 | Piran I | PiranI M1, 14-16, 08 |
| 1973 | 12997 | 2ul10 | 6 | Piran I | PiranI M1, 14-16, 09 |
| 6075 | 12998 | 2ul10 | 6 | Piran I | PiranI M1, 14-16, 10 |
| 1507 | 12999 | 2ul10 | 6 | Piran I | PiranI M1, 14-16, 11 |
| 3210 | 13000 | 2ul10 | 6 | Piran I | PiranI M1, 14-16, 12 |
| 4151 | 13001 | 2ul10 | 6 | Piran I | PiranI M1, 14-16, 13 |
| 3393 | 13002 | 2ul10 | 6 | Piran I | PiranI M1, 14-16, 14 |
| NA | 13003 | 2ul10 | 6 | Piran I | PiranI M1, 14-16, 15 |
| 3634 | 13004 | 2ul10 | 6 | Piran I | PiranI M1, 14-16, 16 |
| 2584 | 13005 | 2ul10-4ul12 | 6 | Piran I | PiranI M1, 14-16, 17 |
| 3143 | 13006 | 2ul10 | 6 | Piran I | PiranI M1, 14-16, 18 |
| 1854 | 13007 | 2ul10 | 6 | Piran I | PiranI M1, 14-16, 19 |
| 1789 | 13008 | 2ul10 | 6 | Piran I | PiranI M1, 14-16, 20 |
| 3086 | 13009 | 2ul10 | 6 | Piran I | PiranI M1, 14-16, 21 |
| 830 | 13010 | 2ul10 | 6 | Piran I | PiranI M1, 14-16, 22 |
| 3288 | 13011 | 2ul10 | 6 | Piran I | PiranI M1, 14-16, 23 |
| 4343 | 13012 | 2ul10 | 6 | Piran I | PiranI M1, 14-16, 24 |
| 3132 | 13013 | 2ul10 | 6 | Piran I | PiranI M1, 14-16, 25 |
| 1195 | 13014 | 2ul10 | 6 | Piran I | PiranI M1, 14-16, 26 |
| 1857 | 13015 | 2ul10 | 6 | Piran I | PiranI M1, 14-16, 27 |
| 3345 | 13016 | 2ul10 | 6 | Piran I | PiranI M1, 14-16, 28 |
| 3368 | 13017 | 2ul10 | 6 | Piran I | PiranI M1, 14-16, 29 |
| 2226 | 13018 | 2ul10 | 6 | Piran I | PiranI M1, 14-16, 30 |
| NA | 12405 | 2ul10 | 6 | PiranII grab5 | PiranII grab5-2 |
| NA | 12406 | 2ul10 | 6 | PiranII grab5 | PiranII grab5-7 |
| 3814 | 12844 | 2ul10 | 6 | Piran II | PiranII M53, 12-14, 01 |
| 3204 | 12845 | 4ul12 | 6 | Piran II | PiranII M53, 12-14, 02 |
| 2798 | 12846 | 2ul10 | 6 | Piran II | PiranII M53, 12-14, 03 |
| 2317 | 12847 | 2ul10 | 6 | Piran II | PiranII M53, 12-14, 04 |
| 537 | 12848 | 2ul10 | 6 | Piran II | PiranII M53, 12-14, 05 |
| 677 | 12849 | 2ul10 | 6 | Piran II | PiranII M53, 12-14, 06 |
| 1697 | 12852 | 2ul10 | 6 | Piran II | PiranII M53, 12-14, 09 |
| 3256 | 12853 | 2ul10 | 6 | Piran II | PiranII M53, 12-14, 10 |
| 3010 | 12854 | 2ul10 | 6 | Piran II | PiranII M53, 12-14, 11 |
| 2679 | 12855 | 2ul10 | 6 ent, t | Piran II | PiranII M53, 12-14, 12 |
| 1135 | 12856 | 2ul10 | 6 | Piran II | PiranII M53, 12-14, 13 |
| 1589 | 12857 | 2ul10 | 6.5 | Piran II | PiranII M53, 12-14, 14 |
| 3396 | 12858 | 2ul10 | 6 | Piran II | PiranII M53, 12-14, 15 |
| 1483 | 12859 | 2ul10 | 6 | Piran II | PiranII M53, 12-14, 16 |
| 3346 | 12860 | 2ul10 | 6 | Piran II | PiranII M53, 12-14, 17 |
| 3066 | 12861 | 2ul10 | 6 | Piran II | PiranII M53, 12-14, 18 |
| 4268 | 12862 | 2ul10 | 6 | Piran II | PiranII M53, 12-14, 19 |

| | | | | | | |
|---|---|---|---|---|---|---|
| 2745 | 12863 | 2ul10 | 6 | | Piran II | PiranII M53, 12-14, 20 |
| 2473 | 12864 | 2ul10 | 6 | | Piran II | PiranII M53, 12-14, 21 |
| 876 | 12865 | 2ul10 | 6 | | Piran II | PiranII M53, 12-14, 22 |
| 2883 | 12866 | 2ul10 | 6 | | Piran II | PiranII M53, 12-14, 23 |
| 1139 | 12867 | 2ul10 | 6 | | Piran II | PiranII M53, 12-14, 24 |
| 4873 | 12868 | 2ul10 | 6 | | Piran II | PiranII M53, 12-14, 25 |
| 1853 | 12869 | 2ul10 | 6 | | Piran II | PiranII M53, 12-14, 26 |
| 620 | 12870 | 2ul10 | 6 | | Piran II | PiranII M53, 12-14, 27 |
| 2928 | 12871 | 2ul10 | 6 | | Piran II | PiranII M53, 12-14, 28 |
| 1059 | 12872 | 2ul10 | 6.5 | | Piran II | PiranII M53, 12-14, 29 |
| 475 | 12873 | 2ul10 | 6 | | Piran II | PiranII M53, 12-14, 30 |
| 3062 | 12876 | 2ul10 | 6 | | Piran II | PiranII M53, 18-20, 03 |
| 4577 | 12877 | 2ul10 | 6 | | Piran II | PiranII M53, 18-20, 04 |
| 2379 | 12878 | 2ul10 | 6 | | Piran II | PiranII M53, 18-20, 05 |
| 2529 | 12879 | 2ul10 | 6 | | Piran II | PiranII M53, 18-20, 06 |
| 4283 | 12880 | 2ul10 | 6 | | Piran II | PiranII M53, 18-20, 07 |
| 3587 | 12881 | 2ul10 | 6 | | Piran II | PiranII M53, 18-20, 08 |
| 2618 | 12883 | 2ul10 | 6 | | Piran II | PiranII M53, 18-20, 10 |
| 4966 | 12885 | 2ul10 | 6 | | Piran II | PiranII M53, 18-20, 12 |
| 11006 | 12886 | 2ul10 | 6 | | Piran II | PiranII M53, 18-20, 13 |
| 6675 | 12888 | 2ul10 | 6.5 | | Piran II | PiranII M53, 18-20, 15 |
| 4921 | 12890 | 2ul10 | 6 | | Piran II | PiranII M53, 18-20, 17 |
| 1255 | 12892 | 2ul10 | 6 | | Piran II | PiranII M53, 18-20, 19 |
| 5473 | 12894 | 2ul10 | 6 | | Piran II | PiranII M53, 18-20, 21 |
| 3741 | 12895 | 2ul10 | 6 | | Piran II | PiranII M53, 18-20, 22 |
| 4221 | 12896 | 2ul10 | 6.5 | | Piran II | PiranII M53, 18-20, 23 |
| 5091 | 12897 | 2ul10 | 6.5 | | Piran II | PiranII M53, 18-20, 24 |
| 3145 | 12898 | 2ul10 | 6.5 | | Piran II | PiranII M53, 18-20, 25 |
| 3125 | 12899 | 2ul10 | 6.5 | | Piran II | PiranII M53, 18-20, 26 |
| 3547 | 12900 | 2ul10 | 6.5 | | Piran II | PiranII M53, 18-20, 27 |
| 3002 | 12901 | 2ul10 | 6.5 | | Piran II | PiranII M53, 18-20, 28 |
| 1338 | 12903 | 2ul10 | 6.5 | | Piran II | PiranII M53, 18-20, 30 |
| 2536 | 10782A | | 6 | | Piran II | PiranII M53-0-2-001 |
| 516 | 10782AA | | 6 | | Piran II | PiranII M53-0-2-027 |
| 1416 | 10782AB | | 6 | | Piran II | PiranII M53-0-2-028 |
| 1106 | 10782AC | | 6 | | Piran II | PiranII M53-0-2-029 |
| 1180 | 10782AD | | 6 | | Piran II | PiranII M53-0-2-030 |
| 1185 | 10782B | | 6 | | Piran II | PiranII M53-0-2-002 |
| 1279 | 10782C | | 6 | | Piran II | PiranII M53-0-2-003 |
| 1730 | 10782D | | 6 | | Piran II | PiranII M53-0-2-004 |
| 660 | 10782E | | 6 | | Piran II | PiranII M53-0-2-005 |
| 598 | 10782F | | 6 | | Piran II | PiranII M53-0-2-006 |
| 757 | 10782G | | 6 | | Piran II | PiranII M53-0-2-007 |
| 3815 | 10782H | | 6 | | Piran II | PiranII M53-0-2-008 |
| 2781 | 10782I | | 6 | | Piran II | PiranII M53-0-2-009 |
| 992 | 10782J | | 6 | | Piran II | PiranII M53-0-2-010 |
| 1038 | 10782K | | 6 | IS 10 | Piran II | PiranII M53-0-2-011 |
| 1770 | 10782K | | 6 | IS 10 | Piran II | PiranII M53-0-2-011 |
| 598 | 10782L | | 6 | | Piran II | PiranII M53-0-2-012 |
| 741 | 10782M | | 6 | | Piran II | PiranII M53-0-2-013 |
| 1027 | 10782N | | 6 | | Piran II | PiranII M53-0-2-014 |
| 403 | 10782O | | 6 | | Piran II | PiranII M53-0-2-015 |
| 954 | 10782P | | 6 | | Piran II | PiranII M53-0-2-016 |
| 1038 | 10782Q | | 6 | | Piran II | PiranII M53-0-2-017 |
| 308 | 10782R | | 6 | | Piran II | PiranII M53-0-2-018 |
| 345 | 10782S | | 6 | | Piran II | PiranII M53-0-2-019 |

| | | | | |
|---|---|---|---|---|
| 3335 | 10782T | 6 | Piran II | PiranII M53-0-2-020 |
| 1090 | 10782U | 6 | Piran II | PiranII M53-0-2-021 |
| 329 | 10782V | 6 | Piran II | PiranII M53-0-2-022 |
| 303 | 10782W | 6 | Piran II | PiranII M53-0-2-023 |
| 3212 | 10782X | 6 | Piran II | PiranII M53-0-2-024 |
| 1444 | 10782Y | 6 | Piran II | PiranII M53-0-2-025 |
| 162 | 10782Z | 6 | Piran II | PiranII M53-0-2-026 |
| 685 | 10783A | 6 | Piran II | PiranII M53-8-10-001 |
| 1696 | 10783AA | 6 | Piran II | PiranII M53-8-10-027 |
| 640 | 10783AB | 6 | Piran II | PiranII M53-8-10-028 |
| 819 | 10783AC | 6 | Piran II | PiranII M53-8-10-029 |
| 1552 | 10783AD | 6 | Piran II | PiranII M53-8-10-030 |
| 1816 | 10783B | 6 | Piran II | PiranII M53-8-10-002 |
| 1405 | 10783C | 6 | Piran II | PiranII M53-8-10-003 |
| 1412 | 10783D | 6 | Piran II | PiranII M53-8-10-004 |
| 3424 | 10783E | 6 | Piran II | PiranII M53-8-10-005 |
| 229 | 10783F | 6 | Piran II | PiranII M53-8-10-006 |
| 2934 | 10783G | 6 | Piran II | PiranII M53-8-10-007 |
| 1359 | 10783H | 6 | Piran II | PiranII M53-8-10-008 |
| 1359 | 10783I | 6 | Piran II | PiranII M53-8-10-009 |
| 1742 | 10783J | 6 | Piran II | PiranII M53-8-10-010 |
| 2126 | 10783L | 6 | Piran II | PiranII M53-8-10-012 |
| 2681 | 10783M | 6 | Piran II | PiranII M53-8-10-013 |
| 1467 | 10783N | 6 | Piran II | PiranII M53-8-10-014 |
| 1446 | 10783O | 6 | Piran II | PiranII M53-8-10-015 |
| 4183 | 10783P | 6 | Piran II | PiranII M53-8-10-016 |
| 2027 | 10783Q | 6 | Piran II | PiranII M53-8-10-017 |
| 1800 | 10783R | 6 | Piran II | PiranII M53-8-10-018 |
| 658 | 10783S | 6 | Piran II | PiranII M53-8-10-019 |
| 4224 | 10783T | 6 | Piran II | PiranII M53-8-10-020 |
| 311 | 10783U | 6 | Piran II | PiranII M53-8-10-021 |
| 7477 | 10783W | 6 | Piran II | PiranII M53-8-10-023 |
| 1584 | 10783X | 6 | Piran II | PiranII M53-8-10-024 |
| 746 | 10783Y | 6 | Piran II | PiranII M53-8-10-025 |
| 473 | 10783Z | 6 | Piran II | PiranII M53-8-10-026 |
| 3725 | 10784A | 6 | Piran II | PiranII M53-25-30-001 |
| 2828 | 10784AA | 6 | Piran II | PiranII M53-25-30-027 |
| 3425 | 10784AB | 6 | Piran II | PiranII M53-25-30-028 |
| 3973 | 10784AC | 6 | Piran II | PiranII M53-25-30-029 |
| 5828 | 10784AD | 6 | Piran II | PiranII M53-25-30-030 |
| 5428 | 10784B | 6 | Piran II | PiranII M53-25-30-002 |
| 4714 | 10784C | 6 | Piran II | PiranII M53-25-30-003 |
| 4981 | 10784D | 6 | Piran II | PiranII M53-25-30-004 |
| 2418 | 10784E | 6 | Piran II | PiranII M53-25-30-005 |
| 3066 | 10784F | 6 | Piran II | PiranII M53-25-30-006 |
| 3220 | 10784G | 6 | Piran II | PiranII M53-25-30-007 |
| 4975 | 10784H | 6 | Piran II | PiranII M53-25-30-008 |
| 3123 | 10784I | 6 | Piran II | PiranII M53-25-30-009 |
| 3713 | 10784J | 6 | Piran II | PiranII M53-25-30-010 |
| 3351 | 10784K | 6 | Piran II | PiranII M53-25-30-011 |
| 2362 | 10784L | 6 | Piran II | PiranII M53-25-30-012 |
| 3559 | 10784M | 6 | Piran II | PiranII M53-25-30-013 |
| 6000 | 10784N | 6 | Piran II | PiranII M53-25-30-014 |
| 3642 | 10784O | 6 | Piran II | PiranII M53-25-30-015 |
| 5080 | 10784P | 6 | Piran II | PiranII M53-25-30-016 |
| 4931 | 10784Q | 6 | Piran II | PiranII M53-25-30-017 |

| | | | | | |
|---|---|---|---|---|---|
| 2303 | 10784R | 6 | | Piran II | PiranII M53-25-30-018 |
| 4674 | 10784S | 6 | | Piran II | PiranII M53-25-30-019 |
| 6855 | 10784T | 6 | | Piran II | PiranII M53-25-30-020 |
| 6023 | 10784U | 6 | | Piran II | PiranII M53-25-30-021 |
| 6144 | 10784V | 6 | | Piran II | PiranII M53-25-30-022 |
| 2429 | 10784W | 6 | | Piran II | PiranII M53-25-30-023 |
| 4352 | 10784X | 6 | | Piran II | PiranII M53-25-30-024 |
| 3586 | 10784Y | 6 | | Piran II | PiranII M53-25-30-025 |
| 7079 | 10784Z | 6 | | Piran II | PiranII M53-25-30-026 |
| 5281 | 10785A | 6 | | Piran II | PiranII M53-45-50-001 |
| 3666 | 10785AA | 6 | | Piran II | PiranII M53-45-50-027 |
| 3718 | 10785AB | 6 | | Piran II | PiranII M53-45-50-028 |
| 3642 | 10785AC | 6 | | Piran II | PiranII M53-45-50-029 |
| 5527 | 10785AD | 6 | | Piran II | PiranII M53-45-50-030 |
| 2622 | 10785B | 6 | | Piran II | PiranII M53-45-50-002 |
| 3547 | 10785C | 6 | | Piran II | PiranII M53-45-50-003 |
| 3639 | 10785D | 6 | | Piran II | PiranII M53-45-50-004 |
| 4943 | 10785E | 6 | | Piran II | PiranII M53-45-50-005 |
| 3859 | 10785F | 6 | | Piran II | PiranII M53-45-50-006 |
| 2432 | 10785G | 6 | | Piran II | PiranII M53-45-50-007 |
| 3492 | 10785H | 6 | | Piran II | PiranII M53-45-50-008 |
| 4515 | 10785I | 6 | | Piran II | PiranII M53-45-50-009 |
| 2910 | 10785J | 6 | | Piran II | PiranII M53-45-50-010 |
| 4307 | 10785K | 6 | | Piran II | PiranII M53-45-50-011 |
| 4649 | 10785L | 6 | | Piran II | PiranII M53-45-50-012 |
| 5748 | 10785M | 6 | | Piran II | PiranII M53-45-50-013 |
| 3370 | 10785N | 6 | | Piran II | PiranII M53-45-50-014 |
| 4861 | 10785O | 6 | | Piran II | PiranII M53-45-50-015 |
| 9719 | 10785P | 6 | | Piran II | PiranII M53-45-50-016 |
| 7065 | 10785Q | 6 | | Piran II | PiranII M53-45-50-017 |
| 4940 | 10785R | 6 | | Piran II | PiranII M53-45-50-018 |
| 5724 | 10785S | 6 | | Piran II | PiranII M53-45-50-019 |
| 4612 | 10785T | 6 | | Piran II | PiranII M53-45-50-020 |
| 3828 | 10785U | 6 | | Piran II | PiranII M53-45-50-021 |
| 3851 | 10785V | 6 | | Piran II | PiranII M53-45-50-022 |
| 4424 | 10785W | 6 | | Piran II | PiranII M53-45-50-023 |
| 5610 | 10785X | 6 | | Piran II | PiranII M53-45-50-024 |
| 3729 | 10785Y | 6 | | Piran II | PiranII M53-45-50-025 |
| 3566 | 10785Z | 6 | | Piran II | PiranII M53-45-50-026 |
| 5384 | 10786A | 6 | | Piran II | PiranII M53-65-70-001 |
| 4233 | 10786AA | 6 | | Piran II | PiranII M53-65-70-027 |
| 6622 | 10786AB | 6 | | Piran II | PiranII M53-65-70-028 |
| 3088 | 10786AC | 6 | as 1( | Piran II | PiranII M53-65-70-029 |
| 3260 | 10786AD | 6 | | Piran II | PiranII M53-65-70-030 |
| 3113 | 10786B | 6 | | Piran II | PiranII M53-65-70-002 |
| 4036 | 10786C | 6 | | Piran II | PiranII M53-65-70-003 |
| 4873 | 10786D | 6 | | Piran II | PiranII M53-65-70-004 |
| 545 | 10786E | 6 | | Piran II | PiranII M53-65-70-005 |
| 5860 | 10786F | 6 | | Piran II | PiranII M53-65-70-006 |
| 3044 | 10786G | 6 | | Piran II | PiranII M53-65-70-007 |
| 416 | 10786H | 6 | | Piran II | PiranII M53-65-70-008 |
| 3841 | 10786I | 6 | | Piran II | PiranII M53-65-70-009 |
| 6066 | 10786J | 6 | | Piran II | PiranII M53-65-70-010 |
| 4858 | 10786K | 6 | | Piran II | PiranII M53-65-70-011 |
| 5295 | 10786L | 6 | | Piran II | PiranII M53-65-70-012 |
| 5774 | 10786M | 6 | | Piran II | PiranII M53-65-70-013 |

| | | | | | |
|---|---|---|---|---|---|
| 4880 | 10786N | 6 | | Piran II | PiranII M53-65-70-014 |
| 7198 | 10786O | 6 | | Piran II | PiranII M53-65-70-015 |
| 5102 | 10786P | 6 | | Piran II | PiranII M53-65-70-016 |
| 5291 | 10786Q | 6 | | Piran II | PiranII M53-65-70-017 |
| 2840 | 10786R | 6 | | Piran II | PiranII M53-65-70-018 |
| 3575 | 10786S | 6 | | Piran II | PiranII M53-65-70-019 |
| 6129 | 10786T | 6 | | Piran II | PiranII M53-65-70-020 |
| 846 | 10786U | 6 | | Piran II | PiranII M53-65-70-021 |
| 4821 | 10786V | 6 | | Piran II | PiranII M53-65-70-022 |
| 3788 | 10786W | 6 | | Piran II | PiranII M53-65-70-023 |
| 6797 | 10786X | 6 | | Piran II | PiranII M53-65-70-024 |
| 3930 | 10786Y | 6 | | Piran II | PiranII M53-65-70-025 |
| 3042 | 10786Z | 6 | s 107[illegible] | Piran II | PiranII M53-65-70-026 |
| 4822 | 10787A | 6 | | Piran II | PiranII M53-85-90-001 |
| 8685 | 10787AA | 6 | | Piran II | PiranII M53-85-90-027 |
| 6277 | 10787AB | 6 | | Piran II | PiranII M53-85-90-028 |
| 10402 | 10787AC | 6 | | Piran II | PiranII M53-85-90-029 |
| 6926 | 10787AD | 6 | | Piran II | PiranII M53-85-90-030 |
| 6762 | 10787B | 6 | | Piran II | PiranII M53-85-90-002 |
| 3401 | 10787C | 6 | | Piran II | PiranII M53-85-90-003 |
| 6844 | 10787D | 6 | | Piran II | PiranII M53-85-90-004 |
| 5863 | 10787E | 6 | | Piran II | PiranII M53-85-90-005 |
| 6016 | 10787F | 6 | | Piran II | PiranII M53-85-90-006 |
| 3173 | 10787G | 6 | | Piran II | PiranII M53-85-90-007 |
| 7518 | 10787H | 6 | | Piran II | PiranII M53-85-90-008 |
| 10494 | 10787I | 6 | | Piran II | PiranII M53-85-90-009 |
| 5593 | 10787J | 6 | | Piran II | PiranII M53-85-90-010 |
| 1251 | 10787K | 6 | | Piran II | PiranII M53-85-90-011 |
| 7271 | 10787L | 6 | | Piran II | PiranII M53-85-90-012 |
| 11502 | 10787M | 6 | | Piran II | PiranII M53-85-90-013 |
| 7779 | 10787N | 6 | | Piran II | PiranII M53-85-90-014 |
| 5069 | 10787O | 6 | | Piran II | PiranII M53-85-90-015 |
| 5404 | 10787P | 6 | | Piran II | PiranII M53-85-90-016 |
| 7063 | 10787Q | 6 | | Piran II | PiranII M53-85-90-017 |
| 7472 | 10787R | 6 | | Piran II | PiranII M53-85-90-018 |
| 2761 | 10787S | 6 | | Piran II | PiranII M53-85-90-019 |
| 4040 | 10787T | 6 | | Piran II | PiranII M53-85-90-020 |
| 1206 | 10787U | 6 | | Piran II | PiranII M53-85-90-021 |
| 7180 | 10787V | 6 | | Piran II | PiranII M53-85-90-022 |
| 6445 | 10787W | 6 | | Piran II | PiranII M53-85-90-023 |
| 5481 | 10787X | 6 | | Piran II | PiranII M53-85-90-024 |
| 7384 | 10787Y | 6 | | Piran II | PiranII M53-85-90-025 |
| 7310 | 10787Z | 6 | | Piran II | PiranII M53-85-90-026 |
| 4506 | 10788A | 6 | | Piran II | PiranII M53-100-105-001 |
| 6606 | 10788AA | 6 | | Piran II | PiranII M53-100-105-027 |
| 4900 | 10788AB | 6 | | Piran II | PiranII M53-100-105-028 |
| 5293 | 10788B | 6 | | Piran II | PiranII M53-100-105-002 |
| 7744 | 10788C | 6 | | Piran II | PiranII M53-100-105-003 |
| 8015 | 10788D | 6 | | Piran II | PiranII M53-100-105-004 |
| 3472 | 10788E | 6 | | Piran II | PiranII M53-100-105-005 |
| 5615 | 10788F | 6 | | Piran II | PiranII M53-100-105-006 |
| 2846 | 10788G | 6 | | Piran II | PiranII M53-100-105-007 |
| 6322 | 10788I | 6 | | Piran II | PiranII M53-100-105-009 |
| NA | 10788J | 6 | | Piran II | PiranII M53-100-105-010 |
| 6129 | 10788K | 6 | | Piran II | PiranII M53-100-105-011 |
| 2531 | 10788L | 6 | | Piran II | PiranII M53-100-105-012 |

| | | | | | |
|---|---|---|---|---|---|
| 4327 | 10788M | 6 | | Piran II | PiranII M53-100-105-013 |
| 4479 | 10788N | 6 | | Piran II | PiranII M53-100-105-014 |
| 7221 | 10788O | 6 | | Piran II | PiranII M53-100-105-015 |
| 3514 | 10788P | 6 | | Piran II | PiranII M53-100-105-016 |
| 5286 | 10788Q | 6 | | Piran II | PiranII M53-100-105-017 |
| 9471 | 10788R | 6 | | Piran II | PiranII M53-100-105-018 |
| 3383 | 10788S | 6 | | Piran II | PiranII M53-100-105-019 |
| 3246 | 10788T | 6 | | Piran II | PiranII M53-100-105-020 |
| 6612 | 10788U | 6 | | Piran II | PiranII M53-100-105-021 |
| 5613 | 10788V | 6 | | Piran II | PiranII M53-100-105-022 |
| 4844 | 10788W | 6 | | Piran II | PiranII M53-100-105-023 |
| 7645 | 10788X | 6 | | Piran II | PiranII M53-100-105-024 |
| 4994 | 10788Y | 6 | | Piran II | PiranII M53-100-105-025 |
| 4997 | 10788Z | 6 | | Piran II | PiranII M53-100-105-026 |
| 6675 | 10789A | 6 | | Piran II | PiranII M53-125-130-001 |
| 7696 | 10789AA | 6 | | Piran II | PiranII M53-125-130-027 |
| 3594 | 10789AB | 6 | | Piran II | PiranII M53-125-130-028 |
| 4718 | 10789AC | 6 | | Piran II | PiranII M53-125-130-029 |
| 5680 | 10789AD | 6 | | Piran II | PiranII M53-125-130-030 |
| 3564 | 10789B | 6 | | Piran II | PiranII M53-125-130-002 |
| 4722 | 10789C | 6 | | Piran II | PiranII M53-125-130-003 |
| 6603 | 10789D | 6 | | Piran II | PiranII M53-125-130-004 |
| 6025 | 10789E | 6 | | Piran II | PiranII M53-125-130-005 |
| 5815 | 10789F | 6 | | Piran II | PiranII M53-125-130-006 |
| 7860 | 10789G | 6 | | Piran II | PiranII M53-125-130-007 |
| 7828 | 10789H | 6 | | Piran II | PiranII M53-125-130-008 |
| 2769 | 10789I | 6 | | Piran II | PiranII M53-125-130-009 |
| 978 | 10789J | 6 | | Piran II | PiranII M53-125-130-010 |
| 5967 | 10789K | 6 | | Piran II | PiranII M53-125-130-011 |
| 4352 | 10789L | 6 | | Piran II | PiranII M53-125-130-012 |
| 3196 | 10789M | 6 | | Piran II | PiranII M53-125-130-013 |
| 3662 | 10789N | 6 | | Piran II | PiranII M53-125-130-014 |
| 6736 | 10789O | 6 | | Piran II | PiranII M53-125-130-015 |
| 5371 | 10789P | 6 | | Piran II | PiranII M53-125-130-016 |
| 5942 | 10789Q | 6 | | Piran II | PiranII M53-125-130-017 |
| 4938 | 10789R | 6 | | Piran II | PiranII M53-125-130-018 |
| 4510 | 10789S | 6 | LE F | Piran II | PiranII M53-125-130-019 |
| 7666 | 10789T | 6 | | Piran II | PiranII M53-125-130-020 |
| 4450 | 10789U | 6 | | Piran II | PiranII M53-125-130-021 |
| 6308 | 10789V | 6 | | Piran II | PiranII M53-125-130-022 |
| 4722 | 10789W | 6 | | Piran II | PiranII M53-125-130-023 |
| 5268 | 10789X | 6 | | Piran II | PiranII M53-125-130-024 |
| 1797 | 10789Y | 6 | | Piran II | PiranII M53-125-130-025 |
| 7873 | 10789Z | 6 | | Piran II | PiranII M53-125-130-026 |
| 4561 | 10790A | 6 | | Piran II | PiranII M53-145-150-001 |
| 1546 | 10790AA | 6 | | Piran II | PiranII M53-145-150-027 |
| 6068 | 10790AB | 6 | | Piran II | PiranII M53-145-150-028 |
| 4779 | 10790AC | 6 | | Piran II | PiranII M53-145-150-029 |
| 2448 | 10790AD | 6 | | Piran II | PiranII M53-145-150-030 |
| 1209 | 10790B | 6 | | Piran II | PiranII M53-145-150-002 |
| 2795 | 10790C | 6 | | Piran II | PiranII M53-145-150-003 |
| 4209 | 10790D | 6 | | Piran II | PiranII M53-145-150-004 |
| 4182 | 10790E | 6 | | Piran II | PiranII M53-145-150-005 |
| 5705 | 10790F | 6 | | Piran II | PiranII M53-145-150-006 |
| 3581 | 10790G | 6 | S ME | Piran II | PiranII M53-145-150-007 |
| 3733 | 10790H | 6 | | Piran II | PiranII M53-145-150-008 |

| | | | | | | |
|---|---|---|---|---|---|---|
| 4259 | 10790I | | 6 | | Piran II | PiranII M53-145-150-009 |
| 7367 | 10790J | | 6 | | Piran II | PiranII M53-145-150-010 |
| 5421 | 10790K | | 6 | | Piran II | PiranII M53-145-150-011 |
| 2394 | 10790L | | 6 | | Piran II | PiranII M53-145-150-012 |
| 4953 | 10790M | | 6 | | Piran II | PiranII M53-145-150-013 |
| 5733 | 10790N | | 6 | | Piran II | PiranII M53-145-150-014 |
| 4281 | 10790O | | 6 | | Piran II | PiranII M53-145-150-015 |
| 5481 | 10790P | | 6 | | Piran II | PiranII M53-145-150-016 |
| 4229 | 10790Q | | 6 | | Piran II | PiranII M53-145-150-017 |
| 3974 | 10790R | | 6 | | Piran II | PiranII M53-145-150-018 |
| 3950 | 10790S | | 6 | | Piran II | PiranII M53-145-150-019 |
| NA | 10790T | | 6 | | Piran II | PiranII M53-145-150-020 |
| 3577 | 10790U | | 6 | | Piran II | PiranII M53-145-150-021 |
| 5349 | 10790V | | 6 | | Piran II | PiranII M53-145-150-022 |
| 4420 | 10790W | | 6 | | Piran II | PiranII M53-145-150-023 |
| 2055 | 10790X | | 6 | | Piran II | PiranII M53-145-150-024 |
| 6950 | 10790Y | | 6 | | Piran II | PiranII M53-145-150-025 |
| 3444 | 10790Z | | 6 | | Piran II | PiranII M53-145-150-026 |
| 991 | 12893B | 2ul10 | 6 | ent, t | Piran II | PiranII M53, 18-20, 20 |
| 3406 | 12899B | 2ul10 | 6 | | Piran II | PiranII M53, 18-20, 26 |
| 2819 | 12902B | 2ul10 | 6 | ent, t | Piran II | PiranII M53, 18-20, 29 |

| depth (cm) | max. depth (cm) | 5 cm group (cm) | 10 cm group (cm) | Batch | taxon | Outlier | Contamination | Replicate | ageMedian | ageYng |
|---|---|---|---|---|---|---|---|---|---|---|
| 0-2 | 2 | 4 | 10 | 9 | Gouldia minima | | | average of repli | NA | NA |
| 0-2 | 2 | 4 | 10 | | Gouldia minima | | | | NA | NA |
| 0-2 | 2 | 4 | 10 | | Gouldia minima | | | | NA | NA |
| 0-2 | 2 | 4 | 10 | | Gouldia minima | | | | NA | NA |
| 0-2 | 2 | 4 | 10 | final | Gouldia minima | | | | NA | NA |
| 0-2 | 2 | 4 | 10 | 10 | Gouldia minima | | | | NA | NA |
| 0-2 | 2 | 4 | 10 | 9 | Gouldia minima | | | | NA | NA |
| 0-2 | 2 | 4 | 10 | 10 | Gouldia minima | | | | NA | NA |
| 0-2 | 2 | 4 | 10 | final | Gouldia minima | | | | NA | NA |
| 0-2 | 2 | 4 | 10 | 10 | Gouldia minima | | | | NA | NA |
| 0-2 | 2 | 4 | 10 | | Gouldia minima | | | | NA | NA |
| 0-2 | 2 | 4 | 10 | | Gouldia minima | | | | NA | NA |
| 0-2 | 2 | 4 | 10 | 10 | Gouldia minima | | | | NA | NA |
| 0-2 | 2 | 4 | 10 | 10 | Gouldia minima | | | | NA | NA |
| 0-2 | 2 | 4 | 10 | final | Gouldia minima | | | | NA | NA |
| 0-2 | 2 | 4 | 10 | 9 | Gouldia minima | | | | NA | NA |
| 0-2 | 2 | 4 | 10 | 9 | Gouldia minima | | | | NA | NA |
| 0-2 | 2 | 4 | 10 | 9 | Gouldia minima | | | | NA | NA |
| 0-2 | 2 | 4 | 10 | 9 | Gouldia minima | | | | NA | NA |
| 0-2 | 2 | 4 | 10 | 10 | Gouldia minima | | | | NA | NA |
| 0-2 | 2 | 4 | 10 | | Gouldia minima | | | | NA | NA |
| 0-2 | 2 | 4 | 10 | 10 | Gouldia minima | | | | NA | NA |
| 0-2 | 2 | 4 | 10 | 10 | Gouldia minima | | | | NA | NA |
| 0-2 | 2 | 4 | 10 | 9 | Gouldia minima | outlier | outlier in conc plot | | NA | NA |
| 0-2 | 2 | 4 | 10 | 9 | Gouldia minima | | | | NA | NA |
| 0-2 | 2 | 4 | 10 | | Gouldia minima | | | | 332 | 164 |
| 0-2 | 2 | 4 | 10 | 9 | Gouldia minima | | | | NA | NA |
| 0-2 | 2 | 4 | 10 | | Gouldia minima | | | | NA | NA |
| 0-2 | 2 | 4 | 10 | 9 | Gouldia minima | | | | NA | NA |
| 0-2 | 2 | 4 | 10 | 9 | Gouldia minima | | | | NA | NA |
| 8-10 | 10 | 12 | 10 | | Gouldia minima | | | | NA | NA |
| 8-10 | 10 | 12 | 10 | | Gouldia minima | | | | 239 | 104 |
| 8-10 | 10 | 12 | 10 | 10 | Gouldia minima | | | | NA | NA |
| 8-10 | 10 | 12 | 10 | 9 | Gouldia minima | | | | NA | NA |
| 8-10 | 10 | 12 | 10 | 9 | Gouldia minima | | | | NA | NA |
| 8-10 | 10 | 12 | 10 | 9 | Gouldia minima | | | | NA | NA |
| 8-10 | 10 | 12 | 10 | 9 | Gouldia minima | | | | NA | NA |
| 8-10 | 10 | 12 | 10 | 10 | Gouldia minima | | | | NA | NA |
| 8-10 | 10 | 12 | 10 | final | Gouldia minima | | | | NA | NA |
| 8-10 | 10 | 12 | 10 | 9 | Gouldia minima | | | | NA | NA |
| 8-10 | 10 | 12 | 10 | 10 | Gouldia minima | | | | NA | NA |
| 8-10 | 10 | 12 | 10 | 9 | Gouldia minima | | | | NA | NA |
| 8-10 | 10 | 12 | 10 | 9 | Gouldia minima | | | | NA | NA |
| 8-10 | 10 | 12 | 10 | 9 | Gouldia minima | | | | NA | NA |
| 8-10 | 10 | 12 | 10 | 10 | Gouldia minima | | | | NA | NA |
| 8-10 | 10 | 12 | 10 | | Gouldia minima | | | | NA | NA |
| 8-10 | 10 | 12 | 10 | 9 | Gouldia minima | | | | NA | NA |
| 8-10 | 10 | 12 | 10 | 9 | Gouldia minima | | | | NA | NA |
| 8-10 | 10 | 12 | 10 | 9 | Gouldia minima | | | | NA | NA |
| 8-10 | 10 | 12 | 10 | 9 | Gouldia minima | | | | NA | NA |

| | | | | | | | | | |
|---|---|---|---|---|---|---|---|---|---|
| 8-10 | 10 | 12 | 10 | | Gouldia minima | | | NA | NA |
| 8-10 | 10 | 12 | 10 | 9 | Gouldia minima | | | NA | NA |
| 8-10 | 10 | 12 | 10 | 10 | Gouldia minima | | | NA | NA |
| 8-10 | 10 | 12 | 10 | | Gouldia minima | | | NA | NA |
| 8-10 | 10 | 12 | 10 | 9 | Gouldia minima | | | NA | NA |
| 8-10 | 10 | 12 | 10 | final | Gouldia minima | | | NA | NA |
| 8-10 | 10 | 12 | 10 | 10 | Gouldia minima | | | NA | NA |
| 8-10 | 10 | 12 | 10 | 9 | Gouldia minima | | | NA | NA |
| 8-10 | 10 | 12 | 10 | | Gouldia minima | | | NA | NA |
| 8-10 | 10 | 12 | 10 | 10 | Gouldia minima | | | NA | NA |
| 20-25 | 25 | 25 | 30 | | Gouldia minima | | | NA | NA |
| 20-25 | 25 | 25 | 30 | | Gouldia minima | | | 4467 | 4281 |
| 20-25 | 25 | 25 | 30 | | Gouldia minima | | | NA | NA |
| 20-25 | 25 | 25 | 30 | | Gouldia minima | | | 4548 | 4362 |
| 20-25 | 25 | 25 | 30 | | Gouldia minima | outlier | outlier in DL plot | NA | NA |
| 20-25 | 25 | 25 | 30 | 10 | Gouldia minima | | | NA | NA |
| 20-25 | 25 | 25 | 30 | 10 | Gouldia minima | | | NA | NA |
| 20-25 | 25 | 25 | 30 | 10 | Gouldia minima | | | NA | NA |
| 20-25 | 25 | 25 | 30 | | Gouldia minima | | | NA | NA |
| 20-25 | 25 | 25 | 30 | | Gouldia minima | | | 2846 | 2746 |
| 20-25 | 25 | 25 | 30 | | Gouldia minima | | | NA | NA |
| 20-25 | 25 | 25 | 30 | | Gouldia minima | | | NA | NA |
| 20-25 | 25 | 25 | 30 | | Gouldia minima | | | NA | NA |
| 20-25 | 25 | 25 | 30 | | Gouldia minima | | | NA | NA |
| 20-25 | 25 | 25 | 30 | 9 | Gouldia minima | | | NA | NA |
| 20-25 | 25 | 25 | 30 | final | Gouldia minima | | | NA | NA |
| 20-25 | 25 | 25 | 30 | 9 | Gouldia minima | | | NA | NA |
| 20-25 | 25 | 25 | 30 | | Gouldia minima | | | NA | NA |
| 20-25 | 25 | 25 | 30 | 10 | Gouldia minima | outlier | outlier in DL plot | NA | NA |
| 20-25 | 25 | 25 | 30 | 10 | Gouldia minima | | | NA | NA |
| 20-25 | 25 | 25 | 30 | 10 | Gouldia minima | | | NA | NA |
| 20-25 | 25 | 25 | 30 | 10 | Gouldia minima | | | NA | NA |
| 20-25 | 25 | 25 | 30 | 9 | Gouldia minima | | | NA | NA |
| 20-25 | 25 | 25 | 30 | 9 | Gouldia minima | | | NA | NA |
| 20-25 | 25 | 25 | 30 | 10 | Gouldia minima | | | NA | NA |
| 20-25 | 25 | 25 | 30 | 9 | Gouldia minima | | | NA | NA |
| 20-25 | 25 | 25 | 30 | 9 | Gouldia minima | | | NA | NA |
| 20-25 | 25 | 25 | 30 | 9 | Gouldia minima | | | NA | NA |
| 20-25 | 25 | 25 | 30 | 10 | Gouldia minima | | | NA | NA |
| 40-45 | 45 | 45 | 50 | | Gouldia minima | | | 4663 | 4493 |
| 40-45 | 45 | 45 | 50 | | Gouldia minima | | | NA | NA |
| 40-45 | 45 | 45 | 50 | 10 | Gouldia minima | | | NA | NA |
| 40-45 | 45 | 45 | 50 | | Gouldia minima | | | NA | NA |
| 40-45 | 45 | 45 | 50 | | Gouldia minima | | | NA | NA |
| 40-45 | 45 | 45 | 50 | | Gouldia minima | | | NA | NA |
| 40-45 | 45 | 45 | 50 | 9 | Gouldia minima | | | NA | NA |
| 40-45 | 45 | 45 | 50 | | Gouldia minima | | | 6031 | 5857 |
| 40-45 | 45 | 45 | 50 | | Gouldia minima | | | NA | NA |
| 40-45 | 45 | 45 | 50 | 10 | Gouldia minima | | | NA | NA |
| 40-45 | 45 | 45 | 50 | 10 | Gouldia minima | | | NA | NA |
| 40-45 | 45 | 45 | 50 | 10 | Gouldia minima | | | NA | NA |
| 40-45 | 45 | 45 | 50 | | Gouldia minima | | | NA | NA |
| 40-45 | 45 | 45 | 50 | 10 | Gouldia minima | | | NA | NA |
| 40-45 | 45 | 45 | 50 | 9 | Gouldia minima | | | NA | NA |
| 40-45 | 45 | 45 | 50 | 9 | Gouldia minima | | | NA | NA |
| 40-45 | 45 | 45 | 50 | 9 | Gouldia minima | | | NA | NA |

| | | | | | | | | | |
|---|---|---|---|---|---|---|---|---|---|
| 40-45 | 45 | 45 | 50 | 10 | Gouldia minima | | | NA | NA |
| 40-45 | 45 | 45 | 50 | final | Gouldia minima | | | NA | NA |
| 40-45 | 45 | 45 | 50 | 9 | Gouldia minima | | | NA | NA |
| 40-45 | 45 | 45 | 50 | | Gouldia minima | | | NA | NA |
| 40-45 | 45 | 45 | 50 | 10 | Gouldia minima | | | NA | NA |
| 40-45 | 45 | 45 | 50 | 9 | Gouldia minima | | | NA | NA |
| 40-45 | 45 | 45 | 50 | final | Gouldia minima | | | NA | NA |
| 40-45 | 45 | 45 | 50 | | Gouldia minima | | average of repli | NA | NA |
| 40-45 | 45 | 45 | 50 | 9 | Gouldia minima | | | NA | NA |
| 40-45 | 45 | 45 | 50 | 9 | Gouldia minima | | | NA | NA |
| 40-45 | 45 | 45 | 50 | 10 | Gouldia minima | | | NA | NA |
| 40-45 | 45 | 45 | 50 | 10 | Gouldia minima | | | NA | NA |
| 40-45 | 45 | 45 | 50 | final | Gouldia minima | | | NA | NA |
| 60-65 | 65 | 65 | 70 | | Gouldia minima | | | NA | NA |
| 60-65 | 65 | 65 | 70 | | Gouldia minima | | | 4604 | 4456 |
| 60-65 | 65 | 65 | 70 | 9 | Gouldia minima | | | NA | NA |
| 60-65 | 65 | 65 | 70 | 9 | Gouldia minima | | | NA | NA |
| 60-65 | 65 | 65 | 70 | | Gouldia minima | | | 3147 | 2970 |
| 60-65 | 65 | 65 | 70 | 9 | Gouldia minima | | | NA | NA |
| 60-65 | 65 | 65 | 70 | 9 | Gouldia minima | | | NA | NA |
| 60-65 | 65 | 65 | 70 | 9 | Gouldia minima | | | NA | NA |
| 60-65 | 65 | 65 | 70 | final | Gouldia minima | | | NA | NA |
| 60-65 | 65 | 65 | 70 | 9 | Gouldia minima | | | NA | NA |
| 60-65 | 65 | 65 | 70 | 10 | Gouldia minima | | | NA | NA |
| 60-65 | 65 | 65 | 70 | 10 | Gouldia minima | | | NA | NA |
| 60-65 | 65 | 65 | 70 | final | Gouldia minima | | | NA | NA |
| 60-65 | 65 | 65 | 70 | 9 | Gouldia minima | | | NA | NA |
| 60-65 | 65 | 65 | 70 | 10 | Gouldia minima | | | NA | NA |
| 60-65 | 65 | 65 | 70 | 9 | Gouldia minima | | | NA | NA |
| 60-65 | 65 | 65 | 70 | 9 | Gouldia minima | | | NA | NA |
| 60-65 | 65 | 65 | 70 | final | Gouldia minima | | | NA | NA |
| 60-65 | 65 | 65 | 70 | 9 | Gouldia minima | outlier | outlier in DL plot | NA | NA |
| 60-65 | 65 | 65 | 70 | final | Gouldia minima | | | NA | NA |
| 60-65 | 65 | 65 | 70 | | Gouldia minima | | | NA | NA |
| 60-65 | 65 | 65 | 70 | 10 | Gouldia minima | | | NA | NA |
| 60-65 | 65 | 65 | 70 | final | Gouldia minima | | | NA | NA |
| 60-65 | 65 | 65 | 70 | 9 | Gouldia minima | | | NA | NA |
| 60-65 | 65 | 65 | 70 | 9 | Gouldia minima | | | NA | NA |
| 60-65 | 65 | 65 | 70 | final | Gouldia minima | | | NA | NA |
| 60-65 | 65 | 65 | 70 | 10 | Gouldia minima | | | NA | NA |
| 60-65 | 65 | 65 | 70 | 10 | Gouldia minima | | | NA | NA |
| 60-65 | 65 | 65 | 70 | 10 | Gouldia minima | | | NA | NA |
| 60-65 | 65 | 65 | 70 | 10 | Gouldia minima | | | NA | NA |
| 85-90 | 90 | 90 | 90 | final | Gouldia minima | | | NA | NA |
| 85-90 | 90 | 90 | 90 | 10 | Gouldia minima | | | NA | NA |
| 85-90 | 90 | 90 | 90 | 10 | Gouldia minima | | | NA | NA |
| 85-90 | 90 | 90 | 90 | 9 | Gouldia minima | | | NA | NA |
| 85-90 | 90 | 90 | 90 | 10 | Gouldia minima | | | NA | NA |
| 85-90 | 90 | 90 | 90 | 10 | Gouldia minima | | | NA | NA |
| 85-90 | 90 | 90 | 90 | 10 | Gouldia minima | | | NA | NA |
| 85-90 | 90 | 90 | 90 | 10 | Gouldia minima | | | NA | NA |
| 85-90 | 90 | 90 | 90 | 10 | Gouldia minima | | | NA | NA |
| 85-90 | 90 | 90 | 90 | 10 | Gouldia minima | | | NA | NA |
| 85-90 | 90 | 90 | 90 | 10 | Gouldia minima | | | NA | NA |
| 85-90 | 90 | 90 | 90 | final | Gouldia minima | | | NA | NA |
| 85-90 | 90 | 90 | 90 | 10 | Gouldia minima | | | NA | NA |

| | | | | | | | | | |
|---|---|---|---|---|---|---|---|---|---|
| 85-90 | 90 | 90 | 90 | 10 | Gouldia minima | | | NA | NA |
| 85-90 | 90 | 90 | 90 | 9 | Gouldia minima | outlier | outlier in DL plot | NA | NA |
| 85-90 | 90 | 90 | 90 | 9 | Gouldia minima | | | NA | NA |
| 85-90 | 90 | 90 | 90 | 9 | Gouldia minima | | | NA | NA |
| 85-90 | 90 | 90 | 90 | 9 | Gouldia minima | | | NA | NA |
| 85-90 | 90 | 90 | 90 | 9 | Gouldia minima | | | NA | NA |
| 85-90 | 90 | 90 | 90 | 9 | Gouldia minima | | | NA | NA |
| 85-90 | 90 | 90 | 90 | 9 | Gouldia minima | | | NA | NA |
| 85-90 | 90 | 90 | 90 | 9 | Gouldia minima | | | NA | NA |
| 85-90 | 90 | 90 | 90 | 9 | Gouldia minima | | | NA | NA |
| 110-115 | 115 | 115 | 120 | final | Gouldia minima | | | NA | NA |
| 110-115 | 115 | 115 | 120 | | Gouldia minima | | | NA | NA |
| 110-115 | 115 | 115 | 120 | 10 | Gouldia minima | | | NA | NA |
| 110-115 | 115 | 115 | 120 | 10 | Gouldia minima | | | NA | NA |
| 110-115 | 115 | 115 | 120 | 10 | Gouldia minima | | | NA | NA |
| 110-115 | 115 | 115 | 120 | 10 | Gouldia minima | | | NA | NA |
| 110-115 | 115 | 115 | 120 | 10 | Gouldia minima | | | NA | NA |
| 110-115 | 115 | 115 | 120 | 10 | Gouldia minima | | | NA | NA |
| 110-115 | 115 | 115 | 120 | 10 | Gouldia minima | | | NA | NA |
| 110-115 | 115 | 115 | 120 | 10 | Gouldia minima | | | NA | NA |
| 110-115 | 115 | 115 | 120 | 10 | Gouldia minima | | | NA | NA |
| 110-115 | 115 | 115 | 120 | 9 | Gouldia minima | | | NA | NA |
| 110-115 | 115 | 115 | 120 | 10 | Gouldia minima | | | NA | NA |
| 110-115 | 115 | 115 | 120 | | Gouldia minima | | | NA | NA |
| 110-115 | 115 | 115 | 120 | final | Gouldia minima | | | NA | NA |
| 110-115 | 115 | 115 | 120 | 10 | Gouldia minima | | | NA | NA |
| 110-115 | 115 | 115 | 120 | final | Gouldia minima | | | NA | NA |
| 110-115 | 115 | 115 | 120 | | Gouldia minima | | | NA | NA |
| 110-115 | 115 | 115 | 120 | 10 | Gouldia minima | | | NA | NA |
| 110-115 | 115 | 115 | 120 | 10 | Gouldia minima | | | NA | NA |
| 110-115 | 115 | 115 | 120 | final | Gouldia minima | | | NA | NA |
| 110-115 | 115 | 115 | 120 | final | Gouldia minima | | | NA | NA |
| 110-115 | 115 | 115 | 120 | | Gouldia minima | | | NA | NA |
| 135-140 | 140 | 140 | 140 | | Gouldia minima | | | NA | NA |
| 135-140 | 140 | 140 | 140 | final | Gouldia minima | | | NA | NA |
| 135-140 | 140 | 140 | 140 | 10 | Gouldia minima | | | NA | NA |
| 135-140 | 140 | 140 | 140 | final | Gouldia minima | | | NA | NA |
| 135-140 | 140 | 140 | 140 | final | Gouldia minima | | | NA | NA |
| 135-140 | 140 | 140 | 140 | final | Gouldia minima | | | NA | NA |
| 135-140 | 140 | 140 | 140 | final | Gouldia minima | | | NA | NA |
| 135-140 | 140 | 140 | 140 | final | Gouldia minima | | | NA | NA |
| 135-140 | 140 | 140 | 140 | 10 | Gouldia minima | | | NA | NA |
| 135-140 | 140 | 140 | 140 | 10 | Gouldia minima | | | NA | NA |
| 135-140 | 140 | 140 | 140 | final | Gouldia minima | | | NA | NA |
| 135-140 | 140 | 140 | 140 | 10 | Gouldia minima | | | NA | NA |
| 135-140 | 140 | 140 | 140 | final | Gouldia minima | | | NA | NA |
| 135-140 | 140 | 140 | 140 | 10 | Gouldia minima | | | NA | NA |
| 135-140 | 140 | 140 | 140 | final | Gouldia minima | | | NA | NA |
| 135-140 | 140 | 140 | 140 | | Gouldia minima | | | NA | NA |
| 135-140 | 140 | 140 | 140 | | Gouldia minima | | | NA | NA |
| 135-140 | 140 | 140 | 140 | 10 | Gouldia minima | | | NA | NA |
| 135-140 | 140 | 140 | 140 | 10 | Gouldia minima | | | NA | NA |
| 135-140 | 140 | 140 | 140 | 10 | Gouldia minima | | | NA | NA |
| 135-140 | 140 | 140 | 140 | final | Gouldia minima | | | NA | NA |
| 135-140 | 140 | 140 | 140 | 10 | Gouldia minima | | | NA | NA |
| 135-140 | 140 | 140 | 140 | 10 | Gouldia minima | | | NA | NA |

| | | | | | | | | | | |
|---|---|---|---|---|---|---|---|---|---|---|
| 135-140 | 140 | 140 | 140 | | Gouldia minima | | | | NA | NA |
| 135-140 | 140 | 140 | 140 | | Gouldia minima | | | | NA | NA |
| 135-140 | 140 | 140 | 140 | final | Gouldia minima | | | | NA | NA |
| 135-140 | 140 | 140 | 140 | | Gouldia minima | outlier | outlier in DL plot | | NA | NA |
| 135-140 | 140 | 140 | 140 | | Gouldia minima | | | | NA | NA |
| 135-140 | 140 | 140 | 140 | | Gouldia minima | | | | NA | NA |
| 135-140 | 140 | 140 | 140 | 10 | Gouldia minima | | | | NA | NA |
| 14-16 | 16 | 16 | 20 | new | Gouldia minima | | | | NA | NA |
| 14-16 | 16 | 16 | 20 | new | Gouldia minima | | | | NA | NA |
| 14-16 | 16 | 16 | 20 | new | Gouldia minima | | | | NA | NA |
| 14-16 | 16 | 16 | 20 | new | Gouldia minima | | | | NA | NA |
| 14-16 | 16 | 16 | 20 | new | Gouldia minima | | | | NA | NA |
| 14-16 | 16 | 16 | 20 | new | Gouldia minima | | | | NA | NA |
| 14-16 | 16 | 16 | 20 | new | Gouldia minima | | | | NA | NA |
| 14-16 | 16 | 16 | 20 | new | Gouldia minima | | | | NA | NA |
| 14-16 | 16 | 16 | 20 | new | Gouldia minima | | | | NA | NA |
| 14-16 | 16 | 16 | 20 | new | Gouldia minima | | | | NA | NA |
| 14-16 | 16 | 16 | 20 | new | Gouldia minima | | | | NA | NA |
| 14-16 | 16 | 16 | 20 | new | Gouldia minima | | | | NA | NA |
| 14-16 | 16 | 16 | 20 | new | Gouldia minima | | | | NA | NA |
| 14-16 | 16 | 16 | 20 | new | Gouldia minima | | | | NA | NA |
| 14-16 | 16 | 16 | 20 | new | Gouldia minima | outlier | outlier in conc plot | | NA | NA |
| 14-16 | 16 | 16 | 20 | new | Gouldia minima | | | | NA | NA |
| 14-16 | 16 | 16 | 20 | new | Gouldia minima | | | average of repli | NA | NA |
| 14-16 | 16 | 16 | 20 | new | Gouldia minima | | | | NA | NA |
| 14-16 | 16 | 16 | 20 | new | Gouldia minima | | | | NA | NA |
| 14-16 | 16 | 16 | 20 | new | Gouldia minima | | | | NA | NA |
| 14-16 | 16 | 16 | 20 | new | Gouldia minima | | | | NA | NA |
| 14-16 | 16 | 16 | 20 | new | Gouldia minima | | | | NA | NA |
| 14-16 | 16 | 16 | 20 | new | Gouldia minima | | | | NA | NA |
| 14-16 | 16 | 16 | 20 | new | Gouldia minima | | | | NA | NA |
| 14-16 | 16 | 16 | 20 | new | Gouldia minima | | | | NA | NA |
| 14-16 | 16 | 16 | 20 | new | Gouldia minima | | | | NA | NA |
| 14-16 | 16 | 16 | 20 | new | Gouldia minima | | | | NA | NA |
| 14-16 | 16 | 16 | 20 | new | Gouldia minima | | | | NA | NA |
| 14-16 | 16 | 16 | 20 | new | Gouldia minima | | | | NA | NA |
| 14-16 | 16 | 16 | 20 | new | Gouldia minima | | | | NA | NA |
| live | live | live | live | 3 | Gouldia minima | | | | 1 | 0.5 |
| live | live | live | live | 3 | Gouldia minima | | high Serine | | 1 | 0.5 |
| 12-14 | 14 | 16 | 20 | | Gouldia minima | | | | NA | NA |
| 12-14 | 14 | 16 | 20 | | Gouldia minima | | | | NA | NA |
| 12-14 | 14 | 16 | 20 | | Gouldia minima | | | | NA | NA |
| 12-14 | 14 | 16 | 20 | | Gouldia minima | | | | NA | NA |
| 12-14 | 14 | 16 | 20 | | Gouldia minima | | | | NA | NA |
| 12-14 | 14 | 16 | 20 | | Gouldia minima | | | | NA | NA |
| 12-14 | 14 | 16 | 20 | | Gouldia minima | | | | NA | NA |
| 12-14 | 14 | 16 | 20 | | Gouldia minima | | | | NA | NA |
| 12-14 | 14 | 16 | 20 | | Gouldia minima | | | | NA | NA |
| 12-14 | 14 | 16 | 20 | | Gouldia minima | | | | NA | NA |
| 12-14 | 14 | 16 | 20 | | Gouldia minima | | | | NA | NA |
| 12-14 | 14 | 16 | 20 | | Gouldia minima | | | | NA | NA |
| 12-14 | 14 | 16 | 20 | | Gouldia minima | | | | NA | NA |
| 12-14 | 14 | 16 | 20 | | Gouldia minima | | | | NA | NA |
| 12-14 | 14 | 16 | 20 | | Gouldia minima | | | | NA | NA |
| 12-14 | 14 | 16 | 20 | | Gouldia minima | | | | NA | NA |
| 12-14 | 14 | 16 | 20 | | Gouldia minima | | | | NA | NA |

| | | | | | | | |
|---|---|---|---|---|---|---|---|
| 12-14 | 14 | 16 | 20 | | Gouldia minima | NA | NA |
| 12-14 | 14 | 16 | 20 | | Gouldia minima | NA | NA |
| 12-14 | 14 | 16 | 20 | | Gouldia minima | NA | NA |
| 12-14 | 14 | 16 | 20 | | Gouldia minima | NA | NA |
| 12-14 | 14 | 16 | 20 | | Gouldia minima | NA | NA |
| 12-14 | 14 | 16 | 20 | | Gouldia minima | NA | NA |
| 12-14 | 14 | 16 | 20 | | Gouldia minima | NA | NA |
| 12-14 | 14 | 16 | 20 | | Gouldia minima | NA | NA |
| 12-14 | 14 | 16 | 20 | | Gouldia minima | NA | NA |
| 12-14 | 14 | 16 | 20 | | Gouldia minima | NA | NA |
| 12-14 | 14 | 16 | 20 | | Gouldia minima | NA | NA |
| 18-20 | 20 | 20 | 20 | | Gouldia minima | NA | NA |
| 18-20 | 20 | 20 | 20 | | Gouldia minima | NA | NA |
| 18-20 | 20 | 20 | 20 | | Gouldia minima | NA | NA |
| 18-20 | 20 | 20 | 20 | | Gouldia minima | NA | NA |
| 18-20 | 20 | 20 | 20 | | Gouldia minima | NA | NA |
| 18-20 | 20 | 20 | 20 | | Gouldia minima | NA | NA |
| 18-20 | 20 | 20 | 20 | | Gouldia minima | NA | NA |
| 18-20 | 20 | 20 | 20 | | Gouldia minima | NA | NA |
| 18-20 | 20 | 20 | 20 | | Gouldia minima | NA | NA |
| 18-20 | 20 | 20 | 20 | | Gouldia minima | NA | NA |
| 18-20 | 20 | 20 | 20 | | Gouldia minima | NA | NA |
| 18-20 | 20 | 20 | 20 | | Gouldia minima | NA | NA |
| 18-20 | 20 | 20 | 20 | | Gouldia minima | NA | NA |
| 18-20 | 20 | 20 | 20 | | Gouldia minima | NA | NA |
| 18-20 | 20 | 20 | 20 | | Gouldia minima | NA | NA |
| 18-20 | 20 | 20 | 20 | | Gouldia minima | NA | NA |
| 18-20 | 20 | 20 | 20 | | Gouldia minima | NA | NA |
| 18-20 | 20 | 20 | 20 | | Gouldia minima | NA | NA |
| 18-20 | 20 | 20 | 20 | | Gouldia minima | NA | NA |
| 18-20 | 20 | 20 | 20 | | Gouldia minima | NA | NA |
| 18-20 | 20 | 20 | 20 | | Gouldia minima | NA | NA |
| 0-2 | 2 | 4 | 10 | 5 | Gouldia minima | NA | NA |
| 0-2 | 2 | 4 | 10 | 4 | Gouldia minima | NA | NA |
| 0-2 | 2 | 4 | 10 | 4 | Gouldia minima | NA | NA |
| 0-2 | 2 | 4 | 10 | 4 | Gouldia minima | NA | NA |
| 0-2 | 2 | 4 | 10 | 4 | Gouldia minima | NA | NA |
| 0-2 | 2 | 4 | 10 | 3 | Gouldia minima | NA | NA |
| 0-2 | 2 | 4 | 10 | 4 | Gouldia minima | NA | NA |
| 0-2 | 2 | 4 | 10 | 4 | Gouldia minima | NA | NA |
| 0-2 | 2 | 4 | 10 | 4 | Gouldia minima | NA | NA |
| 0-2 | 2 | 4 | 10 | 3 | Gouldia minima | NA | NA |
| 0-2 | 2 | 4 | 10 | 4 | Gouldia minima | NA | NA |
| 0-2 | 2 | 4 | 10 | 4 | Gouldia minima | NA | NA |
| 0-2 | 2 | 4 | 10 | 4 | Gouldia minima | NA | NA |
| 0-2 | 2 | 4 | 10 | 4 | Gouldia minima | NA | NA |
| 0-2 | 2 | 4 | 10 | | Gouldia minima | NA | NA |
| 0-2 | 2 | 4 | 10 | | Gouldia minima | NA | NA |
| 0-2 | 2 | 4 | 10 | 4 | Gouldia minima | NA | NA |
| 0-2 | 2 | 4 | 10 | 4 | Gouldia minima | NA | NA |
| 0-2 | 2 | 4 | 10 | 4 | Gouldia minima | NA | NA |
| 0-2 | 2 | 4 | 10 | 4 | Gouldia minima | NA | NA |
| 0-2 | 2 | 4 | 10 | 4 | Gouldia minima | NA | NA |
| 0-2 | 2 | 4 | 10 | 4 | Gouldia minima | NA | NA |
| 0-2 | 2 | 4 | 10 | 4 | Gouldia minima | NA | NA |
| 0-2 | 2 | 4 | 10 | 4 | Gouldia minima | NA | NA |

| | | | | | | | |
|---|---|---|---|---|---|---|---|
| 0-2 | 2 | 4 | 10 | 4 | Gouldia minima | NA | NA |
| 0-2 | 2 | 4 | 10 | 4 | Gouldia minima | NA | NA |
| 0-2 | 2 | 4 | 10 | 4 | Gouldia minima | NA | NA |
| 0-2 | 2 | 4 | 10 | 4 | Gouldia minima | NA | NA |
| 0-2 | 2 | 4 | 10 | 4 | Gouldia minima | NA | NA |
| 0-2 | 2 | 4 | 10 | 4 | Gouldia minima | NA | NA |
| 0-2 | 2 | 4 | 10 | 4 | Gouldia minima | NA | NA |
| 8-10 | 10 | 12 | 10 | 4 | Gouldia minima | 685 | 587 |
| 8-10 | 10 | 12 | 10 | 4 | Gouldia minima | NA | NA |
| 8-10 | 10 | 12 | 10 | 4 | Gouldia minima | NA | NA |
| 8-10 | 10 | 12 | 10 | 4 | Gouldia minima | NA | NA |
| 8-10 | 10 | 12 | 10 | 4 | Gouldia minima | NA | NA |
| 8-10 | 10 | 12 | 10 | 4 | Gouldia minima | NA | NA |
| 8-10 | 10 | 12 | 10 | 4 | Gouldia minima | NA | NA |
| 8-10 | 10 | 12 | 10 | 4 | Gouldia minima | NA | NA |
| 8-10 | 10 | 12 | 10 | 4 | Gouldia minima | NA | NA |
| 8-10 | 10 | 12 | 10 | 4 | Gouldia minima | NA | NA |
| 8-10 | 10 | 12 | 10 | 3 | Gouldia minima | 2934 | 2805 |
| 8-10 | 10 | 12 | 10 | 3 | Gouldia minima | NA | NA |
| 8-10 | 10 | 12 | 10 | 4 | Gouldia minima | NA | NA |
| 8-10 | 10 | 12 | 10 | 4 | Gouldia minima | 1742 | 1593 |
| 8-10 | 10 | 12 | 10 | 4 | Gouldia minima | NA | NA |
| 8-10 | 10 | 12 | 10 | 4 | Gouldia minima | NA | NA |
| 8-10 | 10 | 12 | 10 | 4 | Gouldia minima | NA | NA |
| 8-10 | 10 | 12 | 10 | 4 | Gouldia minima | NA | NA |
| 8-10 | 10 | 12 | 10 | 3 | Gouldia minima | NA | NA |
| 8-10 | 10 | 12 | 10 | 4 | Gouldia minima | NA | NA |
| 8-10 | 10 | 12 | 10 | 4 | Gouldia minima | NA | NA |
| 8-10 | 10 | 12 | 10 | 4 | Gouldia minima | NA | NA |
| 8-10 | 10 | 12 | 10 | 4 | Gouldia minima | NA | NA |
| 8-10 | 10 | 12 | 10 | 4 | Gouldia minima | NA | NA |
| 8-10 | 10 | 12 | 10 | 4 | Gouldia minima | NA | NA |
| 8-10 | 10 | 12 | 10 | 4 | Gouldia minima | NA | NA |
| 8-10 | 10 | 12 | 10 | 4 | Gouldia minima | NA | NA |
| 8-10 | 10 | 12 | 10 | 4 | Gouldia minima | NA | NA |
| 25-30 | 30 | 30 | 30 | 4 | Gouldia minima | NA | NA |
| 25-30 | 30 | 30 | 30 | 4 | Gouldia minima | NA | NA |
| 25-30 | 30 | 30 | 30 | 5 | Gouldia minima | NA | NA |
| 25-30 | 30 | 30 | 30 | 4 | Gouldia minima | NA | NA |
| 25-30 | 30 | 30 | 30 | 4 | Gouldia minima | NA | NA |
| 25-30 | 30 | 30 | 30 | 4 | Gouldia minima | NA | NA |
| 25-30 | 30 | 30 | 30 | 4 | Gouldia minima | NA | NA |
| 25-30 | 30 | 30 | 30 | 4 | Gouldia minima | NA | NA |
| 25-30 | 30 | 30 | 30 | 4 | Gouldia minima | NA | NA |
| 25-30 | 30 | 30 | 30 | 4 | Gouldia minima | NA | NA |
| 25-30 | 30 | 30 | 30 | 4 | Gouldia minima | NA | NA |
| 25-30 | 30 | 30 | 30 | 4 | Gouldia minima | NA | NA |
| 25-30 | 30 | 30 | 30 | 4 | Gouldia minima | NA | NA |
| 25-30 | 30 | 30 | 30 | 4 | Gouldia minima | NA | NA |
| 25-30 | 30 | 30 | 30 | 4 | Gouldia minima | 3351 | 3195 |
| 25-30 | 30 | 30 | 30 | 4 | Gouldia minima | NA | NA |
| 25-30 | 30 | 30 | 30 | 4 | Gouldia minima | NA | NA |
| 25-30 | 30 | 30 | 30 | 4 | Gouldia minima | NA | NA |
| 25-30 | 30 | 30 | 30 | 4 | Gouldia minima | NA | NA |
| 25-30 | 30 | 30 | 30 | 4 | Gouldia minima | NA | NA |
| 25-30 | 30 | 30 | 30 | 4 | Gouldia minima | NA | NA |

| | | | | | | | |
|---|---|---|---|---|---|---|---|
| 25-30 | 30 | 30 | 30 | 4 | Gouldia minima | NA | NA |
| 25-30 | 30 | 30 | 30 | 4 | Gouldia minima | NA | NA |
| 25-30 | 30 | 30 | 30 | 4 | Gouldia minima | NA | NA |
| 25-30 | 30 | 30 | 30 | 4 | Gouldia minima | NA | NA |
| 25-30 | 30 | 30 | 30 | 4 | Gouldia minima | NA | NA |
| 25-30 | 30 | 30 | 30 | 4 | Gouldia minima | NA | NA |
| 25-30 | 30 | 30 | 30 | 4 | Gouldia minima | NA | NA |
| 25-30 | 30 | 30 | 30 | 4 | Gouldia minima | NA | NA |
| 25-30 | 30 | 30 | 30 | 4 | Gouldia minima | NA | NA |
| 45-50 | 50 | 50 | 50 | 4 | Gouldia minima | NA | NA |
| 45-50 | 50 | 50 | 50 | 4 | Gouldia minima | NA | NA |
| 45-50 | 50 | 50 | 50 | 4 | Gouldia minima | NA | NA |
| 45-50 | 50 | 50 | 50 | 4 | Gouldia minima | NA | NA |
| 45-50 | 50 | 50 | 50 | 4 | Gouldia minima | NA | NA |
| 45-50 | 50 | 50 | 50 | 4 | Gouldia minima | NA | NA |
| 45-50 | 50 | 50 | 50 | 4 | Gouldia minima | NA | NA |
| 45-50 | 50 | 50 | 50 | 4 | Gouldia minima | NA | NA |
| 45-50 | 50 | 50 | 50 | 4 | Gouldia minima | NA | NA |
| 45-50 | 50 | 50 | 50 | 4 | Gouldia minima | NA | NA |
| 45-50 | 50 | 50 | 50 | 4 | Gouldia minima | NA | NA |
| 45-50 | 50 | 50 | 50 | 4 | Gouldia minima | NA | NA |
| 45-50 | 50 | 50 | 50 | 4 | Gouldia minima | NA | NA |
| 45-50 | 50 | 50 | 50 | 5 | Gouldia minima | NA | NA |
| 45-50 | 50 | 50 | 50 | 4 | Gouldia minima | NA | NA |
| 45-50 | 50 | 50 | 50 | 4 | Gouldia minima | NA | NA |
| 45-50 | 50 | 50 | 50 | 4 | Gouldia minima | NA | NA |
| 45-50 | 50 | 50 | 50 | 4 | Gouldia minima | NA | NA |
| 45-50 | 50 | 50 | 50 | 4 | Gouldia minima | NA | NA |
| 45-50 | 50 | 50 | 50 | 4 | Gouldia minima | NA | NA |
| 45-50 | 50 | 50 | 50 | 4 | Gouldia minima | NA | NA |
| 45-50 | 50 | 50 | 50 | 4 | Gouldia minima | NA | NA |
| 45-50 | 50 | 50 | 50 | 4 | Gouldia minima | NA | NA |
| 45-50 | 50 | 50 | 50 | 4 | Gouldia minima | NA | NA |
| 45-50 | 50 | 50 | 50 | 4 | Gouldia minima | NA | NA |
| 45-50 | 50 | 50 | 50 | 4 | Gouldia minima | NA | NA |
| 45-50 | 50 | 50 | 50 | 4 | Gouldia minima | NA | NA |
| 45-50 | 50 | 50 | 50 | 4 | Gouldia minima | NA | NA |
| 45-50 | 50 | 50 | 50 | 4 | Gouldia minima | NA | NA |
| 45-50 | 50 | 50 | 50 | 4 | Gouldia minima | NA | NA |
| 65-70 | 70 | 70 | 70 | 5 | Gouldia minima | NA | NA |
| 65-70 | 70 | 70 | 70 | 5 | Gouldia minima | NA | NA |
| 65-70 | 70 | 70 | 70 | 5 | Gouldia minima | NA | NA |
| 65-70 | 70 | 70 | | | Gouldia minima | NA | NA |
| 65-70 | 70 | 70 | 70 | 5 | Gouldia minima | NA | NA |
| 65-70 | 70 | 70 | 70 | 4 | Gouldia minima | NA | NA |
| 65-70 | 70 | 70 | 70 | 4 | Gouldia minima | NA | NA |
| 65-70 | 70 | 70 | 70 | 4 | Gouldia minima | NA | NA |
| 65-70 | 70 | 70 | 70 | 4 | Gouldia minima | NA | NA |
| 65-70 | 70 | 70 | 70 | 4 | Gouldia minima | NA | NA |
| 65-70 | 70 | 70 | 70 | 4 | Gouldia minima | NA | NA |
| 65-70 | 70 | 70 | 70 | 4 | Gouldia minima | NA | NA |
| 65-70 | 70 | 70 | 70 | 4 | Gouldia minima | NA | NA |
| 65-70 | 70 | 70 | 70 | 4 | Gouldia minima | NA | NA |
| 65-70 | 70 | 70 | 70 | 4 | Gouldia minima | NA | NA |
| 65-70 | 70 | 70 | 70 | 5 | Gouldia minima | NA | NA |
| 65-70 | 70 | 70 | 70 | 4 | Gouldia minima | NA | NA |

| | | | | | | | | |
|---|---|---|---|---|---|---|---|---|
| 65-70 | 70 | 70 | 70 | 4 | Gouldia minima | | NA | NA |
| 65-70 | 70 | 70 | 70 | 4 | Gouldia minima | | NA | NA |
| 65-70 | 70 | 70 | 70 | 5 | Gouldia minima | | NA | NA |
| 65-70 | 70 | 70 | 70 | 5 | Gouldia minima | | NA | NA |
| 65-70 | 70 | 70 | 70 | 5 | Gouldia minima | | NA | NA |
| 65-70 | 70 | 70 | 70 | 5 | Gouldia minima | | NA | NA |
| 65-70 | 70 | 70 | 70 | 5 | Gouldia minima | | NA | NA |
| 65-70 | 70 | 70 | 70 | 5 | Gouldia minima | | NA | NA |
| 65-70 | 70 | 70 | 70 | 4 | Gouldia minima | | NA | NA |
| 65-70 | 70 | 70 | 70 | 5 | Gouldia minima | | NA | NA |
| 65-70 | 70 | 70 | 70 | 4 | Gouldia minima | | NA | NA |
| 65-70 | 70 | 70 | 70 | 5 | Gouldia minima | | NA | NA |
| 65-70 | 70 | 70 | 70 | | Gouldia minima | | NA | NA |
| 85-90 | 90 | 90 | 90 | 4 | Gouldia minima | | NA | NA |
| 85-90 | 90 | 90 | 90 | 5 | Gouldia minima | | NA | NA |
| 85-90 | 90 | 90 | 90 | 5 | Gouldia minima | | NA | NA |
| 85-90 | 90 | 90 | 90 | 5 | Gouldia minima | | NA | NA |
| 85-90 | 90 | 90 | 90 | 5 | Gouldia minima | | NA | NA |
| 85-90 | 90 | 90 | 90 | 4 | Gouldia minima | | NA | NA |
| 85-90 | 90 | 90 | 90 | 5 | Gouldia minima | | NA | NA |
| 85-90 | 90 | 90 | 90 | 5 | Gouldia minima | | NA | NA |
| 85-90 | 90 | 90 | 90 | 5 | Gouldia minima | | NA | NA |
| 85-90 | 90 | 90 | 90 | 5 | Gouldia minima | | NA | NA |
| 85-90 | 90 | 90 | 90 | 5 | Gouldia minima | | NA | NA |
| 85-90 | 90 | 90 | 90 | 5 | Gouldia minima | | NA | NA |
| 85-90 | 90 | 90 | 90 | 4 | Gouldia minima | | NA | NA |
| 85-90 | 90 | 90 | 90 | 4 | Gouldia minima | | NA | NA |
| 85-90 | 90 | 90 | 90 | 5 | Gouldia minima | | NA | NA |
| 85-90 | 90 | 90 | 90 | 5 | Gouldia minima | | NA | NA |
| 85-90 | 90 | 90 | 90 | 5 | Gouldia minima | | NA | NA |
| 85-90 | 90 | 90 | 90 | 5 | Gouldia minima | | NA | NA |
| 85-90 | 90 | 90 | 90 | 5 | Gouldia minima | | NA | NA |
| 85-90 | 90 | 90 | 90 | 5 | Gouldia minima | | NA | NA |
| 85-90 | 90 | 90 | 90 | 5 | Gouldia minima | | NA | NA |
| 85-90 | 90 | 90 | 90 | 5 | Gouldia minima | | NA | NA |
| 85-90 | 90 | 90 | 90 | 5 | Gouldia minima | | NA | NA |
| 85-90 | 90 | 90 | 90 | 5 | Gouldia minima | | NA | NA |
| 85-90 | 90 | 90 | 90 | 5 | Gouldia minima | | NA | NA |
| 85-90 | 90 | 90 | 90 | 5 | Gouldia minima | | NA | NA |
| 85-90 | 90 | 90 | 90 | 5 | Gouldia minima | | NA | NA |
| 85-90 | 90 | 90 | 90 | 5 | Gouldia minima | | NA | NA |
| 85-90 | 90 | 90 | 90 | 5 | Gouldia minima | | NA | NA |
| 85-90 | 90 | 90 | 90 | 5 | Gouldia minima | | NA | NA |
| 100-105 | 105 | 105 | 110 | 5 | Gouldia minima | | NA | NA |
| 100-105 | 105 | 105 | 110 | 5 | Gouldia minima | | NA | NA |
| 100-105 | 105 | 105 | 110 | 5 | Gouldia minima | | NA | NA |
| 100-105 | 105 | 105 | 110 | 5 | Gouldia minima | | NA | NA |
| 100-105 | 105 | 105 | 110 | 5 | Gouldia minima | | NA | NA |
| 100-105 | 105 | 105 | 110 | 5 | Gouldia minima | | NA | NA |
| 100-105 | 105 | 105 | 110 | 5 | Gouldia minima | | NA | NA |
| 100-105 | 105 | 105 | 110 | 4 | Gouldia minima | | NA | NA |
| 100-105 | 105 | 105 | 110 | 5 | Gouldia minima | | NA | NA |
| 100-105 | 105 | 105 | 110 | 5 | Gouldia minima | | NA | NA |
| 100-105 | 105 | 105 | 110 | 5 | Gouldia minima | outlier in DL plot and in conc plot | NA | NA |
| 100-105 | 105 | 105 | 110 | 5 | Gouldia minima | | NA | NA |
| 100-105 | 105 | 105 | 110 | 5 | Gouldia minima | | NA | NA |

| | | | | | | | |
|---|---|---|---|---|---|---|---|
| 100-105 | 105 | 105 | 110 | 5 | Gouldia minima | NA | NA |
| 100-105 | 105 | 105 | 110 | 5 | Gouldia minima | NA | NA |
| 100-105 | 105 | 105 | 110 | 5 | Gouldia minima | NA | NA |
| 100-105 | 105 | 105 | 110 | 5 | Gouldia minima | NA | NA |
| 100-105 | 105 | 105 | 110 | 5 | Gouldia minima | NA | NA |
| 100-105 | 105 | 105 | 110 | 5 | Gouldia minima | NA | NA |
| 100-105 | 105 | 105 | 110 | 5 | Gouldia minima | NA | NA |
| 100-105 | 105 | 105 | 110 | 4 | Gouldia minima | NA | NA |
| 100-105 | 105 | 105 | 110 | 5 | Gouldia minima | NA | NA |
| 100-105 | 105 | 105 | 110 | 5 | Gouldia minima | NA | NA |
| 100-105 | 105 | 105 | 110 | 4 | Gouldia minima | NA | NA |
| 100-105 | 105 | 105 | 110 | 5 | Gouldia minima | NA | NA |
| 100-105 | 105 | 105 | 110 | 4 | Gouldia minima | NA | NA |
| 100-105 | 105 | 105 | 110 | 4 | Gouldia minima | NA | NA |
| 125-130 | 130 | 130 | 130 | 5 | Gouldia minima | NA | NA |
| 125-130 | 130 | 130 | 130 | 5 | Gouldia minima | NA | NA |
| 125-130 | 130 | 130 | 130 | 5 | Gouldia minima | NA | NA |
| 125-130 | 130 | 130 | 130 | 5 | Gouldia minima | NA | NA |
| 125-130 | 130 | 130 | 130 | 5 | Gouldia minima | NA | NA |
| 125-130 | 130 | 130 | 130 | 5 | Gouldia minima | NA | NA |
| 125-130 | 130 | 130 | 130 | 5 | Gouldia minima | NA | NA |
| 125-130 | 130 | 130 | 130 | 5 | Gouldia minima | NA | NA |
| 125-130 | 130 | 130 | 130 | 5 | Gouldia minima | NA | NA |
| 125-130 | 130 | 130 | 130 | 5 | Gouldia minima | NA | NA |
| 125-130 | 130 | 130 | 130 | 5 | Gouldia minima | NA | NA |
| 125-130 | 130 | 130 | 130 | 5 | Gouldia minima | NA | NA |
| 125-130 | 130 | 130 | 130 | 5 | Gouldia minima | NA | NA |
| 125-130 | 130 | 130 | 130 | 5 | Gouldia minima | NA | NA |
| 125-130 | 130 | 130 | 130 | 5 | Gouldia minima | NA | NA |
| 125-130 | 130 | 130 | 130 | 5 | Gouldia minima | NA | NA |
| 125-130 | 130 | 130 | 130 | 5 | Gouldia minima | NA | NA |
| 125-130 | 130 | 130 | 130 | 5 | Gouldia minima | NA | NA |
| 125-130 | 130 | 130 | 130 | 5 | Gouldia minima | NA | NA |
| 125-130 | 130 | 130 | 130 | 5 | Gouldia minima | NA | NA |
| 125-130 | 130 | 130 | 130 | 5 | Gouldia minima | NA | NA |
| 125-130 | 130 | 130 | 130 | 5 | Gouldia minima | NA | NA |
| 125-130 | 130 | 130 | 130 | | Gouldia minima | NA | NA |
| 125-130 | 130 | 130 | 130 | 5 | Gouldia minima | NA | NA |
| 125-130 | 130 | 130 | 130 | 5 | Gouldia minima | NA | NA |
| 125-130 | 130 | 130 | 130 | 5 | Gouldia minima | NA | NA |
| 125-130 | 130 | 130 | 130 | 5 | Gouldia minima | NA | NA |
| 125-130 | 130 | 130 | 130 | 5 | Gouldia minima | NA | NA |
| 125-130 | 130 | 130 | 130 | 5 | Gouldia minima | NA | NA |
| 125-130 | 130 | 130 | 130 | 5 | Gouldia minima | NA | NA |
| 145-150 | 150 | 150 | 150 | | Gouldia minima | NA | NA |
| 145-150 | 150 | 150 | 150 | 5 | Gouldia minima | NA | NA |
| 145-150 | 150 | 150 | 150 | 5 | Gouldia minima | NA | NA |
| 145-150 | 150 | 150 | 150 | 5 | Gouldia minima | NA | NA |
| 145-150 | 150 | 150 | 150 | 5 | Gouldia minima | NA | NA |
| 145-150 | 150 | 150 | 150 | 5 | Gouldia minima | NA | NA |
| 145-150 | 150 | 150 | 150 | 5 | Gouldia minima | NA | NA |
| 145-150 | 150 | 150 | 150 | 5 | Gouldia minima | NA | NA |
| 145-150 | 150 | 150 | 150 | 5 | Gouldia minima | NA | NA |
| 145-150 | 150 | 150 | 150 | 5 | Gouldia minima | NA | NA |
| 145-150 | 150 | 150 | 150 | | Gouldia minima | NA | NA |
| 145-150 | 150 | 150 | 150 | 5 | Gouldia minima | NA | NA |

| | | | | | | | | | |
|---|---|---|---|---|---|---|---|---|---|
| 145-150 | 150 | 150 | 150 | 5 | Gouldia minima | | | NA | NA |
| 145-150 | 150 | 150 | 150 | 5 | Gouldia minima | | | NA | NA |
| 145-150 | 150 | 150 | 150 | 5 | Gouldia minima | | | NA | NA |
| 145-150 | 150 | 150 | 150 | 5 | Gouldia minima | | | NA | NA |
| 145-150 | 150 | 150 | 150 | 5 | Gouldia minima | | | NA | NA |
| 145-150 | 150 | 150 | 150 | 5 | Gouldia minima | | | NA | NA |
| 145-150 | 150 | 150 | 150 | 5 | Gouldia minima | | | NA | NA |
| 145-150 | 150 | 150 | 150 | 5 | Gouldia minima | | | NA | NA |
| 145-150 | 150 | 150 | 150 | 5 | Gouldia minima | | | NA | NA |
| 145-150 | 150 | 150 | 150 | 5 | Gouldia minima | | | NA | NA |
| 145-150 | 150 | 150 | 150 | 5 | Gouldia minima | | | NA | NA |
| 145-150 | 150 | 150 | 150 | 5 | Gouldia minima | outlier | outlier in L Ser plots | NA | NA |
| 145-150 | 150 | 150 | 150 | 5 | Gouldia minima | | | NA | NA |
| 145-150 | 150 | 150 | 150 | 5 | Gouldia minima | | | NA | NA |
| 145-150 | 150 | 150 | 150 | 5 | Gouldia minima | | | NA | NA |
| 145-150 | 150 | 150 | 150 | 5 | Gouldia minima | | | NA | NA |
| 145-150 | 150 | 150 | 150 | 5 | Gouldia minima | | | NA | NA |
| 145-150 | 150 | 150 | 150 | 5 | Gouldia minima | | | NA | NA |
| 18-20 | 20 | 20 | 20 | | Gouldia minima | | | NA | NA |
| 18-20 | 20 | 20 | 20 | | Gouldia minima | | | NA | NA |
| 18-20 | 20 | 20 | 20 | | Gouldia minima | | | NA | NA |

| ageOld | Asp_DL | Glu_DL | DL_Ser | DL_Ala | DL_Val | DL_Phe | DL_Ile | DL_Leu | L-Ser/ L-Asp | [Asp] | [Glu] | [Ser] | [Ala] | [Val] | [Phe] |
|---|---|---|---|---|---|---|---|---|---|---|---|---|---|---|---|
| NA | 0.263 | 0.125 | 0.375 | 0.200 | 0.079 | 0.147 | 0.089 | 0.165 | 0.2 | 662 | 311 | 154 | 332 | 137 | 83 |
| NA | 0.183 | 0.039 | 0.336 | 0.084 | 0.028 | 0.057 | 0.036 | 0.081 | 0.4 | 631 | 646 | 282 | 415 | 161 | 115 |
| NA | 0.222 | 0.079 | 0.399 | 0.143 | 0.048 | 0.091 | 0.079 | 0.116 | 0.3 | 658 | 382 | 222 | 364 | 171 | 116 |
| NA | 0.145 | 0.067 | 0.196 | 0.110 | 0.022 | 0.044 | 0.032 | 0.094 | 0.3 | 926 | 526 | 312 | 511 | 283 | 179 |
| NA | 0.243 | 0.099 | 0.386 | 0.130 | 0.054 | 0.125 | 0.071 | 0.160 | 0.3 | 746 | 358 | 217 | 444 | 159 | 109 |
| NA | 0.206 | 0.057 | 0.162 | 0.111 | 0.043 | 0.059 | 0.045 | 0.066 | 0.6 | 895 | 743 | 487 | 547 | 255 | 189 |
| NA | 0.162 | 0.071 | 0.238 | 0.106 | 0.024 | 0.054 | 0.049 | 0.111 | 0.3 | 930 | 522 | 310 | 537 | 284 | 171 |
| NA | 0.140 | 0.045 | 0.308 | 0.064 | 0.019 | 0.040 | 0.043 | 0.057 | 0.4 | 1154 | 740 | 523 | 840 | 306 | 209 |
| NA | 0.220 | 0.084 | 0.367 | 0.105 | 0.043 | 0.082 | 0.067 | 0.130 | 0.3 | 711 | 378 | 217 | 453 | 162 | 106 |
| NA | 0.222 | 0.078 | 0.424 | 0.108 | 0.044 | 0.079 | 0.072 | 0.087 | 0.3 | 752 | 497 | 250 | 533 | 208 | 143 |
| NA | 0.188 | 0.091 | 0.281 | 0.157 | 0.044 | 0.078 | 0.068 | 0.066 | 0.3 | 927 | 445 | 275 | 549 | 277 | 168 |
| NA | 0.184 | 0.097 | 0.220 | 0.141 | 0.039 | 0.075 | 0.064 | 0.089 | 0.3 | 867 | 479 | 279 | 595 | 284 | 176 |
| NA | 0.247 | 0.097 | 0.430 | 0.164 | 0.068 | 0.116 | 0.082 | 0.117 | 0.3 | 637 | 377 | 190 | 414 | 186 | 122 |
| NA | 0.231 | 0.092 | 0.398 | 0.148 | 0.056 | 0.095 | 0.068 | 0.115 | 0.3 | 774 | 455 | 235 | 452 | 196 | 122 |
| NA | 0.258 | 0.104 | 0.419 | 0.171 | 0.074 | 0.131 | 0.087 | 0.171 | 0.2 | 723 | 378 | 202 | 441 | 161 | 112 |
| NA | 0.193 | 0.072 | 0.348 | 0.093 | 0.037 | 0.072 | 0.055 | 0.107 | 0.3 | 816 | 515 | 289 | 576 | 251 | 165 |
| NA | 0.212 | 0.084 | 0.332 | 0.126 | 0.046 | 0.083 | 0.060 | 0.106 | 0.3 | 831 | 552 | 277 | 564 | 276 | 163 |
| NA | 0.119 | 0.050 | 0.221 | 0.058 | 0.012 | 0.035 | 0.015 | 0.055 | 0.3 | 1486 | 969 | 562 | 889 | 504 | 330 |
| NA | 0.171 | 0.055 | 0.327 | 0.076 | 0.041 | 0.046 | 0.032 | 0.051 | 0.4 | 976 | 659 | 390 | 656 | 282 | 190 |
| NA | 0.182 | 0.077 | 0.303 | 0.110 | 0.027 | 0.061 | 0.041 | 0.096 | 0.3 | 1273 | 628 | 426 | 632 | 269 | 201 |
| NA | 0.318 | 0.138 | 0.447 | 0.267 | 0.116 | 0.189 | 0.153 | 0.235 | 0.2 | 524 | 330 | 124 | 335 | 135 | 89 |
| NA | 0.123 | 0.038 | 0.254 | 0.053 | 0.022 | 0.039 | 0.046 | 0.048 | 0.4 | 892 | 657 | 433 | 669 | 259 | 188 |
| NA | 0.142 | 0.048 | 0.312 | 0.058 | 0.024 | 0.042 | 0.045 | 0.051 | 0.4 | 1112 | 586 | 542 | 828 | 301 | 232 |
| NA | 0.103 | 0.063 | 0.149 | 0.060 | 0.012 | 0.022 | 0.048 | 0.098 | 0.3 | 2590 | 1087 | 707 | 1278 | 682 | 417 |
| NA | 0.123 | 0.052 | 0.206 | 0.062 | 0.021 | 0.035 | 0.033 | 0.088 | 0.4 | 1316 | 1071 | 510 | 951 | 512 | 306 |
| 487 | 0.102 | 0.054 | 0.123 | 0.061 | 0.013 | 0.050 | 0.077 | 0.044 | 0.4 | 2114 | 1349 | 832 | 1536 | 997 | 595 |
| NA | 0.190 | 0.092 | 0.259 | 0.117 | 0.042 | 0.074 | 0.057 | 0.091 | 0.3 | 718 | 515 | 237 | 681 | 282 | 168 |
| NA | 0.081 | 0.045 | 0.083 | 0.057 | 0.014 | 0.018 | 0.022 | 0.033 | 0.4 | 3011 | 2086 | 1207 | 2496 | 1453 | 936 |
| NA | 0.119 | 0.034 | 0.251 | 0.044 | 0.014 | 0.032 | 0.023 | 0.048 | 0.5 | 1620 | 1142 | 824 | 922 | 513 | 346 |
| NA | 0.212 | 0.105 | 0.262 | 0.163 | 0.050 | 0.090 | 0.064 | 0.121 | 0.3 | 1002 | 630 | 287 | 759 | 391 | 206 |
| NA | 0.201 | 0.120 | 0.282 | 0.142 | 0.053 | 0.114 | 0.039 | 0.101 | 0.3 | 994 | 620 | 274 | 677 | 345 | 222 |
| 350 | 0.137 | 0.053 | 0.279 | 0.052 | 0.020 | 0.049 | 0.060 | 0.041 | 0.4 | 2396 | 1229 | 1001 | 1137 | 660 | 471 |
| NA | 0.154 | 0.085 | 0.264 | 0.093 | 0.016 | 0.041 | 0.044 | 0.070 | 0.2 | 1434 | 665 | 382 | 779 | 388 | 219 |
| NA | 0.118 | 0.032 | 0.197 | 0.051 | 0.020 | 0.039 | 0.030 | 0.041 | 0.5 | 1070 | 859 | 559 | 654 | 303 | 218 |
| NA | 0.246 | 0.085 | 0.388 | 0.134 | 0.048 | 0.107 | 0.080 | 0.102 | 0.2 | 865 | 573 | 227 | 582 | 249 | 149 |
| NA | 0.186 | 0.061 | 0.341 | 0.082 | 0.035 | 0.066 | 0.048 | 0.065 | 0.3 | 845 | 553 | 301 | 487 | 216 | 142 |
| NA | 0.189 | 0.035 | 0.432 | 0.074 | 0.037 | 0.069 | 0.047 | 0.050 | 0.4 | 604 | 698 | 313 | 460 | 172 | 143 |
| NA | 0.224 | 0.077 | 0.397 | 0.119 | 0.043 | 0.078 | 0.074 | 0.101 | 0.3 | 944 | 568 | 303 | 553 | 262 | 176 |
| NA | 0.239 | 0.084 | 0.464 | 0.142 | 0.056 | 0.100 | 0.069 | 0.110 | 0.3 | 713 | 486 | 218 | 544 | 222 | 139 |
| NA | 0.227 | 0.078 | 0.438 | 0.101 | 0.037 | 0.072 | 0.054 | 0.085 | 0.3 | 895 | 645 | 283 | 668 | 276 | 165 |
| NA | 0.166 | 0.059 | 0.265 | 0.086 | 0.023 | 0.048 | 0.033 | 0.071 | 0.3 | 1210 | 731 | 392 | 755 | 471 | 288 |
| NA | 0.255 | 0.114 | 0.399 | 0.199 | 0.076 | 0.121 | 0.103 | 0.129 | 0.2 | 691 | 446 | 177 | 480 | 223 | 129 |
| NA | 0.202 | 0.108 | 0.287 | 0.163 | 0.045 | 0.080 | 0.059 | 0.072 | 0.3 | 2079 | 1251 | 563 | 1319 | 714 | 416 |
| NA | 0.118 | 0.036 | 0.226 | 0.044 | 0.015 | 0.033 | 0.039 | 0.050 | 0.4 | 2054 | 1335 | 906 | 1198 | 566 | 419 |
| NA | 0.179 | 0.079 | 0.322 | 0.090 | 0.032 | 0.058 | 0.066 | 0.130 | 0.3 | 1021 | 571 | 333 | 748 | 361 | 207 |
| NA | 0.111 | 0.053 | 0.154 | 0.097 | 0.016 | 0.034 | 0.021 | 0.050 | 0.4 | 1519 | 895 | 611 | 1170 | 647 | 289 |
| NA | 0.180 | 0.061 | 0.376 | 0.079 | 0.035 | 0.049 | 0.055 | 0.090 | 0.3 | 831 | 566 | 296 | 532 | 247 | 146 |
| NA | 0.184 | 0.090 | 0.275 | 0.138 | 0.036 | 0.064 | 0.060 | 0.074 | 0.3 | 844 | 507 | 262 | 595 | 306 | 187 |
| NA | 0.239 | 0.097 | 0.405 | 0.162 | 0.051 | 0.091 | 0.077 | 0.129 | 0.2 | 872 | 532 | 231 | 598 | 286 | 158 |
| NA | 0.187 | 0.067 | 0.345 | 0.091 | 0.032 | 0.060 | 0.043 | 0.083 | 0.3 | 711 | 488 | 243 | 459 | 221 | 131 |

| | | | | | | | | | | | | | | | |
|---|---|---|---|---|---|---|---|---|---|---|---|---|---|---|---|
| NA | 0.213 | 0.094 | 0.357 | 0.117 | 0.050 | 0.079 | 0.077 | 0.188 | 0.3 | 726 | 400 | 230 | 513 | 222 | 136 |
| NA | 0.134 | 0.040 | 0.260 | 0.054 | 0.019 | 0.037 | 0.027 | 0.078 | 0.4 | 1029 | 742 | 466 | 649 | 307 | 209 |
| NA | 0.168 | 0.096 | 0.213 | 0.126 | 0.037 | 0.065 | 0.046 | 0.101 | 0.3 | 1060 | 649 | 340 | 781 | 423 | 236 |
| NA | 0.154 | 0.069 | 0.285 | 0.081 | 0.027 | 0.048 | 0.032 | 0.075 | 0.3 | 1082 | 556 | 314 | 737 | 391 | 223 |
| NA | 0.202 | 0.094 | 0.273 | 0.158 | 0.051 | 0.081 | 0.048 | 0.138 | 0.3 | 753 | 418 | 225 | 479 | 246 | 156 |
| NA | 0.177 | 0.060 | 0.383 | 0.064 | 0.030 | 0.057 | 0.031 | 0.125 | 0.3 | 876 | 550 | 344 | 623 | 239 | 160 |
| NA | 0.159 | 0.043 | 0.337 | 0.068 | 0.022 | 0.048 | 0.049 | 0.053 | 0.4 | 850 | 692 | 359 | 560 | 229 | 151 |
| NA | 0.155 | 0.044 | 0.346 | 0.058 | 0.027 | 0.050 | 0.029 | 0.117 | 0.4 | 1154 | 689 | 520 | 654 | 318 | 213 |
| NA | 0.152 | 0.072 | 0.224 | 0.094 | 0.029 | 0.047 | 0.036 | 0.098 | 0.3 | 1258 | 703 | 379 | 893 | 469 | 281 |
| NA | 0.181 | 0.093 | 0.263 | 0.121 | 0.040 | 0.080 | 0.045 | 0.126 | 0.3 | 1096 | 524 | 301 | 558 | 282 | 168 |
| NA | 0.263 | 0.086 | 0.495 | 0.158 | 0.054 | 0.127 | 0.069 | 0.098 | 0.3 | 635 | 509 | 225 | 523 | 223 | 156 |
| 4640 | 0.297 | 0.095 | 0.544 | 0.193 | 0.069 | 0.152 | 0.079 | 0.121 | 0.2 | 1022 | 769 | 279 | 816 | 379 | 216 |
| NA | 0.260 | 0.075 | 0.581 | 0.152 | 0.047 | 0.135 | 0.052 | 0.100 | 0.3 | 747 | 595 | 245 | 606 | 295 | 178 |
| 4753 | 0.313 | 0.100 | 0.598 | 0.224 | 0.074 | 0.170 | 0.077 | 0.137 | 0.2 | 1049 | 894 | 295 | 774 | 361 | 245 |
| NA | 0.229 | 0.137 | 0.414 | 0.125 | 0.045 | 0.122 | 0.042 | 0.087 | 0.2 | 1110 | 620 | 302 | 640 | 305 | 188 |
| NA | 0.290 | 0.058 | 0.580 | 0.137 | 0.049 | 0.107 | 0.057 | 0.065 | 0.3 | 1072 | 880 | 355 | 679 | 337 | 235 |
| NA | 0.300 | 0.065 | 0.562 | 0.171 | 0.061 | 0.128 | 0.074 | 0.080 | 0.2 | 997 | 864 | 293 | 660 | 324 | 232 |
| NA | 0.291 | 0.062 | 0.568 | 0.142 | 0.049 | 0.117 | 0.064 | 0.078 | 0.3 | 1190 | 918 | 368 | 837 | 399 | 284 |
| NA | 0.300 | 0.085 | 0.402 | 0.186 | 0.064 | 0.174 | 0.076 | 0.125 | 0.4 | 658 | 533 | 282 | 560 | 229 | 161 |
| 2986 | 0.273 | 0.086 | 0.559 | 0.156 | 0.051 | 0.122 | 0.057 | 0.109 | 0.3 | 833 | 588 | 289 | 604 | 317 | 197 |
| NA | 0.256 | 0.072 | 0.500 | 0.151 | 0.048 | 0.128 | 0.052 | 0.098 | 0.3 | 940 | 702 | 347 | 732 | 350 | 242 |
| NA | 0.273 | 0.087 | 0.541 | 0.180 | 0.063 | 0.153 | 0.084 | 0.101 | 0.2 | 633 | 559 | 174 | 485 | 222 | 126 |
| NA | 0.276 | 0.073 | 0.573 | 0.169 | 0.068 | 0.144 | 0.052 | 0.088 | 0.3 | 757 | 690 | 259 | 571 | 266 | 195 |
| NA | 0.349 | 0.074 | 0.499 | 0.249 | 0.086 | 0.190 | 0.106 | 0.102 | 0.3 | 828 | 1030 | 260 | 626 | 302 | 216 |
| NA | 0.172 | 0.057 | 0.372 | 0.072 | 0.035 | 0.064 | 0.045 | 0.046 | 0.3 | 947 | 698 | 364 | 644 | 284 | 185 |
| NA | 0.307 | 0.088 | 0.592 | 0.171 | 0.067 | 0.131 | 0.076 | 0.088 | 0.2 | 873 | 704 | 240 | 656 | 279 | 176 |
| NA | 0.342 | 0.111 | 0.534 | 0.275 | 0.113 | 0.190 | 0.123 | 0.146 | 0.2 | 597 | 594 | 135 | 461 | 217 | 135 |
| NA | 0.278 | 0.112 | 0.519 | 0.180 | 0.066 | 0.159 | 0.066 | 0.136 | 0.2 | 661 | 440 | 187 | 496 | 215 | 127 |
| NA | 0.189 | 0.124 | 0.242 | 0.121 | 0.028 | 0.054 | 0.042 | 0.097 | 0.2 | 1436 | 793 | 349 | 835 | 455 | 251 |
| NA | 0.266 | 0.055 | 0.617 | 0.116 | 0.039 | 0.098 | 0.057 | 0.061 | 0.3 | 1078 | 786 | 395 | 774 | 314 | 239 |
| NA | 0.175 | 0.035 | 0.445 | 0.048 | 0.014 | 0.041 | 0.018 | 0.034 | 0.5 | 1882 | 1156 | 1093 | 948 | 500 | 458 |
| NA | 0.294 | 0.104 | 0.550 | 0.195 | 0.078 | 0.143 | 0.084 | 0.113 | 0.2 | 708 | 512 | 184 | 493 | 200 | 127 |
| NA | 0.279 | 0.093 | 0.520 | 0.181 | 0.073 | 0.128 | 0.098 | 0.121 | 0.2 | 517 | 467 | 152 | 454 | 201 | 120 |
| NA | 0.295 | 0.111 | 0.519 | 0.190 | 0.078 | 0.141 | 0.106 | 0.150 | 0.2 | 652 | 399 | 161 | 455 | 198 | 118 |
| NA | 0.272 | 0.073 | 0.611 | 0.156 | 0.048 | 0.106 | 0.061 | 0.090 | 0.3 | 896 | 659 | 290 | 594 | 283 | 203 |
| NA | 0.226 | 0.077 | 0.424 | 0.110 | 0.036 | 0.075 | 0.069 | 0.090 | 0.3 | 737 | 447 | 249 | 435 | 182 | 126 |
| NA | 0.281 | 0.105 | 0.466 | 0.148 | 0.078 | 0.107 | 0.064 | 0.064 | 0.3 | 676 | 378 | 193 | 389 | 161 | 110 |
| NA | 0.288 | 0.103 | 0.524 | 0.178 | 0.069 | 0.140 | 0.085 | 0.099 | 0.2 | 632 | 405 | 169 | 426 | 172 | 115 |
| NA | 0.233 | 0.078 | 0.438 | 0.110 | 0.042 | 0.077 | 0.063 | 0.081 | 0.3 | 879 | 543 | 288 | 571 | 261 | 172 |
| 4848 | 0.298 | 0.103 | 0.623 | 0.204 | 0.076 | 0.167 | 0.075 | 0.128 | 0.2 | 863 | 582 | 206 | 589 | 302 | 170 |
| NA | 0.315 | 0.094 | 0.558 | 0.221 | 0.091 | 0.165 | 0.114 | 0.125 | 0.2 | 639 | 538 | 170 | 478 | 194 | 126 |
| NA | 0.291 | 0.078 | 0.565 | 0.187 | 0.068 | 0.149 | 0.087 | 0.095 | 0.2 | 1856 | 1806 | 560 | 1365 | 629 | 465 |
| NA | 0.311 | 0.074 | 0.627 | 0.184 | 0.064 | 0.129 | 0.072 | 0.084 | 0.2 | 1080 | 752 | 311 | 640 | 331 | 234 |
| NA | 0.321 | 0.104 | 0.623 | 0.228 | 0.081 | 0.151 | 0.093 | 0.138 | 0.2 | 814 | 503 | 208 | 551 | 257 | 179 |
| NA | 0.284 | 0.092 | 0.289 | 0.153 | 0.074 | 0.126 | 0.085 | 0.120 | 0.4 | 736 | 534 | 323 | 586 | 220 | 144 |
| NA | 0.276 | 0.074 | 0.568 | 0.158 | 0.059 | 0.120 | 0.071 | 0.085 | 0.2 | 801 | 717 | 236 | 615 | 264 | 157 |
| 6230 | 0.330 | 0.107 | 0.513 | 0.290 | 0.088 | 0.191 | 0.099 | 0.131 | 0.2 | 716 | 619 | 192 | 523 | 251 | 167 |
| NA | 0.310 | 0.085 | 0.558 | 0.206 | 0.078 | 0.139 | 0.091 | 0.107 | 0.2 | 855 | 768 | 236 | 610 | 272 | 186 |
| NA | 0.279 | 0.079 | 0.566 | 0.163 | 0.055 | 0.121 | 0.069 | 0.089 | 0.3 | 987 | 765 | 310 | 712 | 326 | 244 |
| NA | 0.346 | 0.138 | 0.572 | 0.271 | 0.122 | 0.203 | 0.152 | 0.214 | 0.2 | 497 | 324 | 105 | 362 | 148 | 88 |
| NA | 0.278 | 0.052 | 0.559 | 0.139 | 0.051 | 0.105 | 0.056 | 0.058 | 0.3 | 1092 | 1225 | 367 | 741 | 353 | 242 |
| NA | 0.301 | 0.084 | 0.563 | 0.184 | 0.085 | 0.149 | 0.090 | 0.134 | 0.2 | 740 | 676 | 199 | 589 | 251 | 163 |
| NA | 0.311 | 0.069 | 0.580 | 0.175 | 0.058 | 0.121 | 0.075 | 0.072 | 0.2 | 1189 | 1164 | 344 | 841 | 388 | 270 |
| NA | 0.311 | 0.090 | 0.580 | 0.190 | 0.070 | 0.137 | 0.084 | 0.113 | 0.2 | 1037 | 746 | 270 | 697 | 338 | 212 |
| NA | 0.372 | 0.118 | 0.572 | 0.279 | 0.119 | 0.220 | 0.149 | 0.169 | 0.2 | 631 | 560 | 143 | 491 | 221 | 130 |
| NA | 0.259 | 0.058 | 0.590 | 0.095 | 0.035 | 0.081 | 0.049 | 0.042 | 0.3 | 1122 | 723 | 460 | 720 | 309 | 262 |

| | | | | | | | | | | | | | | | | |
|---|---|---|---|---|---|---|---|---|---|---|---|---|---|---|---|---|
| NA | 0.305 | 0.069 | 0.586 | 0.159 | 0.050 | 0.127 | 0.064 | 0.075 | 0.2 | 1224 | 851 | 371 | 731 | 381 | 274 |
| NA | 0.286 | 0.101 | 0.531 | 0.187 | 0.069 | 0.133 | 0.085 | 0.161 | 0.3 | 586 | 398 | 183 | 441 | 177 | 117 |
| NA | 0.293 | 0.103 | 0.550 | 0.156 | 0.064 | 0.128 | 0.076 | 0.220 | 0.2 | 614 | 294 | 177 | 421 | 179 | 118 |
| NA | 0.293 | 0.108 | 0.507 | 0.211 | 0.072 | 0.148 | 0.084 | 0.149 | 0.2 | 849 | 662 | 242 | 712 | 352 | 207 |
| NA | 0.323 | 0.099 | 0.549 | 0.222 | 0.088 | 0.167 | 0.106 | 0.133 | 0.2 | 842 | 696 | 187 | 613 | 318 | 188 |
| NA | 0.281 | 0.066 | 0.527 | 0.146 | 0.057 | 0.118 | 0.080 | 0.085 | 0.3 | 688 | 607 | 229 | 546 | 218 | 154 |
| NA | 0.296 | 0.064 | 0.573 | 0.167 | 0.056 | 0.111 | 0.066 | 0.077 | 0.3 | 969 | 832 | 303 | 690 | 332 | 225 |
| NA | 0.278 | 0.096 | 0.573 | 0.196 | 0.076 | 0.146 | 0.076 | 0.138 | 0.2 | 671 | 590 | 179 | 582 | 273 | 140 |
| NA | 0.316 | 0.093 | 0.534 | 0.200 | 0.088 | 0.153 | 0.105 | 0.107 | 0.2 | 711 | 605 | 182 | 559 | 215 | 143 |
| NA | 0.352 | 0.090 | 0.549 | 0.228 | 0.086 | 0.168 | 0.105 | 0.117 | 0.2 | 855 | 774 | 203 | 530 | 280 | 173 |
| NA | 0.314 | 0.101 | 0.559 | 0.194 | 0.094 | 0.158 | 0.109 | 0.126 | 0.2 | 610 | 503 | 159 | 479 | 183 | 122 |
| NA | 0.305 | 0.079 | 0.574 | 0.187 | 0.074 | 0.137 | 0.087 | 0.091 | 0.2 | 821 | 735 | 242 | 548 | 254 | 169 |
| NA | 0.262 | 0.062 | 0.535 | 0.118 | 0.035 | 0.082 | 0.046 | 0.070 | 0.3 | 1086 | 759 | 396 | 706 | 345 | 255 |
| NA | 0.282 | 0.075 | 0.552 | 0.166 | 0.046 | 0.127 | 0.065 | 0.094 | 0.3 | 1260 | 808 | 388 | 715 | 419 | 307 |
| 4799 | 0.293 | 0.092 | 0.558 | 0.192 | 0.068 | 0.117 | 0.068 | 0.104 | 0.2 | 687 | 550 | 192 | 526 | 266 | 158 |
| NA | 0.335 | 0.115 | 0.622 | 0.257 | 0.106 | 0.190 | 0.131 | 0.149 | 0.2 | 707 | 585 | 166 | 536 | 245 | 150 |
| NA | 0.297 | 0.076 | 0.573 | 0.155 | 0.063 | 0.125 | 0.070 | 0.092 | 0.2 | 915 | 706 | 271 | 659 | 279 | 190 |
| 3319 | 0.258 | 0.063 | 0.535 | 0.154 | 0.045 | 0.115 | 0.045 | 0.081 | 0.3 | 961 | 829 | 327 | 680 | 369 | 223 |
| NA | 0.323 | 0.076 | 0.544 | 0.189 | 0.068 | 0.140 | 0.085 | 0.084 | 0.3 | 716 | 622 | 228 | 503 | 222 | 151 |
| NA | 0.311 | 0.096 | 0.569 | 0.200 | 0.080 | 0.151 | 0.094 | 0.116 | 0.2 | 708 | 583 | 189 | 543 | 235 | 152 |
| NA | 0.306 | 0.077 | 0.568 | 0.177 | 0.066 | 0.140 | 0.085 | 0.088 | 0.2 | 847 | 678 | 243 | 621 | 266 | 183 |
| NA | 0.297 | 0.083 | 0.542 | 0.181 | 0.061 | 0.123 | 0.080 | 0.115 | 0.2 | 758 | 603 | 217 | 580 | 264 | 174 |
| NA | 0.347 | 0.088 | 0.547 | 0.209 | 0.086 | 0.161 | 0.106 | 0.122 | 0.2 | 644 | 557 | 166 | 477 | 197 | 135 |
| NA | 0.304 | 0.094 | 0.567 | 0.175 | 0.075 | 0.144 | 0.095 | 0.118 | 0.2 | 774 | 571 | 212 | 592 | 244 | 149 |
| NA | 0.296 | 0.094 | 0.471 | 0.172 | 0.074 | 0.128 | 0.088 | 0.126 | 0.2 | 615 | 427 | 156 | 419 | 169 | 112 |
| NA | 0.299 | 0.077 | 0.549 | 0.162 | 0.061 | 0.123 | 0.073 | 0.090 | 0.2 | 782 | 671 | 231 | 602 | 259 | 172 |
| NA | 0.352 | 0.079 | 0.498 | 0.219 | 0.087 | 0.173 | 0.102 | 0.099 | 0.2 | 741 | 781 | 177 | 539 | 275 | 186 |
| NA | 0.345 | 0.071 | 0.506 | 0.225 | 0.088 | 0.180 | 0.105 | 0.103 | 0.2 | 731 | 816 | 173 | 559 | 269 | 174 |
| NA | 0.299 | 0.070 | 0.521 | 0.148 | 0.056 | 0.118 | 0.071 | 0.083 | 0.2 | 986 | 754 | 283 | 675 | 308 | 218 |
| NA | 0.291 | 0.057 | 0.538 | 0.132 | 0.052 | 0.108 | 0.073 | 0.065 | 0.3 | 902 | 774 | 283 | 641 | 283 | 207 |
| NA | 0.346 | 0.115 | 0.570 | 0.254 | 0.105 | 0.189 | 0.136 | 0.163 | 0.2 | 745 | 612 | 166 | 566 | 245 | 144 |
| NA | 0.431 | 0.072 | 0.419 | 0.238 | 0.116 | 0.202 | 0.148 | 0.083 | 0.3 | 641 | 789 | 165 | 432 | 179 | 128 |
| NA | 0.367 | 0.077 | 0.483 | 0.261 | 0.092 | 0.177 | 0.117 | 0.103 | 0.2 | 573 | 706 | 141 | 439 | 215 | 137 |
| NA | 0.320 | 0.086 | 0.540 | 0.235 | 0.079 | 0.176 | 0.092 | 0.121 | 0.2 | 849 | 760 | 200 | 689 | 347 | 216 |
| NA | 0.337 | 0.107 | 0.557 | 0.255 | 0.106 | 0.182 | 0.132 | 0.147 | 0.2 | 652 | 629 | 138 | 542 | 243 | 143 |
| NA | 0.294 | 0.085 | 0.505 | 0.175 | 0.072 | 0.133 | 0.085 | 0.094 | 0.2 | 897 | 831 | 253 | 728 | 300 | 180 |
| NA | 0.318 | 0.116 | 0.553 | 0.216 | 0.097 | 0.166 | 0.118 | 0.145 | 0.2 | 649 | 458 | 158 | 478 | 191 | 119 |
| NA | 0.305 | 0.073 | 0.536 | 0.162 | 0.062 | 0.126 | 0.069 | 0.084 | 0.3 | 721 | 580 | 212 | 510 | 218 | 151 |
| NA | 0.403 | 0.088 | 0.484 | 0.286 | 0.111 | 0.209 | 0.147 | 0.139 | 0.2 | 495 | 554 | 116 | 378 | 174 | 107 |
| NA | 0.318 | 0.084 | 0.522 | 0.202 | 0.075 | 0.145 | 0.097 | 0.093 | 0.2 | 896 | 845 | 238 | 671 | 291 | 182 |
| NA | 0.267 | 0.065 | 0.574 | 0.147 | 0.052 | 0.115 | 0.067 | 0.081 | 0.2 | 951 | 817 | 293 | 667 | 285 | 196 |
| NA | 0.251 | 0.054 | 0.533 | 0.102 | 0.033 | 0.079 | 0.057 | 0.068 | 0.3 | 1367 | 1045 | 525 | 870 | 437 | 317 |
| NA | 0.400 | 0.119 | 0.539 | 0.265 | 0.112 | 0.212 | 0.142 | 0.169 | 0.2 | 408 | 316 | 102 | 318 | 144 | 103 |
| NA | 0.286 | 0.082 | 0.578 | 0.180 | 0.055 | 0.121 | 0.081 | 0.118 | 0.2 | 852 | 608 | 253 | 567 | 285 | 176 |
| NA | 0.313 | 0.105 | 0.575 | 0.203 | 0.083 | 0.149 | 0.099 | 0.133 | 0.2 | 744 | 518 | 185 | 550 | 227 | 144 |
| NA | 0.328 | 0.084 | 0.544 | 0.217 | 0.083 | 0.163 | 0.102 | 0.100 | 0.2 | 650 | 621 | 174 | 532 | 228 | 154 |
| NA | 0.343 | 0.072 | 0.447 | 0.212 | 0.078 | 0.171 | 0.086 | no D peak | 0.2 | 1069 | 1028 | 286 | 693 | 353 | 263 |
| NA | 0.358 | 0.111 | 0.593 | 0.258 | 0.096 | 0.187 | 0.125 | 0.155 | 0.2 | 693 | 544 | 175 | 506 | 235 | 152 |
| NA | 0.346 | 0.114 | 0.562 | 0.240 | 0.091 | 0.167 | 0.127 | 0.164 | 0.2 | 563 | 367 | 143 | 328 | 149 | 96 |
| NA | 0.249 | 0.068 | 0.548 | 0.125 | 0.045 | 0.102 | 0.051 | 0.074 | 0.3 | 983 | 729 | 330 | 805 | 344 | 248 |
| NA | 0.312 | 0.074 | 0.478 | 0.202 | 0.066 | 0.149 | 0.085 | 0.079 | 0.2 | 1174 | 1117 | 280 | 880 | 433 | 290 |
| NA | 0.326 | 0.095 | 0.550 | 0.221 | 0.092 | 0.169 | 0.119 | 0.120 | 0.2 | 808 | 754 | 197 | 684 | 291 | 174 |
| NA | 0.265 | 0.103 | 0.455 | 0.161 | 0.054 | 0.117 | 0.102 | 0.148 | 0.2 | 606 | 305 | 148 | 326 | 124 | 80 |
| NA | 0.335 | 0.081 | 0.543 | 0.208 | 0.078 | 0.160 | 0.101 | 0.096 | 0.2 | 903 | 794 | 225 | 618 | 311 | 202 |
| NA | 0.319 | 0.096 | 0.557 | 0.226 | 0.093 | 0.165 | 0.119 | 0.129 | 0.2 | 795 | 746 | 197 | 692 | 296 | 173 |
| NA | 0.335 | 0.075 | 0.527 | 0.195 | 0.077 | 0.159 | 0.096 | 0.104 | 0.2 | 888 | 757 | 227 | 689 | 291 | 200 |

| | | | | | | | | | | | | | | | |
|---|---|---|---|---|---|---|---|---|---|---|---|---|---|---|---|
| NA | 0.282 | 0.059 | 0.565 | 0.134 | 0.042 | 0.093 | 0.045 | 0.060 | 0.3 | 1277 | 1005 | 430 | 738 | 387 | 289 |
| NA | 0.434 | 0.074 | 0.409 | 0.245 | 0.117 | 0.217 | 0.162 | 0.115 | 0.2 | 457 | 571 | 105 | 329 | 139 | 87 |
| NA | 0.319 | 0.076 | 0.528 | 0.155 | 0.062 | 0.131 | 0.082 | 0.104 | 0.3 | 742 | 600 | 228 | 495 | 222 | 157 |
| NA | 0.384 | 0.124 | 0.551 | 0.264 | 0.113 | 0.208 | 0.153 | 0.182 | 0.2 | 445 | 326 | 104 | 318 | 129 | 86 |
| NA | 0.337 | 0.087 | 0.532 | 0.173 | 0.076 | 0.147 | 0.100 | 0.121 | 0.2 | 491 | 414 | 138 | 336 | 129 | 95 |
| NA | 0.422 | 0.105 | 0.495 | 0.248 | 0.106 | 0.202 | 0.139 | 0.150 | 0.2 | 612 | 491 | 135 | 418 | 175 | 120 |
| NA | 0.290 | 0.053 | 0.527 | 0.139 | 0.056 | 0.123 | 0.074 | 0.063 | 0.3 | 738 | 769 | 257 | 542 | 250 | 184 |
| NA | 0.332 | 0.077 | 0.525 | 0.207 | 0.085 | 0.170 | 0.093 | 0.096 | 0.2 | 891 | 820 | 238 | 644 | 310 | 221 |
| NA | 0.297 | 0.066 | 0.488 | 0.127 | 0.055 | 0.116 | 0.082 | 0.088 | 0.3 | 586 | 520 | 199 | 483 | 202 | 139 |
| NA | 0.370 | 0.140 | 0.464 | 0.245 | 0.099 | 0.176 | 0.116 | 0.186 | 0.2 | 486 | 255 | 96 | 264 | 116 | 73 |
| NA | 0.290 | 0.098 | 0.584 | 0.199 | 0.069 | 0.144 | 0.092 | 0.144 | 0.2 | 865 | 641 | 243 | 632 | 320 | 186 |
| NA | 0.297 | 0.120 | 0.525 | 0.209 | 0.078 | 0.163 | 0.082 | 0.152 | 0.2 | 619 | 468 | 154 | 454 | 234 | 127 |
| NA | 0.331 | 0.074 | 0.525 | 0.227 | 0.090 | 0.168 | 0.105 | 0.099 | 0.2 | 681 | 780 | 177 | 576 | 282 | 184 |
| NA | 0.298 | 0.090 | 0.543 | 0.175 | 0.068 | 0.121 | 0.073 | 0.110 | 0.2 | 763 | 577 | 202 | 566 | 251 | 156 |
| NA | 0.308 | 0.070 | 0.535 | 0.176 | 0.074 | 0.130 | 0.083 | 0.089 | 0.3 | 731 | 760 | 235 | 529 | 221 | 159 |
| NA | 0.326 | 0.092 | 0.577 | 0.226 | 0.080 | 0.176 | 0.105 | 0.106 | 0.2 | 895 | 702 | 204 | 615 | 316 | 197 |
| NA | 0.321 | 0.100 | 0.503 | 0.204 | 0.085 | 0.147 | 0.107 | 0.124 | 0.2 | 719 | 503 | 167 | 481 | 203 | 128 |
| NA | 0.355 | 0.140 | 0.562 | 0.267 | 0.117 | 0.190 | 0.151 | 0.172 | 0.2 | 713 | 525 | 162 | 512 | 232 | 138 |
| NA | 0.315 | 0.073 | 0.516 | 0.176 | 0.071 | 0.131 | 0.088 | 0.090 | 0.3 | 734 | 762 | 215 | 531 | 230 | 155 |
| NA | 0.330 | 0.110 | 0.571 | 0.229 | 0.091 | 0.162 | 0.110 | 0.157 | 0.2 | 712 | 446 | 195 | 437 | 192 | 129 |
| NA | 0.325 | 0.073 | 0.538 | 0.172 | 0.066 | 0.132 | 0.086 | 0.122 | 0.3 | 526 | 424 | 154 | 363 | 158 | 109 |
| NA | 0.313 | 0.069 | 0.515 | 0.158 | 0.060 | 0.119 | 0.079 | 0.092 | 0.3 | 816 | 768 | 245 | 563 | 270 | 176 |
| NA | 0.306 | 0.092 | 0.556 | 0.163 | 0.070 | 0.135 | 0.090 | 0.110 | 0.2 | 897 | 682 | 235 | 688 | 278 | 177 |
| NA | 0.335 | 0.062 | 0.464 | 0.220 | 0.072 | 0.150 | 0.107 | 0.052 | 0.2 | 773 | 965 | 201 | 529 | 308 | 199 |
| NA | 0.356 | 0.117 | 0.581 | 0.268 | 0.111 | 0.217 | 0.150 | 0.206 | 0.2 | 568 | 455 | 131 | 462 | 169 | 108 |
| NA | 0.273 | 0.058 | 0.523 | 0.158 | 0.048 | 0.116 | 0.059 | 0.083 | 0.3 | 702 | 686 | 242 | 556 | 258 | 177 |
| NA | 0.350 | 0.124 | 0.512 | 0.241 | 0.090 | 0.041 | 0.123 | 0.219 | 0.2 | 523 | 319 | 122 | 300 | 132 | 79 |
| NA | 0.351 | 0.089 | 0.569 | 0.234 | 0.087 | 0.169 | 0.104 | 0.154 | 0.2 | 660 | 486 | 158 | 463 | 204 | 143 |
| NA | 0.271 | 0.045 | 0.489 | 0.154 | 0.058 | 0.155 | 0.081 | 0.073 | 0.3 | 529 | 696 | 180 | 409 | 190 | 132 |
| NA | 0.357 | 0.153 | 0.513 | 0.272 | 0.119 | 0.206 | 0.164 | 0.255 | 0.2 | 560 | 280 | 105 | 341 | 137 | 87 |
| NA | 0.299 | 0.081 | 0.601 | 0.179 | 0.057 | 0.132 | 0.072 | 0.099 | 0.2 | 740 | 502 | 212 | 568 | 239 | 162 |
| NA | 0.329 | 0.111 | 0.570 | 0.239 | 0.091 | 0.169 | 0.132 | 0.189 | 0.2 | 684 | 444 | 188 | 424 | 171 | 121 |
| NA | 0.325 | 0.085 | 0.502 | 0.191 | 0.072 | 0.144 | 0.092 | 0.150 | 0.2 | 589 | 442 | 160 | 390 | 163 | 116 |
| NA | 0.294 | 0.093 | 0.612 | 0.189 | 0.064 | 0.154 | 0.086 | 0.122 | 0.2 | 851 | 651 | 245 | 646 | 325 | 205 |
| NA | 0.296 | 0.087 | 0.570 | 0.211 | 0.080 | 0.154 | 0.108 | 0.148 | 0.2 | 560 | 497 | 161 | 468 | 193 | 127 |
| NA | 0.273 | 0.075 | 0.527 | 0.139 | 0.054 | 0.105 | 0.065 | 0.098 | 0.3 | 784 | 594 | 245 | 585 | 250 | 164 |
| NA | 0.328 | 0.110 | 0.555 | 0.229 | 0.099 | 0.165 | 0.129 | 0.174 | 0.2 | 553 | 477 | 136 | 468 | 199 | 116 |
| NA | 0.299 | 0.093 | 0.562 | 0.194 | 0.073 | 0.130 | 0.091 | 0.124 | 0.2 | 759 | 544 | 206 | 519 | 236 | 148 |
| NA | 0.299 | 0.094 | 0.570 | 0.188 | 0.072 | 0.147 | 0.086 | 0.129 | 0.2 | 795 | 622 | 226 | 613 | 265 | 180 |
| NA | 0.281 | 0.056 | 0.525 | 0.142 | 0.041 | 0.101 | 0.059 | 0.072 | 0.3 | 1184 | 1057 | 378 | 806 | 396 | 276 |
| NA | 0.342 | 0.141 | 0.563 | 0.253 | 0.124 | 0.185 | 0.149 | 0.207 | 0.2 | 589 | 464 | 133 | 495 | 207 | 119 |
| NA | 0.297 | 0.070 | 0.529 | 0.163 | 0.063 | 0.146 | 0.076 | 0.090 | 0.2 | 927 | 808 | 261 | 669 | 292 | 197 |
| NA | 0.292 | 0.083 | 0.542 | 0.172 | 0.069 | 0.123 | 0.085 | 0.106 | 0.2 | 861 | 722 | 226 | 619 | 309 | 199 |
| NA | 0.288 | 0.068 | 0.529 | 0.167 | 0.061 | 0.123 | 0.078 | 0.097 | 0.2 | 703 | 689 | 205 | 551 | 235 | 150 |
| NA | 0.310 | 0.117 | 0.521 | 0.209 | 0.090 | 0.155 | 0.116 | 0.164 | 0.2 | 858 | 610 | 183 | 712 | 336 | 175 |
| NA | 0.281 | 0.066 | 0.538 | 0.155 | 0.052 | 0.103 | 0.069 | 0.102 | 0.3 | 756 | 599 | 241 | 532 | 229 | 165 |
| NA | 0.329 | 0.104 | 0.571 | 0.209 | 0.081 | 0.151 | 0.107 | 0.216 | 0.2 | 1041 | 678 | 261 | 708 | 390 | 264 |
| NA | 0.303 | 0.106 | 0.540 | 0.213 | 0.082 | 0.166 | 0.106 | 0.173 | 0.2 | 594 | 434 | 150 | 468 | 200 | 115 |
| NA | 0.312 | 0.090 | 0.524 | 0.188 | 0.076 | 0.141 | 0.081 | 0.117 | 0.2 | 779 | 501 | 207 | 540 | 230 | 149 |
| NA | 0.267 | 0.052 | 0.521 | 0.147 | 0.052 | 0.114 | 0.060 | 0.077 | 0.3 | 807 | 832 | 270 | 635 | 278 | 203 |
| NA | 0.338 | 0.119 | 0.383 | 0.249 | 0.087 | 0.152 | 0.092 | 0.152 | 0.2 | 1188 | 521 | 236 | 475 | 271 | 201 |
| NA | 0.287 | 0.065 | 0.500 | 0.161 | 0.060 | 0.110 | 0.066 | 0.082 | 0.3 | 749 | 769 | 241 | 563 | 262 | 177 |
| NA | 0.320 | 0.066 | 0.471 | 0.154 | 0.062 | 0.121 | 0.083 | 0.078 | 0.3 | 642 | 665 | 198 | 520 | 215 | 146 |
| NA | 0.313 | 0.115 | 0.558 | 0.197 | 0.088 | 0.144 | 0.114 | 0.149 | 0.2 | 661 | 492 | 166 | 529 | 222 | 132 |
| NA | 0.332 | 0.105 | 0.589 | 0.237 | 0.075 | 0.154 | 0.094 | 0.142 | 0.2 | 781 | 480 | 191 | 554 | 287 | 187 |
| NA | 0.315 | 0.091 | 0.563 | 0.190 | 0.079 | 0.138 | 0.073 | 0.127 | 0.2 | 628 | 455 | 179 | 432 | 182 | 125 |

| | | | | | | | | | | | | | | | |
|---|---|---|---|---|---|---|---|---|---|---|---|---|---|---|---|
| NA | 0.287 | 0.076 | 0.506 | 0.153 | 0.065 | 0.118 | 0.070 | 0.095 | 0.3 | 833 | 580 | 255 | 581 | 239 | 177 |
| NA | 0.398 | 0.092 | 0.502 | 0.302 | 0.125 | 0.215 | 0.150 | 0.144 | 0.2 | 539 | 545 | 133 | 385 | 171 | 119 |
| NA | 0.259 | 0.055 | 0.529 | 0.153 | 0.047 | 0.106 | 0.049 | 0.055 | 0.3 | 751 | 808 | 285 | 648 | 242 | 215 |
| NA | 0.358 | 0.162 | 0.559 | 0.284 | 0.134 | 0.190 | 0.161 | 0.258 | 0.2 | 458 | 243 | 96 | 282 | 109 | 75 |
| NA | 0.315 | 0.109 | 0.543 | 0.214 | 0.101 | 0.163 | 0.136 | 0.159 | 0.2 | 635 | 489 | 155 | 489 | 187 | 124 |
| NA | 0.290 | 0.082 | 0.527 | 0.209 | 0.080 | 0.140 | 0.099 | 0.096 | 0.2 | 535 | 546 | 157 | 436 | 187 | 122 |
| NA | 0.321 | 0.071 | 0.595 | 0.184 | 0.055 | 0.118 | 0.065 | 0.082 | 0.3 | 999 | 632 | 316 | 557 | 268 | 213 |
| NA | 0.263 | 0.108 | 0.522 | 0.183 | 0.068 | 0.122 | 0.084 | 0.297 | 0.2 | 882 | 572 | 231 | 596 | 349 | 185 |
| NA | 0.319 | 0.090 | 0.616 | 0.202 | 0.071 | 0.146 | 0.086 | 0.142 | 0.2 | 1010 | 665 | 257 | 611 | 327 | 202 |
| NA | 0.201 | 0.052 | 0.452 | 0.080 | 0.031 | 0.062 | 0.031 | 0.074 | 0.4 | 1112 | 583 | 496 | 641 | 282 | 206 |
| NA | 0.283 | 0.103 | 0.469 | 0.180 | 0.071 | 0.127 | 0.095 | 0.174 | 0.2 | 708 | 481 | 185 | 448 | 222 | 140 |
| NA | 0.207 | 0.086 | 0.336 | 0.139 | 0.040 | 0.079 | 0.073 | 0.151 | 0.2 | 916 | 559 | 244 | 584 | 328 | 166 |
| NA | 0.287 | 0.097 | 0.555 | 0.199 | 0.076 | 0.143 | 0.094 | 0.149 | 0.2 | 548 | 363 | 164 | 338 | 137 | 96 |
| NA | 0.215 | 0.075 | 0.422 | 0.125 | 0.047 | 0.082 | 0.048 | 0.125 | 0.3 | 801 | 512 | 263 | 562 | 230 | 150 |
| NA | 0.320 | 0.108 | 0.540 | 0.237 | 0.099 | 0.174 | 0.114 | 0.142 | 0.2 | 585 | 397 | 127 | 383 | 150 | 95 |
| NA | 0.227 | 0.099 | 0.321 | 0.169 | 0.053 | 0.100 | 0.073 | 0.143 | 0.2 | 825 | 509 | 199 | 454 | 229 | 132 |
| NA | 0.334 | 0.137 | 0.496 | 0.274 | 0.109 | 0.190 | 0.138 | 0.260 | 0.2 | 519 | 406 | 123 | 404 | 178 | 113 |
| NA | 0.207 | 0.059 | 0.475 | 0.089 | 0.035 | 0.066 | 0.036 | 0.085 | 0.3 | 941 | 514 | 342 | 565 | 265 | 172 |
| NA | 0.267 | 0.106 | 0.500 | 0.168 | 0.069 | 0.137 | 0.092 | 0.207 | 0.2 | 759 | 474 | 197 | 545 | 248 | 138 |
| NA | 0.292 | 0.097 | 0.557 | 0.215 | 0.084 | 0.143 | 0.104 | 0.157 | 0.2 | 687 | 588 | 181 | 553 | 243 | 142 |
| NA | 0.273 | 0.107 | 0.504 | 0.178 | 0.065 | 0.135 | 0.088 | 0.173 | 0.2 | 708 | 503 | 183 | 469 | 228 | 135 |
| NA | 0.117 | 0.034 | 0.281 | 0.039 | 0.017 | 0.030 | 0.019 | 0.087 | 0.3 | 3511 | 1376 | 1280 | 1415 | 771 | 532 |
| NA | 0.280 | 0.100 | 0.517 | 0.180 | 0.074 | 0.133 | 0.082 | 0.119 | 0.2 | 696 | 494 | 183 | 500 | 240 | 151 |
| NA | 0.248 | 0.040 | 0.583 | 0.093 | 0.030 | 0.072 | 0.043 | 0.053 | 0.3 | 1307 | 1067 | 517 | 848 | 377 | 277 |
| NA | 0.266 | 0.066 | 0.579 | 0.146 | 0.045 | 0.100 | 0.069 | 0.084 | 0.3 | 1185 | 819 | 389 | 818 | 394 | 254 |
| NA | 0.221 | 0.041 | 0.533 | 0.082 | 0.026 | 0.063 | 0.027 | 0.049 | 0.3 | 1553 | 1226 | 660 | 944 | 441 | 348 |
| NA | 0.220 | 0.055 | 0.498 | 0.092 | 0.032 | 0.067 | 0.030 | 0.070 | 0.3 | 1478 | 797 | 565 | 860 | 405 | 286 |
| NA | 0.265 | 0.083 | 0.556 | 0.152 | 0.059 | 0.114 | 0.076 | 0.126 | 0.2 | 724 | 450 | 207 | 523 | 207 | 142 |
| NA | 0.169 | 0.046 | 0.380 | 0.071 | 0.026 | 0.049 | 0.030 | 0.085 | 0.3 | 985 | 687 | 395 | 661 | 282 | 182 |
| NA | 0.270 | 0.117 | 0.454 | 0.200 | 0.080 | 0.130 | 0.142 | 0.227 | 0.2 | 617 | 365 | 169 | 388 | 171 | 113 |
| NA | 0.297 | 0.107 | 0.433 | 0.208 | 0.077 | 0.146 | 0.118 | 0.202 | 0.2 | 826 | 622 | 213 | 634 | 296 | 173 |
| NA | 0.266 | 0.110 | 0.478 | 0.196 | 0.080 | 0.120 | 0.098 | 0.196 | 0.2 | 550 | 355 | 150 | 386 | 175 | 119 |
| NA | 0.191 | 0.040 | 0.450 | 0.073 | 0.026 | 0.058 | 0.028 | 0.049 | 0.4 | 1078 | 903 | 506 | 733 | 342 | 248 |
| NA | 0.222 | 0.073 | 0.460 | 0.111 | 0.044 | 0.081 | 0.047 | 0.118 | 0.3 | 1107 | 701 | 369 | 675 | 305 | 200 |
| NA | 0.272 | 0.071 | 0.553 | 0.145 | 0.054 | 0.109 | 0.060 | 0.104 | 0.3 | 918 | 670 | 285 | 597 | 260 | 177 |
| NA | 0.272 | 0.074 | 0.537 | 0.161 | 0.061 | 0.113 | 0.066 | 0.095 | 0.2 | 930 | 694 | 265 | 555 | 289 | 177 |
| NA | 0.237 | 0.048 | 0.590 | 0.083 | 0.028 | 0.068 | 0.029 | 0.062 | 0.3 | 2062 | 1173 | 809 | 1022 | 611 | 479 |
| 2 | 0.039 | 0.019 | 0.022 | 0.048 | 0.006 | 0.018 | 0.010 | 0.047 | 0.6 | 2151 | 1264 | 1301 | 1007 | 484 | 434 |
| 2 | 0.038 | 0.018 | 0.022 | 0.037 | 0.017 | 0.014 | 0.016 | 0.041 | 0.7 | 4369 | 4141 | 2900 | 3144 | 1624 | 1254 |
| NA | 0.284 | 0.089 | 0.586 | 0.185 | 0.065 | 0.127 | 0.079 | 0.124 | 0.2 | 1102 | 755 | 300 | 754 | 382 | 229 |
| NA | 0.268 | 0.112 | 0.494 | 0.187 | 0.063 | 0.121 | 0.072 | 0.129 | 0.2 | 667 | 448 | 175 | 466 | 227 | 129 |
| NA | 0.255 | 0.056 | 0.528 | 0.093 | 0.044 | 0.079 | 0.050 | 0.069 | 0.3 | 1043 | 725 | 402 | 634 | 289 | 218 |
| NA | 0.240 | 0.048 | 0.552 | 0.098 | 0.035 | 0.070 | 0.028 | 0.067 | 0.3 | 1187 | 708 | 482 | 725 | 348 | 274 |
| NA | 0.146 | 0.053 | 0.284 | 0.084 | 0.027 | 0.037 | 0.032 | 0.079 | 0.4 | 917 | 574 | 375 | 593 | 271 | 187 |
| NA | 0.159 | 0.046 | 0.386 | 0.070 | 0.027 | 0.044 | 0.023 | 0.054 | 0.3 | 1133 | 799 | 467 | 767 | 378 | 236 |
| NA | 0.216 | 0.082 | 0.425 | 0.130 | 0.048 | 0.077 | 0.045 | 0.179 | 0.3 | 904 | 516 | 278 | 568 | 319 | 178 |
| NA | 0.269 | 0.103 | 0.483 | 0.196 | 0.084 | 0.133 | 0.118 | 0.229 | 0.2 | 523 | 360 | 139 | 363 | 171 | 105 |
| NA | 0.262 | 0.076 | 0.541 | 0.129 | 0.051 | 0.091 | 0.056 | 0.094 | 0.2 | 1220 | 708 | 371 | 763 | 384 | 241 |
| NA | 0.252 | 0.073 | 0.516 | 0.153 | 0.055 | 0.097 | 0.057 | 0.091 | 0.3 | 718 | 483 | 221 | 509 | 213 | 139 |
| NA | 0.188 | 0.068 | 0.333 | 0.142 | 0.041 | 0.056 | 0.058 | 0.145 | 0.3 | 967 | 534 | 275 | 552 | 262 | 153 |
| NA | 0.211 | 0.114 | 0.350 | 0.116 | 0.037 | 0.071 | 0.053 | 0.145 | 0.2 | 1150 | 668 | 305 | 631 | 310 | 181 |
| NA | 0.274 | 0.106 | 0.507 | 0.186 | 0.076 | 0.119 | 0.104 | 0.199 | 0.2 | 671 | 468 | 178 | 519 | 235 | 133 |
| NA | 0.206 | 0.051 | 0.507 | 0.084 | 0.029 | 0.059 | 0.030 | 0.061 | 0.3 | 1033 | 612 | 435 | 656 | 299 | 215 |
| NA | 0.272 | 0.110 | 0.445 | 0.196 | 0.102 | 0.121 | 0.097 | 0.200 | 0.2 | 729 | 392 | 188 | 419 | 183 | 115 |
| NA | 0.264 | 0.077 | 0.496 | 0.164 | 0.073 | 0.107 | 0.056 | 0.091 | 0.2 | 779 | 624 | 221 | 551 | 254 | 159 |
| NA | 0.295 | 0.134 | 0.451 | 0.242 | 0.094 | 0.140 | 0.128 | 0.213 | 0.2 | 621 | 340 | 148 | 409 | 174 | 106 |

| | | | | | | | | | | | | | | | | |
|---|---|---|---|---|---|---|---|---|---|---|---|---|---|---|---|---|
| NA | 0.254 | 0.075 | 0.532 | 0.127 | 0.055 | 0.101 | 0.072 | 0.122 | 0.3 | 738 | 508 | 228 | 553 | 225 | 150 |
| NA | 0.245 | 0.062 | 0.525 | 0.142 | 0.053 | 0.096 | 0.052 | 0.076 | 0.3 | 1231 | 954 | 394 | 922 | 457 | 288 |
| NA | 0.173 | 0.050 | 0.378 | 0.082 | 0.025 | 0.047 | 0.035 | 0.072 | 0.3 | 870 | 624 | 340 | 601 | 288 | 179 |
| NA | 0.258 | 0.102 | 0.435 | 0.186 | 0.071 | 0.107 | 0.061 | 0.148 | 0.2 | 677 | 403 | 168 | 468 | 204 | 117 |
| NA | 0.189 | 0.074 | 0.363 | 0.108 | 0.041 | 0.062 | 0.058 | 0.174 | 0.3 | 987 | 536 | 302 | 634 | 297 | 185 |
| NA | 0.308 | 0.116 | 0.582 | 0.229 | 0.093 | 0.149 | 0.084 | 0.145 | 0.2 | 602 | 407 | 144 | 437 | 193 | 118 |
| NA | 0.222 | 0.040 | 0.539 | 0.061 | 0.021 | 0.053 | 0.023 | 0.046 | 0.4 | 2377 | 1421 | 1086 | 1171 | 674 | 588 |
| NA | 0.154 | 0.036 | 0.358 | 0.061 | 0.027 | 0.039 | 0.020 | 0.051 | 0.4 | 1242 | 911 | 579 | 748 | 361 | 267 |
| NA | 0.260 | 0.053 | 0.519 | 0.144 | 0.046 | 0.093 | 0.053 | 0.064 | 0.3 | 1015 | 897 | 347 | 701 | 313 | 232 |
| NA | 0.185 | 0.108 | 0.210 | 0.136 | 0.036 | 0.048 | 0.048 | 0.117 | 0.2 | 2098 | 1039 | 532 | 1413 | 828 | 406 |
| NA | 0.140 | 0.035 | 0.336 | 0.051 | 0.023 | 0.038 | 0.021 | 0.048 | 0.4 | 787 | 534 | 406 | 578 | 250 | 193 |
| NA | 0.264 | 0.089 | 0.551 | 0.168 | 0.064 | 0.112 | 0.060 | 0.117 | 0.2 | 1081 | 620 | 314 | 688 | 349 | 214 |
| NA | 0.301 | 0.087 | 0.525 | 0.216 | 0.081 | 0.136 | 0.096 | 0.128 | 0.2 | 566 | 472 | 159 | 390 | 181 | 112 |
| NA | 0.242 | 0.064 | 0.522 | 0.140 | 0.048 | 0.090 | 0.047 | 0.074 | 0.3 | 812 | 635 | 287 | 550 | 272 | 167 |
| NA | 0.247 | 0.085 | 0.404 | 0.165 | 0.052 | 0.096 | 0.074 | 0.152 | 0.3 | 770 | 500 | 221 | 494 | 260 | 193 |
| NA | 0.296 | 0.101 | 0.431 | 0.237 | 0.095 | 0.149 | 0.104 | 0.145 | 0.2 | 750 | 578 | 176 | 546 | 244 | 149 |
| NA | 0.278 | 0.100 | 0.524 | 0.201 | 0.077 | 0.131 | 0.084 | 0.156 | 0.2 | 602 | 430 | 168 | 412 | 187 | 116 |
| NA | 0.250 | 0.066 | 0.574 | 0.115 | 0.040 | 0.078 | 0.034 | 0.063 | 0.3 | 1905 | 1057 | 627 | 1003 | 591 | 388 |
| NA | 0.310 | 0.105 | 0.562 | 0.234 | 0.100 | 0.154 | 0.109 | 0.163 | 0.2 | 557 | 418 | 148 | 361 | 172 | 113 |
| NA | 0.408 | 0.152 | 0.594 | 0.377 | 0.163 | 0.279 | 0.201 | 0.256 | 0.2 | 402 | 325 | 95 | 322 | 146 | 95 |
| NA | 0.344 | 0.101 | 0.607 | 0.225 | 0.095 | 0.147 | 0.097 | 0.139 | 0.2 | 788 | 523 | 208 | 466 | 218 | 159 |
| NA | 0.310 | 0.086 | 0.572 | 0.240 | 0.065 | 0.136 | 0.088 | 0.122 | 0.3 | 711 | 478 | 221 | 362 | 208 | 158 |
| NA | 0.195 | 0.089 | 0.317 | 0.137 | 0.043 | 0.063 | 0.074 | 0.151 | 0.2 | 865 | 456 | 235 | 475 | 251 | 146 |
| NA | 0.321 | 0.105 | 0.580 | 0.237 | 0.101 | 0.153 | 0.098 | 0.126 | 0.2 | 834 | 639 | 204 | 597 | 292 | 187 |
| NA | 0.283 | 0.096 | 0.534 | 0.199 | 0.083 | 0.124 | 0.077 | 0.136 | 0.2 | 740 | 518 | 200 | 500 | 241 | 147 |
| NA | 0.294 | 0.122 | 0.494 | 0.219 | 0.094 | 0.141 | 0.109 | 0.240 | 0.2 | 727 | 464 | 163 | 518 | 254 | 140 |
| NA | 0.313 | 0.106 | 0.581 | 0.225 | 0.095 | 0.149 | 0.103 | 0.130 | 0.2 | 689 | 504 | 168 | 491 | 232 | 150 |
| NA | 0.266 | 0.102 | 0.488 | 0.167 | 0.066 | 0.112 | 0.076 | 0.154 | 0.2 | 667 | 371 | 194 | 439 | 184 | 119 |
| NA | 0.265 | 0.117 | 0.430 | 0.213 | 0.091 | 0.120 | 0.100 | 0.156 | 0.2 | 687 | 488 | 158 | 540 | 258 | 147 |
| NA | 0.277 | 0.081 | 0.567 | 0.173 | 0.070 | 0.117 | 0.074 | 0.114 | 0.2 | 754 | 557 | 230 | 511 | 239 | 163 |
| NA | 0.262 | 0.085 | 0.424 | 0.169 | 0.064 | 0.120 | 0.058 | 0.097 | 0.3 | 659 | 513 | 206 | 499 | 248 | 148 |
| NA | 0.198 | 0.054 | 0.403 | 0.099 | 0.036 | 0.059 | 0.028 | 0.047 | 0.3 | 866 | 680 | 324 | 549 | 271 | 177 |
| NA | 0.247 | 0.102 | 0.381 | 0.192 | 0.069 | 0.124 | 0.108 | 0.093 | 0.2 | 1331 | 884 | 329 | 959 | 485 | 276 |
| NA | 0.144 | 0.061 | 0.175 | 0.099 | 0.019 | 0.046 | 0.156 | 0.043 | 0.3 | 1627 | 896 | 563 | 1033 | 572 | 341 |
| NA | 0.203 | 0.085 | 0.285 | 0.154 | 0.051 | 0.100 | 0.144 | 0.100 | 0.3 | 932 | 496 | 287 | 529 | 251 | 146 |
| NA | 0.186 | 0.066 | 0.298 | 0.126 | 0.033 | 0.077 | 0.182 | 0.066 | 0.3 | 932 | 592 | 333 | 596 | 268 | 164 |
| NA | 0.190 | 0.063 | 0.319 | 0.090 | 0.025 | 0.072 | 0.059 | 0.065 | 0.3 | 1015 | 571 | 367 | 623 | 260 | 174 |
| NA | 0.191 | 0.046 | 0.447 | 0.067 | 0.027 | 0.057 | 0.058 | 0.088 | 0.4 | 1585 | 942 | 682 | 918 | 432 | 320 |
| NA | 0.196 | 0.053 | 0.375 | 0.096 | 0.029 | 0.073 | 0.064 | 0.043 | 0.4 | 855 | 539 | 368 | 497 | 220 | 152 |
| NA | 0.217 | 0.097 | 0.341 | 0.143 | 0.041 | 0.113 | 0.127 | 0.097 | 0.2 | 889 | 357 | 243 | 397 | 171 | 109 |
| NA | 0.157 | 0.066 | 0.274 | 0.097 | 0.030 | 0.070 | 0.148 | 0.089 | 0.3 | 1334 | 750 | 419 | 861 | 487 | 255 |
| NA | 0.151 | 0.081 | 0.194 | 0.111 | 0.035 | 0.072 | 0.133 | 0.067 | 0.3 | 1320 | 792 | 399 | 980 | 549 | 329 |
| NA | 0.164 | 0.049 | 0.358 | 0.084 | 0.028 | 0.065 | 0.102 | 0.042 | 0.3 | 1244 | 760 | 453 | 730 | 330 | 216 |
| NA | 0.284 | 0.119 | 0.350 | 0.227 | 0.078 | 0.181 | 0.067 | 0.169 | 0.2 | 803 | 443 | 189 | 466 | 204 | 125 |
| NA | 0.255 | 0.120 | 0.332 | 0.193 | 0.063 | 0.149 | 0.123 | 0.163 | 0.2 | 920 | 461 | 217 | 520 | 243 | 136 |
| NA | 0.180 | 0.078 | 0.219 | 0.131 | 0.029 | 0.064 | 0.132 | 0.062 | 0.3 | 1424 | 701 | 487 | 931 | 437 | 291 |
| NA | 0.182 | 0.089 | 0.230 | 0.145 | 0.037 | 0.075 | 0.128 | 0.077 | 0.3 | 1239 | 643 | 350 | 789 | 380 | 209 |
| NA | 0.219 | 0.091 | 0.327 | 0.155 | 0.047 | 0.096 | 0.113 | 0.107 | 0.3 | 906 | 449 | 262 | 494 | 246 | 156 |
| NA | 0.152 | 0.049 | 0.310 | 0.086 | 0.019 | 0.062 | 0.137 | 0.040 | 0.4 | 1006 | 661 | 422 | 613 | 279 | 188 |
| NA | 0.163 | 0.067 | 0.280 | 0.099 | 0.026 | 0.080 | 0.085 | 0.055 | 0.3 | 963 | 503 | 299 | 535 | 282 | 155 |
| NA | 0.182 | 0.090 | 0.238 | 0.147 | 0.045 | 0.085 | 0.107 | 0.076 | 0.3 | 1205 | 695 | 323 | 806 | 410 | 268 |
| NA | 0.132 | 0.046 | 0.240 | 0.070 | 0.021 | 0.047 | 0.099 | 0.063 | 0.4 | 1208 | 723 | 485 | 723 | 364 | 234 |
| NA | 0.177 | 0.081 | 0.244 | 0.134 | 0.041 | 0.076 | 0.078 | 0.078 | 0.3 | 1092 | 602 | 344 | 722 | 370 | 221 |
| NA | 0.183 | 0.069 | 0.287 | 0.117 | 0.037 | 0.078 | 0.113 | 0.058 | 0.3 | 1175 | 705 | 375 | 759 | 371 | 233 |
| NA | 0.122 | 0.046 | 0.188 | 0.065 | 0.017 | 0.044 | 0.043 | 0.026 | 0.4 | 1449 | 934 | 559 | 939 | 443 | 315 |
| NA | 0.126 | 0.043 | 0.223 | 0.066 | 0.020 | 0.040 | 0.061 | 0.039 | 0.4 | 1563 | 1026 | 640 | 998 | 484 | 325 |

| | | | | | | | | | | | | | | | |
|---|---|---|---|---|---|---|---|---|---|---|---|---|---|---|---|
| NA | 0.271 | 0.091 | 0.495 | 0.186 | 0.065 | 0.139 | 0.084 | 0.102 | 0.2 | 992 | 760 | 272 | 692 | 298 | 191 |
| NA | 0.186 | 0.048 | 0.363 | 0.097 | 0.027 | 0.059 | 0.066 | 0.040 | 0.4 | 1204 | 901 | 493 | 742 | 358 | 245 |
| NA | 0.125 | 0.060 | 0.114 | 0.092 | 0.020 | 0.044 | 0.107 | 0.037 | 0.4 | 1862 | 1183 | 814 | 1306 | 829 | 492 |
| NA | 0.121 | 0.048 | 0.184 | 0.074 | 0.014 | 0.044 | 0.109 | 0.074 | 0.4 | 1791 | 1044 | 735 | 1175 | 606 | 377 |
| NA | 0.268 | 0.121 | 0.388 | 0.225 | 0.075 | 0.162 | 0.061 | 0.191 | 0.2 | 817 | 378 | 189 | 437 | 203 | 114 |
| NA | 0.204 | 0.076 | 0.377 | 0.118 | 0.042 | 0.099 | 0.211 | 0.069 | 0.3 | 797 | 422 | 278 | 466 | 196 | 129 |
| NA | 0.099 | 0.050 | 0.087 | 0.076 | 0.019 | 0.031 | 0.056 | 0.030 | 0.4 | 3336 | 1951 | 1235 | 2412 | 1471 | 938 |
| 779 | 0.172 | 0.043 | 0.438 | 0.063 | 0.016 | 0.049 | 0.029 | 0.041 | 0.4 | 1647 | 1030 | 756 | 1164 | 582 | 357 |
| NA | 0.214 | 0.063 | 0.422 | 0.107 | 0.031 | 0.085 | 0.044 | 0.071 | 0.3 | 810 | 495 | 297 | 486 | 206 | 146 |
| NA | 0.155 | 0.042 | 0.254 | 0.083 | 0.017 | 0.056 | 0.048 | 0.042 | 0.4 | 939 | 816 | 387 | 596 | 270 | 174 |
| NA | 0.169 | 0.050 | 0.324 | 0.092 | 0.023 | 0.063 | 0.093 | 0.053 | 0.4 | 892 | 673 | 365 | 596 | 260 | 174 |
| NA | 0.210 | 0.086 | 0.311 | 0.149 | 0.039 | 0.084 | 0.183 | 0.114 | 0.3 | 895 | 487 | 254 | 537 | 270 | 166 |
| NA | 0.221 | 0.077 | 0.430 | 0.141 | 0.037 | 0.088 | 0.150 | 0.075 | 0.3 | 1083 | 761 | 323 | 717 | 351 | 202 |
| NA | 0.203 | 0.075 | 0.392 | 0.116 | 0.038 | 0.095 | 0.055 | 0.123 | 0.3 | 847 | 487 | 299 | 518 | 231 | 156 |
| NA | 0.203 | 0.048 | 0.483 | 0.074 | 0.018 | 0.059 | 0.102 | 0.040 | 0.4 | 2158 | 1206 | 980 | 1170 | 601 | 444 |
| NA | 0.274 | 0.103 | 0.395 | 0.210 | 0.073 | 0.132 | 0.116 | 0.095 | 0.2 | 913 | 644 | 229 | 606 | 304 | 171 |
| NA | 0.111 | 0.028 | 0.261 | 0.038 | 0.019 | 0.039 | 0.026 | 0.021 | 0.6 | 2453 | 1529 | 1533 | 1325 | 809 | 687 |
| 3089 | 0.270 | 0.091 | 0.565 | 0.155 | 0.053 | 0.138 | 0.144 | 0.104 | 0.2 | 1039 | 658 | 297 | 802 | 412 | 233 |
| NA | 0.200 | 0.069 | 0.461 | 0.081 | 0.025 | 0.066 | 0.053 | 0.092 | 0.3 | 1216 | 674 | 451 | 833 | 387 | 239 |
| NA | 0.200 | 0.078 | 0.391 | 0.122 | 0.036 | 0.080 | 0.145 | 0.060 | 0.2 | 1480 | 863 | 413 | 1009 | 475 | 265 |
| 1892 | 0.220 | 0.096 | 0.341 | 0.126 | 0.034 | 0.072 | 0.175 | 0.056 | 0.2 | 1533 | 1035 | 412 | 969 | 529 | 294 |
| NA | 0.234 | 0.116 | 0.336 | 0.183 | 0.058 | 0.113 | 0.134 | 0.111 | 0.2 | 1051 | 562 | 253 | 693 | 332 | 184 |
| NA | 0.252 | 0.102 | 0.397 | 0.161 | 0.051 | 0.122 | 0.112 | 0.089 | 0.2 | 1020 | 539 | 260 | 606 | 282 | 155 |
| NA | 0.205 | 0.115 | 0.294 | 0.133 | 0.036 | 0.068 | 0.128 | 0.057 | 0.2 | 1688 | 861 | 397 | 929 | 477 | 301 |
| NA | 0.204 | 0.064 | 0.354 | 0.102 | 0.033 | 0.070 | 0.034 | 0.073 | 0.3 | 957 | 533 | 330 | 527 | 217 | 150 |
| NA | 0.293 | 0.085 | 0.535 | 0.168 | 0.063 | 0.129 | 0.130 | 0.089 | 0.2 | 981 | 714 | 264 | 668 | 304 | 202 |
| NA | 0.229 | 0.064 | 0.412 | 0.102 | 0.028 | 0.090 | 0.045 | 0.079 | 0.3 | 735 | 448 | 250 | 485 | 182 | 129 |
| NA | 0.220 | 0.099 | 0.395 | 0.163 | 0.062 | 0.113 | 0.114 | 0.085 | 0.2 | 1015 | 522 | 257 | 624 | 279 | 164 |
| NA | 0.156 | 0.089 | 0.168 | 0.133 | 0.026 | 0.062 | 0.105 | 0.063 | 0.3 | 1590 | 921 | 461 | 1126 | 692 | 360 |
| NA | 0.294 | 0.109 | 0.515 | 0.182 | 0.063 | 0.155 | 0.052 | 0.139 | 0.2 | 701 | 376 | 172 | 442 | 183 | 114 |
| NA | 0.121 | 0.035 | 0.240 | 0.059 | 0.016 | 0.041 | 0.035 | 0.091 | 0.5 | 1236 | 863 | 695 | 764 | 389 | 299 |
| NA | 0.357 | 0.116 | 0.559 | 0.280 | 0.110 | 0.231 | 0.128 | 0.170 | 0.2 | 699 | 562 | 159 | 503 | 229 | 140 |
| NA | 0.211 | 0.059 | 0.412 | 0.106 | 0.031 | 0.077 | 0.029 | 0.073 | 0.3 | 991 | 723 | 389 | 622 | 304 | 215 |
| NA | 0.164 | 0.049 | 0.318 | 0.078 | 0.027 | 0.063 | 0.027 | 0.047 | 0.4 | 915 | 612 | 385 | 555 | 245 | 176 |
| NA | 0.140 | 0.059 | 0.209 | 0.093 | 0.017 | 0.054 | 0.082 | 0.055 | 0.3 | 1476 | 786 | 492 | 971 | 549 | 329 |
| NA | 0.281 | 0.089 | 0.575 | 0.168 | 0.060 | 0.129 | 0.073 | 0.100 | 0.2 | 1291 | 771 | 327 | 876 | 404 | 239 |
| NA | 0.257 | 0.117 | 0.399 | 0.203 | 0.065 | 0.144 | 0.119 | 0.109 | 0.2 | 834 | 497 | 204 | 501 | 244 | 146 |
| NA | 0.274 | 0.090 | 0.496 | 0.165 | 0.068 | 0.140 | 0.071 | 0.097 | 0.2 | 806 | 487 | 234 | 473 | 203 | 146 |
| NA | 0.288 | 0.115 | 0.492 | 0.216 | 0.066 | 0.143 | 0.179 | 0.146 | 0.2 | 782 | 472 | 189 | 503 | 231 | 138 |
| NA | 0.328 | 0.074 | 0.443 | 0.215 | 0.070 | 0.150 | 0.031 | 0.111 | 0.3 | 1034 | 996 | 322 | 677 | 309 | 209 |
| NA | 0.320 | 0.081 | 0.583 | 0.204 | 0.079 | 0.182 | 0.160 | 0.108 | 0.2 | 970 | 668 | 251 | 595 | 186 | 163 |
| NA | 0.305 | 0.106 | 0.549 | 0.219 | 0.084 | 0.161 | 0.200 | 0.114 | 0.2 | 925 | 649 | 213 | 626 | 298 | 171 |
| NA | 0.311 | 0.106 | 0.594 | 0.231 | 0.079 | 0.174 | 0.111 | 0.140 | 0.2 | 1022 | 692 | 238 | 768 | 381 | 231 |
| NA | 0.243 | 0.080 | 0.430 | 0.148 | 0.046 | 0.107 | 0.120 | 0.071 | 0.2 | 894 | 707 | 257 | 581 | 266 | 169 |
| NA | 0.264 | 0.074 | 0.442 | 0.145 | 0.040 | 0.106 | 0.112 | 0.082 | 0.3 | 860 | 595 | 277 | 537 | 234 | 158 |
| NA | 0.269 | 0.062 | 0.508 | 0.127 | 0.041 | 0.106 | 0.050 | 0.081 | 0.3 | 954 | 725 | 340 | 588 | 318 | 222 |
| NA | 0.311 | 0.120 | 0.517 | 0.230 | 0.078 | 0.162 | 0.090 | 0.145 | 0.2 | 754 | 455 | 186 | 506 | 220 | 133 |
| NA | 0.265 | 0.091 | 0.491 | 0.164 | 0.060 | 0.131 | 0.075 | 0.094 | 0.2 | 909 | 612 | 246 | 602 | 260 | 163 |
| NA | 0.281 | 0.088 | 0.548 | 0.172 | 0.061 | 0.140 | 0.041 | 0.103 | 0.2 | 888 | 575 | 247 | 635 | 264 | 168 |
| 3504 | 0.247 | 0.107 | 0.428 | 0.186 | 0.059 | 0.133 | 0.162 | 0.103 | 0.2 | 1035 | 625 | 214 | 655 | 315 | 175 |
| NA | 0.241 | 0.101 | 0.414 | 0.154 | 0.048 | 0.101 | 0.160 | 0.085 | 0.2 | 1642 | 970 | 443 | 950 | 544 | 311 |
| NA | 0.279 | 0.073 | 0.564 | 0.158 | 0.048 | 0.116 | 0.081 | 0.080 | 0.2 | 954 | 667 | 278 | 699 | 300 | 189 |
| NA | 0.332 | 0.082 | 0.525 | 0.191 | 0.060 | 0.152 | 0.051 | 0.118 | 0.2 | 771 | 556 | 215 | 504 | 225 | 151 |
| NA | 0.280 | 0.097 | 0.513 | 0.191 | 0.053 | 0.130 | 0.046 | 0.097 | 0.2 | 729 | 466 | 209 | 454 | 208 | 129 |
| NA | 0.314 | 0.120 | 0.583 | 0.234 | 0.080 | 0.169 | 0.092 | 0.177 | 0.2 | 728 | 445 | 177 | 464 | 203 | 129 |
| NA | 0.310 | 0.103 | 0.514 | 0.189 | 0.065 | 0.135 | 0.039 | 0.137 | 0.2 | 819 | 490 | 212 | 497 | 231 | 137 |

| | | | | | | | | | | | | | | | |
|---|---|---|---|---|---|---|---|---|---|---|---|---|---|---|---|
| NA | 0.239 | 0.059 | 0.575 | 0.102 | 0.033 | 0.082 | 0.043 | 0.059 | 0.3 | 1909 | 1163 | 708 | 1072 | 593 | 418 |
| NA | 0.305 | 0.088 | 0.512 | 0.178 | 0.068 | 0.166 | 0.056 | 0.121 | 0.2 | 711 | 489 | 196 | 477 | 207 | 133 |
| NA | 0.347 | 0.094 | 0.548 | 0.248 | 0.089 | 0.191 | 0.059 | 0.116 | 0.2 | 734 | 561 | 176 | 419 | 171 | 117 |
| NA | 0.332 | 0.131 | 0.604 | 0.246 | 0.084 | 0.180 | 0.064 | 0.167 | 0.2 | 866 | 503 | 197 | 605 | 270 | 153 |
| NA | 0.334 | 0.076 | 0.592 | 0.201 | 0.065 | 0.163 | 0.033 | 0.103 | 0.2 | 1048 | 863 | 294 | 710 | 376 | 262 |
| NA | 0.244 | 0.082 | 0.481 | 0.119 | 0.039 | 0.098 | 0.033 | 0.082 | 0.3 | 804 | 433 | 264 | 515 | 198 | 142 |
| NA | 0.297 | 0.098 | 0.527 | 0.185 | 0.064 | 0.148 | 0.039 | 0.133 | 0.2 | 689 | 406 | 203 | 403 | 169 | 119 |
| NA | 0.278 | 0.096 | 0.502 | 0.152 | 0.052 | 0.133 | 0.084 | 0.111 | 0.3 | 649 | 318 | 211 | 356 | 158 | 107 |
| NA | 0.351 | 0.136 | 0.529 | 0.288 | 0.115 | 0.211 | 0.052 | 0.177 | 0.2 | 708 | 459 | 150 | 437 | 192 | 120 |
| NA | 0.318 | 0.118 | 0.528 | 0.256 | 0.088 | 0.174 | 0.074 | 0.133 | 0.2 | 759 | 608 | 172 | 576 | 249 | 150 |
| NA | 0.280 | 0.095 | 0.502 | 0.182 | 0.076 | 0.144 | 0.089 | 0.096 | 0.2 | 764 | 520 | 198 | 525 | 193 | 137 |
| NA | 0.282 | 0.069 | 0.553 | 0.172 | 0.053 | 0.127 | 0.041 | 0.074 | 0.2 | 1048 | 872 | 312 | 683 | 332 | 212 |
| NA | 0.280 | 0.063 | 0.575 | 0.126 | 0.033 | 0.103 | 0.045 | 0.056 | 0.3 | 1420 | 1065 | 466 | 946 | 483 | 323 |
| NA | 0.322 | 0.092 | 0.579 | 0.211 | 0.083 | 0.184 | 0.083 | 0.100 | 0.2 | 945 | 764 | 241 | 685 | 292 | 190 |
| NA | 0.250 | 0.058 | 0.558 | 0.121 | 0.045 | 0.105 | 0.029 | 0.055 | 0.3 | 1380 | 1013 | 528 | 904 | 391 | 326 |
| NA | 0.277 | 0.060 | 0.545 | 0.144 | 0.042 | 0.116 | 0.032 | 0.073 | 0.3 | 1010 | 947 | 336 | 699 | 357 | 239 |
| NA | 0.279 | 0.085 | 0.567 | 0.188 | 0.065 | 0.139 | 0.073 | 0.110 | 0.2 | 747 | 626 | 216 | 600 | 276 | 165 |
| NA | 0.310 | 0.107 | 0.550 | 0.221 | 0.088 | 0.165 | 0.145 | 0.117 | 0.2 | 767 | 593 | 181 | 579 | 258 | 149 |
| NA | 0.285 | 0.082 | 0.541 | 0.169 | 0.070 | 0.160 | 0.031 | 0.095 | 0.2 | 919 | 770 | 270 | 681 | 292 | 205 |
| NA | 0.244 | 0.064 | 0.454 | 0.139 | 0.043 | 0.104 | 0.041 | 0.056 | 0.3 | 855 | 793 | 286 | 587 | 304 | 173 |
| NA | 0.276 | 0.082 | 0.568 | 0.174 | 0.056 | 0.132 | 0.031 | 0.092 | 0.3 | 995 | 745 | 319 | 649 | 347 | 221 |
| NA | 0.301 | 0.080 | 0.609 | 0.187 | 0.064 | 0.141 | 0.022 | 0.089 | 0.2 | 1398 | 1014 | 392 | 897 | 479 | 343 |
| NA | 0.259 | 0.095 | 0.531 | 0.181 | 0.070 | 0.129 | 0.049 | 0.123 | 0.2 | 894 | 608 | 247 | 716 | 296 | 189 |
| NA | 0.296 | 0.092 | 0.500 | 0.173 | 0.053 | 0.134 | 0.066 | 0.114 | 0.2 | 947 | 709 | 264 | 677 | 302 | 189 |
| NA | 0.303 | 0.111 | 0.531 | 0.208 | 0.078 | 0.175 | 0.070 | 0.144 | 0.2 | 825 | 540 | 198 | 552 | 241 | 147 |
| NA | 0.327 | 0.135 | 0.503 | 0.256 | 0.106 | 0.198 | 0.075 | 0.176 | 0.2 | 694 | 430 | 146 | 449 | 186 | 117 |
| NA | 0.273 | 0.063 | 0.554 | 0.166 | 0.051 | 0.117 | 0.022 | 0.069 | 0.3 | 1318 | 1193 | 407 | 927 | 496 | 342 |
| NA | 0.309 | 0.114 | 0.545 | 0.227 | 0.081 | 0.174 | 0.077 | 0.145 | 0.2 | 745 | 530 | 178 | 598 | 239 | 140 |
| NA | 0.391 | 0.066 | 0.424 | 0.268 | 0.093 | 0.203 | 0.035 | 0.101 | 0.2 | 792 | 1048 | 199 | 569 | 258 | 174 |
| NA | 0.351 | 0.095 | 0.598 | 0.246 | 0.083 | 0.164 | 0.232 | 0.114 | 0.2 | 1308 | 955 | 293 | 837 | 463 | 297 |
| NA | 0.311 | 0.093 | 0.568 | 0.209 | 0.076 | 0.160 | 0.051 | 0.126 | 0.2 | 1043 | 732 | 274 | 707 | 304 | 206 |
| NA | 0.326 | 0.082 | 0.594 | 0.232 | 0.086 | 0.166 | 0.067 | 0.105 | 0.2 | 1304 | 1241 | 332 | 880 | 434 | 307 |
| NA | 0.303 | 0.119 | 0.603 | 0.244 | 0.085 | 0.181 | 0.077 | 0.168 | 0.2 | 1005 | 654 | 246 | 781 | 341 | 202 |
| NA | 0.284 | 0.093 | 0.569 | 0.193 | 0.067 | 0.135 | 0.082 | 0.102 | 0.2 | 1147 | 693 | 248 | 680 | 300 | 184 |
| NA | 0.285 | 0.073 | 0.505 | 0.160 | 0.052 | 0.107 | 0.030 | 0.092 | 0.3 | 884 | 672 | 259 | 576 | 286 | 181 |
| NA | 0.299 | 0.065 | 0.623 | 0.135 | 0.047 | 0.118 | 0.051 | 0.079 | 0.3 | 1551 | 991 | 513 | 988 | 457 | 373 |
| NA | 0.324 | 0.106 | 0.460 | 0.206 | 0.078 | 0.145 | 0.050 | 0.118 | 0.2 | 639 | 391 | 174 | 409 | 169 | 115 |
| NA | 0.281 | 0.060 | 0.413 | 0.139 | 0.041 | 0.092 | 0.080 | 0.064 | 0.3 | 764 | 745 | 254 | 496 | 250 | 158 |
| NA | 0.277 | 0.099 | 0.513 | 0.151 | 0.061 | 0.129 | 0.051 | 0.104 | 0.3 | 660 | 356 | 200 | 438 | 177 | 131 |
| NA | 0.319 | 0.073 | 0.420 | 0.192 | 0.067 | 0.134 | 0.082 | 0.082 | 0.3 | 770 | 709 | 235 | 563 | 263 | 180 |
| NA | 0.293 | 0.106 | 0.473 | 0.196 | 0.075 | 0.148 | 0.056 | 0.113 | 0.2 | 753 | 428 | 171 | 443 | 204 | 124 |
| NA | 0.343 | 0.088 | 0.535 | 0.215 | 0.089 | 0.169 | 0.050 | 0.090 | 0.2 | 791 | 655 | 213 | 540 | 212 | 156 |
| NA | 0.264 | 0.062 | 0.537 | 0.158 | 0.055 | 0.118 | 0.032 | 0.072 | 0.3 | 925 | 963 | 312 | 706 | 314 | 211 |
| NA | 0.269 | 0.085 | 0.460 | 0.141 | 0.048 | 0.112 | 0.061 | 0.082 | 0.3 | 704 | 386 | 228 | 395 | 183 | 132 |
| NA | 0.265 | 0.107 | 0.405 | 0.180 | 0.065 | 0.126 | 0.131 | 0.118 | 0.2 | 856 | 401 | 226 | 484 | 219 | 148 |
| NA | 0.290 | 0.072 | 0.520 | 0.146 | 0.051 | 0.110 | 0.049 | 0.067 | 0.3 | 890 | 675 | 274 | 608 | 280 | 186 |
| NA | 0.309 | 0.136 | 0.443 | 0.250 | 0.084 | 0.161 | 0.161 | 0.174 | 0.2 | 1020 | 451 | 180 | 479 | 212 | 126 |
| NA | 0.146 | 0.042 | 0.303 | 0.066 | 0.021 | 0.049 | 0.045 | 0.040 | 0.4 | 1783 | 1083 | 818 | 959 | 550 | 369 |
| NA | 0.329 | 0.078 | 0.594 | 0.202 | 0.066 | 0.152 | 0.063 | 0.089 | 0.2 | 1378 | 1295 | 361 | 951 | 465 | 301 |
| NA | 0.263 | 0.103 | 0.446 | 0.178 | 0.052 | 0.127 | 0.186 | 0.142 | 0.2 | 754 | 365 | 184 | 413 | 189 | 115 |
| NA | 0.134 | 0.085 | 0.121 | 0.129 | 0.039 | 0.072 | 0.102 | 0.071 | 0.3 | 1811 | 910 | 515 | 1138 | 615 | 396 |
| NA | 0.284 | 0.104 | 0.493 | 0.199 | 0.063 | 0.125 | 0.135 | 0.103 | 0.2 | 700 | 405 | 193 | 447 | 179 | 127 |
| NA | 0.333 | 0.085 | 0.628 | 0.187 | 0.066 | 0.148 | 0.068 | 0.090 | 0.2 | 1040 | 698 | 283 | 654 | 301 | 207 |
| NA | 0.309 | 0.107 | 0.505 | 0.228 | 0.090 | 0.179 | 0.142 | 0.146 | 0.2 | 1188 | 831 | 274 | 787 | 311 | 210 |
| NA | 0.317 | 0.116 | 0.523 | 0.222 | 0.084 | 0.158 | 0.150 | 0.140 | 0.2 | 711 | 410 | 189 | 410 | 169 | 118 |
| NA | 0.327 | 0.105 | 0.522 | 0.208 | 0.078 | 0.168 | 0.054 | 0.124 | 0.2 | 690 | 450 | 179 | 395 | 168 | 112 |

| | | | | | | | | | | | | | | | |
|---|---|---|---|---|---|---|---|---|---|---|---|---|---|---|---|
| NA | 0.309 | 0.077 | 0.533 | 0.182 | 0.061 | 0.140 | 0.027 | 0.084 | 0.2 | 946 | 769 | 263 | 696 | 322 | 202 |
| NA | 0.353 | 0.129 | 0.578 | 0.282 | 0.112 | 0.233 | 0.114 | 0.174 | 0.2 | 725 | 543 | 163 | 550 | 219 | 149 |
| NA | 0.314 | 0.122 | 0.600 | 0.222 | 0.083 | 0.169 | 0.129 | 0.139 | 0.2 | 800 | 513 | 188 | 609 | 280 | 166 |
| NA | 0.318 | 0.128 | 0.504 | 0.214 | 0.076 | 0.182 | 0.060 | 0.131 | 0.2 | 638 | 332 | 142 | 359 | 147 | 92 |
| NA | 0.257 | 0.071 | 0.417 | 0.132 | 0.042 | 0.086 | 0.034 | 0.063 | 0.3 | 913 | 600 | 302 | 513 | 232 | 162 |
| NA | 0.278 | 0.057 | 0.523 | 0.135 | 0.041 | 0.119 | 0.025 | 0.047 | 0.3 | 1083 | 1064 | 352 | 739 | 357 | 234 |
| NA | 0.333 | 0.085 | 0.546 | 0.245 | 0.083 | 0.179 | 0.033 | 0.114 | 0.2 | 898 | 904 | 243 | 640 | 282 | 177 |
| NA | 0.171 | 0.057 | 0.360 | 0.090 | 0.029 | 0.070 | 0.129 | 0.047 | 0.3 | 1213 | 734 | 401 | 746 | 335 | 212 |
| NA | 0.307 | 0.077 | 0.555 | 0.189 | 0.064 | 0.125 | 0.034 | 0.099 | 0.2 | 1072 | 768 | 293 | 726 | 350 | 241 |
| NA | 0.283 | 0.109 | 0.449 | 0.179 | 0.060 | 0.131 | 0.152 | 0.101 | 0.2 | 775 | 440 | 195 | 510 | 207 | 141 |
| NA | 0.346 | 0.126 | 0.473 | 0.223 | 0.096 | 0.172 | 0.066 | 0.160 | 0.2 | 694 | 354 | 150 | 351 | 150 | 102 |
| NA | 0.287 | 0.086 | 0.507 | 0.154 | 0.054 | 0.126 | 0.041 | 0.096 | 0.3 | 822 | 549 | 247 | 522 | 247 | 158 |
| NA | 0.263 | 0.058 | 0.563 | 0.156 | 0.059 | 0.133 | 0.020 | 0.069 | 0.3 | 1503 | 1555 | 561 | 1232 | 545 | 387 |
| NA | 0.308 | 0.100 | 0.519 | 0.208 | 0.075 | 0.175 | 0.063 | 0.124 | 0.2 | 576 | 387 | 159 | 371 | 154 | 103 |
| NA | 0.376 | 0.134 | 0.608 | 0.321 | 0.112 | 0.242 | 0.051 | 0.195 | 0.2 | 581 | 378 | 125 | 385 | 146 | 103 |
| NA | 0.337 | 0.096 | 0.536 | 0.250 | 0.086 | 0.200 | 0.041 | 0.123 | 0.2 | 730 | 719 | 174 | 561 | 243 | 154 |
| NA | 0.401 | 0.151 | 0.480 | 0.318 | 0.120 | 0.268 | 0.042 | 0.255 | 0.2 | 635 | 349 | 126 | 406 | 167 | 104 |
| NA | 0.348 | 0.066 | 0.441 | 0.235 | 0.079 | 0.180 | 0.041 | 0.084 | 0.3 | 697 | 864 | 187 | 492 | 251 | 164 |
| NA | 0.345 | 0.069 | 0.546 | 0.202 | 0.071 | 0.167 | 0.090 | 0.079 | 0.2 | 1003 | 1057 | 267 | 763 | 330 | 229 |
| NA | 0.274 | 0.064 | 0.457 | 0.130 | 0.039 | 0.117 | 0.054 | 0.069 | 0.3 | 850 | 655 | 296 | 597 | 260 | 175 |
| NA | 0.347 | 0.129 | 0.563 | 0.271 | 0.109 | 0.194 | 0.088 | 0.163 | 0.2 | 1275 | 1006 | 252 | 924 | 392 | 244 |
| NA | 0.328 | 0.089 | 0.581 | 0.237 | 0.086 | 0.192 | 0.033 | 0.110 | 0.2 | 863 | 816 | 218 | 673 | 313 | 203 |
| NA | 0.332 | 0.080 | 0.551 | 0.210 | 0.074 | 0.169 | 0.026 | 0.099 | 0.2 | 1098 | 957 | 289 | 773 | 398 | 261 |
| NA | 0.267 | 0.108 | 0.428 | 0.166 | 0.056 | 0.120 | 0.175 | 0.102 | 0.2 | 1128 | 636 | 270 | 779 | 394 | 213 |
| NA | 0.358 | 0.131 | 0.567 | 0.277 | 0.099 | 0.216 | 0.095 | 0.175 | 0.2 | 854 | 508 | 189 | 567 | 249 | 166 |
| NA | 0.401 | 0.073 | 0.481 | 0.239 | 0.086 | 0.177 | 0.034 | 0.091 | 0.2 | 579 | 630 | 138 | 404 | 181 | 115 |
| NA | 0.324 | 0.129 | 0.463 | 0.204 | 0.072 | 0.176 | 0.052 | 0.165 | 0.2 | 988 | 437 | 210 | 500 | 196 | 132 |
| NA | 0.195 | 0.107 | 0.277 | 0.155 | 0.038 | 0.076 | 0.197 | 0.064 | 0.2 | 1188 | 516 | 271 | 654 | 345 | 173 |
| NA | 0.354 | 0.108 | 0.598 | 0.250 | 0.100 | 0.205 | 0.040 | 0.131 | 0.2 | 812 | 595 | 175 | 577 | 238 | 155 |
| NA | 0.414 | 0.133 | 0.643 | 0.364 | 0.156 | 0.301 | 0.106 | 0.221 | 0.2 | 474 | 400 | 94 | 311 | 133 | 87 |
| NA | 0.362 | 0.075 | 0.439 | 0.253 | 0.095 | 0.188 | 0.038 | 0.091 | 0.2 | 670 | 765 | 172 | 467 | 193 | 129 |
| NA | 0.312 | 0.070 | 0.538 | 0.182 | 0.053 | 0.126 | 0.034 | 0.077 | 0.2 | 1066 | 1000 | 305 | 747 | 317 | 208 |
| NA | 0.320 | 0.071 | 0.539 | 0.210 | 0.070 | 0.159 | 0.047 | 0.076 | 0.2 | 1214 | 1092 | 336 | 805 | 422 | 291 |
| NA | 0.351 | 0.099 | 0.569 | 0.249 | 0.095 | 0.186 | 0.039 | 0.118 | 0.2 | 905 | 799 | 209 | 651 | 290 | 183 |
| NA | 0.357 | 0.114 | 0.612 | 0.251 | 0.086 | 0.191 | 0.038 | 0.140 | 0.2 | 912 | 673 | 198 | 644 | 297 | 177 |
| NA | 0.255 | 0.070 | 0.552 | 0.154 | 0.048 | 0.116 | 0.027 | 0.079 | 0.3 | 1068 | 817 | 383 | 774 | 384 | 263 |
| NA | 0.289 | 0.103 | 0.517 | 0.187 | 0.072 | 0.156 | 0.096 | 0.107 | 0.2 | 615 | 466 | 161 | 474 | 208 | 126 |
| NA | 0.191 | 0.089 | 0.270 | 0.147 | 0.043 | 0.094 | 0.079 | 0.078 | 0.3 | 983 | 565 | 316 | 700 | 329 | 200 |
| NA | 0.352 | 0.108 | 0.562 | 0.262 | 0.105 | 0.210 | 0.041 | 0.137 | 0.2 | 821 | 636 | 178 | 576 | 254 | 171 |
| NA | 0.339 | 0.078 | 0.538 | 0.225 | 0.077 | 0.173 | 0.043 | 0.113 | 0.2 | 927 | 933 | 229 | 719 | 343 | 216 |
| NA | 0.322 | 0.117 | 0.460 | 0.237 | 0.083 | 0.170 | 0.114 | 0.150 | 0.2 | 845 | 422 | 196 | 476 | 194 | 124 |
| NA | 0.356 | 0.082 | 0.494 | 0.231 | 0.084 | 0.195 | 0.094 | 0.120 | 0.2 | 945 | 761 | 246 | 626 | 266 | 193 |
| NA | 0.354 | 0.123 | 0.570 | 0.272 | 0.100 | 0.217 | 0.043 | 0.160 | 0.2 | 798 | 573 | 170 | 539 | 243 | 149 |
| NA | 0.300 | 0.086 | 0.541 | 0.189 | 0.062 | 0.150 | 0.051 | 0.108 | 0.3 | 777 | 523 | 240 | 540 | 213 | 154 |
| NA | 0.343 | 0.126 | 0.567 | 0.279 | 0.104 | 0.217 | 0.040 | 0.172 | 0.2 | 696 | 450 | 148 | 515 | 208 | 135 |
| NA | 0.309 | 0.068 | 0.604 | 0.151 | 0.055 | 0.126 | 0.031 | 0.074 | 0.2 | 1186 | 874 | 351 | 738 | 324 | 243 |
| NA | 0.317 | 0.106 | 0.560 | 0.207 | 0.077 | 0.175 | 0.075 | 0.138 | 0.2 | 998 | 578 | 241 | 673 | 262 | 179 |
| NA | 0.362 | 0.098 | 0.626 | 0.243 | 0.094 | 0.200 | 0.031 | 0.116 | 0.2 | 1068 | 671 | 250 | 676 | 312 | 233 |
| NA | 0.366 | 0.132 | 0.601 | 0.260 | 0.109 | 0.219 | 0.029 | 0.157 | 0.2 | 742 | 483 | 150 | 514 | 238 | 147 |
| NA | 0.275 | 0.076 | 0.404 | 0.144 | 0.049 | 0.116 | 0.057 | 0.078 | 0.3 | 878 | 486 | 319 | 480 | 213 | 172 |
| NA | 0.324 | 0.095 | 0.537 | 0.222 | 0.081 | 0.172 | 0.069 | 0.130 | 0.2 | 856 | 667 | 220 | 562 | 212 | 163 |
| NA | 0.257 | 0.076 | 0.544 | 0.164 | 0.043 | 0.108 | 0.058 | 0.071 | 0.3 | 1504 | 1060 | 462 | 903 | 522 | 317 |
| NA | 0.338 | 0.066 | 0.548 | 0.236 | 0.076 | 0.177 | 0.042 | 0.090 | 0.2 | 922 | 1003 | 239 | 659 | 306 | 205 |
| NA | 0.358 | 0.054 | 0.440 | 0.236 | 0.086 | 0.195 | 0.042 | 0.064 | 0.2 | 961 | 1446 | 244 | 685 | 325 | 228 |
| NA | 0.334 | 0.107 | 0.507 | 0.237 | 0.083 | 0.190 | 0.054 | 0.132 | 0.2 | 752 | 507 | 182 | 466 | 208 | 131 |
| NA | 0.247 | 0.082 | 0.449 | 0.136 | 0.042 | 0.110 | 0.048 | 0.055 | 0.3 | 775 | 417 | 240 | 440 | 173 | 125 |

| | | | | | | | | | | | | | | | |
|---|---|---|---|---|---|---|---|---|---|---|---|---|---|---|---|
| NA | 0.296 | 0.107 | 0.588 | 0.189 | 0.084 | 0.161 | 0.061 | 0.112 | 0.3 | 453 | 329 | 139 | 394 | 154 | 106 |
| NA | 0.299 | 0.108 | 0.552 | 0.198 | 0.076 | 0.157 | 0.068 | 0.115 | 0.2 | 728 | 437 | 191 | 448 | 191 | 131 |
| NA | 0.353 | 0.102 | 0.572 | 0.221 | 0.068 | 0.174 | 0.043 | 0.171 | 0.2 | 893 | 582 | 231 | 642 | 286 | 194 |
| NA | 0.276 | 0.092 | 0.397 | 0.171 | 0.057 | 0.125 | 0.022 | 0.105 | 0.3 | 727 | 493 | 205 | 536 | 223 | 145 |
| NA | 0.317 | 0.106 | 0.574 | 0.212 | 0.081 | 0.165 | 0.039 | 0.115 | 0.2 | 1077 | 792 | 254 | 765 | 351 | 226 |
| NA | 0.388 | 0.146 | 0.580 | 0.308 | 0.125 | 0.234 | 0.033 | 0.206 | 0.2 | 682 | 445 | 136 | 506 | 197 | 128 |
| NA | 0.273 | 0.106 | 0.446 | 0.178 | 0.064 | 0.141 | 0.057 | 0.116 | 0.2 | 698 | 338 | 188 | 375 | 150 | 108 |
| NA | 0.269 | 0.092 | 0.429 | 0.165 | 0.049 | 0.126 | 0.099 | 0.101 | 0.2 | 926 | 533 | 229 | 529 | 255 | 155 |
| NA | 0.343 | 0.085 | 0.548 | 0.218 | 0.080 | 0.174 | 0.054 | 0.115 | 0.2 | 740 | 644 | 194 | 542 | 214 | 142 |
| NA | 0.324 | 0.066 | 0.572 | 0.180 | 0.054 | 0.147 | 0.028 | 0.076 | 0.3 | 1181 | 982 | 357 | 812 | 333 | 241 |
| NA | 0.308 | 0.069 | 0.465 | 0.170 | 0.053 | 0.127 | 0.055 | 0.082 | 0.3 | 740 | 654 | 236 | 478 | 188 | 137 |
| NA | 0.360 | 0.118 | 0.591 | 0.289 | 0.102 | 0.221 | 0.058 | 0.177 | 0.2 | 800 | 672 | 177 | 600 | 243 | 162 |
| NA | 0.311 | 0.071 | 0.499 | 0.176 | 0.063 | 0.146 | 0.028 | 0.096 | 0.2 | 744 | 678 | 210 | 498 | 216 | 142 |
| NA | 0.311 | 0.121 | 0.457 | 0.236 | 0.094 | 0.178 | 0.087 | 0.130 | 0.2 | 895 | 690 | 204 | 616 | 301 | 182 |
| NA | 0.344 | 0.099 | 0.571 | 0.259 | 0.099 | 0.186 | 0.028 | 0.105 | 0.2 | 954 | 847 | 206 | 699 | 324 | 205 |
| NA | 0.361 | 0.102 | 0.591 | 0.264 | 0.096 | 0.213 | 0.070 | 0.129 | 0.2 | 796 | 653 | 171 | 568 | 242 | 150 |
| NA | 0.279 | 0.076 | 0.516 | 0.166 | 0.058 | 0.128 | 0.073 | 0.098 | 0.3 | 772 | 587 | 233 | 533 | 205 | 141 |
| NA | 0.304 | 0.069 | 0.583 | 0.172 | 0.056 | 0.130 | 0.027 | 0.090 | 0.2 | 1067 | 868 | 321 | 737 | 342 | 250 |
| NA | 0.325 | 0.066 | 0.562 | 0.198 | 0.077 | 0.170 | 0.020 | 0.081 | 0.2 | 630 | 666 | 175 | 482 | 191 | 137 |
| NA | 0.278 | 0.074 | 0.540 | 0.164 | 0.058 | 0.128 | 0.079 | 0.078 | 0.2 | 956 | 694 | 285 | 598 | 260 | 171 |
| NA | 0.306 | 0.063 | 0.567 | 0.164 | 0.047 | 0.138 | 0.031 | 0.069 | 0.2 | 819 | 798 | 245 | 558 | 275 | 181 |
| NA | 0.342 | 0.063 | 0.497 | 0.204 | 0.075 | 0.172 | 0.029 | 0.072 | 0.3 | 694 | 753 | 199 | 452 | 214 | 140 |
| NA | 0.332 | 0.094 | 0.563 | 0.242 | 0.085 | 0.187 | 0.080 | 0.141 | 0.2 | 900 | 775 | 216 | 667 | 293 | 184 |
| NA | 0.328 | 0.068 | 0.570 | 0.198 | 0.063 | 0.144 | 0.029 | 0.086 | 0.2 | 1294 | 1052 | 349 | 810 | 440 | 306 |
| NA | 0.363 | 0.122 | 0.592 | 0.263 | 0.100 | 0.204 | 0.041 | 0.185 | 0.2 | 661 | 451 | 147 | 458 | 188 | 118 |
| NA | 0.363 | 0.109 | 0.610 | 0.263 | 0.104 | 0.211 | 0.031 | 0.142 | 0.2 | 1002 | 820 | 220 | 680 | 298 | 198 |
| NA | 0.255 | 0.059 | 0.527 | 0.139 | 0.048 | 0.116 | 0.023 | 0.055 | 0.3 | 1389 | 1196 | 436 | 1005 | 539 | 343 |
| NA | 0.180 | 0.040 | 0.438 | 0.065 | 0.023 | 0.056 | 0.026 | 0.057 | 0.4 | 2238 | 1371 | 1145 | 1205 | 603 | 511 |
| NA | 0.330 | 0.111 | 0.578 | 0.243 | 0.090 | 0.179 | 0.039 | 0.155 | 0.2 | 943 | 605 | 225 | 636 | 276 | 180 |
| NA | 0.297 | 0.071 | 0.583 | 0.169 | 0.050 | 0.124 | 0.023 | 0.084 | 0.2 | 1209 | 977 | 357 | 822 | 404 | 276 |
| NA | 0.267 | 0.095 | 0.435 | 0.155 | 0.049 | 0.118 | 0.074 | 0.104 | 0.2 | 1040 | 558 | 286 | 607 | 289 | 179 |
| NA | 0.281 | 0.117 | 0.407 | 0.198 | 0.077 | 0.157 | 0.148 | 0.184 | 0.2 | 745 | 428 | 177 | 474 | 227 | 144 |
| NA | 0.345 | 0.077 | 0.519 | 0.195 | 0.065 | 0.142 | 0.022 | 0.094 | 0.3 | 1290 | 1005 | 382 | 823 | 404 | 290 |
| NA | 0.319 | 0.069 | 0.563 | 0.204 | 0.065 | 0.143 | 0.039 | 0.089 | 0.2 | 1228 | 1000 | 331 | 792 | 405 | 285 |
| NA | 0.330 | 0.091 | 0.542 | 0.204 | 0.084 | 0.165 | 0.039 | 0.128 | 0.2 | 752 | 582 | 193 | 525 | 201 | 141 |
| NA | 0.310 | 0.060 | 0.554 | 0.156 | 0.049 | 0.137 | 0.032 | 0.060 | 0.3 | 853 | 850 | 254 | 585 | 272 | 188 |
| NA | 0.301 | 0.114 | 0.494 | 0.199 | 0.100 | 0.065 | 12.951 | 0.850 | 0.2 | 713 | 367 | 165 | 395 | 168 | 2222 |
| NA | 0.360 | 0.062 | 0.507 | 0.221 | 0.078 | 0.190 | 0.054 | 0.080 | 0.2 | 835 | 1074 | 196 | 619 | 289 | 185 |
| NA | 0.299 | 0.067 | 0.553 | 0.165 | 0.057 | 0.142 | 0.037 | 0.076 | 0.2 | 951 | 812 | 283 | 664 | 306 | 215 |
| NA | 0.337 | 0.076 | 0.547 | 0.222 | 0.086 | 0.181 | 0.035 | 0.106 | 0.2 | 803 | 751 | 200 | 532 | 217 | 155 |
| NA | 0.306 | 0.121 | 0.476 | 0.206 | 0.071 | 0.158 | 0.083 | 0.149 | 0.2 | 809 | 376 | 183 | 422 | 178 | 117 |
| NA | 0.317 | 0.084 | 0.547 | 0.194 | 0.064 | 0.151 | 0.035 | 0.106 | 0.2 | 722 | 528 | 210 | 544 | 237 | 153 |
| NA | 0.220 | 0.049 | 0.444 | 0.079 | 0.022 | 0.066 | 0.028 | 0.046 | 0.4 | 1061 | 652 | 476 | 603 | 290 | 228 |
| NA | 0.364 | 0.099 | 0.474 | 0.248 | 0.092 | 0.190 | 0.053 | 0.165 | 0.2 | 603 | 422 | 136 | 358 | 140 | 92 |
| NA | 0.302 | 0.066 | 0.593 | 0.151 | 0.041 | 0.114 | 0.071 | 0.055 | 0.2 | 1088 | 976 | 320 | 726 | 331 | 212 |
| NA | 0.209 | 0.087 | 0.276 | 0.131 | 0.034 | 0.100 | 0.077 | 0.139 | 0.2 | 1212 | 622 | 319 | 764 | 482 | 246 |
| NA | 0.333 | 0.132 | 0.424 | 0.217 | 0.090 | 0.186 | 0.042 | 0.190 | 0.2 | 691 | 326 | 141 | 329 | 133 | 84 |
| NA | 0.307 | 0.079 | 0.496 | 0.167 | 0.061 | 0.137 | 0.042 | 0.092 | 0.3 | 583 | 446 | 185 | 383 | 158 | 115 |
| NA | 0.245 | 0.049 | 0.581 | 0.082 | 0.029 | 0.078 | 0.048 | 0.050 | 0.3 | 1461 | 912 | 608 | 866 | 414 | 307 |
| NA | 0.192 | 0.039 | 0.410 | 0.072 | 0.024 | 0.056 | 0.242 | 0.033 | 0.4 | 1778 | 1279 | 870 | 1190 | 551 | 426 |
| NA | 0.255 | 0.097 | 0.358 | 0.184 | 0.057 | 0.146 | 0.108 | 0.093 | 0.2 | 870 | 506 | 194 | 530 | 243 | 147 |
| NA | 0.293 | 0.101 | 0.539 | 0.184 | 0.068 | 0.148 | 0.102 | 0.109 | 0.2 | 641 | 385 | 166 | 407 | 161 | 107 |
| NA | 0.293 | 0.089 | 0.526 | 0.186 | 0.071 | 0.130 | 0.126 | 0.112 | 0.3 | 718 | 495 | 219 | 443 | 193 | 155 |
| NA | 0.326 | 0.087 | 0.599 | 0.195 | 0.068 | 0.159 | 0.025 | 0.091 | 0.2 | 1084 | 837 | 271 | 728 | 344 | 209 |
| NA | 0.278 | 0.079 | 0.522 | 0.136 | 0.053 | 0.115 | 0.112 | 0.088 | 0.3 | 848 | 530 | 262 | 515 | 222 | 161 |
| NA | 0.282 | 0.102 | 0.455 | 0.174 | 0.059 | 0.127 | 0.062 | 0.170 | 0.2 | 625 | 337 | 161 | 366 | 147 | 95 |

| | | | | | | | | | | | | | | | | |
|---|---|---|---|---|---|---|---|---|---|---|---|---|---|---|---|---|
| NA | 0.295 | 0.115 | 0.415 | 0.205 | 0.073 | 0.140 | 0.056 | 0.140 | 0.2 | 811 | 392 | 185 | 396 | 181 | 128 |
| NA | 0.356 | 0.083 | 0.593 | 0.241 | 0.090 | 0.206 | 0.033 | 0.117 | 0.2 | 912 | 864 | 231 | 633 | 274 | 181 |
| NA | 0.320 | 0.114 | 0.531 | 0.246 | 0.089 | 0.172 | 0.060 | 0.128 | 0.2 | 819 | 618 | 187 | 550 | 253 | 160 |
| NA | 0.242 | 0.115 | 0.418 | 0.167 | 0.055 | 0.118 | 0.096 | 0.086 | 0.2 | 879 | 546 | 234 | 545 | 276 | 155 |
| NA | 0.311 | 0.124 | 0.472 | 0.207 | 0.069 | 0.162 | 0.074 | 0.177 | 0.2 | 647 | 316 | 150 | 361 | 149 | 99 |
| NA | 0.326 | 0.071 | 0.521 | 0.195 | 0.064 | 0.152 | 0.042 | 0.084 | 0.2 | 1232 | 1087 | 321 | 856 | 441 | 311 |
| NA | 0.295 | 0.085 | 0.487 | 0.156 | 0.056 | 0.116 | 0.106 | 0.093 | 0.3 | 733 | 444 | 228 | 393 | 188 | 135 |
| NA | 0.321 | 0.082 | 0.487 | 0.193 | 0.060 | 0.156 | 0.093 | 0.115 | 0.2 | 724 | 561 | 201 | 470 | 236 | 158 |
| NA | 0.294 | 0.093 | 0.464 | 0.178 | 0.063 | 0.146 | 0.060 | 0.110 | 0.3 | 689 | 377 | 198 | 371 | 159 | 111 |
| NA | 0.288 | 0.079 | 0.481 | 0.156 | 0.060 | 0.130 | 0.137 | 0.097 | 0.3 | 769 | 543 | 233 | 510 | 220 | 160 |
| NA | 0.288 | 0.071 | 0.495 | 0.134 | 0.048 | 0.116 | 0.037 | 0.071 | 0.3 | 949 | 602 | 351 | 548 | 246 | 189 |
| NA | 0.210 | 0.047 | 0.107 | 0.114 | 0.030 | 0.070 | 0.047 | 0.050 | 0.8 | 1149 | 1128 | 857 | 691 | 345 | 277 |
| NA | 0.278 | 0.093 | 0.496 | 0.181 | 0.061 | 0.131 | 0.052 | 0.137 | 0.2 | 990 | 625 | 253 | 664 | 267 | 174 |
| NA | 0.319 | 0.107 | 0.523 | 0.214 | 0.081 | 0.159 | 0.098 | 0.117 | 0.2 | 615 | 426 | 157 | 385 | 159 | 114 |
| NA | 0.299 | 0.087 | 0.600 | 0.190 | 0.070 | 0.136 | 0.043 | 0.104 | 0.2 | 676 | 545 | 205 | 500 | 209 | 141 |
| NA | 0.230 | 0.052 | 0.452 | 0.103 | 0.035 | 0.081 | 0.044 | 0.059 | 0.3 | 1050 | 792 | 417 | 695 | 298 | 230 |
| NA | 0.348 | 0.078 | 0.573 | 0.247 | 0.101 | 0.210 | 0.030 | 0.105 | 0.2 | 952 | 1093 | 248 | 705 | 308 | 204 |
| NA | 0.273 | 0.109 | 0.448 | 0.179 | 0.065 | 0.124 | 0.163 | 0.134 | 0.2 | 829 | 391 | 200 | 463 | 183 | 122 |
| NA | 0.180 | 0.057 | 0.349 | 0.093 | 0.032 | 0.066 | 0.047 | 0.096 | 0.3 | 890 | 675 | 301 | 643 | 368 | 192 |
| NA | 0.273 | 0.124 | 0.422 | 0.207 | 0.084 | 0.139 | 0.097 | 0.196 | 0.3 | 548 | 384 | 153 | 413 | 215 | 132 |
| NA | 0.256 | 0.065 | 0.528 | 0.145 | 0.055 | 0.107 | 0.057 | 0.069 | 0.3 | 793 | 714 | 266 | 550 | 244 | 165 |

| [Ile] | [Leu] | [total] | L Asp | D Asp | L Glu | D Glu | L Ser | D Ser | Gly | L Ala | L hArg |
|---|---|---|---|---|---|---|---|---|---|---|---|
| 76 | 105 | 1859 | 2842.5 | 763.9 | 1541.75 | 194.05 | 620.3 | 230.75 | 1491.85 | 1592.9 | 1100.25 |
| 90 | 260 | 2599 | 663.7 | 121.6 | 774.4 | 30.2 | 263.1 | 88.4 | 691.1 | 476.5 | 249.1 |
| 94 | 154 | 2160 | 639.1 | 141.9 | 419.9 | 33.2 | 188.6 | 75.2 | 361.7 | 377.9 | 237.4 |
| 157 | 236 | 3131 | 1031.3 | 149.1 | 628.5 | 41.8 | 332.9 | 65.1 | 542.9 | 587.1 | 254.9 |
| 86 | 131 | 2249 | 694.6 | 168.7 | 377.2 | 37.3 | 180.9 | 69.9 | 372.2 | 455.4 | 231.5 |
| 169 | 283 | 3568 | 838.8 | 172.5 | 793.6 | 45.3 | 473.8 | 76.7 | 490.5 | 555.6 | 225.9 |
| 155 | 226 | 3134 | 990 | 161 | 603 | 43 | 310 | 74 | 449 | 600 | 248 |
| 179 | 291 | 4241 | 1221.5 | 171.3 | 854.2 | 38.5 | 481.8 | 148.6 | 685.5 | 952.5 | 241.3 |
| 86 | 142 | 2255 | 675.9 | 149 | 404.6 | 33.9 | 183.8 | 67.5 | 340.1 | 475.8 | 232.1 |
| 118 | 205 | 2705 | 681.8 | 151.3 | 510.3 | 39.8 | 194.2 | 82.4 | 386.7 | 532.6 | 221.5 |
| 147 | 179 | 2968 | 911.5 | 171 | 475.9 | 43.2 | 250.8 | 70.6 | 478.8 | 554.3 | 233.5 |
| 158 | 220 | 3057 | 827.9 | 152 | 493.3 | 47.7 | 258.1 | 56.8 | 508.7 | 589 | 226.1 |
| 101 | 146 | 2173 | 586.5 | 144.7 | 394.7 | 38.4 | 152.2 | 65.4 | 269.4 | 407.9 | 229.5 |
| 102 | 162 | 2498 | 700.7 | 161.7 | 463.7 | 42.8 | 187.1 | 74.4 | 345.6 | 438.3 | 222.8 |
| 89 | 149 | 2254 | 697.5 | 180.2 | 416.4 | 43.1 | 172.7 | 72.4 | 407.2 | 457.4 | 242.9 |
| 136 | 209 | 2956 | 765 | 148 | 537 | 39 | 240 | 83 | 390 | 589 | 224 |
| 150 | 234 | 3046 | 775 | 164 | 576 | 48 | 235 | 78 | 378 | 566 | 226 |
| 285 | 464 | 5489 | 1498 | 178 | 1041 | 53 | 519 | 115 | 750 | 948 | 226 |
| 154 | 263 | 3569 | 1117 | 192 | 837 | 46 | 394 | 129 | 688 | 817 | 268 |
| 158 | 226 | 3813 | 1084.1 | 197 | 586.6 | 44.9 | 328.6 | 99.6 | 542.3 | 572.9 | 201.2 |
| 77 | 134 | 1748 | 461.8 | 146.8 | 337.2 | 46.4 | 99.6 | 44.5 | 312.4 | 307.2 | 232.3 |
| 137 | 252 | 3486 | 963.9 | 118.5 | 767.8 | 28.9 | 418.5 | 106.5 | 627.3 | 770.9 | 242.7 |
| 173 | 255 | 4028 | 1171.8 | 166 | 672.9 | 32 | 496.8 | 155.2 | 647.5 | 942 | 240.7 |
| 390 | 593 | 7745 | 2986 | 309 | 1302 | 82 | 783 | 117 | 1100 | 1534 | 254 |
| 287 | 485 | 5439 | 1329 | 163 | 1154 | 60 | 480 | 99 | 755 | 1016 | 227 |
| 574 | 763 | 8760 | 16936 | 1733 | 11304 | 613 | 6547 | 803 | 10038 | 12780 | 1766 |
| 156 | 244 | 3000 | 749 | 142 | 585 | 54 | 234 | 60 | 452 | 757 | 248 |
| 865 | 1393 | 13445 | 2804 | 226 | 2010 | 90 | 1122 | 93 | 1676 | 2376 | 201 |
| 263 | 450 | 6081 | 1682 | 200 | 1283 | 43 | 765 | 192 | 994 | 1027 | 232 |
| 198 | 258 | 3731 | 928 | 196 | 640 | 67 | 255 | 67 | 510 | 733 | 224 |
| 194 | 277 | 3604 | 4063 | 816 | 2717 | 327 | 1049 | 296 | 2559 | 2913 | 982 |
| 355 | 511 | 7761 | 9768 | 1339 | 5408 | 289 | 3628 | 1012 | 4699 | 5008 | 927 |
| 186 | 257 | 4310 | 1438.8 | 221.9 | 709.5 | 60.6 | 350 | 92.5 | 603.1 | 825.9 | 231.6 |
| 165 | 349 | 4178 | 1123 | 133 | 977 | 32 | 548 | 108 | 839 | 731 | 235 |
| 129 | 209 | 2982 | 787 | 193 | 597 | 51 | 185 | 72 | 494 | 581 | 226 |
| 121 | 203 | 2868 | 833 | 155 | 610 | 38 | 262 | 89 | 512 | 526 | 234 |
| 95 | 182 | 2667 | 614 | 116 | 513 | 28 | 264 | 114 | 1015 | 517 | 242 |
| 141 | 214 | 3161 | 897 | 201 | 613.5 | 47.3 | 252.7 | 100.4 | 515.8 | 575.2 | 232.7 |
| 117 | 189 | 2628 | 686.2 | 163.8 | 534.5 | 45 | 177.6 | 82.4 | 438.8 | 567.4 | 238.4 |
| 136 | 239 | 3307 | 906 | 206 | 744 | 58 | 245 | 107 | 571 | 754 | 249 |
| 226 | 337 | 4408 | 1211.1 | 200.5 | 805 | 47.8 | 361.9 | 95.9 | 653.6 | 811.2 | 233.4 |
| 120 | 175 | 2441 | 655 | 167 | 477 | 54 | 150 | 60 | 341 | 477 | 238 |
| 365 | 523 | 7229 | 1736 | 350 | 1133 | 122 | 439 | 126 | 861 | 1138 | 201 |
| 332 | 563 | 7373 | 1869 | 221 | 1310 | 47 | 752 | 170 | 940 | 1167 | 203 |
| 174 | 245 | 3661 | 980.1 | 175.6 | 599.4 | 47.2 | 285.1 | 91.7 | 518.5 | 776.5 | 226.3 |
| 241 | 422 | 5796 | 1595.7 | 176.6 | 991.4 | 52.5 | 618 | 95.3 | 918 | 1244.5 | 233.3 |
| 126 | 214 | 2959 | 880 | 159 | 667 | 41 | 269 | 101 | 539 | 617 | 250 |
| 160 | 248 | 3109 | 866 | 159 | 565 | 51 | 250 | 69 | 505 | 635 | 243 |
| 134 | 205 | 3016 | 871 | 208 | 601 | 58 | 203 | 82 | 437 | 637 | 247 |
| 108 | 184 | 2545 | 692 | 129 | 528 | 36 | 208 | 72 | 389 | 486 | 231 |

| | | | | | | | | | | | |
|---|---|---|---|---|---|---|---|---|---|---|---|
| 114 | 174 | 2514 | 727.5 | 155.3 | 444.8 | 42 | 206.3 | 73.6 | 474.4 | 558.1 | 243.3 |
| 163 | 288 | 3854 | 1036 | 139 | 815 | 32 | 423 | 110 | 641 | 703 | 228 |
| 215 | 302 | 4005 | 991.3 | 166.9 | 646.3 | 62.3 | 306 | 65.2 | 594.2 | 757.4 | 218.5 |
| 189 | 251 | 3742 | 1136.1 | 174.5 | 630.3 | 43.3 | 295.9 | 84.3 | 558.1 | 826.5 | 242.3 |
| 136 | 189 | 2601 | 771 | 155 | 470 | 44 | 217 | 59 | 404 | 509 | 246 |
| 127 | 209 | 3127 | 893.4 | 158 | 622.1 | 37.3 | 298.7 | 114.4 | 424.6 | 702.6 | 240 |
| 120 | 262 | 3223 | 845.8 | 134.4 | 765 | 32.9 | 309.1 | 104.3 | 642 | 604.2 | 230.5 |
| 157 | 279 | 3984 | 1223 | 190 | 808 | 35 | 473 | 164 | 635 | 757 | 245 |
| 265 | 346 | 4594 | 1284 | 194.7 | 771.7 | 55.2 | 363.7 | 81.6 | 616.1 | 959.8 | 235.1 |
| 143 | 200 | 3272 | 1000.8 | 181.6 | 517.4 | 48.1 | 256.6 | 67.6 | 443.1 | 536.7 | 215.7 |
| 127 | 228 | 2626 | 2502 | 657 | 2335 | 200 | 750 | 371 | 2014 | 2247 | 995 |
| 171 | 302 | 3955 | 3917 | 1163 | 3495 | 332 | 900 | 489 | 2516 | 3401 | 995 |
| 146 | 237 | 3050 | 2980 | 774 | 2782 | 210 | 777 | 452 | 2089 | 2643 | 1005 |
| 224 | 379 | 4220 | 3754 | 1173 | 3817 | 380 | 866 | 518 | 2537 | 2970 | 939 |
| 154 | 240 | 3558 | 4436 | 1018 | 2679 | 368 | 1049 | 434 | 2786 | 2796 | 983 |
| 175 | 386 | 4119 | 977.1 | 283 | 978 | 56.9 | 264 | 153 | 818.9 | 701.4 | 235.1 |
| 167 | 361 | 3898 | 913.5 | 274.5 | 966.5 | 62.4 | 223.5 | 125.6 | 739.4 | 671.3 | 238.2 |
| 199 | 408 | 4603 | 1095.3 | 318.5 | 1027.4 | 63.8 | 279.1 | 158.6 | 803.4 | 870.5 | 237.7 |
| 126 | 223 | 2772 | 2555 | 767 | 2483 | 210 | 1015 | 408 | 2087 | 2384 | 1010 |
| 150 | 229 | 3209 | 3208 | 876 | 2653 | 229 | 909 | 509 | 1922 | 2560 | 980 |
| 173 | 278 | 3764 | 3641 | 934 | 3186 | 231 | 1125 | 563 | 2374 | 3092 | 973 |
| 96 | 207 | 2502 | 2375 | 648 | 2456 | 214 | 541 | 292 | 1660 | 1964 | 955 |
| 149 | 293 | 3180 | 2846 | 784 | 3086 | 225 | 790 | 453 | 2504 | 2342 | 959 |
| 174 | 469 | 3906 | 2998 | 1046 | 4682 | 347 | 846 | 422 | 3914 | 2450 | 977 |
| 153 | 278 | 3553 | 1006 | 173 | 822 | 47 | 331 | 123 | 675 | 748 | 249 |
| 145 | 280 | 3353 | 786.9 | 241.2 | 763 | 66.9 | 177.4 | 105.1 | 504.7 | 660 | 235.6 |
| 113 | 236 | 2488 | 517 | 177 | 621 | 69 | 102 | 55 | 376 | 420 | 232 |
| 102 | 174 | 2403 | 2451 | 681 | 1875 | 211 | 582 | 302 | 1576 | 1992 | 947 |
| 228 | 328 | 4676 | 1541.7 | 291.8 | 900.9 | 111.3 | 358.8 | 86.7 | 722.8 | 951 | 255.3 |
| 169 | 342 | 4097 | 1039.2 | 276.6 | 909.7 | 50.1 | 298.1 | 183.8 | 756.3 | 846.7 | 244.1 |
| 276 | 503 | 6817 | 1868.8 | 327.1 | 1302.4 | 45.7 | 882.4 | 392.4 | 1254.7 | 1055.3 | 233.3 |
| 108 | 194 | 2527 | 599.5 | 176.3 | 508.9 | 52.7 | 130.3 | 71.7 | 341.3 | 452.3 | 219.2 |
| 100 | 175 | 2187 | 473 | 132 | 500 | 47 | 117 | 61 | 392 | 450 | 234 |
| 97 | 144 | 2225 | 588 | 173 | 420 | 47 | 124 | 64 | 349 | 447 | 233 |
| 151 | 260 | 3336 | 830.5 | 225.9 | 724.6 | 52.9 | 212.5 | 129.8 | 553.4 | 605.6 | 235.8 |
| 103 | 171 | 2450 | 772 | 175 | 534 | 41 | 225 | 95 | 511 | 503 | 257 |
| 92 | 132 | 2131 | 648 | 182 | 420 | 44 | 162 | 76 | 420 | 416 | 246 |
| 98 | 157 | 2175 | 646 | 186 | 484 | 50 | 147 | 77 | 394 | 477 | 264 |
| 142 | 218 | 3075 | 840.8 | 195.8 | 594 | 46.6 | 236.2 | 103.5 | 475.3 | 606.5 | 235.8 |
| 135 | 214 | 3062 | 3235 | 965 | 2568 | 264 | 616 | 384 | 1815 | 2381 | 973 |
| 100 | 207 | 2452 | 591.8 | 186.3 | 599 | 56.5 | 132.9 | 74.1 | 514.5 | 477.4 | 243.7 |
| 338 | 721 | 7740 | 1328.7 | 386 | 1548.4 | 120.3 | 330.8 | 187 | 1039.8 | 1062.5 | 184.8 |
| 175 | 308 | 3831 | 995 | 309.8 | 846.5 | 62.4 | 231 | 144.8 | 696.2 | 652.4 | 241.6 |
| 142 | 207 | 2862 | 754.1 | 242.4 | 558.2 | 58 | 157.1 | 97.9 | 475.6 | 549.7 | 244.9 |
| 123 | 210 | 2876 | 684.5 | 194.4 | 584.3 | 53.7 | 299.7 | 86.6 | 530.7 | 606.8 | 238.9 |
| 144 | 290 | 3224 | 823 | 227 | 874 | 65 | 197 | 112 | 711 | 695 | 262 |
| 136 | 268 | 2872 | 2585 | 854 | 2686 | 288 | 608 | 312 | 2163 | 1945 | 961 |
| 153 | 303 | 3383 | 775.9 | 240.3 | 841.8 | 71.2 | 180 | 100.5 | 684.8 | 601.5 | 237.8 |
| 175 | 301 | 3820 | 848.6 | 236.4 | 779.7 | 61.4 | 217.3 | 123.1 | 547.7 | 672.4 | 219.8 |
| 71 | 118 | 1714 | 434 | 150.2 | 334.3 | 46.3 | 78.8 | 45.1 | 287.4 | 335.2 | 235.2 |
| 187 | 527 | 4734 | 1060.2 | 295 | 1445 | 75.8 | 292.3 | 163.4 | 1098.9 | 807.2 | 248.2 |
| 136 | 282 | 3037 | 677 | 204.1 | 741.9 | 62.6 | 151.6 | 85.4 | 617.8 | 592.1 | 238.1 |
| 227 | 511 | 4934 | 1085.3 | 337.3 | 1303.2 | 89.4 | 260.5 | 151.1 | 990.5 | 855.4 | 239.2 |
| 171 | 281 | 3752 | 918 | 285 | 794 | 71 | 198 | 115 | 501 | 679 | 232 |
| 107 | 220 | 2503 | 545 | 203 | 594 | 70 | 108 | 62 | 467 | 455 | 237 |
| 186 | 318 | 4099 | 1117 | 289 | 856 | 49 | 362 | 214 | 1225 | 823 | 251 |

| | | | | | | | | | | | |
|---|---|---|---|---|---|---|---|---|---|---|---|
| 196 | 363 | 4389 | 1033.2 | 314.8 | 877 | 60.1 | 257.7 | 150.9 | 714.8 | 694.9 | 220.3 |
| 98 | 164 | 2164 | 569.3 | 163.1 | 451.7 | 45.7 | 149.4 | 79.3 | 391.3 | 465.1 | 250.1 |
| 88 | 110 | 2000 | 580 | 170 | 326 | 34 | 139 | 77 | 293 | 444 | 244 |
| 164 | 246 | 3433 | 3149 | 923 | 2865 | 310 | 769 | 390 | 1764 | 2820 | 959 |
| 149 | 270 | 3264 | 765.2 | 247.5 | 762.2 | 75.3 | 145.2 | 79.7 | 527 | 603.3 | 240.5 |
| 111 | 256 | 2810 | 634 | 178 | 672 | 45 | 177 | 94 | 574 | 562 | 236 |
| 176 | 359 | 3886 | 1009 | 298.6 | 1054.9 | 67.9 | 260 | 149.1 | 766.7 | 798.5 | 270 |
| 115 | 215 | 2764 | 2635.75 | 729.55 | 2682.9 | 261.85 | 575.1 | 328.1 | 1929.6 | 2426.65 | 1006.4 |
| 121 | 243 | 2778 | 632 | 200 | 647 | 60 | 139 | 74 | 491 | 545 | 234 |
| 148 | 293 | 3256 | 714 | 252 | 802 | 72 | 148 | 81 | 585 | 487 | 226 |
| 99 | 202 | 2357 | 543.1 | 170.3 | 534.4 | 54.2 | 119.6 | 66.9 | 429.9 | 469.2 | 234 |
| 136 | 296 | 3202 | 749.4 | 228.2 | 810.8 | 64.3 | 183.2 | 105.1 | 671.8 | 549.9 | 238.1 |
| 187 | 327 | 4060 | 1044.4 | 274 | 867.2 | 53.7 | 313.2 | 167.7 | 670.2 | 766.1 | 242.8 |
| 217 | 314 | 4429 | 4721 | 1329 | 3607 | 271 | 1200 | 663 | 2634 | 2943 | 960 |
| 128 | 205 | 2713 | 288 | 84 | 273 | 25 | 67 | 37 | 223 | 240 | 109 |
| 119 | 225 | 2734 | 597 | 200 | 592 | 68 | 115 | 72 | 411 | 480 | 225 |
| 143 | 288 | 3451 | 833 | 247 | 775 | 59 | 203 | 117 | 612 | 674 | 236 |
| 167 | 317 | 3873 | 3764 | 971 | 3840 | 243 | 1051 | 562 | 2947 | 2902 | 985 |
| 119 | 252 | 2814 | 642 | 208 | 686 | 52 | 175 | 95 | 589 | 501 | 237 |
| 127 | 232 | 2768 | 629 | 195 | 620 | 59 | 140 | 80 | 508 | 527 | 233 |
| 139 | 284 | 3260 | 752 | 230 | 730 | 56 | 180 | 102 | 596 | 612 | 232 |
| 138 | 252 | 2986 | 700.7 | 208.4 | 668.2 | 55.2 | 168.4 | 91.3 | 560.5 | 588.3 | 239.8 |
| 107 | 223 | 2507 | 558 | 194 | 597 | 53 | 126 | 69 | 534 | 460 | 233 |
| 115 | 235 | 2892 | 697.8 | 211.8 | 613.2 | 57.7 | 158.8 | 90 | 498 | 591.5 | 235 |
| 89 | 171 | 2157 | 564.9 | 167 | 464.1 | 43.6 | 125.9 | 59.3 | 390.5 | 425.1 | 238 |
| 136 | 269 | 3122 | 722.7 | 216.4 | 748.3 | 57.6 | 178.9 | 98.3 | 570.4 | 622.5 | 240.3 |
| 141 | 338 | 3178 | 642 | 226 | 849 | 67 | 138 | 69 | 641 | 519 | 234 |
| 131 | 356 | 3210 | 638.8 | 220.1 | 895.2 | 63.4 | 134.8 | 68.2 | 697.5 | 536 | 234.9 |
| 162 | 294 | 3679 | 901 | 269 | 837 | 58 | 221 | 115 | 691 | 699 | 238 |
| 155 | 331 | 3576 | 812 | 237 | 852 | 48 | 214 | 115 | 700 | 658 | 233 |
| 123 | 250 | 2851 | 668.4 | 231.6 | 663.8 | 76.2 | 127.5 | 72.7 | 496 | 545.1 | 241.7 |
| 105 | 321 | 2760 | 507 | 219 | 834 | 60 | 132 | 55 | 682 | 395 | 227 |
| 110 | 294 | 2614 | 501 | 183.9 | 783.5 | 60 | 113.4 | 54.8 | 603.9 | 415.6 | 239 |
| 171 | 310 | 3543 | 3144 | 1007 | 3422 | 293 | 636 | 343 | 2567 | 2727 | 978 |
| 122 | 250 | 2720 | 597.9 | 201.7 | 697 | 74.4 | 109 | 60.7 | 496.3 | 529.7 | 245.2 |
| 148 | 323 | 3659 | 821.8 | 241.8 | 908.1 | 77.1 | 199.8 | 100.8 | 650.7 | 734.5 | 237.2 |
| 95 | 171 | 2319 | 572 | 182 | 477 | 55 | 118 | 65 | 436 | 457 | 233 |
| 117 | 231 | 2741 | 628 | 192 | 616 | 45 | 157 | 84 | 515 | 500 | 228 |
| 92 | 238 | 2154 | 455.8 | 183.8 | 657.8 | 58.1 | 100.7 | 48.7 | 551.7 | 379.8 | 258.6 |
| 155 | 340 | 3617 | 803.6 | 255.5 | 921.5 | 77.8 | 184.6 | 96.3 | 709.6 | 659.6 | 236.4 |
| 154 | 332 | 3695 | 880.2 | 234.7 | 899.9 | 58.5 | 217.9 | 125.1 | 713.4 | 682 | 234.5 |
| 243 | 451 | 5254 | 1265.4 | 317.1 | 1147.1 | 62.3 | 396.3 | 211.4 | 968.6 | 914.4 | 231.5 |
| 79 | 132 | 1602 | 352.1 | 140.9 | 340.7 | 40.6 | 80.3 | 43.3 | 299.4 | 303.7 | 241.5 |
| 138 | 228 | 3107 | 811.3 | 231.9 | 687.8 | 56.7 | 196.5 | 113.5 | 503.7 | 588.5 | 244.8 |
| 116 | 200 | 2684 | 667.1 | 208.8 | 551.6 | 58.1 | 138.4 | 79.6 | 450.2 | 537.6 | 235.3 |
| 132 | 263 | 2754 | 576.8 | 189.1 | 675.2 | 56.5 | 132.5 | 72.1 | 568.4 | 514.8 | 235.7 |
| 186 | | | 819 | 281 | 986 | 71 | 203 | 91 | 625 | 588 | 206 |
| 128 | 223 | 2657 | 602.1 | 215.3 | 577.4 | 64.1 | 129.5 | 76.8 | 502 | 473.9 | 235.8 |
| 79 | 137 | 1864 | 488.6 | 169 | 384.2 | 43.8 | 107.1 | 60.2 | 359.8 | 308.8 | 233.5 |
| 215 | 339 | 3993 | 954.3 | 237.6 | 827.4 | 56.2 | 258.9 | 141.8 | 639.9 | 867.2 | 242.5 |
| 249 | 501 | 4923 | 1066.9 | 332.8 | 1239.7 | 91.2 | 225.7 | 107.8 | 925.8 | 872.1 | 238.4 |
| 167 | 318 | 3394 | 714 | 232.9 | 806.7 | 76.8 | 148.9 | 81.9 | 611 | 656.2 | 234.3 |
| 67 | 109 | 1765 | 583.5 | 154.9 | 336.4 | 34.5 | 124.3 | 56.5 | 352.6 | 341.5 | 243.6 |
| 153 | 327 | 3534 | 785.9 | 263 | 853.5 | 69.1 | 169.7 | 92.1 | 684.3 | 594 | 232.4 |
| 167 | 322 | 3388 | 747.8 | 238.6 | 845.1 | 81.5 | 157.1 | 87.5 | 561.2 | 701 | 248.3 |
| 157 | 328 | 3538 | 742.2 | 248.7 | 785.2 | 59.1 | 166.2 | 87.6 | 573.7 | 643.7 | 223.2 |

| | | | | | | | | | | | |
|---|---|---|---|---|---|---|---|---|---|---|---|
| 219 | 437 | 4782 | 1178 | 332.4 | 1122.6 | 65.8 | 325.2 | 183.9 | 845.7 | 770.1 | 236.6 |
| 71 | 226 | 1984 | 373 | 162 | 622 | 46 | 87 | 36 | 566 | 309 | 234 |
| 120 | 236 | 2800 | 684 | 218 | 677 | 52 | 181 | 96 | 567 | 521 | 243 |
| 69 | 122 | 1599 | 390 | 150 | 351 | 44 | 82 | 45 | 276 | 305 | 242 |
| 74 | 161 | 1838 | 442 | 149 | 458 | 40 | 108 | 58 | 411 | 344 | 241 |
| 101 | 195 | 2248 | 497 | 210 | 513 | 54 | 105 | 52 | 462 | 387 | 231 |
| 139 | 341 | 3221 | 702 | 204 | 897 | 48 | 207 | 109 | 717 | 584 | 246 |
| 163 | 353 | 3640 | 822 | 273 | 935 | 72 | 192 | 101 | 708 | 654 | 246 |
| 106 | 224 | 2459 | 535 | 159 | 577 | 38 | 158 | 77 | 489 | 508 | 237 |
| 65 | 100 | 1455 | 432 | 160 | 273 | 38 | 80 | 37 | 289 | 259 | 244 |
| 147 | 237 | 3271 | 807.2 | 233.9 | 702.6 | 69.1 | 184.9 | 107.9 | 500 | 634.5 | 240.8 |
| 107 | 180 | 2342 | 2245 | 667 | 1966 | 237 | 474 | 249 | 1336 | 1767 | 941 |
| 145 | 346 | 3170 | 618.4 | 204.6 | 877.7 | 65.1 | 140.5 | 73.7 | 688.9 | 567.2 | 241.8 |
| 119 | 208 | 2842 | 682.5 | 203.3 | 615 | 55.2 | 152.4 | 82.8 | 513.2 | 559.7 | 232.3 |
| 125 | 319 | 3079 | 649.8 | 200.1 | 825.5 | 58 | 178 | 95.2 | 679.2 | 523.3 | 232.5 |
| 146 | 268 | 3343 | 817.4 | 266.4 | 777.9 | 71.8 | 156.2 | 90.2 | 597.1 | 606.8 | 242.1 |
| 104 | 196 | 2501 | 644.9 | 207 | 541.6 | 54.3 | 131.7 | 66.2 | 452.9 | 473 | 237.1 |
| 126 | 201 | 2609 | 603.3 | 214.3 | 528.5 | 74.1 | 119.3 | 67 | 400.8 | 463.5 | 229.5 |
| 135 | 311 | 3075 | 657.2 | 206.8 | 836 | 60.7 | 167 | 86.2 | 704.3 | 531.6 | 235.3 |
| 101 | 179 | 2390 | 632.7 | 209 | 474.9 | 52.1 | 146.5 | 83.7 | 426.4 | 419.9 | 236.4 |
| 83 | 180 | 1998 | 463.6 | 150.6 | 460.8 | 33.5 | 117.2 | 63 | 421.8 | 361 | 233.4 |
| 143 | 305 | 3286 | 714 | 224 | 826 | 57 | 186 | 96 | 696 | 559 | 230 |
| 152 | 272 | 3382 | 786.1 | 240.8 | 714.5 | 65.9 | 173.2 | 96.3 | 532.5 | 677.4 | 228.9 |
| 165 | 418 | 3558 | 699.9 | 234.3 | 1097.7 | 67.6 | 165.9 | 77 | 663.5 | 523.9 | 241.6 |
| 90 | 176 | 2158 | 527.5 | 187.9 | 513.1 | 59.8 | 104.5 | 60.7 | 429 | 459 | 252 |
| 127 | 302 | 3049 | 664.2 | 181.1 | 781.2 | 45.6 | 191.4 | 100.1 | 715.4 | 578.3 | 240.9 |
| 72 | 123 | 1669 | 466.6 | 163.1 | 341.4 | 42.3 | 97.2 | 49.8 | 315.2 | 290.5 | 240.6 |
| 109 | 200 | 2424 | 595.3 | 208.7 | 543.7 | 48.3 | 123 | 70 | 491.4 | 457.1 | 243.6 |
| 102 | 311 | 2548 | 508.2 | 137.9 | 812.8 | 36.8 | 147.3 | 72.1 | 729.7 | 433 | 244.2 |
| 75 | 112 | 1697 | 476.1 | 170.2 | 280.7 | 42.9 | 79.9 | 41 | 265 | 309.7 | 230.8 |
| 120 | 214 | 2757 | 843.8 | 252 | 687.6 | 55.5 | 195.8 | 117.7 | 528 | 713.1 | 296.2 |
| 102 | 179 | 2313 | 587.3 | 193.5 | 455.8 | 50.7 | 136.8 | 78 | 400.9 | 390.7 | 228.3 |
| 87 | 193 | 2140 | 544.3 | 177.1 | 498.6 | 42.5 | 130.8 | 65.7 | 481.9 | 400.6 | 244.9 |
| 156 | 261 | 3342 | 3012 | 887 | 2728 | 254 | 697 | 427 | 1932 | 2489 | 916 |
| 103 | 209 | 2318 | 505 | 149.5 | 534 | 46.6 | 120 | 68.4 | 397.3 | 451.8 | 233.8 |
| 141 | 235 | 2998 | 719.7 | 196.2 | 645.7 | 48.5 | 187.4 | 98.7 | 519.9 | 600 | 233.7 |
| 96 | 191 | 2235 | 582.5 | 190.9 | 600.5 | 66.2 | 122.1 | 67.8 | 445.5 | 531.8 | 279.5 |
| 117 | 223 | 2753 | 695.8 | 208 | 592.5 | 55.2 | 157.2 | 88.3 | 473.5 | 517.2 | 238 |
| 151 | 258 | 3111 | 717.8 | 214.8 | 667.5 | 62.7 | 169.2 | 96.4 | 504.9 | 605.7 | 234.7 |
| 215 | 455 | 4768 | 1092 | 306.6 | 1182.6 | 65.9 | 293 | 153.8 | 951.7 | 833.2 | 236.2 |
| 105 | 187 | 2300 | 517 | 177 | 479 | 67.7 | 100.4 | 56.5 | 333.6 | 465.5 | 235.7 |
| 153 | 333 | 3640 | 845 | 251.2 | 892.7 | 62.2 | 201.9 | 106.8 | 710.4 | 679.5 | 236.4 |
| 159 | 290 | 3384 | 783.7 | 228.5 | 783.5 | 65 | 172.3 | 93.4 | 567 | 620.7 | 235.1 |
| 116 | 294 | 2944 | 641.2 | 184.4 | 756.9 | 51.4 | 157.8 | 83.4 | 611.1 | 554.4 | 234.8 |
| 168 | 247 | 3289 | 781.3 | 242.4 | 651.5 | 76.4 | 143.8 | 74.9 | 458 | 702.3 | 238.6 |
| 124 | 256 | 2902 | 712 | 200.2 | 678.5 | 44.8 | 189.4 | 101.9 | 557.2 | 555.6 | 241.4 |
| 213 | 294 | 3849 | 942.9 | 310.1 | 738.6 | 76.8 | 200.1 | 114.2 | 462.2 | 704.7 | 240.7 |
| 97 | 163 | 2220 | 551.3 | 166.9 | 474.2 | 50.4 | 117.4 | 63.4 | 371.5 | 466.3 | 241.8 |
| 121 | 200 | 2727 | 712.1 | 222.1 | 551.4 | 49.9 | 162.5 | 85.2 | 484.3 | 545.7 | 239.9 |
| 152 | 365 | 3542 | 752.2 | 201 | 934.3 | 48.2 | 210.1 | 109.4 | 765.7 | 654.3 | 236.3 |
| 150 | 204 | 3246 | 1035.6 | 350.2 | 543.1 | 64.7 | 199.1 | 76.2 | 408.2 | 443.6 | 233.3 |
| 144 | 336 | 3241 | 682.7 | 195.7 | 847.5 | 55.5 | 188.2 | 94.1 | 660.2 | 569.3 | 234.7 |
| 128 | 282 | 2794 | 573.1 | 183.5 | 734.9 | 48.6 | 158.6 | 74.7 | 620.4 | 531.7 | 235.8 |
| 103 | 184 | 2489 | 579.6 | 181.3 | 507.6 | 58.2 | 122.5 | 68.4 | 321.4 | 508.5 | 230.1 |
| 143 | 202 | 2825 | 723.5 | 240.3 | 535.5 | 56.4 | 148.3 | 87.3 | 404.2 | 553.1 | 246.8 |
| 94 | 183 | 2278 | 579.3 | 182.3 | 505.9 | 46.2 | 138.9 | 78.2 | 433.3 | 440.2 | 242.7 |

| | | | | | | | | | | | |
|---|---|---|---|---|---|---|---|---|---|---|---|
| 135 | 239 | 3039 | 748.3 | 214.4 | 622.8 | 47.3 | 196.1 | 99.2 | 560.7 | 582.6 | 231.2 |
| 93 | 225 | 2211 | 457.1 | 181.7 | 591.6 | 54.3 | 105.1 | 52.8 | 542.4 | 350 | 236.9 |
| 138 | 346 | 3433 | 4002.4 | 1036.6 | 5145.3 | 281.9 | 1250.5 | 661.6 | 3058.6 | 3771.2 | 1342.8 |
| 60 | 91 | 1413 | 408.3 | 146.1 | 252.8 | 41 | 75 | 41.9 | 306.1 | 266 | 242.3 |
| 100 | 194 | 2374 | 555.7 | 174.9 | 507.6 | 55.4 | 115.8 | 62.9 | 453.8 | 463.8 | 230.1 |
| 99 | 223 | 2307 | 497.3 | 144.2 | 605.2 | 49.5 | 123.2 | 64.9 | 527.5 | 432.3 | 239.6 |
| 151 | 278 | 3413 | 928.6 | 298.2 | 724.6 | 51.4 | 243.6 | 144.9 | 605.1 | 577.5 | 245.7 |
| 158 | 221 | 3195 | 796 | 209 | 588 | 64 | 173 | 90 | 294 | 574 | 228 |
| 170 | 268 | 3510 | 929.5 | 296.6 | 740.3 | 66.7 | 192.9 | 118.8 | 512.2 | 617.1 | 242.7 |
| 159 | 252 | 3732 | 1028.7 | 206.4 | 615.9 | 32 | 379.9 | 171.6 | 513.4 | 659.8 | 222.2 |
| 121 | 204 | 2509 | 596.7 | 169.1 | 471.7 | 48.7 | 136.3 | 63.9 | 296.5 | 411 | 216.3 |
| 152 | 234 | 3184 | 937.8 | 194.3 | 635.7 | 54.5 | 225.8 | 75.8 | 413.9 | 633.9 | 247.1 |
| 77 | 139 | 1863 | 482.1 | 138.4 | 374.7 | 36.5 | 119.7 | 66.4 | 320.9 | 319.1 | 226.5 |
| 122 | 219 | 2860 | 747.3 | 161 | 540 | 40.4 | 209.8 | 88.5 | 414.4 | 566.4 | 226.8 |
| 80 | 148 | 1966 | 518.9 | 166.2 | 420 | 45.4 | 96.6 | 52.2 | 371.2 | 362.7 | 234.2 |
| 123 | 206 | 2677 | 802.3 | 182 | 553.4 | 54.6 | 179.8 | 57.7 | 346.4 | 463.4 | 238.7 |
| 105 | 186 | 2035 | 411.9 | 137.6 | 378.1 | 51.7 | 87.1 | 43.2 | 243 | 335.2 | 211.6 |
| 140 | 205 | 3144 | 876.1 | 181 | 545 | 32.3 | 260.8 | 123.8 | 417 | 582.8 | 224.7 |
| 119 | 188 | 2667 | 672.8 | 179.5 | 480.8 | 51.1 | 147.3 | 73.6 | 330.2 | 523.6 | 224.6 |
| 119 | 232 | 2746 | 622 | 182 | 627 | 61 | 136 | 76 | 423 | 533 | 234 |
| 123 | 191 | 2541 | 651.1 | 178 | 532.1 | 56.7 | 142.8 | 72 | 330.6 | 466.3 | 234.2 |
| 384 | 568 | 9835 | 3652.1 | 427.9 | 1546.7 | 52.2 | 1160.4 | 326.4 | 1076.2 | 1582.6 | 232.4 |
| 126 | 196 | 2587 | 679.1 | 190.3 | 561.1 | 56.2 | 151 | 78 | 352.3 | 529.4 | 249.9 |
| 203 | 462 | 5057 | 1226.9 | 304.65 | 1202.9 | 47.95 | 382.5 | 223 | 877.55 | 909.35 | 234.3 |
| 202 | 329 | 4389 | 1009 | 269 | 830 | 55 | 266 | 154 | 590 | 770 | 216 |
| 253 | 532 | 5957 | 1475 | 325 | 1364 | 57 | 499 | 266 | 970 | 1011 | 232 |
| 215 | 313 | 4919 | 1292.6 | 284.4 | 805.8 | 44.7 | 402.3 | 200.5 | 618.8 | 840.6 | 213.4 |
| 114 | 189 | 2555 | 654.6 | 173.6 | 475.9 | 39.5 | 152.5 | 84.8 | 367 | 519.2 | 228.9 |
| 150 | 284 | 3627 | 952.7 | 161.1 | 742.5 | 34.3 | 324 | 123.1 | 575.5 | 697 | 226.1 |
| 106 | 154 | 2083 | 499 | 134.8 | 335.7 | 39.2 | 119.1 | 54.1 | 260.4 | 331.7 | 205.4 |
| 169 | 268 | 3203 | 722 | 214 | 637 | 68 | 169 | 73 | 322 | 595 | 227 |
| 88 | 140 | 1964 | 516.3 | 137.4 | 380.2 | 41.7 | 120.8 | 57.8 | 232.1 | 384 | 237.6 |
| 182 | 390 | 4383 | 1097.7 | 209.9 | 1053 | 42.1 | 423 | 190.5 | 670.5 | 828.8 | 242.5 |
| 166 | 278 | 3801 | 883.5 | 196.5 | 637.8 | 46.5 | 246.3 | 113.2 | 410.7 | 592.5 | 195.1 |
| 142 | 267 | 3315 | 804.7 | 219 | 697.8 | 49.7 | 204.5 | 113.1 | 519.4 | 581.8 | 223 |
| 147 | 278 | 3334 | 814.8 | 221.9 | 720.3 | 53 | 192.1 | 103.1 | 504.8 | 532.7 | 223 |
| 319 | 482 | 6956 | 1934.8 | 457.9 | 1299.7 | 61.9 | 590.5 | 348.1 | 936.1 | 1095.1 | 232.1 |
| 279 | 437 | 7357 | 2311 | 91 | 1386 | 26 | 1421 | 31 | 2184 | 1073 | 223 |
| 1169 | 2130 | 20730 | 4762 | 179 | 4600 | 83 | 3209 | 71 | 6716 | 3428 | 226 |
| 188 | 298 | 4007 | 1062 | 302 | 858 | 77 | 234 | 137 | 560 | 787 | 247 |
| 114 | 187 | 2414 | 5695 | 1527 | 4362 | 487 | 1269 | 628 | 2338 | 4247 | 2165 |
| 170 | 319 | 3800 | 954 | 243 | 789 | 44 | 303 | 160 | 584 | 666 | 230 |
| 186 | 316 | 4226 | 1203 | 289 | 850 | 41 | 390 | 215 | 733 | 831 | 252 |
| 167 | 235 | 3319 | 1090 | 160 | 743 | 40 | 399 | 113 | 617 | 745 | 273 |
| 197 | 335 | 4312 | 1115 | 177 | 872 | 40 | 384 | 148 | 640 | 818 | 228 |
| 145 | 193 | 3101 | 880 | 190 | 564 | 47 | 231 | 98 | 353 | 594 | 237 |
| 93 | 146 | 1900 | 486 | 131 | 384 | 40 | 110 | 53 | 174 | 358 | 236 |
| 192 | 272 | 4151 | 1150 | 301 | 783 | 60 | 286 | 155 | 569 | 803 | 238 |
| 110 | 194 | 2588 | 739 | 186 | 580 | 42 | 188 | 97 | 461 | 569 | 258 |
| 141 | 226 | 3110 | 1076 | 202 | 660 | 45 | 273 | 91 | 487 | 639 | 264 |
| 162 | 278 | 3685 | 1173 | 248 | 740 | 85 | 279 | 98 | 535 | 699 | 247 |
| 113 | 191 | 2508 | 594 | 163 | 478 | 51 | 133 | 67 | 291 | 494 | 226 |
| 166 | 263 | 3678 | 1116 | 230 | 758 | 39 | 376 | 191 | 609 | 789 | 261 |
| 104 | 162 | 2293 | 674 | 183 | 415 | 46 | 153 | 68 | 336 | 412 | 235 |
| 129 | 264 | 2981 | 722 | 191 | 679 | 53 | 173 | 86 | 532 | 555 | 234 |
| 93 | 135 | 2026 | 754 | 223 | 472 | 63 | 161 | 73 | 395 | 518 | 314 |

| | | | | | | | | | | | |
|---|---|---|---|---|---|---|---|---|---|---|---|
| 121 | 207 | 2729 | 723 | 183 | 580 | 44 | 182 | 97 | 366 | 602 | 246 |
| 228 | 385 | 4860 | 1188 | 291 | 1079 | 67 | 311 | 163 | 686 | 970 | 240 |
| 147 | 225 | 3274 | 981 | 170 | 787 | 39 | 327 | 123 | 589 | 734 | 265 |
| 97 | 151 | 2283 | 650 | 168 | 441 | 45 | 141 | 61 | 342 | 477 | 242 |
| 155 | 229 | 3326 | 963 | 182 | 579 | 43 | 257 | 93 | 385 | 664 | 232 |
| 94 | 160 | 2157 | 519 | 160 | 411 | 48 | 103 | 60 | 295 | 401 | 225 |
| 384 | 616 | 8318 | 2229 | 495 | 1565 | 63 | 809 | 436 | 1144 | 1266 | 229 |
| 195 | 387 | 4689 | 1290 | 199 | 1053 | 38 | 510 | 183 | 773 | 845 | 240 |
| 177 | 385 | 4069 | 1066 | 277 | 1127 | 60 | 303 | 157 | 822 | 811 | 265 |
| 396 | 506 | 7216 | 2144 | 396 | 1135 | 122 | 532 | 112 | 1169 | 1505 | 242 |
| 139 | 231 | 3118 | 827 | 116 | 618 | 22 | 364 | 122 | 519 | 659 | 240 |
| 177 | 252 | 3695 | 1027 | 271 | 684 | 61 | 243 | 134 | 478 | 707 | 240 |
| 96 | 185 | 2161 | 585 | 176 | 583 | 51 | 140 | 74 | 468 | 430 | 269 |
| 147 | 269 | 3140 | 763 | 185 | 697 | 45 | 221 | 115 | 512 | 563 | 234 |
| 151 | 219 | 2809 | 797 | 197 | 595 | 51 | 203 | 82 | 404 | 547 | 258 |
| 130 | 239 | 2813 | 703 | 208 | 637 | 65 | 149 | 64 | 468 | 537 | 243 |
| 99 | 177 | 2191 | 606 | 168 | 503 | 50 | 142 | 74 | 362 | 441 | 257 |
| 290 | 365 | 6225 | 1815 | 454 | 1182 | 78 | 474 | 273 | 830 | 1071 | 238 |
| 96 | 173 | 2037 | 489 | 152 | 434 | 46 | 109 | 61 | 275 | 336 | 230 |
| 77 | 135 | 1598 | 349 | 142 | 345 | 52 | 73 | 43 | 244 | 286 | 244 |
| 132 | 226 | 2720 | 669 | 230 | 542 | 55 | 148 | 90 | 379 | 434 | 228 |
| 124 | 203 | 2464 | 657 | 204 | 533 | 46 | 170 | 97 | 243 | 353 | 242 |
| 136 | 187 | 2751 | 859 | 168 | 497 | 44 | 212 | 67 | 348 | 496 | 237 |
| 150 | 253 | 3156 | 753 | 242 | 690 | 72 | 154 | 89 | 487 | 576 | 239 |
| 123 | 198 | 2667 | 682 | 193 | 558 | 54 | 154 | 82 | 376 | 493 | 236 |
| 129 | 192 | 2587 | 629 | 185 | 463 | 57 | 122 | 60 | 281 | 476 | 224 |
| 123 | 199 | 2557 | 596 | 187 | 517 | 55 | 120 | 70 | 371 | 455 | 227 |
| 106 | 151 | 2231 | 630 | 168 | 402 | 41 | 155 | 76 | 340 | 450 | 239 |
| 120 | 202 | 2599 | 617 | 163 | 496 | 58 | 126 | 54 | 370 | 506 | 227 |
| 133 | 237 | 2822 | 707 | 196 | 616 | 50 | 176 | 100 | 471 | 522 | 240 |
| 124 | 197 | 2595 | 644 | 169 | 583 | 49 | 179 | 76 | 372 | 527 | 247 |
| 141 | 261 | 3269 | 842 | 167 | 752 | 41 | 269 | 109 | 597 | 582 | 233 |
| 247 | 338 | 4849 | 9281.3 | 2290.9 | 6971.4 | 712.8 | 2073.9 | 790.1 | 4848.5 | 6995.7 | 1739.1 |
| 354 | 435 | 5821 | 12857.9 | 1856.3 | 7640.9 | 462.7 | 4332.2 | 758.3 | 6722 | 8496.8 | 1808.6 |
| 140 | 190 | 2971 | 6807.8 | 1381.8 | 4011.6 | 341.6 | 1959.7 | 558.8 | 4090.8 | 4027.6 | 1756.5 |
| 149 | 236 | 3270 | 7592.9 | 1411.9 | 5359.1 | 355.7 | 2477.8 | 738.3 | 4041 | 5112.2 | 1931.9 |
| 145 | 218 | 3373 | 8200 | 1559.7 | 5165 | 325.9 | 2677.8 | 853.4 | 4968.8 | 5495.4 | 1924 |
| 239 | 392 | 5511 | 12498.3 | 2386.4 | 8457.2 | 391.4 | 4424.2 | 1978.5 | 9085.4 | 8087.2 | 1878.5 |
| 122 | 215 | 2969 | 6669.1 | 1307.4 | 4775.7 | 252.5 | 2497.9 | 937.3 | 4710.6 | 4230.6 | 1865.5 |
| 99 | 122 | 2387 | 6519.7 | 1416.1 | 2906.6 | 281.5 | 1614.9 | 550.7 | 3406.9 | 3099.9 | 1785.3 |
| 235 | 300 | 4642 | 10372 | 1628.6 | 6329.7 | 419.7 | 2958.5 | 811.5 | 5251 | 7062.5 | 1798.8 |
| 323 | 409 | 5100 | 10778.7 | 1625 | 6882.3 | 558.1 | 3137.5 | 609.9 | 5686.5 | 8290 | 1879.7 |
| 185 | 297 | 4216 | 9741.5 | 1597.1 | 6604.3 | 323.1 | 3037.7 | 1086.6 | 5585.6 | 6139.8 | 1822.2 |
| 107 | 184 | 2523 | 5552.9 | 1574.9 | 3517.7 | 417 | 1243.8 | 435 | 3537.3 | 3373.7 | 1774.8 |
| 126 | 178 | 2802 | 6514.3 | 1658 | 3658.6 | 437.9 | 1449.4 | 480.8 | 3703.5 | 3872.7 | 1776.3 |
| 294 | 362 | 4927 | 11138.1 | 2001.1 | 6000 | 469 | 3690 | 807.3 | 6575.6 | 7596.5 | 1845.7 |
| 214 | 290 | 4115 | 9310.6 | 1693.9 | 5247.5 | 466.4 | 2530.1 | 581.9 | 5184.2 | 6117.2 | 1776.2 |
| 135 | 186 | 2833 | 6601.1 | 1447 | 3653 | 333.8 | 1749.7 | 573 | 3715.1 | 3794.2 | 1776.2 |
| 169 | 267 | 3605 | 7921.2 | 1206.5 | 5713 | 279.4 | 2924.4 | 907.5 | 4536.9 | 5116.2 | 1814.2 |
| 131 | 194 | 3062 | 7651 | 1243.9 | 4354.9 | 291.2 | 2160.3 | 605.3 | 3942 | 4498.9 | 1847.9 |
| 255 | 346 | 4309 | 9434.1 | 1717.2 | 5901.6 | 528.4 | 2415.4 | 574.6 | 4618 | 6507.1 | 1850.6 |
| 210 | 313 | 4260 | 9644.6 | 1276 | 6243.8 | 290.3 | 3534.3 | 848.9 | 5432.2 | 6103.1 | 1807.7 |
| 210 | 288 | 3848 | 8300.6 | 1467.6 | 4986.6 | 403.6 | 2471.3 | 603.3 | 5007.9 | 5694 | 1789.6 |
| 206 | 313 | 4137 | 8582.8 | 1573.7 | 5697.1 | 394.9 | 2518.9 | 722.4 | 5043.3 | 5875.2 | 1729 |
| 273 | 448 | 5359 | 12210.8 | 1487.5 | 8444.3 | 386.3 | 4447.3 | 836.2 | 7474.1 | 8333.5 | 1890.7 |
| 308 | 488 | 5832 | 12613.2 | 1583 | 8943.3 | 382.8 | 4756.5 | 1058.5 | 7028.8 | 8505.7 | 1817.1 |

| | | | | | | | | | | | |
|---|---|---|---|---|---|---|---|---|---|---|---|
| 168 | 308 | 3680 | 7036.8 | 1907.2 | 6279.7 | 569.5 | 1641.1 | 812.1 | 4701.4 | 5258.3 | 1803.4 |
| 192 | 372 | 4507 | 8924.8 | 1659.1 | 7561.5 | 362 | 3179.9 | 1155 | 5744.9 | 5943 | 1758.2 |
| 500 | 638 | 7623 | 15211.5 | 1908 | 10258.7 | 619.7 | 6718.7 | 765.2 | 9150.8 | 10999.9 | 1839 |
| 366 | 526 | 6621 | 15083.5 | 1829.8 | 9406.4 | 455.3 | 5866.9 | 1076.7 | 7980.6 | 10333.9 | 1888.6 |
| 98 | 139 | 2374 | 5972.2 | 1602 | 3122.1 | 377 | 1261.8 | 489.3 | 3047.4 | 3303.7 | 1853.6 |
| 116 | 158 | 2562 | 6309.8 | 1284.8 | 3740.3 | 284.4 | 1921.7 | 723.8 | 3234.5 | 3969.2 | 1905.7 |
| 948 | 1211 | 13503 | 28300 | 2795 | 17319.2 | 871.4 | 10586.8 | 925.1 | 14823 | 20894.5 | 1864.3 |
| 259 | 397 | 6193 | 12683.2 | 2182.1 | 8915.7 | 383.4 | 4743.5 | 2078.4 | 7764.5 | 9888.3 | 1804.9 |
| 115 | 204 | 2758 | 6435 | 1379.8 | 4496 | 281 | 2013.5 | 849.3 | 4288.2 | 4235.9 | 1930.5 |
| 150 | 323 | 3654 | 7453.3 | 1157.2 | 7171.5 | 303.5 | 2828 | 718 | 6211.7 | 5043.9 | 1833 |
| 149 | 258 | 3368 | 7020.2 | 1185.3 | 5897 | 294.5 | 2534.1 | 820.8 | 4671.6 | 5019.7 | 1840.1 |
| 153 | 211 | 2975 | 7316.6 | 1536.7 | 4433.8 | 380.3 | 1918.5 | 596.6 | 4006.6 | 4626.7 | 1977.7 |
| 183 | 275 | 3895 | 8297.2 | 1836.1 | 6610.3 | 512.1 | 2116.4 | 909.3 | 4694.6 | 5880.8 | 1871.4 |
| 131 | 194 | 2865 | 6414.8 | 1305.3 | 4133.9 | 308.2 | 1961.1 | 768.2 | 3933.3 | 4229.2 | 1822.6 |
| 312 | 496 | 7368 | 16265.5 | 3301.2 | 10433.1 | 504.6 | 5990.1 | 2895.9 | 8114.8 | 9878.9 | 1813.3 |
| 152 | 251 | 3270 | 6598.9 | 1807.7 | 5375.1 | 554.1 | 1511.1 | 596.5 | 4355.8 | 4608.6 | 1841.2 |
| 435 | 682 | 9452 | 20243.8 | 2246.7 | 13634.4 | 383.5 | 11147.8 | 2909.4 | 15903.3 | 11706.4 | 1833.8 |
| 198 | 266 | 3906 | 8176 | 2206.2 | 6023.7 | 548.6 | 1893.9 | 1070.7 | 4123 | 6938.1 | 1997.6 |
| 194 | 251 | 4246 | 9039.3 | 1803.7 | 5626.2 | 385.8 | 2751.7 | 1267.3 | 4702.5 | 6865.4 | 1782.7 |
| 244 | 321 | 5070 | 11147.8 | 2224.3 | 7233 | 560.9 | 2685 | 1049 | 5801.4 | 8128.4 | 1807 |
| 288 | 430 | 5490 | 10844.3 | 2385.4 | 8149.8 | 783.5 | 2652.3 | 904.2 | 5626.7 | 7425 | 1725.9 |
| 181 | 237 | 3494 | 7762.2 | 1819.1 | 4594.4 | 531.7 | 1727.9 | 580.9 | 4293.7 | 5341.3 | 1823 |
| 146 | 201 | 3209 | 7475.8 | 1884.3 | 4487.6 | 456.8 | 1705 | 677.5 | 4457.4 | 4795.9 | 1835.8 |
| 295 | 388 | 5337 | 13113.7 | 2688.6 | 7234 | 829.3 | 2875.4 | 845.4 | 6115.3 | 7681.2 | 1872.5 |
| 126 | 207 | 3048 | 7205.9 | 1470.9 | 4544.6 | 289 | 2213.2 | 782.5 | 4886.6 | 4332.8 | 1813.2 |
| 175 | 313 | 3620 | 7128.1 | 2091.8 | 6188.2 | 523.4 | 1615.6 | 863.6 | 4790.6 | 5374.2 | 1880.3 |
| 102 | 176 | 2508 | 5919.3 | 1354.8 | 4164.3 | 268.5 | 1754.9 | 723.3 | 4117.1 | 4357.7 | 1979.5 |
| 144 | 201 | 3206 | 7367.8 | 1622.7 | 4205.9 | 414.7 | 1631.4 | 644.7 | 3595.5 | 4750.1 | 1771.8 |
| 344 | 460 | 5953 | 12768.1 | 1997.7 | 7854.1 | 702.4 | 3671 | 615.9 | 7614.4 | 9230.8 | 1857.9 |
| 91 | 152 | 2230 | 4936.1 | 1449.3 | 3089.2 | 336.5 | 1031.6 | 531.2 | 3031.1 | 3404.4 | 1821.9 |
| 221 | 406 | 4875 | 10119.3 | 1224.9 | 7655.1 | 264.9 | 5141.3 | 1232.4 | 8314.9 | 6619.7 | 1835.1 |
| 108 | 230 | 2630 | 4883.6 | 1744.7 | 4773.2 | 554.2 | 965 | 539.6 | 4048.4 | 3728.3 | 1896.8 |
| 172 | 280 | 3695 | 7839.2 | 1656.3 | 6534.7 | 385.2 | 2640.3 | 1086.8 | 5910.8 | 5388.2 | 1915.5 |
| 138 | 240 | 3265 | 7379.8 | 1213.7 | 5478.8 | 271.2 | 2741.9 | 872.1 | 5240.1 | 4835.7 | 1878.4 |
| 307 | 403 | 5312 | 12372 | 1727.7 | 7092 | 417.3 | 3885.2 | 811.8 | 6609.1 | 8488 | 1910.8 |
| 201 | 288 | 4395 | 9180.6 | 2576.6 | 6442 | 575.1 | 1890.3 | 1087.2 | 5007.7 | 6829.7 | 1821.1 |
| 138 | 210 | 2774 | 6034.5 | 1550.7 | 4047.2 | 472.9 | 1328.3 | 529.7 | 3744 | 3793.3 | 1819.9 |
| 112 | 189 | 2649 | 4587.7 | 1258.2 | 3239.3 | 292.6 | 1135 | 562.6 | 3148.9 | 2944.1 | 1450.7 |
| 129 | 197 | 2641 | 5541.7 | 1597.7 | 3869.5 | 443.6 | 1154.3 | 567.9 | 3041.2 | 3772.7 | 1826.2 |
| 175 | 436 | 4159 | 7340.1 | 2407.2 | 8745.3 | 642.9 | 2099.7 | 931.1 | 7286.9 | 5249.8 | 1884.5 |
| 103 | 274 | 3210 | 6684.1 | 2141.4 | 5619.2 | 455.2 | 1440.6 | 840 | 4498 | 4499 | 1819.8 |
| 159 | 255 | 3295 | 6361.2 | 1942.3 | 5270.9 | 560.7 | 1234.3 | 677.2 | 3370.2 | 4612.7 | 1796.2 |
| 187 | 270 | 3790 | 7134.4 | 2221.6 | 5727.7 | 607.2 | 1366 | 811.1 | 3915.3 | 5707.8 | 1830.7 |
| 150 | 293 | 3316 | 6656.1 | 1620.6 | 6055.4 | 484.7 | 1663 | 715.5 | 4691.3 | 4681.3 | 1851 |
| 136 | 249 | 3047 | 6287.6 | 1662.2 | 5128 | 377.7 | 1777.3 | 784.8 | 4444.4 | 4338.2 | 1849.8 |
| 174 | 311 | 3632 | 6979.4 | 1874 | 6328.9 | 394.4 | 2094.5 | 1063.3 | 5954.6 | 4839.3 | 1855.6 |
| 119 | 188 | 2562 | 5421.3 | 1686.5 | 3827.1 | 460.4 | 1158.5 | 598.9 | 3279.8 | 3883.7 | 1885.9 |
| 130 | 246 | 3168 | 6507.9 | 1727.2 | 5085 | 461.7 | 1492.8 | 733.1 | 4405.9 | 4682.3 | 1811.2 |
| 132 | 230 | 3141 | 6372.8 | 1788.5 | 4854.6 | 426.5 | 1468.3 | 805.2 | 3763.6 | 4975.9 | 1837.4 |
| 173 | 255 | 3446 | 7712.7 | 1907.4 | 5246.6 | 560.1 | 1394 | 597.2 | 3905.3 | 5134.7 | 1859.4 |
| 283 | 345 | 5487 | 12266.4 | 2952.9 | 8164.6 | 827.1 | 2903.4 | 1201.1 | 5638.8 | 7631.4 | 1854.2 |
| 150 | 273 | 3509 | 6985.5 | 1946 | 5820.5 | 422 | 1663.8 | 938.9 | 4162.3 | 5649 | 1873 |
| 117 | 232 | 2771 | 5270.2 | 1750.5 | 4683.1 | 384.5 | 1282.9 | 673.7 | 4366.2 | 3854.8 | 1821.9 |
| 101 | 184 | 2480 | 5340.3 | 1493.3 | 3978.3 | 385.2 | 1295.9 | 664.6 | 3662.5 | 3574.7 | 1873.8 |
| 106 | 180 | 2432 | 5303.4 | 1665.3 | 3808.3 | 455.9 | 1068.5 | 623.1 | 3253.1 | 3597.2 | 1915 |
| 113 | 194 | 2693 | 5490.3 | 1699.3 | 3900.2 | 402.8 | 1231.1 | 632.6 | 3845 | 3667.4 | 1755.1 |

| | | | | | | | | | | | |
|---|---|---|---|---|---|---|---|---|---|---|---|
| 297 | 427 | 6587 | 14200.1 | 3391.1 | 10113.6 | 597.1 | 4143.9 | 2383.5 | 7774.8 | 8963.4 | 1842.7 |
| 111 | 214 | 2539 | 5205.4 | 1589.5 | 4294.9 | 378.6 | 1239.6 | 634.7 | 3905.5 | 3866.4 | 1910.5 |
| 91 | 223 | 2491 | 4974.4 | 1725.2 | 4677.7 | 441.9 | 1035.8 | 568 | 4104.4 | 3069.9 | 1826.4 |
| 134 | 203 | 2931 | 6074 | 2018.7 | 4157.6 | 545.4 | 1145.5 | 691.5 | 3211.2 | 4537.2 | 1868.9 |
| 206 | 396 | 4156 | 7283.7 | 2431.5 | 7428 | 567.2 | 1711.8 | 1013 | 5992.7 | 5478.2 | 1853.5 |
| 109 | 171 | 2635 | 5771.7 | 1410.9 | 3578.9 | 292 | 1591.3 | 765.6 | 3493.3 | 4107.1 | 1786.9 |
| 88 | 164 | 2240 | 4877.9 | 1450.5 | 3396.3 | 331.9 | 1219.3 | 642.3 | 3210.5 | 3125.7 | 1837.2 |
| 88 | 125 | 2012 | 4835.3 | 1342.4 | 2763.4 | 264.9 | 1334.4 | 669.8 | 2756.4 | 2941.8 | 1904.1 |
| 93 | 184 | 2344 | 4789.2 | 1682.7 | 3693 | 503.6 | 895 | 473.4 | 3368.1 | 3098.9 | 1827 |
| 131 | 253 | 2899 | 5349.2 | 1701.8 | 5054.6 | 596.6 | 1049.2 | 553.5 | 4043 | 4263.3 | 1858.5 |
| 110 | 206 | 2653 | 5510.3 | 1540.9 | 4388.2 | 416.8 | 1217.4 | 610.8 | 3868.4 | 4102.9 | 1846.5 |
| 167 | 354 | 3982 | 7770.8 | 2193.7 | 7748.8 | 538.1 | 1911.3 | 1057.2 | 6395.3 | 5545.2 | 1901.5 |
| 246 | 479 | 5428 | 10255.2 | 2869.9 | 9262.7 | 583.8 | 2735.6 | 1572.5 | 8262.2 | 7761.9 | 1848.3 |
| 154 | 313 | 3584 | 6577.4 | 2116.2 | 6440.9 | 590.4 | 1402.7 | 811.6 | 5463.4 | 5206.3 | 1840.4 |
| 230 | 436 | 5206 | 9526.8 | 2378.4 | 8259.2 | 478.6 | 2921.6 | 1630.8 | 6585.7 | 6960.7 | 1725.9 |
| 182 | 412 | 4180 | 7246.2 | 2005.3 | 8185.1 | 491.8 | 1990.1 | 1085.4 | 6564.9 | 5595.4 | 1832.7 |
| 129 | 255 | 3016 | 5336.3 | 1487.5 | 5273.8 | 450.2 | 1259.2 | 714 | 4036.3 | 4618.3 | 1827.8 |
| 128 | 235 | 2889 | 5277.7 | 1638.7 | 4831.8 | 519.1 | 1053.4 | 579.7 | 3438.2 | 4280.7 | 1804.2 |
| 156 | 323 | 3615 | 6575.9 | 1870.9 | 6536.9 | 533.6 | 1608.4 | 869.6 | 5692.8 | 5351.8 | 1837.4 |
| 142 | 296 | 3435 | 6426.9 | 1566.8 | 6964.8 | 443.8 | 1835.2 | 833.7 | 5589.2 | 4815.8 | 1869.5 |
| 165 | 295 | 3736 | 7159.8 | 1978.2 | 6324.7 | 516.8 | 1869.1 | 1061.1 | 4878.5 | 5078.4 | 1837.2 |
| 244 | 415 | 5183 | 9877.1 | 2971.6 | 8633 | 687.2 | 2240.7 | 1365.3 | 6257.6 | 6943 | 1837.5 |
| 147 | 254 | 3351 | 6505.2 | 1685.8 | 5084.2 | 483.8 | 1481 | 785.9 | 4380.1 | 5552.2 | 1832.4 |
| 162 | 306 | 3556 | 6752.2 | 1997.4 | 5995.4 | 552.5 | 1626.6 | 813 | 5056.3 | 5328.1 | 1847.5 |
| 129 | 224 | 2858 | 5857.3 | 1773.3 | 4497.4 | 498.7 | 1199.1 | 636.2 | 3710.2 | 4227.6 | 1849.5 |
| 101 | 177 | 2301 | 4814.9 | 1575.3 | 3488.5 | 471.4 | 893.5 | 449.5 | 3348.1 | 3290.9 | 1841.8 |
| 263 | 512 | 5458 | 9554.6 | 2610.2 | 10355.7 | 654.7 | 2420.4 | 1339.8 | 7441.8 | 7334.7 | 1845.9 |
| 120 | 215 | 2766 | 5266.4 | 1625.7 | 4403.4 | 500.9 | 1066.1 | 581.3 | 3607.9 | 4506.1 | 1849.2 |
| 136 | 465 | 3641 | 5330 | 2083.2 | 9212.4 | 607 | 1308.8 | 554.5 | 7950.4 | 4203.7 | 1873.1 |
| 268 | 409 | 4829 | 8850.5 | 3106.7 | 7966.8 | 759 | 1677.7 | 1002.9 | 4945.5 | 6142.3 | 1828.2 |
| 164 | 321 | 3751 | 7137.4 | 2216.7 | 6006.2 | 559 | 1568.3 | 891.2 | 5023.7 | 5239.9 | 1793 |
| 241 | 541 | 5281 | 8986.1 | 2925.7 | 10484.6 | 855.8 | 1901.9 | 1129.6 | 7524.2 | 6524.9 | 1826.9 |
| 158 | 249 | 3634 | 6685.6 | 2027.9 | 5064 | 602 | 1327.8 | 800.5 | 3546.1 | 5436.9 | 1733.3 |
| 153 | 272 | 3676 | 8088.4 | 2298.2 | 5736.8 | 536.2 | 1432.2 | 815.5 | 4424.1 | 5165.2 | 1811.4 |
| 141 | 269 | 3268 | 6381.3 | 1817.9 | 5806.3 | 425.7 | 1597.9 | 806.5 | 4046.8 | 4606.1 | 1855.3 |
| 256 | 447 | 5576 | 10921.4 | 3270.9 | 8520.7 | 553 | 2892.8 | 1802.2 | 8157.9 | 7971.7 | 1830.5 |
| 89 | 152 | 2137 | 5914.1 | 1915.7 | 4327.8 | 460.8 | 1461.8 | 672.9 | 4030.4 | 4153.1 | 2451.7 |
| 128 | 287 | 3082 | 5580.1 | 1568.4 | 6577.9 | 391.4 | 1681.7 | 694.2 | 6418 | 4074.6 | 1870.9 |
| 101 | 143 | 2205 | 4815.8 | 1332.5 | 3013.3 | 299.7 | 1228.4 | 629.6 | 3204.8 | 3540.6 | 1862.3 |
| 147 | 316 | 3182 | 5373.6 | 1711.7 | 6084.2 | 443.1 | 1525.3 | 640.9 | 5565.3 | 4349.4 | 1841.5 |
| 105 | 171 | 2400 | 5986.4 | 1752.6 | 3975.9 | 420.3 | 1194.8 | 565.6 | 2936.4 | 3804.4 | 2054.3 |
| 119 | 278 | 2963 | 5579.4 | 1913.9 | 5695.8 | 502.2 | 1313.1 | 703 | 4742.2 | 4208.5 | 1893.5 |
| 159 | 418 | 4008 | 7082 | 1870.9 | 8773.7 | 544 | 1964.1 | 1055.5 | 6696.1 | 5905.3 | 1935.9 |
| 108 | 156 | 2291 | 5399.2 | 1453.4 | 3460.5 | 295.4 | 1520.8 | 699.8 | 3408.6 | 3364.1 | 1945.6 |
| 126 | 174 | 2635 | 6179.8 | 1638.9 | 3312.7 | 354 | 1472.4 | 596.1 | 3155.9 | 3744.3 | 1827.2 |
| 140 | 265 | 3317 | 6370.9 | 1846.3 | 5818.5 | 417.3 | 1662.8 | 865.3 | 5302.9 | 4899.7 | 1847.5 |
| 122 | 173 | 2763 | 6806.6 | 2104.5 | 3464.4 | 471.9 | 1090.8 | 483.3 | 3241.1 | 3350 | 1747.4 |
| 272 | 426 | 6260 | 14336.2 | 2094.3 | 9574.5 | 402.3 | 5782 | 1750.7 | 7908.5 | 8292.9 | 1842.6 |
| 228 | 550 | 5530 | 9030.7 | 2975.6 | 10475.3 | 813.3 | 1971.8 | 1171.3 | 8489.8 | 6898.7 | 1742.9 |
| 103 | 152 | 2274 | 5548.9 | 1457.1 | 3074.3 | 317.3 | 1182.2 | 527.4 | 2824.4 | 3257.8 | 1859 |
| 381 | 465 | 6232 | 14623 | 1956.4 | 7678.5 | 653.4 | 4209.8 | 507.9 | 8154 | 9224.6 | 1830.5 |
| 107 | 169 | 2329 | 4996.6 | 1418.3 | 3358 | 350.9 | 1187 | 585.5 | 2981.9 | 3418 | 1832.6 |
| 151 | 294 | 3628 | 7117 | 2368.4 | 5860 | 499.5 | 1583.3 | 993.7 | 5089.2 | 5023.4 | 1823.5 |
| 175 | 336 | 4111 | 7302.3 | 2258.3 | 6041.1 | 648.3 | 1464.5 | 740 | 4025.1 | 5157 | 1609.8 |
| 106 | 170 | 2282 | 5004.4 | 1588.5 | 3400.6 | 396 | 1152.4 | 602.2 | 3005.7 | 3107.9 | 1853.5 |
| 89 | 169 | 2252 | 4723.7 | 1544.8 | 3702.1 | 389.1 | 1070.4 | 559.1 | 3703.2 | 2973.7 | 1818 |

| | | | | | | | | | | | |
|---|---|---|---|---|---|---|---|---|---|---|---|
| 156 | 323 | 3678 | 6667.7 | 2057.3 | 6583.6 | 510.1 | 1580.5 | 842.5 | 5600.6 | 5431 | 1843.9 |
| 126 | 238 | 2712 | 4925.9 | 1738.4 | 4422.2 | 569.5 | 947 | 547 | 3887.4 | 3942.6 | 1838.7 |
| 142 | 196 | 2895 | 5427 | 1701.6 | 4072.8 | 495.9 | 1049.7 | 629.4 | 2793.8 | 4436.2 | 1781.6 |
| 71 | 113 | 1893 | 4980.1 | 1586.1 | 3027.8 | 386.8 | 974 | 490.7 | 2912.8 | 3041 | 2058.5 |
| 131 | 233 | 3087 | 6968.8 | 1788.1 | 5369.8 | 380 | 2046.3 | 852.5 | 5210 | 4348.9 | 1917.5 |
| 175 | 435 | 4438 | 8426.2 | 2345.2 | 10004.2 | 573.8 | 2297.3 | 1200.4 | 7752.8 | 6468.6 | 1988.7 |
| 158 | 393 | 3695 | 6150 | 2045.8 | 7608.3 | 643 | 1435.9 | 783.5 | 5429.4 | 4694.2 | 1825.8 |
| 189 | 288 | 4118 | 9592.2 | 1637.4 | 6426.7 | 367.6 | 2731.7 | 982.7 | 5008.1 | 6331.2 | 1851.2 |
| 180 | 339 | 3969 | 7465.3 | 2291.2 | 6484.4 | 499.3 | 1716.6 | 952.9 | 4798 | 5555.4 | 1819.8 |
| 125 | 180 | 2573 | 5814.7 | 1647.6 | 3815.2 | 416 | 1297 | 582.2 | 3112.7 | 4165.2 | 1924.6 |
| 82 | 132 | 2015 | 4736.7 | 1639 | 2889.1 | 363.1 | 936 | 442.8 | 2975.4 | 2633.8 | 1837.2 |
| 120 | 210 | 2875 | 6388.4 | 1830.9 | 5055.8 | 435.9 | 1641.3 | 831.8 | 4664.5 | 4520.5 | 2000.4 |
| 265 | 664 | 6711 | 9994.1 | 2626 | 12334.4 | 721.1 | 3011.6 | 1695.5 | 9733.2 | 8948.4 | 1679.4 |
| 82 | 153 | 1985 | 4284.7 | 1317.8 | 3417.3 | 340.8 | 1017.1 | 527.6 | 3343.5 | 2985.8 | 1943.8 |
| 79 | 159 | 1955 | 4101.6 | 1542.6 | 3242.3 | 434.7 | 752.8 | 457.7 | 2926.6 | 2830.9 | 1944.1 |
| 124 | 299 | 3004 | 5662 | 1909 | 6802.2 | 654.4 | 1177.2 | 630.6 | 5161.4 | 4654.6 | 2074.9 |
| 85 | 143 | 2015 | 4527.9 | 1814.9 | 3028.8 | 456.8 | 852.3 | 409.2 | 3130.8 | 3074.9 | 1997 |
| 123 | 362 | 3140 | 4928.9 | 1713.6 | 7727.3 | 507.2 | 1238.8 | 545.7 | 6696.4 | 3801.6 | 1906.7 |
| 195 | 482 | 4326 | 7053.7 | 2434.2 | 9349.6 | 648.1 | 1634.5 | 893 | 7799.4 | 6006.6 | 1892.3 |
| 137 | 269 | 3238 | 5909.4 | 1618.4 | 5455.7 | 350 | 1797 | 821.9 | 5376.9 | 4679.8 | 1772.2 |
| 208 | 407 | 4707 | 7941.4 | 2755.6 | 7473 | 965.5 | 1353.2 | 761.5 | 5276.2 | 6095.2 | 1677.5 |
| 153 | 339 | 3579 | 6033.9 | 1977.3 | 6958.1 | 617.9 | 1282.9 | 744.9 | 5057.9 | 5053 | 1856.7 |
| 199 | 419 | 4394 | 7841.2 | 2603.3 | 8429.3 | 673 | 1773.8 | 976.5 | 6582.3 | 6077.1 | 1902.3 |
| 202 | 245 | 3866 | 8047.5 | 2151.4 | 5191.9 | 558.8 | 1709.5 | 732.2 | 3754.5 | 6039.4 | 1808.3 |
| 132 | 211 | 2876 | 6439.5 | 2306.3 | 4598.1 | 604.4 | 1234.6 | 700.3 | 4136.2 | 4543.3 | 2047 |
| 87 | 260 | 2393 | 3876.8 | 1556.1 | 5510 | 402.2 | 875.7 | 421.6 | 5142.4 | 3059.3 | 1877.6 |
| 107 | 162 | 2731 | 6074.3 | 1968.9 | 3149 | 407.3 | 1171.7 | 542.3 | 3792.3 | 3380.7 | 1628.9 |
| 180 | 195 | 3521 | 8930.2 | 1739.3 | 4185.1 | 447 | 1902.1 | 527.3 | 4318.7 | 5082.5 | 1795.6 |
| 116 | 236 | 2905 | 5865 | 2074 | 5250.4 | 564.7 | 1069.6 | 639.2 | 4340.3 | 4508.3 | 1954.7 |
| 65 | 162 | 1726 | 3305.2 | 1368.3 | 3480.9 | 464.7 | 563.9 | 362.8 | 2644.6 | 2247.8 | 1970.9 |
| 107 | 315 | 2819 | 4542.7 | 1645.3 | 6578.6 | 490.7 | 1106.9 | 485.8 | 5769.3 | 3441.8 | 1847.5 |
| 177 | 439 | 4259 | 7834.4 | 2444.8 | 9012.4 | 633.8 | 1908.9 | 1027.9 | 7404.9 | 6089.6 | 1928.3 |
| 215 | 469 | 4843 | 9024.8 | 2888.1 | 10013.1 | 706.9 | 2140 | 1153.6 | 5971.6 | 6529.9 | 1962.8 |
| 150 | 319 | 3506 | 6745.8 | 2364.8 | 7321.1 | 727.3 | 1339.8 | 761.8 | 3852.8 | 5248.6 | 2013.7 |
| 152 | 267 | 3320 | 6791.9 | 2424.4 | 6106.1 | 693.1 | 1238.4 | 757.8 | 4427.1 | 5201.7 | 2020.9 |
| 187 | 346 | 4222 | 7883.9 | 2013.2 | 7074.5 | 497.8 | 2284.2 | 1261.9 | 5605.5 | 6210.5 | 1852.6 |
| 103 | 189 | 2343 | 4586.8 | 1327.4 | 4060.9 | 416.4 | 1022.1 | 528.6 | 3043.7 | 3835.8 | 1922.2 |
| 176 | 259 | 3529 | 7812.9 | 1493.1 | 4915 | 435.1 | 2357.3 | 635.9 | 5446.4 | 5781.3 | 1893.2 |
| 131 | 266 | 3033 | 5705.6 | 2008.3 | 5401.8 | 581.2 | 1071.3 | 601.6 | 4270.4 | 4290 | 1880.2 |
| 182 | 420 | 3969 | 6487.5 | 2199.1 | 8110 | 635.5 | 1398.4 | 751.9 | 6354.8 | 5499.8 | 1875.1 |
| 107 | 162 | 2526 | 6214 | 1998.1 | 3669.3 | 430 | 1304.4 | 599.4 | 3596.7 | 3744 | 1944.2 |
| 149 | 326 | 3512 | 6887.9 | 2455.3 | 6953 | 571 | 1625.6 | 803.5 | 6113.1 | 5024 | 1976.4 |
| 112 | 236 | 2820 | 5619.8 | 1991.5 | 4862 | 599.9 | 1034.1 | 589 | 4150.9 | 4035.9 | 1906.4 |
| 113 | 223 | 2782 | 5995.9 | 1799.5 | 4838.1 | 416.1 | 1561.3 | 844.8 | 4682.1 | 4562.6 | 2007.7 |
| 99 | 184 | 2434 | 4803.8 | 1646.8 | 3702.3 | 466.1 | 874 | 495.9 | 2951.1 | 3734.4 | 1853.8 |
| 176 | 375 | 4267 | 8174.7 | 2523.2 | 7390.3 | 499.9 | 1975.1 | 1193.6 | 5950.3 | 5786.3 | 1804.6 |
| 137 | 230 | 3299 | 6972 | 2210.4 | 4807.3 | 510.5 | 1421.2 | 795.8 | 4313 | 5131 | 1840.5 |
| 165 | 300 | 3675 | 7384.4 | 2675.2 | 5756.8 | 565.4 | 1449.1 | 907.1 | 4629.4 | 5120.1 | 1883.4 |
| 114 | 200 | 2589 | 5135.5 | 1880.6 | 4035.1 | 531.5 | 888.5 | 534.4 | 3445.5 | 3863.4 | 1891.9 |
| 125 | 199 | 2872 | 6698.3 | 1838.9 | 4389.4 | 335 | 2207.4 | 892.7 | 4387 | 4085 | 1944.8 |
| 129 | 271 | 3081 | 5455.4 | 1766.3 | 5137.8 | 488.7 | 1208.9 | 648.6 | 4324.6 | 3878.2 | 1687.3 |
| 243 | 374 | 5384 | 11253.5 | 2893 | 9262.8 | 706.3 | 2814.2 | 1529.7 | 5724.5 | 7302.1 | 1881.7 |
| 156 | 444 | 3934 | 6517.1 | 2201 | 8889 | 588 | 1461.5 | 800.9 | 6921.8 | 5040.5 | 1890.6 |
| 168 | 632 | 4689 | 6743.2 | 2415.8 | 13080.6 | 704.1 | 1618.2 | 712.6 | 10789.9 | 5284.3 | 1906.8 |
| 103 | 198 | 2546 | 5227.1 | 1744.3 | 4241.4 | 455.9 | 1117.5 | 566.9 | 3868.8 | 3490.8 | 1853.9 |
| 94 | 159 | 2424 | 5859.3 | 1449.6 | 3638.7 | 297.7 | 1558.7 | 700.1 | 3744.1 | 3657.2 | 1886 |

| | | | | | | | | | | | |
|---|---|---|---|---|---|---|---|---|---|---|---|
| 83 | 133 | 1791 | 3567.9 | 1055.1 | 3034.2 | 323.6 | 892.1 | 524.9 | 2282.6 | 3380.3 | 2040.8 |
| 106 | 169 | 2400 | 5533.3 | 1654.9 | 3895.5 | 422 | 1215.4 | 670.8 | 3570.1 | 3690.5 | 1976.1 |
| 144 | 273 | 3247 | 6374.7 | 2250.9 | 5102 | 522 | 1421.5 | 813 | 5064.7 | 5078 | 1931.1 |
| 117 | 225 | 2672 | 5451.3 | 1505.7 | 4323.4 | 396.5 | 1404.3 | 558.2 | 3912.9 | 4377 | 1913 |
| 176 | 321 | 3961 | 8277.4 | 2624.6 | 7253.4 | 765.6 | 1632.3 | 937.5 | 4879.7 | 6389.8 | 2025.2 |
| 102 | 194 | 2390 | 4608.9 | 1786.2 | 3640.9 | 531.1 | 804.4 | 466.8 | 3340 | 3629.9 | 1875.2 |
| 84 | 130 | 2070 | 5640.6 | 1537.4 | 3144.6 | 333.2 | 1336.7 | 596.3 | 3427.1 | 3274.8 | 2057.3 |
| 132 | 216 | 2976 | 6578 | 1771.6 | 4397.7 | 406.4 | 1445.8 | 620.4 | 3915.2 | 4095.7 | 1802.8 |
| 118 | 270 | 2862 | 5205.3 | 1786.9 | 5607.4 | 478.3 | 1185.1 | 649.4 | 4658.1 | 4206.3 | 1890.5 |
| 171 | 425 | 4502 | 7785.8 | 2519.2 | 8038.1 | 528.2 | 1978.7 | 1132.8 | 6972.1 | 6007.6 | 1745.1 |
| 103 | 268 | 2804 | 5496.9 | 1693.8 | 5944.8 | 409 | 1566.7 | 728.1 | 5392.7 | 3965.5 | 1943.5 |
| 126 | 284 | 3064 | 5431.2 | 1954.8 | 5545.5 | 652.1 | 1026.7 | 607.1 | 4565.2 | 4297.5 | 1845.5 |
| 108 | 271 | 2868 | 5172.5 | 1608.3 | 5776.6 | 409.9 | 1277.4 | 637.8 | 5140.9 | 3862.3 | 1823.7 |
| 148 | 287 | 3323 | 6390.6 | 1988.1 | 5761.4 | 696.8 | 1307.6 | 597.1 | 4543.5 | 4666 | 1871.9 |
| 156 | 349 | 3740 | 6708.9 | 2310.5 | 7280.8 | 724.1 | 1238.9 | 707.8 | 5573.2 | 5251.2 | 1891.2 |
| 116 | 270 | 2966 | 5509.7 | 1988.1 | 5587.5 | 569.1 | 1012.7 | 598.6 | 4341.3 | 4232.6 | 1884.4 |
| 116 | 240 | 2826 | 5669.8 | 1581.4 | 5124.4 | 387.6 | 1441.3 | 744.1 | 4436.7 | 4289.6 | 1878.4 |
| 178 | 394 | 4157 | 7771.1 | 2359.4 | 7704.1 | 533.5 | 1926.9 | 1123.4 | 6039.2 | 5971.3 | 1898.4 |
| 100 | 282 | 2663 | 4609.6 | 1499.2 | 6053.4 | 401.6 | 1084.4 | 609.9 | 5273.7 | 3896 | 1938.5 |
| 132 | 267 | 3363 | 6774.7 | 1884.2 | 5855.3 | 432.5 | 1674.7 | 904.2 | 4604.8 | 4658.6 | 1811.8 |
| 132 | 339 | 3347 | 6080 | 1859.3 | 7278.3 | 456.9 | 1517.3 | 859.7 | 6524.4 | 4652.2 | 1939.6 |
| 106 | 305 | 2863 | 4910.7 | 1680.9 | 6737 | 423.3 | 1261.2 | 626.7 | 6005.8 | 3565.3 | 1900.6 |
| 144 | 326 | 3505 | 6539.6 | 2169.1 | 6853.1 | 642.9 | 1339.3 | 754.1 | 5199 | 5196.7 | 1935 |
| 213 | 464 | 4928 | 9013.2 | 2957.7 | 9118 | 616.6 | 2057.4 | 1173.4 | 6116.1 | 6255.4 | 1850.3 |
| 96 | 186 | 2305 | 4562.3 | 1654.1 | 3776.5 | 462.5 | 866.1 | 512.3 | 3347.4 | 3410.2 | 1879.9 |
| 148 | 337 | 3704 | 6404.7 | 2323.8 | 6442.7 | 703.9 | 1190.3 | 725.5 | 4821.9 | 4695 | 1742.9 |
| 246 | 502 | 5656 | 9840 | 2510.3 | 10049.4 | 591.4 | 2541.7 | 1339.8 | 7796.9 | 7847 | 1778.7 |
| 348 | 574 | 7996 | 18072.8 | 3249.1 | 12568.4 | 496.5 | 7581.9 | 3324.3 | 10605.1 | 10780.9 | 1905.2 |
| 140 | 244 | 3249 | 7031.2 | 2320.7 | 5397.8 | 598.7 | 1411.4 | 815.2 | 4365.9 | 5071.8 | 1982.5 |
| 228 | 428 | 4701 | 8719.4 | 2587 | 8530.1 | 605.4 | 2108.6 | 1229.1 | 6509.7 | 6577 | 1870.9 |
| 152 | 229 | 3339 | 7498.5 | 2002.9 | 4656.1 | 442.1 | 1820.9 | 791.6 | 4314.9 | 4808.7 | 1828 |
| 126 | 191 | 2512 | 5460.2 | 1535 | 3596.6 | 419.7 | 1183.2 | 482 | 2844.4 | 3714.3 | 1877.7 |
| 223 | 459 | 4877 | 7950.1 | 2743.6 | 7735.1 | 597.3 | 2087 | 1082.9 | 6445.1 | 5710.7 | 1658.1 |
| 205 | 441 | 4686 | 8973.7 | 2865 | 9017.1 | 620.7 | 2042.3 | 1149.7 | 6597.7 | 6342.7 | 1928.3 |
| 117 | 244 | 2753 | 4816.9 | 1589.4 | 4537.3 | 414.9 | 1063.3 | 576 | 4298.6 | 3714.1 | 1703.1 |
| 137 | 361 | 3498 | 6218.8 | 1930.7 | 7664.3 | 457.4 | 1559 | 864.1 | 6884.4 | 4833.8 | 1911 |
| 1158 | 233 | 5421 | 5081.7 | 1528.8 | 3057.7 | 349.6 | 1024.2 | 505.5 | 2940.2 | 3058.7 | 1854.9 |
| 146 | 474 | 3818 | 5742.2 | 2068 | 9449.5 | 586.7 | 1217.6 | 617.6 | 7340.8 | 4740.9 | 1869.6 |
| 160 | 339 | 3729 | 6941.9 | 2073.8 | 7218.5 | 483.5 | 1726.9 | 955.3 | 6194.8 | 5401.1 | 1896.3 |
| 115 | 317 | 3090 | 5676.7 | 1911.8 | 6599.7 | 500.1 | 1224.9 | 670.4 | 5480.1 | 4115 | 1890.7 |
| 97 | 148 | 2330 | 5834.4 | 1784.8 | 3165 | 382.1 | 1171 | 557.5 | 3659.3 | 3295.3 | 1884.6 |
| 112 | 229 | 2734 | 5550.2 | 1758.2 | 4925.1 | 411.6 | 1372 | 750.2 | 4851.2 | 4609.4 | 2023.3 |
| 160 | 285 | 3757 | 8416.9 | 1848.6 | 6018.7 | 293.1 | 3193.1 | 1417 | 7065.8 | 5411.7 | 1935.4 |
| 71 | 163 | 1986 | 4763.8 | 1735.4 | 4140.9 | 408.9 | 996.3 | 472.7 | 3810.4 | 3092.1 | 2155.5 |
| 170 | 384 | 4206 | 9347.8 | 2822.5 | 10242 | 672.6 | 2245 | 1330.4 | 7760.3 | 7053.8 | 2237.1 |
| 206 | 291 | 4141 | 9198.2 | 1918.8 | 5249.7 | 457.1 | 2293.4 | 633.7 | 5081 | 6199.2 | 1835.2 |
| 74 | 121 | 1901 | 5301.8 | 1765.9 | 2948.3 | 389.7 | 1015.6 | 430.6 | 3648 | 2765.4 | 2045.6 |
| 83 | 182 | 2136 | 3060.9 | 939.1 | 2834.3 | 224.7 | 848.6 | 421.1 | 3085.1 | 2254.5 | 1372 |
| 227 | 404 | 5200 | 10807.8 | 2644.5 | 7998.4 | 393.3 | 3539.5 | 2057.8 | 6905.6 | 7367.4 | 1840.9 |
| 355 | 577 | 7027 | 13606.9 | 2608.7 | 11227.3 | 443.4 | 5625.8 | 2305.8 | 8321.6 | 10129.8 | 1824.3 |
| 129 | 200 | 2818 | 6646.7 | 1691.7 | 4421.1 | 429.6 | 1369.4 | 490 | 4324 | 4288.8 | 1917.2 |
| 89 | 153 | 2108 | 4663.3 | 1366.2 | 3292.1 | 332.5 | 1013.6 | 545.9 | 3111.3 | 3232.6 | 1880.9 |
| 122 | 219 | 2565 | 5340.4 | 1565.6 | 4373.1 | 388.4 | 1379 | 725.7 | 3399.9 | 3594.6 | 1923.3 |
| 154 | 314 | 3941 | 7066.4 | 2302.3 | 6656.5 | 579.3 | 1466.4 | 878.2 | 5071.7 | 5263 | 1728.7 |
| 130 | 207 | 2873 | 6293.2 | 1748.9 | 4657.2 | 368.7 | 1629.9 | 851.5 | 4169.2 | 4302.7 | 1897.8 |
| 84 | 134 | 1949 | 4819.6 | 1358.1 | 3024.4 | 308 | 1092.7 | 496.8 | 3134.2 | 3084.6 | 1977.1 |

| | | | | | | | | | | | |
|---|---|---|---|---|---|---|---|---|---|---|---|
| 102 | 157 | 2353 | 5803.3 | 1714.1 | 3259.1 | 374.3 | 1214.6 | 504.1 | 3459.5 | 3047.4 | 1853.9 |
| 137 | 374 | 3606 | 6432.5 | 2288 | 7632.4 | 636.4 | 1386.8 | 822.7 | 6436.6 | 4879.4 | 1913.3 |
| 129 | 244 | 2960 | 5827.5 | 1862.5 | 5212.2 | 592.7 | 1145.3 | 608 | 3904 | 4142.8 | 1877.9 |
| 133 | 213 | 2980 | 6476.3 | 1565 | 4481.2 | 515.1 | 1507.8 | 629.8 | 3109.3 | 4278.5 | 1830 |
| 80 | 125 | 1928 | 4697 | 1461.9 | 2682 | 331.3 | 972.2 | 458.4 | 2898.8 | 2844.3 | 1904.2 |
| 227 | 499 | 4972 | 8875.7 | 2894.6 | 9691.1 | 690.8 | 2014.7 | 1048.9 | 7649.4 | 6840.7 | 1910.7 |
| 115 | 192 | 2426 | 5148.1 | 1520.5 | 3719.3 | 316.9 | 1393.5 | 678.6 | 3247 | 3091.5 | 1820 |
| 125 | 233 | 2710 | 5453.1 | 1752.5 | 5162.5 | 422.5 | 1348.1 | 656.3 | 4704.2 | 3922.2 | 1989.9 |
| 82 | 147 | 2135 | 5306.1 | 1558.6 | 3442.5 | 321 | 1351.6 | 626.7 | 3699.1 | 3139.6 | 1994.1 |
| 135 | 227 | 2797 | 5472.1 | 1578.7 | 4618 | 363.2 | 1441 | 692.7 | 3873.1 | 4050.2 | 1834.7 |
| 142 | 251 | 3278 | 6618.6 | 1909.1 | 5056.2 | 357.6 | 2108.3 | 1043.8 | 5748.8 | 4341.3 | 1797.4 |
| 240 | 494 | 5183 | 8854.4 | 1858.3 | 10039.1 | 472.1 | 7222.8 | 769.5 | 8790.1 | 5781.8 | 1864.3 |
| 145 | 258 | 3376 | 7193 | 1996.5 | 5311.1 | 494.1 | 1569.9 | 778.8 | 4194.8 | 5219.8 | 1856.8 |
| 95 | 175 | 2127 | 4280.2 | 1366.8 | 3532.1 | 376.2 | 944.1 | 493.4 | 2853.7 | 2911.9 | 1835 |
| 106 | 233 | 2614 | 4976.6 | 1489.4 | 4801.5 | 416.3 | 1224.6 | 734.4 | 4143.8 | 4018.1 | 1913.5 |
| 175 | 326 | 3982 | 7901.2 | 1815.6 | 6969 | 364.7 | 2655.6 | 1199.9 | 5756.2 | 5828.1 | 1851.1 |
| 165 | 476 | 4151 | 6514.9 | 2266.5 | 9362.7 | 725.7 | 1454.8 | 834.3 | 7590.6 | 5213 | 1845.2 |
| 113 | 158 | 2460 | 6612.6 | 1803.2 | 3579.9 | 390.1 | 1403.1 | 628.6 | 3414.6 | 3988.4 | 2030.3 |
| 159 | 294 | 3521 | 949 | 171 | 805 | 46 | 281 | 98 | 596 | 740 | 252 |
| 118 | 173 | 2137 | 213.9 | 58.4 | 169.9 | 21 | 53.5 | 22.6 | 120.8 | 169.8 | 99.3 |
| 133 | 286 | 3152 | 765 | 196 | 812 | 52 | 211 | 111 | 565 | 581 | 242 |

| D Ala | L Val | D Val | L Phe | L Ile | D Phe | L Leu | D alle | D Leu | UAL_num | UAL_num | **Valve** |
|---|---|---|---|---|---|---|---|---|---|---|---|
| 319.75 | 699.7 | 58.4 | 388.6 | 384.05 | 62.95 | 499.65 | 31.35 | 98.85 | 11365 | 11365 | right |
| 39.9 | 195.2 | 5.5 | 135.3 | 107.8 | 7.7 | 299.8 | 3.9 | 24.2 | 11366 | 11366 | left |
| 54.1 | 193.4 | 9.2 | 125.8 | 103.9 | 11.4 | 163.5 | 8.2 | 19 | 11367 | 11367 | left |
| 64.6 | 353 | 7.9 | 218.2 | 194.4 | 9.5 | 275.1 | 6.3 | 25.9 | 11368 | 11368 | left |
| 59 | 174.6 | 9.4 | 111.9 | 92.5 | 14 | 130.3 | 6.6 | 20.8 | 11369 | 11369 | left |
| 61.8 | 275.9 | 11.8 | 201.3 | 182.5 | 11.9 | 300.1 | 8.2 | 19.7 | 11370 | 11370 | left |
| 64 | 343 | 8 | 201 | 182 | 11 | 251 | 9 | 28 | 11371 | 11371 | left |
| 60.8 | 362.2 | 6.9 | 242.5 | 206.6 | 9.8 | 332 | 8.8 | 19 | 11372 | 11372 | left |
| 49.8 | 180.8 | 7.7 | 113.7 | 93.7 | 9.3 | 145.8 | 6.3 | 19 | 11373 | 11373 | left |
| 57.6 | 220.7 | 9.8 | 146.4 | 122.2 | 11.6 | 208.7 | 8.8 | 18.1 | 11374 | 11374 | left |
| 87.2 | 309.8 | 13.5 | 182.2 | 160.7 | 14.3 | 196.5 | 10.9 | 13 | 11375 | 11375 | left |
| 83.3 | 309.3 | 12.1 | 185.6 | 167.9 | 13.9 | 228.1 | 10.7 | 20.4 | 11376 | 11376 | left |
| 67 | 199.8 | 13.6 | 125.4 | 107.2 | 14.5 | 150 | 8.8 | 17.6 | 11377 | 11377 | left |
| 64.9 | 206.5 | 11.5 | 124.2 | 106.8 | 11.8 | 162.4 | 7.3 | 18.6 | 11378 | 11378 | left |
| 78.2 | 182.2 | 13.4 | 120 | 99.4 | 15.7 | 154.4 | 8.6 | 26.4 | 11379 | 11379 | left |
| 55 | 271 | 10 | 172 | 144 | 12 | 211 | 8 | 23 | 11380 | 11380 | left |
| 72 | 298 | 14 | 170 | 160 | 14 | 239 | 10 | 25 | 11381 | 11381 | left |
| 55 | 562 | 7 | 359 | 317 | 13 | 496 | 5 | 28 | 11382 | 11382 | left |
| 62 | 363 | 15 | 243 | 201 | 11 | 335 | 6 | 17 | 11383 | 11383 | right |
| 62.9 | 263.2 | 7.2 | 190.7 | 153 | 11.7 | 207.6 | 6.2 | 19.9 | 11384 | 11384 | left |
| 82.1 | 140.1 | 16.2 | 87 | 77.8 | 16.4 | 126.1 | 11.9 | 29.6 | 11385 | 11385 | left |
| 40.8 | 307.5 | 6.9 | 219.7 | 159.4 | 8.5 | 291.6 | 7.4 | 13.9 | 11386 | 11386 | left |
| 54.5 | 353.8 | 8.5 | 267.6 | 199.7 | 11.2 | 292.3 | 8.9 | 14.9 | 11387 | 11387 | left |
| 92 | 857 | 10 | 518 | 474 | 12 | 687 | 23 | 68 | 11388 | 11388 | left |
| 63 | 569 | 12 | 336 | 315 | 12 | 506 | 10 | 44 | 11389 | 11389 | left |
| 785 | 8697 | 109 | 5004 | 4705 | 252 | 6458 | 363 | 283 | 11390 | 11390 | left |
| 89 | 336 | 14 | 194 | 183 | 14 | 277 | 11 | 25 | 11391 | 11391 | left |
| 137 | 1442 | 20 | 925 | 852 | 16 | 1357 | 19 | 45 | 11392 | 11392 | left |
| 45 | 588 | 9 | 390 | 298 | 13 | 499 | 7 | 24 | 11393 | 11393 | left |
| 119 | 417 | 21 | 212 | 209 | 19 | 258 | 13 | 31 | 11394 | 11394 | left |
| 412 | 1607 | 85 | 978 | 918 | 111 | 1235 | 36 | 125 | 11395 | 11395 | right |
| 261 | 3001 | 59 | 2081 | 1552 | 103 | 2276 | 93 | 94 | 11396 | 11396 | right |
| 76.4 | 441.8 | 7.1 | 243.2 | 206.6 | 9.9 | 278.7 | 9.1 | 19.4 | 11397 | 11397 | right |
| 37 | 349 | 7 | 247 | 188 | 10 | 394 | 6 | 16 | 11398 | 11398 | right |
| 78 | 269 | 13 | 152 | 135 | 16 | 215 | 11 | 22 | 11399 | 11399 | right |
| 43 | 244 | 9 | 156 | 136 | 10 | 223 | 7 | 14 | 11400 | 11400 | right |
| 39 | 201 | 7 | 162 | 110 | 11 | 210 | 5 | 10 | 11401 | 11401 | right |
| 68.3 | 292 | 12.7 | 190.4 | 152.2 | 14.8 | 225.8 | 11.3 | 22.9 | 11402 | 11402 | right |
| 80.6 | 251 | 14.1 | 151 | 130.2 | 15.1 | 202.9 | 9 | 22.3 | 11403 | 11403 | right |
| 76 | 330 | 12 | 192 | 160 | 14 | 274 | 9 | 23 | 11404 | 11404 | right |
| 69.8 | 536.7 | 12.5 | 320.3 | 254.9 | 15.3 | 366.8 | 8.5 | 25.9 | 11405 | 11405 | right |
| 95 | 247 | 19 | 138 | 130 | 17 | 185 | 13 | 24 | 11406 | 11406 | right |
| 185 | 685 | 31 | 387 | 346 | 31 | 489 | 20 | 35 | 11407 | 11407 | right |
| 52 | 567 | 9 | 413 | 325 | 14 | 545 | 13 | 27 | 11408 | 11408 | right |
| 69.9 | 396.1 | 12.5 | 221.1 | 184.8 | 12.9 | 244.9 | 12.2 | 31.9 | 11409 | 11409 | right |
| 120.7 | 743.4 | 11.7 | 325.6 | 275.9 | 11.2 | 469.4 | 5.8 | 23.3 | 11410 | 11410 | right |
| 49 | 299 | 11 | 173 | 150 | 9 | 246 | 8 | 22 | 11411 | 11411 | right |
| 88 | 359 | 13 | 214 | 184 | 14 | 280 | 11 | 21 | 11412 | 11412 | right |
| 103 | 336 | 17 | 179 | 154 | 16 | 225 | 12 | 29 | 11413 | 11413 | right |
| 44 | 248 | 8 | 142 | 119 | 9 | 197 | 5 | 16 | 11414 | 11414 | right |

| | | | | | | | | | | | |
|---|---|---|---|---|---|---|---|---|---|---|---|
| 65.4 | 256.9 | 12.8 | 153.6 | 128.8 | 12.2 | 178.1 | 9.9 | 33.5 | 11415 | 11415 | right |
| 38 | 344 | 7 | 230 | 182 | 9 | 306 | 5 | 24 | 11416 | 11416 | right |
| 95.6 | 445.2 | 16.6 | 242 | 224.2 | 15.7 | 299.7 | 10.3 | 30.4 | 11417 | 11417 | right |
| 66.6 | 460.7 | 12.5 | 257.4 | 221.8 | 12.3 | 282.4 | 7.2 | 21.3 | 11418 | 11418 | right |
| 81 | 288 | 15 | 177 | 160 | 14 | 205 | 8 | 28 | 11419 | 11419 | right |
| 44.9 | 278.3 | 8.4 | 181.1 | 147.9 | 10.4 | 222.8 | 4.6 | 27.8 | 11420 | 11420 | right |
| 41 | 258.2 | 5.7 | 166.1 | 131.5 | 8 | 287.1 | 6.4 | 15.2 | 11421 | 11421 | right |
| 44 | 379 | 10 | 248 | 187 | 13 | 306 | 6 | 36 | 11422 | 11422 | right |
| 90.2 | 535.5 | 15.5 | 315.1 | 300.2 | 14.9 | 370.8 | 10.9 | 36.4 | 11423 | 11423 | right |
| 65.2 | 292.2 | 11.6 | 168.1 | 147.2 | 13.4 | 191.5 | 6.6 | 24.1 | 11424 | 11424 | right |
| 356 | 1052 | 57 | 688 | 593 | 87 | 1034 | 41 | 101 | 11425 | 11425 | left |
| 655 | 1765 | 122 | 932 | 790 | 142 | 1340 | 62 | 163 | 11427 | 11427 | left |
| 402 | 1415 | 66 | 790 | 698 | 106 | 1084 | 36 | 109 | 11428 | 11428 | left |
| 665 | 1579 | 117 | 984 | 975 | 167 | 1566 | 75 | 214 | 11429 | 11429 | left |
| 349 | 1433 | 65 | 822 | 727 | 100 | 1085 | 31 | 94 | 11430 | 11430 | left |
| 96.4 | 377.6 | 18.4 | 249.4 | 195.1 | 26.6 | 426 | 11.2 | 27.8 | 11431 | 11431 | left |
| 115.1 | 363.3 | 22.1 | 245.3 | 184.7 | 31.3 | 397.7 | 13.7 | 31.9 | 11432 | 11432 | left |
| 123.8 | 452.1 | 22.2 | 302.1 | 222.8 | 35.3 | 449.8 | 14.2 | 34.9 | 11433 | 11433 | left |
| 444 | 1089 | 70 | 691 | 593 | 120 | 1000 | 45 | 125 | 11434 | 11434 | left |
| 399 | 1479 | 76 | 861 | 698 | 105 | 1014 | 40 | 111 | 11435 | 11435 | left |
| 468 | 1624 | 78 | 1044 | 800 | 134 | 1232 | 42 | 120 | 11436 | 11436 | left |
| 354 | 998 | 63 | 520 | 422 | 79 | 897 | 36 | 91 | 11437 | 11437 | left |
| 395 | 1195 | 82 | 816 | 679 | 117 | 1293 | 36 | 114 | 11438 | 11438 | left |
| 609 | 1359 | 116 | 885 | 770 | 168 | 2080 | 82 | 212 | 11439 | 11439 | left |
| 54 | 342 | 12 | 216 | 183 | 14 | 331 | 8 | 15 | 11440 | 11440 | left |
| 112.8 | 308.2 | 20.7 | 182.9 | 159.1 | 23.9 | 303 | 12.1 | 26.7 | 11441 | 11441 | left |
| 116 | 227 | 26 | 132 | 117 | 25 | 240 | 14 | 35 | 11442 | 11442 | left |
| 358 | 956 | 64 | 520 | 453 | 83 | 726 | 30 | 99 | 11443 | 11443 | left |
| 115.5 | 564.7 | 15.6 | 304.5 | 279.8 | 16.5 | 381.8 | 11.7 | 37 | 11444 | 11444 | left |
| 98 | 368.8 | 14.4 | 265.4 | 194.9 | 25.9 | 393.1 | 11.2 | 23.9 | 11445 | 11445 | left |
| 50.6 | 576 | 7.8 | 513.5 | 316.4 | 21.1 | 567.4 | 5.8 | 19.4 | 11446 | 11446 | left |
| 88.3 | 203.7 | 15.8 | 122.1 | 108.9 | 17.4 | 190.7 | 9.2 | 21.6 | 11447 | 11447 | left |
| 82 | 219 | 16 | 125 | 107 | 16 | 183 | 11 | 22 | 11448 | 11448 | left |
| 85 | 214 | 17 | 121 | 103 | 17 | 146 | 11 | 22 | 11449 | 11449 | left |
| 94.4 | 317.8 | 15.3 | 216.1 | 168.2 | 23 | 280.8 | 10.2 | 25.2 | 11450 | 11450 | left |
| 56 | 225 | 8 | 151 | 124 | 11 | 201 | 9 | 18 | 11451 | 11451 | left |
| 62 | 184 | 14 | 122 | 106 | 13 | 152 | 7 | 10 | 11452 | 11452 | left |
| 85 | 213 | 15 | 133 | 119 | 19 | 188 | 10 | 19 | 11453 | 11453 | left |
| 66.6 | 295.7 | 12.3 | 188.2 | 158 | 14.4 | 237.7 | 10 | 19.3 | 11454 | 11454 | left |
| 485 | 1368 | 104 | 709 | 611 | 118 | 924 | 46 | 119 | 11455 | 11455 | left |
| 105.5 | 217.2 | 19.7 | 131.4 | 109.9 | 21.7 | 224.4 | 12.5 | 28 | 11456 | 11456 | left |
| 198.5 | 544.4 | 37.2 | 373.6 | 287.3 | 55.8 | 607.9 | 25.1 | 58 | 11457 | 11457 | left |
| 120.3 | 375.9 | 23.9 | 250.5 | 197.1 | 32.4 | 343.4 | 14.1 | 28.8 | 11458 | 11458 | left |
| 125.3 | 291.6 | 23.6 | 190.3 | 159.5 | 28.8 | 222.9 | 14.9 | 30.7 | 11459 | 11459 | left |
| 93.1 | 244.7 | 18 | 153.2 | 135.5 | 19.3 | 223.6 | 11.5 | 26.9 | 11460 | 11460 | left |
| 110 | 327 | 19 | 183 | 176 | 22 | 350 | 13 | 30 | 11461 | 11461 | left |
| 564 | 1109 | 97 | 673 | 596 | 129 | 1137 | 59 | 149 | 11462 | 11462 | left |
| 123.9 | 300.5 | 23.4 | 194 | 166.3 | 27 | 325.7 | 15.1 | 34.9 | 11463 | 11463 | left |
| 109.7 | 340.1 | 18.7 | 238.7 | 179.9 | 29 | 303.6 | 12.5 | 26.9 | 11464 | 11464 | left |
| 91 | 155.4 | 19 | 86.3 | 72.5 | 17.5 | 114.2 | 11 | 24.4 | 11465 | 11465 | left |
| 112.1 | 416.4 | 21.1 | 272 | 219.8 | 28.5 | 617.5 | 12.4 | 36.1 | 11466 | 11466 | left |
| 109.2 | 275.4 | 23.4 | 169.4 | 148.1 | 25.2 | 296.5 | 13.4 | 39.8 | 11467 | 11467 | left |
| 150.1 | 438 | 25.5 | 287.8 | 251.9 | 34.9 | 570.3 | 19 | 41.3 | 11468 | 11468 | left |
| 129 | 367 | 26 | 217 | 183 | 30 | 293 | 15 | 33 | 11469 | 11469 | left |
| 127 | 235 | 28 | 126 | 110 | 28 | 223 | 16 | 38 | 11470 | 11470 | left |
| 79 | 374 | 13 | 303 | 223 | 25 | 382 | 11 | 16 | 11471 | 11471 | left |

| | | | | | | | | | | | |
|---|---|---|---|---|---|---|---|---|---|---|---|
| 110.6 | 399.4 | 20.1 | 267.4 | 202.5 | 33.9 | 371.7 | 12.9 | 27.8 | 11472 | 11472 | left |
| 86.9 | 206.9 | 14.3 | 129.3 | 112.7 | 17.2 | 176.8 | 9.6 | 28.4 | 11473 | 11473 | left |
| 70 | 205 | 13 | 127 | 100 | 16 | 110 | 8 | 24 | 11474 | 11474 | left |
| 596 | 1572 | 114 | 863 | 724 | 127 | 1027 | 61 | 153 | 11475 | 11475 | left |
| 134.1 | 351 | 30.9 | 193.5 | 162.5 | 32.4 | 286.4 | 17.2 | 38.2 | 11476 | 11476 | left |
| 82 | 243 | 14 | 163 | 122 | 19 | 278 | 10 | 24 | 11477 | 11477 | left |
| 133.5 | 424.9 | 23.6 | 274 | 222.6 | 30.3 | 450 | 14.6 | 34.5 | 11478 | 11478 | left |
| 475.3 | 1283 | 92.75 | 603.2 | 531 | 93.75 | 946.2 | 34.65 | 123.2 | 11479 | 11479 | left |
| 109 | 231 | 20 | 145 | 128 | 22 | 257 | 13 | 27 | 11480 | 11480 | left |
| 111 | 291 | 25 | 167 | 151 | 28 | 296 | 16 | 35 | 11481 | 11481 | left |
| 90.8 | 195.8 | 18.4 | 123.8 | 104.1 | 19.5 | 209.6 | 11.3 | 26.4 | 11482 | 11482 | left |
| 103 | 281.4 | 20.8 | 177.2 | 148.9 | 24.3 | 323.1 | 12.9 | 29.4 | 11483 | 11483 | left |
| 90.7 | 404.7 | 14.3 | 285.5 | 216.7 | 23.5 | 370.7 | 9.9 | 26.1 | 11484 | 11484 | left |
| 489 | 1923 | 89 | 1308 | 979 | 166 | 1379 | 63 | 130 | 11485 | 11485 | left |
| 46 | 135 | 9 | 77 | 65 | 9 | 101 | 4 | 11 | 11486 | 11486 | left |
| 123 | 250 | 27 | 142 | 119 | 27 | 221 | 16 | 33 | 11487 | 11487 | right |
| 104 | 309 | 20 | 200 | 158 | 25 | 311 | 11 | 29 | 11488 | 11488 | right |
| 447 | 1740 | 78 | 984 | 789 | 113 | 1446 | 35 | 117 | 11489 | 11489 | left |
| 95 | 247 | 17 | 158 | 130 | 22 | 276 | 11 | 23 | 11490 | 11490 | left |
| 105 | 254 | 20 | 153 | 135 | 23 | 242 | 13 | 28 | 11491 | 11491 | left |
| 108 | 290 | 19 | 186 | 148 | 26 | 302 | 13 | 27 | 11492 | 11492 | right |
| 106.7 | 297.7 | 18.3 | 186 | 153.2 | 22.9 | 271.5 | 12.2 | 31.2 | 11493 | 11493 | right |
| 96 | 212 | 18 | 136 | 113 | 22 | 232 | 12 | 28 | 11494 | 11494 | left |
| 103.8 | 266.6 | 20 | 153.2 | 123.7 | 22.1 | 247.3 | 11.8 | 29.1 | 11495 | 11495 | left |
| 73.3 | 187.4 | 13.8 | 117.7 | 97.7 | 15.1 | 180.3 | 8.6 | 22.8 | 11496 | 11496 | left |
| 100.8 | 293.6 | 17.9 | 184.2 | 152.8 | 22.7 | 296.8 | 11.2 | 26.7 | 11497 | 11497 | left |
| 114 | 296 | 26 | 186 | 150 | 32 | 360 | 15 | 36 | 11498 | 11498 | left |
| 120.6 | 290.9 | 25.6 | 173.4 | 139.2 | 31.2 | 379 | 14.6 | 39.1 | 11499 | 11499 | left |
| 103 | 346 | 19 | 231 | 179 | 27 | 322 | 13 | 27 | 11500 | 11500 | left |
| 87 | 313 | 16 | 217 | 168 | 24 | 361 | 12 | 24 | 11501 | 11501 | left |
| 138.6 | 268.2 | 28.2 | 146.7 | 130.7 | 27.7 | 259.6 | 17.8 | 42.2 | 11502 | 11502 | left |
| 94 | 182 | 21 | 120 | 103 | 24 | 335 | 15 | 28 | 11503 | 11503 | right |
| 108.5 | 235.6 | 21.7 | 139.4 | 117.2 | 24.7 | 318.2 | 13.7 | 32.7 | 11504 | 11504 | right |
| 641 | 1574 | 125 | 898 | 766 | 158 | 1352 | 71 | 164 | 11505 | 11505 | right |
| 135.2 | 269.9 | 28.5 | 148.5 | 131.9 | 27.1 | 266.6 | 17.4 | 39.3 | 11506 | 11506 | right |
| 128.7 | 332.3 | 24 | 187.9 | 161.6 | 25 | 349.8 | 13.7 | 32.8 | 11507 | 11507 | right |
| 99 | 203 | 20 | 119 | 99 | 20 | 174 | 12 | 25 | 11508 | 11508 | left |
| 81 | 234 | 15 | 153 | 125 | 19 | 243 | 9 | 20 | 11509 | 11509 | right |
| 108.6 | 202.9 | 22.6 | 114.9 | 104.3 | 24 | 270.6 | 15.3 | 37.5 | 11510 | 11510 | left |
| 133 | 319.8 | 24.1 | 187.9 | 166.6 | 27.3 | 367.7 | 16.1 | 34.2 | 11511 | 11511 | right |
| 100.2 | 317.3 | 16.6 | 206.4 | 169.4 | 23.8 | 359.6 | 11.3 | 29.1 | 11512 | 11512 | left |
| 93.1 | 489.6 | 16.1 | 339.8 | 265.5 | 26.8 | 488.7 | 15.2 | 33.1 | 11513 | 11513 | right |
| 80.4 | 156.7 | 17.5 | 102.5 | 83.3 | 21.7 | 135.9 | 11.8 | 23 | 11514 | 11514 | left |
| 105.8 | 330.6 | 18.1 | 191.9 | 156.1 | 23.3 | 249.2 | 12.6 | 29.3 | 11515 | 11515 | right |
| 108.9 | 247 | 20.6 | 147.1 | 124.5 | 21.9 | 207.3 | 12.3 | 27.5 | 11516 | 11516 | right |
| 111.8 | 248.3 | 20.6 | 156.1 | 141.7 | 25.4 | 281.7 | 14.4 | 28.2 | 11517 | 11517 | left |
| 125 | 337 | 26 | 231 | 176 | 40 | 405 | 15 | no data | 11518 | 11518 | left |
| 122.4 | 253.4 | 24.2 | 151.2 | 134 | 28.3 | 228.2 | 16.8 | 35.3 | 11519 | 11519 | left |
| 74.2 | 160 | 14.5 | 96.5 | 82.2 | 16.1 | 137.6 | 10.4 | 22.5 | 11520 | 11520 | right |
| 108.4 | 398.9 | 17.9 | 273.1 | 247.6 | 27.9 | 382.8 | 12.7 | 28.3 | 11521 | 11521 | left |
| 176.4 | 484.4 | 31.8 | 301.4 | 273.2 | 44.8 | 553 | 23.2 | 43.9 | 11522 | 11522 | left |
| 145.2 | 312.5 | 28.9 | 174.2 | 174.5 | 29.5 | 332.5 | 20.8 | 40 | 11523 | 11523 | left |
| 55.1 | 143.8 | 7.7 | 87.5 | 74.3 | 10.2 | 115.2 | 7.6 | 17.1 | 11524 | 11524 | left |
| 123.6 | 335.6 | 26.3 | 202.6 | 161.7 | 32.5 | 346.9 | 16.3 | 33.2 | 11525 | 11525 | left |
| 158.7 | 335.9 | 31.4 | 183.8 | 185.1 | 30.4 | 354.2 | 22.1 | 45.6 | 11526 | 11526 | left |
| 125.7 | 302 | 23.2 | 192.8 | 159.6 | 30.7 | 331.8 | 15.4 | 34.4 | 11527 | 11527 | left |

| | | | | | | | | | | | |
|---|---|---|---|---|---|---|---|---|---|---|---|
| 103.1 | 438.8 | 18.5 | 312.9 | 248.2 | 29 | 487.9 | 11.2 | 29.2 | 11528 | 11528 | left |
| 76 | 145 | 17 | 84 | 72 | 18 | 237 | 12 | 27 | 11529 | 11529 | right |
| 81 | 254 | 16 | 169 | 135 | 22 | 260 | 11 | 27 | 11530 | 11530 | right |
| 80 | 140 | 16 | 86 | 73 | 18 | 125 | 11 | 23 | 11531 | 11531 | right |
| 60 | 144 | 11 | 99 | 81 | 15 | 173 | 8 | 21 | 11532 | 11532 | right |
| 96 | 183 | 19 | 115 | 103 | 23 | 196 | 14 | 29 | 11534 | 11534 | right |
| 81 | 291 | 16 | 202 | 159 | 25 | 395 | 12 | 25 | 11535 | 11535 | right |
| 136 | 351 | 30 | 232 | 183 | 39 | 395 | 17 | 38 | 11536 | 11536 | right |
| 64 | 227 | 12 | 147 | 116 | 17 | 244 | 10 | 21 | 11537 | 11537 | right |
| 63 | 129 | 13 | 76 | 71 | 13 | 102 | 8 | 19 | 11538 | 11538 | left |
| 126 | 359.8 | 25 | 196.1 | 162.3 | 28.2 | 249.7 | 15 | 36 | 11539 | 11539 | left |
| 369 | 1024 | 80 | 512 | 463 | 84 | 735 | 38 | 112 | 11540 | 11540 | left |
| 128.7 | 312.8 | 28 | 190 | 158.4 | 31.9 | 381.3 | 16.6 | 37.6 | 11541 | 11541 | right |
| 97.7 | 273 | 18.7 | 161.9 | 128.6 | 19.6 | 217.7 | 9.4 | 23.9 | 11542 | 11542 | left |
| 92.1 | 238.9 | 17.7 | 163.6 | 134.3 | 21.3 | 340.5 | 11.2 | 30.4 | 11543 | 11543 | right |
| 137.1 | 354.2 | 28.5 | 202.8 | 160.2 | 35.6 | 293.5 | 16.8 | 31.2 | 11544 | 11544 | left |
| 96.7 | 222.2 | 18.8 | 132.8 | 111.4 | 19.5 | 207.1 | 11.9 | 25.7 | 11545 | 11545 | left |
| 123.8 | 238 | 27.8 | 133.5 | 125.2 | 25.3 | 197 | 18.9 | 33.8 | 11546 | 11546 | right |
| 93.7 | 252.7 | 17.9 | 160.9 | 146.2 | 21.1 | 336 | 12.8 | 30.4 | 11547 | 11547 | right |
| 96.1 | 208.4 | 19 | 130.7 | 107.7 | 21.2 | 182.4 | 11.9 | 28.6 | 11548 | 11548 | left |
| 62.2 | 173.5 | 11.4 | 112.7 | 89.3 | 14.9 | 187.3 | 7.7 | 22.8 | 11549 | 11549 | left |
| 88 | 293 | 18 | 181 | 152 | 22 | 321 | 12 | 30 | 11550 | 11550 | left |
| 110.2 | 297.6 | 20.9 | 178.5 | 159.3 | 24.1 | 280.7 | 14.3 | 31 | 11551 | 11551 | right |
| 115 | 346.6 | 25 | 209.4 | 179.9 | 31.5 | 480.1 | 19.3 | 25 | 11552 | 11552 | right |
| 123.2 | 191.5 | 21.3 | 111.3 | 99.1 | 24.2 | 183.9 | 14.9 | 37.8 | 11553 | 11553 | left |
| 91.4 | 295.9 | 14.3 | 190.5 | 144.3 | 22.1 | 336.1 | 8.5 | 27.8 | 11554 | 11554 | left |
| 70 | 145.1 | 13.1 | 91.1 | 77.3 | 3.7 | 121.1 | 9.5 | 26.5 | 11555 | 11555 | left |
| 107.1 | 228.4 | 19.9 | 149.3 | 120.7 | 25.2 | 210.6 | 12.6 | 32.4 | 11556 | 11556 | right |
| 66.6 | 219.1 | 12.6 | 140 | 114.7 | 21.7 | 353.5 | 9.3 | 25.8 | 11557 | 11557 | right |
| 84.3 | 140.8 | 16.8 | 83.2 | 74.4 | 17.1 | 103.1 | 12.2 | 26.3 | 11558 | 11558 | right |
| 127.8 | 335.1 | 19.2 | 212.6 | 165.8 | 28 | 288.1 | 11.9 | 28.6 | 11559 | 11559 | right |
| 93.5 | 179.3 | 16.4 | 118.4 | 102.7 | 20 | 171.7 | 13.6 | 32.4 | 11560 | 11560 | right |
| 76.4 | 186.7 | 13.5 | 123.8 | 97.3 | 17.8 | 206 | 9 | 30.8 | 11562 | 11562 | right |
| 471 | 1401 | 90 | 812 | 659 | 125 | 1065 | 57 | 130 | 11563 | 11563 | left |
| 95.2 | 209 | 16.8 | 128.6 | 108.6 | 19.8 | 213.2 | 11.7 | 31.5 | 11564 | 11564 | right |
| 83.2 | 277.4 | 14.9 | 173.7 | 154.4 | 18.3 | 250.2 | 10.1 | 24.4 | 11565 | 11565 | right |
| 121.9 | 252.7 | 24.9 | 139.3 | 118.5 | 23 | 227.2 | 15.3 | 39.5 | 11566 | 11566 | right |
| 100.1 | 261.4 | 19 | 156.3 | 128.1 | 20.3 | 236.2 | 11.6 | 29.3 | 11567 | 11567 | left |
| 113.6 | 289.8 | 21 | 184.1 | 163.6 | 27.1 | 268.4 | 14.1 | 34.6 | 11568 | 11568 | right |
| 118.7 | 449.1 | 18.2 | 296.5 | 239.8 | 30 | 501.3 | 14.2 | 36.2 | 11569 | 11569 | right |
| 117.7 | 216.6 | 26.9 | 118.7 | 108.2 | 22 | 183 | 16.1 | 37.8 | 11570 | 11570 | right |
| 111 | 324.3 | 20.3 | 203.6 | 167.7 | 29.7 | 360.9 | 12.7 | 32.5 | 11571 | 11571 | left |
| 106.9 | 339.9 | 23.6 | 207.9 | 172.1 | 25.5 | 307.9 | 14.7 | 32.7 | 11572 | 11572 | left |
| 92.4 | 260.2 | 15.8 | 156.4 | 126.7 | 19.2 | 314.9 | 9.9 | 30.7 | 11573 | 11573 | left |
| 147.1 | 367.4 | 33 | 180.4 | 179.8 | 28 | 253 | 20.8 | 41.5 | 11574 | 11574 | right |
| 86.3 | 263 | 13.7 | 180.4 | 139.8 | 18.5 | 280.1 | 9.6 | 28.7 | 11575 | 11575 | left |
| 147.2 | 433.9 | 35.3 | 276.5 | 231.9 | 41.7 | 291.2 | 24.7 | 62.9 | 11576 | 11576 | left |
| 99.2 | 223.6 | 18.3 | 119.5 | 106 | 19.8 | 167.9 | 11.2 | 29.1 | 11577 | 11577 | left |
| 102.6 | 256.5 | 19.6 | 156.9 | 133.8 | 22.1 | 214.7 | 10.9 | 25.1 | 11578 | 11578 | right |
| 96 | 311.8 | 16.3 | 215.7 | 169.6 | 24.5 | 400.6 | 10.2 | 30.9 | 11579 | 11579 | left |
| 110.6 | 291.1 | 25.3 | 203.3 | 160.4 | 30.9 | 206.6 | 14.8 | 31.5 | 11580 | 11580 | right |
| 91.8 | 290.3 | 17.4 | 187.1 | 158.4 | 20.5 | 364.5 | 10.5 | 29.9 | 11581 | 11581 | right |
| 81.8 | 238.5 | 14.8 | 153.2 | 139.1 | 18.5 | 307.9 | 11.5 | 24.1 | 11582 | 11582 | right |
| 100.1 | 234.8 | 20.7 | 132.4 | 106.4 | 19 | 184.1 | 12.1 | 27.4 | 11583 | 11583 | left |
| 130.9 | 329.3 | 24.6 | 200.3 | 161.4 | 30.8 | 218.3 | 15.1 | 30.9 | 11584 | 11584 | right |
| 83.7 | 205.2 | 16.2 | 133.6 | 106.6 | 18.5 | 197.1 | 7.8 | 25.1 | 11585 | 11585 | right |

| | | | | | | | | | | | |
|---|---|---|---|---|---|---|---|---|---|---|---|
| 89.2 | 259.7 | 16.8 | 182.6 | 146.2 | 21.6 | 252 | 10.3 | 24 | 11586 | 11586 | right |
| 105.7 | 180.6 | 22.5 | 116.1 | 95.8 | 25 | 233.3 | 14.4 | 33.5 | 11587 | 11587 | left |
| 577.7 | 1551.8 | 72.5 | 1307.1 | 882.1 | 138.8 | 2204.1 | 43.3 | 121.7 | 11588 | 11588 | right |
| 75.5 | 116.1 | 15.5 | 76.8 | 62.1 | 14.6 | 87.3 | 10 | 22.5 | 11589 | 11589 | right |
| 99.2 | 195.3 | 19.7 | 122.8 | 101 | 20 | 192.6 | 13.7 | 30.7 | 11590 | 11590 | right |
| 90.3 | 207.2 | 16.6 | 128.6 | 108.4 | 18 | 244.3 | 10.7 | 23.4 | 11591 | 11591 | left |
| 106.5 | 311.6 | 17.1 | 233.7 | 174.6 | 27.6 | 315.3 | 11.3 | 25.8 | 11592 | 11592 | right |
| 105 | 372 | 25 | 188 | 166 | 23 | 194 | 14 | 58 | 12989 | 12989 | right |
| 124.6 | 370.7 | 26.5 | 214.1 | 189.5 | 31.2 | 284.6 | 16.3 | 40.4 | 12990 | 12990 | right |
| 52.9 | 303.8 | 9.3 | 215.6 | 171.5 | 13.4 | 260.8 | 5.3 | 19.2 | 12991 | 12991 | right |
| 73.8 | 224.2 | 16 | 134.4 | 119.2 | 17.1 | 187.7 | 11.3 | 32.7 | 12992 | 12992 | right |
| 87.9 | 390 | 15.7 | 190.5 | 174.7 | 15 | 251.5 | 12.7 | 37.9 | 12993 | 12993 | right |
| 63.4 | 143.8 | 10.9 | 95.5 | 80 | 13.7 | 137.2 | 7.5 | 20.5 | 12994 | 12994 | right |
| 70.7 | 249.1 | 11.8 | 157.6 | 132.4 | 13 | 220.9 | 6.4 | 27.7 | 12995 | 12995 | right |
| 86.1 | 159.8 | 15.9 | 94.8 | 83.9 | 16.5 | 151.6 | 9.6 | 21.5 | 12996 | 12996 | right |
| 78.2 | 260 | 13.8 | 143.5 | 136.5 | 14.3 | 215.1 | 10 | 30.7 | 12997 | 12997 | right |
| 91.9 | 170.2 | 18.6 | 100.1 | 97.8 | 19 | 156.3 | 13.5 | 40.6 | 12998 | 12998 | right |
| 52 | 287.8 | 10 | 181 | 152.1 | 11.9 | 211.9 | 5.5 | 18 | 12999 | 12999 | right |
| 87.9 | 261 | 17.9 | 135.9 | 122.5 | 18.6 | 174.5 | 11.3 | 36.2 | 13000 | 13000 | right |
| 115 | 262 | 22 | 145 | 127 | 21 | 235 | 13 | 37 | 13001 | 13001 | right |
| 82.9 | 251.1 | 16.4 | 139 | 132.4 | 18.7 | 191 | 11.7 | 33.1 | 13002 | 13002 | right |
| 61.4 | 880.6 | 15 | 599.7 | 437.4 | 18 | 607 | 8.4 | 52.7 | 13003 | 13003 | right |
| 95.1 | 279.6 | 20.6 | 166.4 | 145.7 | 22.2 | 219.4 | 12 | 26.1 | 13004 | 13004 | right |
| 84.5 | 428.7 | 13 | 302.35 | 228.4 | 21.75 | 514.35 | 10.05 | 27.65 | 13005 | 13005-r1 | right |
| 113 | 406 | 18 | 249 | 204 | 25 | 328 | 14 | 28 | 13006 | 13006 | right |
| 83 | 498 | 13 | 379 | 286 | 24 | 588 | 8 | 29 | 13007 | 13007 | right |
| 77.4 | 418.9 | 13.4 | 286.1 | 222.3 | 19.1 | 311.9 | 6.7 | 21.8 | 13008 | 13008 | right |
| 79.1 | 223.9 | 13.2 | 145.5 | 121.2 | 16.6 | 191.7 | 9.2 | 24.1 | 13009 | 13009 | right |
| 49.8 | 311 | 8.1 | 196.1 | 164.6 | 9.7 | 296.4 | 4.9 | 25.2 | 13010 | 13010 | right |
| 66.4 | 162.7 | 13 | 102.5 | 95.4 | 13.3 | 129.2 | 13.5 | 29.3 | 13011 | 13011 | right |
| 124 | 312 | 24 | 172 | 171 | 25 | 253 | 20 | 51 | 13012 | 13012 | right |
| 75.1 | 192.2 | 15.3 | 126.3 | 95.5 | 15.1 | 138.8 | 9.4 | 27.2 | 13013 | 13013 | right |
| 60.1 | 404.3 | 10.5 | 284.3 | 214.9 | 16.5 | 451.2 | 6.1 | 22 | 13014 | 13014 | right |
| 65.6 | 285.2 | 12.5 | 180.6 | 154.6 | 14.7 | 242.9 | 7.2 | 28.6 | 13015 | 13015 | right |
| 84.3 | 274.5 | 14.9 | 177.8 | 148.8 | 19.3 | 269.3 | 9 | 28.1 | 13016 | 13016 | right |
| 86 | 304 | 18.4 | 177.7 | 153.5 | 20 | 282.9 | 10.2 | 26.8 | 13017 | 13017 | right |
| 90.7 | 690 | 19 | 519.8 | 359.7 | 35.5 | 526.9 | 10.3 | 32.6 | 13018 | 13018 | double |
| 52 | 537 | 3 | 476 | 308 | 9 | 466 | 3 | 22 | 12405 | 12405 | double |
| 129 | 1805 | 31 | 1399 | 1302 | 19 | 2315 | 20 | 94 | 12406 | 12406 | double |
| 146 | 443 | 29 | 251 | 215 | 32 | 328 | 17 | 41 | 12844 | 12844 | left |
| 795 | 2317 | 145 | 1246 | 1153 | 151 | 1788 | 83 | 231 | 12845 | 12845 | left |
| 62 | 317 | 14 | 232 | 186 | 18 | 343 | 9 | 24 | 12846 | 12846 | left |
| 81 | 423 | 15 | 322 | 228 | 22 | 372 | 6 | 25 | 12847 | 12847 | left |
| 63 | 360 | 10 | 246 | 221 | 9 | 297 | 7 | 24 | 12848 | 12848 | left |
| 58 | 421 | 11 | 258 | 220 | 11 | 363 | 5 | 20 | 12849 | 12849 | left |
| 77 | 361 | 17 | 195 | 164 | 15 | 193 | 7 | 35 | 12852 | 12852 | left |
| 70 | 186 | 16 | 110 | 98 | 15 | 140 | 12 | 32 | 12853 | 12853 | left |
| 104 | 435 | 22 | 263 | 216 | 24 | 296 | 12 | 28 | 12854 | 12854 | left |
| 87 | 260 | 14 | 163 | 135 | 16 | 230 | 8 | 21 | 12855 | 12855 | left |
| 91 | 333 | 14 | 192 | 176 | 11 | 261 | 10 | 38 | 12856 | 12856 | left |
| 81 | 369 | 14 | 209 | 190 | 15 | 301 | 10 | 44 | 12857 | 12857 | left |
| 92 | 246 | 19 | 134 | 116 | 16 | 180 | 12 | 36 | 12858 | 12858 | left |
| 66 | 379 | 11 | 265 | 210 | 16 | 323 | 6 | 20 | 12859 | 12859 | left |
| 81 | 196 | 20 | 121 | 112 | 15 | 159 | 11 | 32 | 12860 | 12860 | left |
| 91 | 278 | 20 | 168 | 143 | 18 | 283 | 8 | 26 | 12861 | 12861 | left |
| 125 | 249 | 23 | 146 | 130 | 20 | 175 | 17 | 37 | 12862 | 12862 | left |

| | | | | | | | | | | | |
|---|---|---|---|---|---|---|---|---|---|---|---|
| 77 | 261 | 15 | 168 | 138 | 17 | 227 | 10 | 28 | 12863 | 12863 | left |
| 137 | 521 | 28 | 315 | 261 | 30 | 430 | 14 | 33 | 12864 | 12864 | left |
| 60 | 372 | 9 | 226 | 188 | 11 | 278 | 7 | 20 | 12865 | 12865 | left |
| 89 | 230 | 16 | 127 | 110 | 14 | 159 | 7 | 24 | 12866 | 12866 | left |
| 72 | 331 | 14 | 203 | 170 | 13 | 226 | 10 | 39 | 12867 | 12867 | left |
| 92 | 199 | 19 | 116 | 98 | 17 | 158 | 8 | 23 | 12868 | 12868 | left |
| 77 | 757 | 16 | 640 | 431 | 34 | 675 | 10 | 31 | 12869 | 12869 | left |
| 51 | 421 | 11 | 308 | 229 | 12 | 441 | 5 | 23 | 12870 | 12870 | left |
| 117 | 396 | 18 | 281 | 223 | 26 | 479 | 12 | 31 | 12871 | 12871 | left |
| 205 | 967 | 35 | 469 | 457 | 23 | 548 | 22 | 64 | 12872 | 12872 | left |
| 34 | 293 | 7 | 222 | 163 | 9 | 264 | 3 | 13 | 12873 | 12873 | left |
| 119 | 394 | 25 | 232 | 200 | 26 | 271 | 12 | 32 | 12876 | 12876 | left |
| 93 | 225 | 18 | 132 | 118 | 18 | 220 | 11 | 28 | 12877 | 12877 | left |
| 79 | 303 | 15 | 179 | 165 | 16 | 293 | 8 | 22 | 12878 | 12878 | left |
| 91 | 319 | 17 | 227 | 182 | 22 | 246 | 14 | 37 | 12879 | 12879 | left |
| 127 | 271 | 26 | 158 | 143 | 24 | 254 | 15 | 37 | 12880 | 12880 | left |
| 89 | 223 | 17 | 131 | 117 | 17 | 196 | 10 | 31 | 12881 | 12881 | left |
| 124 | 677 | 27 | 429 | 335 | 34 | 409 | 11 | 26 | 12883 | 12883 | left |
| 79 | 179 | 18 | 112 | 99 | 17 | 171 | 11 | 28 | 12885 | 12885 | left |
| 108 | 154 | 25 | 90 | 79 | 25 | 132 | 16 | 34 | 12886 | 12886 | left |
| 98 | 228 | 22 | 158 | 138 | 23 | 226 | 13 | 32 | 12888 | 12887 | left |
| 85 | 237 | 15 | 168 | 138 | 23 | 219 | 12 | 27 | 12890 | 12890 | left |
| 68 | 286 | 12 | 163 | 150 | 10 | 193 | 11 | 29 | 12892 | 12892 | left |
| 137 | 316 | 32 | 194 | 163 | 30 | 268 | 16 | 34 | 12894 | 12894 | left |
| 98 | 263 | 22 | 155 | 135 | 19 | 206 | 10 | 28 | 12895 | 12895 | left |
| 104 | 260 | 25 | 137 | 131 | 19 | 173 | 14 | 42 | 12896 | 12896 | left |
| 102 | 240 | 23 | 149 | 126 | 22 | 200 | 13 | 26 | 12897 | 12897 | left |
| 75 | 207 | 14 | 128 | 117 | 14 | 156 | 9 | 24 | 12898 | 12898 | left |
| 108 | 268 | 24 | 149 | 124 | 18 | 199 | 12 | 31 | 12899 | 12899 | left |
| 90 | 268 | 19 | 175 | 148 | 21 | 254 | 11 | 29 | 12900 | 12900 | left |
| 89 | 288 | 18 | 164 | 144 | 20 | 221 | 8 | 21 | 12901 | 12901 | left |
| 58 | 305 | 11 | 195 | 160 | 11 | 291 | 5 | 14 | 12903 | 12903 | left |
| 1341.1 | 3944.2 | 270.2 | 2136.9 | 1934.9 | 264 | 2693.4 | 209.9 | 250 | 10782A | 10782A | right |
| 844.6 | 5078.3 | 98.3 | 2944 | 2768.9 | 135.8 | 3770.7 | 431.1 | 163.7 | 10782AA | 10782AA | right |
| 619.4 | 2099.4 | 107.1 | 1164.8 | 1072.4 | 116.7 | 1518.9 | 154.9 | 151.6 | 10782AB | 10782AB | right |
| 643.7 | 2507 | 83.7 | 1473 | 1219.6 | 113.6 | 2138.2 | 222.2 | 140.1 | 10782AC | 10782AC | right |
| 496.6 | 2445.2 | 60.5 | 1561.4 | 1318.2 | 112.6 | 1966.9 | 78.1 | 127.3 | 10782AD | 10782AD | right |
| 538.2 | 3951.8 | 108.4 | 2847.1 | 2124.5 | 161.7 | 3386.6 | 123.6 | 297.9 | 10782B | 10782B | right |
| 407 | 1999.5 | 57 | 1318.9 | 1067 | 96.6 | 1923.7 | 67.8 | 83.1 | 10782C | 10782C | right |
| 444.8 | 1467.7 | 60.2 | 873.3 | 781.4 | 98.9 | 993.3 | 99.1 | 96.3 | 10782D | 10782D | right |
| 682.9 | 4254.3 | 128.6 | 2140 | 1838.6 | 150.7 | 2475.1 | 272.7 | 220.9 | 10782E | 10782E | right |
| 921.1 | 4984.2 | 173.6 | 2884.4 | 2681.7 | 206.6 | 3599.5 | 355.4 | 241.5 | 10782F | 10782F | right |
| 515 | 2923.9 | 83.3 | 1844.9 | 1531.1 | 120.5 | 2600.2 | 156.1 | 108.9 | 10782G | 10782G | right |
| 765 | 1682 | 130.7 | 937.2 | 891.6 | 170 | 1400.9 | 60 | 236.2 | 10782H | 10782H | right |
| 748.5 | 2027.1 | 127.8 | 1051.4 | 998 | 156.8 | 1361.4 | 123.2 | 221.8 | 10782I | 10782I | right |
| 994 | 3924.3 | 112.7 | 2520.6 | 2396 | 161.5 | 3146.9 | 315.7 | 194.8 | 10782J | 10782J | right |
| 889.1 | 3259 | 119.6 | 1723.7 | 1685.5 | 129.9 | 2391.7 | 215.6 | 185.2 | 10782K | 10782K | right |
| 589.1 | 2083.8 | 97.9 | 1264.3 | 1079.7 | 121.2 | 1491.1 | 121.6 | 159.7 | 10782K | 10782K | right |
| 440.2 | 2481.9 | 48.1 | 1604.2 | 1352.3 | 98.8 | 2330.1 | 185 | 93.4 | 10782L | 10782L | right |
| 444.1 | 2543.8 | 65.3 | 1327.3 | 1114.6 | 106 | 1695.1 | 94.7 | 93.9 | 10782M | 10782M | right |
| 954 | 3633 | 163 | 2283.1 | 2134.7 | 194.1 | 2974.4 | 228.2 | 225.8 | 10782N | 10782N | right |
| 429.5 | 3221.8 | 68.8 | 2024.7 | 1726 | 94.6 | 2664 | 170.6 | 166.8 | 10782O | 10782O | right |
| 764 | 3180.4 | 130 | 1840 | 1742.8 | 139.6 | 2387.1 | 135.6 | 187.2 | 10782P | 10782P | right |
| 688.3 | 3089.2 | 115.3 | 1869.2 | 1599.7 | 145.9 | 2562 | 180.3 | 147.9 | 10782Q | 10782Q | right |
| 541.4 | 4118.3 | 69.4 | 2849 | 2471.1 | 125.4 | 4130.4 | 105.1 | 106.6 | 10782R | 10782R | right |
| 563.1 | 4306.9 | 86.3 | 2835.5 | 2640.1 | 113.9 | 4268.8 | 161.4 | 167 | 10782S | 10782S | right |

| | | | | | | | | | | | |
|---|---|---|---|---|---|---|---|---|---|---|---|
| 978.1 | 2524.4 | 163.8 | 1508.8 | 1396.6 | 210 | 2525.2 | 116.8 | 256.4 | 10782T | 10782T | right |
| 576.2 | 3063.7 | 83.2 | 2029.9 | 1584 | 120.1 | 3144.4 | 103.9 | 127 | 10782U | 10782U | right |
| 1008 | 7470.1 | 148.3 | 4334.2 | 4149 | 191.1 | 5662 | 444.6 | 208.5 | 10782V | 10782V | right |
| 763.9 | 5643.2 | 78.3 | 3410.8 | 3114.9 | 150.2 | 4621.9 | 339.9 | 342.6 | 10782W | 10782W | right |
| 743.5 | 1751.1 | 130.9 | 910 | 853 | 147.6 | 1081.1 | 52.2 | 206.2 | 10782X | 10782X | right |
| 467.5 | 1789.1 | 75.7 | 1119 | 916 | 110.8 | 1407.1 | 193.4 | 96.4 | 10782Y | 10782Y | right |
| 1589 | 13461.1 | 254.3 | 8487.7 | 8365.6 | 259.8 | 10959.4 | 467.6 | 333.3 | 10782Z | 10782Z | right |
| 619.2 | 5169.9 | 82.4 | 3072 | 2269 | 150.1 | 3441.7 | 65.7 | 141.5 | 10783A | 10783A | left |
| 453.2 | 1923.3 | 60.4 | 1301.7 | 1067.1 | 110 | 1842.2 | 46.9 | 131 | 10783AA | 10783AA | left |
| 417.7 | 2429.6 | 41.6 | 1509.4 | 1310.4 | 84.9 | 2836 | 62.7 | 119.9 | 10783AB | 10783AB | left |
| 463.3 | 2341.2 | 53.2 | 1509.6 | 1258.3 | 95.2 | 2257 | 116.5 | 120 | 10783AC | 10783AC | left |
| 688.1 | 2574.1 | 100.4 | 1517.6 | 1282.5 | 127.2 | 1871.2 | 234.6 | 213.1 | 10783AD | 10783AD | left |
| 832.1 | 3165.6 | 116.9 | 1733.6 | 1487.4 | 152.1 | 2394.9 | 222.6 | 179 | 10783B | 10783B | left |
| 492.4 | 2029.4 | 78.1 | 1297.6 | 1134.2 | 122.7 | 1575.8 | 62.4 | 193.7 | 10783C | 10783C | left |
| 731.2 | 5350.6 | 98.7 | 3803.3 | 2563.7 | 225.2 | 4328.2 | 261.1 | 173 | 10783D | 10783D | left |
| 967.1 | 2607.9 | 190 | 1393.6 | 1257.4 | 183.7 | 2109 | 146.2 | 200.5 | 10783E | 10783E | left |
| 441 | 7275.9 | 139.4 | 6063.6 | 3890.9 | 234 | 6123.5 | 99.3 | 130.3 | 10783F | 10783F | left |
| 1073.2 | 3911.5 | 207.9 | 2047.6 | 1726.1 | 282.6 | 2404.1 | 249.2 | 250.4 | 10783G | 10783G | left |
| 556.7 | 3368.1 | 83.8 | 2001.2 | 1638.9 | 131.7 | 2053 | 86.5 | 188.4 | 10783H | 10783H | left |
| 991.6 | 4143.7 | 149.4 | 2217.9 | 1921 | 176.5 | 2734.2 | 279.1 | 165 | 10783I | 10783I | left |
| 937.6 | 4416.1 | 149.6 | 2363.4 | 2118.2 | 169.9 | 3512.7 | 370.1 | 197.8 | 10783J | 10783J | left |
| 975.9 | 2859.1 | 165.6 | 1506.5 | 1456.8 | 170.6 | 1940.8 | 195.6 | 214.9 | 10783L | 10783L | left |
| 770.5 | 2467.6 | 125.2 | 1269.1 | 1202.4 | 155.3 | 1697.5 | 134.1 | 150.9 | 10783M | 10783M | left |
| 1018.5 | 4313.7 | 153.5 | 2641.6 | 2446.1 | 178.9 | 3433.8 | 313.5 | 197.2 | 10783N | 10783N | left |
| 440.5 | 1904.6 | 62 | 1271 | 1106.1 | 89.2 | 1753.1 | 37.8 | 127.2 | 10783O | 10783O | left |
| 902.1 | 2684.9 | 170.2 | 1680 | 1454.3 | 216.5 | 2707.1 | 189.6 | 240 | 10783P | 10783P | left |
| 445.5 | 1753.7 | 49.4 | 1173.2 | 962.7 | 105.7 | 1615.4 | 43.2 | 127.3 | 10783Q | 10783Q | left |
| 776 | 2330.9 | 144.5 | 1306.5 | 1145.4 | 147.3 | 1641.9 | 130.9 | 139.7 | 10783R | 10783R | left |
| 1230 | 6262.9 | 161.7 | 3145.7 | 2887.7 | 195.2 | 4019.8 | 304.2 | 252.5 | 10783S | 10783S | left |
| 619.6 | 1569.3 | 98.7 | 900 | 786.1 | 139.7 | 1212.9 | 40.8 | 168.2 | 10783T | 10783T | left |
| 391.6 | 3516.3 | 57.2 | 2637.2 | 1961.8 | 108.3 | 3416.1 | 68.4 | 311.8 | 10783U | 10783U | left |
| 1042.5 | 1956.2 | 214.4 | 1082.4 | 910.2 | 249.8 | 1863.5 | 116.1 | 317.1 | 10783W | 10783W | left |
| 572.4 | 2821.2 | 88.2 | 1909.1 | 1597.5 | 147.7 | 2494.5 | 45.8 | 183.2 | 10783X | 10783X | left |
| 375.1 | 2236.9 | 59.9 | 1553.2 | 1262.3 | 97.9 | 2156.3 | 33.9 | 100.5 | 10783Y | 10783Y | left |
| 787.3 | 5158.1 | 89.5 | 2981.8 | 2712 | 160.1 | 3650.2 | 222.8 | 199.2 | 10783Z | 10783Z | left |
| 1144.1 | 3465.8 | 209.3 | 1925.4 | 1702.6 | 249.3 | 2381.3 | 124.8 | 237.9 | 10784A | 10784A | left |
| 768.9 | 2086.4 | 135.1 | 1161.6 | 1120.3 | 167.6 | 1722.4 | 133.2 | 188.3 | 10784AA | 10784AA | left |
| 487.1 | 1377.4 | 93.1 | 928.6 | 756.2 | 130.4 | 1247.2 | 53.7 | 121.2 | 10784AB | 10784AB | left |
| 816.1 | 1977.3 | 131.3 | 1102 | 997.6 | 157.8 | 1573.3 | 178.9 | 229.8 | 10784AC | 10784AC | left |
| 1129.8 | 2721.2 | 191.8 | 1715.6 | 1599.3 | 256.5 | 3701.5 | 50.3 | 410.7 | 10784AD | 10784AD | left |
| 919.2 | 1568.4 | 123.9 | 1255.4 | 809.5 | 228.7 | 2250.5 | 129.3 | 242.3 | 10784B | 10784B | left |
| 1011.3 | 2468.8 | 208 | 1321.3 | 1187.9 | 212.8 | 2053.9 | 237 | 233.3 | 10784C | 10784C | left |
| 1320.7 | 3234.4 | 254.3 | 1800.5 | 1543.9 | 314.1 | 2171.2 | 171.8 | 304.6 | 10784D | 10784D | left |
| 693.7 | 2352.2 | 108.3 | 1412.6 | 1238.4 | 150.5 | 2530.9 | 148.3 | 179.5 | 10784E | 10784E | left |
| 628.9 | 2084.1 | 83.6 | 1321.2 | 1134.4 | 140.5 | 2129.2 | 127.6 | 173.7 | 10784F | 10784F | left |
| 616.7 | 2834 | 116.9 | 1860.6 | 1534.4 | 196.8 | 2673.6 | 76 | 216.3 | 10784G | 10784G | left |
| 892.1 | 1926.8 | 150.4 | 1082.6 | 1031.4 | 175.9 | 1548.2 | 93.2 | 225.2 | 10784H | 10784H | left |
| 770 | 2218.2 | 133.7 | 1301.5 | 1098.6 | 170.5 | 2036.3 | 82 | 191.5 | 10784I | 10784I | left |
| 855.8 | 2290.8 | 139.1 | 1358.1 | 1166.6 | 189.8 | 1915 | 48.2 | 197.1 | 10784J | 10784J | left |
| 955.5 | 2761.9 | 163.2 | 1435.8 | 1385.8 | 190.6 | 2149.8 | 224.7 | 221.4 | 10784K | 10784K | left |
| 1176.8 | 4813.6 | 232.6 | 2615.8 | 2263 | 262.9 | 2945.8 | 361.7 | 251.1 | 10784L | 10784L | left |
| 894.8 | 2681.2 | 129 | 1586.6 | 1299.3 | 184.8 | 2366.4 | 105.8 | 189.8 | 10784M | 10784M | left |
| 736.8 | 1935.1 | 115.8 | 1193 | 1017.4 | 181.3 | 1889.6 | 51.6 | 222.3 | 10784N | 10784N | left |
| 682.5 | 1851.2 | 97.7 | 1071.7 | 901 | 139 | 1568.5 | 41.5 | 151.8 | 10784O | 10784O | left |
| 843.4 | 1799.1 | 143.8 | 1058.6 | 933.3 | 179 | 1465.5 | 86.1 | 258.9 | 10784P | 10784P | left |
| 693.5 | 1904.7 | 123.4 | 1058.2 | 952.1 | 142.9 | 1495.5 | 37.1 | 205.3 | 10784Q | 10784Q | left |

| | | | | | | | | | | | |
|---|---|---|---|---|---|---|---|---|---|---|---|
| 913.3 | 5293.7 | 174.1 | 3561.4 | 2621.3 | 290.5 | 3710.9 | 112.5 | 219.4 | 10784R | 10784R | left |
| 687.9 | 1855.2 | 126.9 | 1086.7 | 1006.5 | 179.9 | 1824.5 | 56.3 | 221.6 | 10784S | 10784S | left |
| 760.4 | 1433.9 | 128.1 | 893.7 | 782 | 170.3 | 1829 | 46.2 | 211.7 | 10784T | 10784T | left |
| 1114.9 | 2328.8 | 195.4 | 1213.3 | 1177.5 | 218.1 | 1621.1 | 75 | 271.3 | 10784U | 10784U | left |
| 1102.7 | 3274.9 | 213.1 | 2090.4 | 1844.5 | 339.9 | 3328.6 | 61.5 | 343 | 10784V | 10784V | left |
| 490.2 | 1702 | 65.9 | 1151.8 | 946.4 | 112.9 | 1413.3 | 31.4 | 115.4 | 10784W | 10784W | left |
| 579.4 | 1455.5 | 93.4 | 948.4 | 776.7 | 140.3 | 1331.2 | 30.2 | 176.5 | 10784X | 10784X | left |
| 447.3 | 1430.3 | 74.7 | 901.9 | 772.4 | 119.9 | 1074.1 | 64.7 | 118.7 | 10784Y | 10784Y | left |
| 892.1 | 1573.6 | 181.1 | 905.1 | 805.8 | 190.7 | 1430.2 | 41.5 | 253.7 | 10784Z | 10784Z | left |
| 1092.3 | 2130 | 186.9 | 1190.9 | 1130.2 | 206.9 | 2070.1 | 83.9 | 276.3 | 10785A | 10785A | right |
| 748 | 1652 | 125.3 | 1104.8 | 934 | 159.6 | 1732 | 82.7 | 166.5 | 10785AA | 10785AA | right |
| 951.5 | 3000.8 | 160.3 | 1792.1 | 1529.4 | 228 | 3136.4 | 62.2 | 232.8 | 10785AB | 10785AB | right |
| 980.2 | 4318.7 | 142.1 | 2702.7 | 2173.2 | 278.3 | 4188.3 | 97.8 | 236.5 | 10785AC | 10785AC | right |
| 1100.5 | 2479.6 | 205.8 | 1476.9 | 1305.9 | 271.5 | 2619.8 | 108.9 | 262.4 | 10785AD | 10785AD | right |
| 841.8 | 3226.7 | 144.4 | 2544.1 | 1927.5 | 267.5 | 3567.9 | 56.7 | 195.9 | 10785B | 10785B | right |
| 806.8 | 3134.6 | 133.1 | 1960.7 | 1614.6 | 226.9 | 3516.3 | 52.2 | 255.1 | 10785C | 10785C | right |
| 868.4 | 2373.7 | 153.2 | 1326.4 | 1101.2 | 184.6 | 2103.5 | 80.4 | 230.6 | 10785D | 10785D | right |
| 945.1 | 2135.8 | 187.2 | 1153.6 | 1007.4 | 190.8 | 1895.8 | 145.9 | 222.1 | 10785E | 10785E | right |
| 904.4 | 2511.5 | 175.4 | 1620.2 | 1386.4 | 259.7 | 2707.3 | 43.5 | 256.7 | 10785F | 10785F | right |
| 668.2 | 2723.6 | 117.6 | 1461.7 | 1274.1 | 152.6 | 2623.3 | 52.1 | 147.6 | 10785G | 10785G | right |
| 885.7 | 3022.2 | 168.3 | 1791.6 | 1467 | 236 | 2484.4 | 45.5 | 228.6 | 10785H | 10785H | right |
| 1297.1 | 4134.6 | 264.4 | 2763 | 2197.5 | 388.9 | 3500 | 47.7 | 311.3 | 10785I | 10785I | right |
| 1004.3 | 2536.2 | 176.3 | 1530.8 | 1287.4 | 196.8 | 2073.1 | 63.7 | 255.6 | 10785J | 10785J | right |
| 921.8 | 2644.1 | 141.3 | 1541.7 | 1407.4 | 207 | 2541.3 | 93.1 | 289.6 | 10785K | 10785K | right |
| 880.6 | 2065.6 | 161.1 | 1158 | 1118.3 | 202.3 | 1813.1 | 77.8 | 260.3 | 10785L | 10785L | right |
| 842.6 | 1551.6 | 164.6 | 899 | 868.3 | 177.8 | 1388.7 | 65.3 | 244 | 10785M | 10785M | right |
| 1220 | 4354.3 | 220 | 2825.4 | 2378.2 | 331.2 | 4414.6 | 52.8 | 306.4 | 10785N | 10785N | right |
| 1022.4 | 2043.4 | 165.2 | 1099.8 | 1033.8 | 191.6 | 1739.1 | 79.3 | 251.3 | 10785O | 10785O | right |
| 1126 | 2213.9 | 205.1 | 1351.2 | 1230.4 | 274.8 | 3955 | 42.5 | 397.7 | 10785P | 10785P | right |
| 1508 | 3905.5 | 322.4 | 2329.7 | 1988.4 | 381.5 | 3352.7 | 461.5 | 382.7 | 10785Q | 10785Q | right |
| 1095.2 | 2530.4 | 191.8 | 1590.5 | 1399.8 | 254.4 | 2556.3 | 71.3 | 321.3 | 10785R | 10785R | right |
| 1516.7 | 3651.9 | 314 | 2403.1 | 2067.5 | 399.4 | 4468.8 | 138.4 | 470.8 | 10785S | 10785S | right |
| 1327.9 | 2721.9 | 230.9 | 1479.1 | 1268.8 | 267.5 | 1848 | 98 | 310.9 | 10785T | 10785T | right |
| 996.1 | 2542.7 | 171.2 | 1468.4 | 1283.8 | 197.9 | 2230.8 | 105.3 | 228.3 | 10785U | 10785U | right |
| 738.4 | 2520.7 | 132.1 | 1515.4 | 1271.7 | 162.9 | 2287.6 | 37.7 | 211.6 | 10785V | 10785V | right |
| 1075.4 | 3994.8 | 188.9 | 3052 | 2226.7 | 358.8 | 3794.1 | 114.1 | 298.3 | 10785W | 10785W | right |
| 857.6 | 1923.2 | 149.6 | 1232 | 1040.5 | 178.6 | 1664.2 | 52.5 | 196.6 | 10785X | 10785X | right |
| 568.1 | 2243.1 | 91.6 | 1349.9 | 1105.4 | 124 | 2526.9 | 88.3 | 161.2 | 10785Y | 10785Y | right |
| 534.1 | 1555.9 | 94.6 | 1078.3 | 891.2 | 138.9 | 1204.5 | 45.5 | 125.3 | 10785Z | 10785Z | right |
| 833.7 | 2270.4 | 151.5 | 1460.1 | 1248.5 | 196 | 2688.2 | 102.3 | 220.2 | 10786A | 10786A | left |
| 747 | 1949.9 | 146.7 | 1110.1 | 1024.5 | 163.9 | 1576.6 | 57.8 | 178.1 | 10786AA | 10786AA | left |
| 904.1 | 1840.7 | 164.3 | 1262.5 | 1069.7 | 213.9 | 2413.9 | 53.5 | 218 | 10786AB | 10786AB | left |
| 930.3 | 2880 | 159.1 | 1826.4 | 1493.3 | 216 | 3777.9 | 47.7 | 271.3 | | | left |
| 475.4 | 1694.9 | 81.9 | 1150.8 | 988.3 | 128.5 | 1398.2 | 59.9 | 114.7 | 10786AD | 10786AD | left |
| 675.3 | 1879.1 | 121.3 | 1204 | 1021 | 151.6 | 1424.2 | 133.4 | 167.5 | 10786B | 10786B | left |
| 717.3 | 2458.3 | 125 | 1552.1 | 1233 | 170.4 | 2292 | 60.5 | 153.2 | 10786C | 10786C | left |
| 836.7 | 1710.2 | 144.5 | 949.5 | 918.5 | 152.9 | 1285.8 | 147.6 | 223.7 | 10786D | 10786D | left |
| 543.6 | 4962.1 | 103 | 3237.2 | 2402 | 159.6 | 3770.7 | 107.6 | 150.7 | 10786E | 10786E | left |
| 1390.6 | 3802.1 | 252 | 2280.5 | 1868.1 | 345.7 | 4400.7 | 118.6 | 392.7 | 10786F | 10786F | left |
| 580.5 | 1668.4 | 86.2 | 947.3 | 806.5 | 120.7 | 1239.4 | 149.8 | 175.9 | 10786G | 10786G | left |
| 1193 | 5417.7 | 208.7 | 3384.1 | 3160.9 | 244.2 | 3973.7 | 323.5 | 280.3 | 10786H | 10786H | left |
| 679.5 | 1546.2 | 97 | 1036.4 | 863.7 | 129.5 | 1407.9 | 116.8 | 144.8 | 10786I | 10786I | left |
| 938.5 | 2573.7 | 170.5 | 1648.2 | 1291.8 | 243.6 | 2461.4 | 88.2 | 220.6 | 10786J | 10786J | left |
| 1174 | 2296.7 | 207.7 | 1432.3 | 1230.5 | 255.7 | 2359.2 | 174.8 | 345.1 | 10786K | 10786K | left |
| 689.7 | 1442.9 | 121.8 | 940.2 | 850.3 | 149 | 1381.4 | 127.9 | 193.6 | 10786L | 10786L | left |
| 619.4 | 1416.7 | 110 | 872.1 | 765.2 | 146.3 | 1370.5 | 41.7 | 170 | 10786M | 10786M | left |

| | | | | | | | | | | | |
|---|---|---|---|---|---|---|---|---|---|---|---|
| 988.6 | 2798.6 | 171.4 | 1636.8 | 1400.3 | 228.9 | 2747.7 | 37.2 | 229.9 | 10786N | 10786N | left |
| 1112.5 | 1810.5 | 202 | 1109.5 | 1043.1 | 258.1 | 1865.7 | 118.6 | 324.4 | 10786O | 10786O | left |
| 985.3 | 2303.9 | 191.9 | 1265 | 1119.2 | 214.3 | 1536.2 | 144.5 | 213.2 | 10786P | 10786P | left |
| 649.4 | 1402 | 107.2 | 800.6 | 687.8 | 145.5 | 1031.9 | 41.6 | 135.6 | 10786Q | 10786Q | left |
| 573.2 | 2131.7 | 90.2 | 1428.6 | 1218.6 | 123.4 | 2102.6 | 41.7 | 133.1 | 10786R | 10786R | left |
| 874.8 | 3404.6 | 141.1 | 2080.3 | 1701.5 | 247.1 | 4128.1 | 42.4 | 194.5 | 10786S | 10786S | left |
| 1151.1 | 2377.1 | 197.5 | 1374 | 1396.2 | 245.8 | 3215.7 | 45.6 | 367.7 | 10786T | 10786T | left |
| 571.9 | 3010.5 | 88.3 | 1835.3 | 1548.8 | 128.2 | 2543.7 | 199.9 | 119.3 | 10786U | 10786U | left |
| 1048.6 | 2991.2 | 191.2 | 1950.6 | 1585.6 | 243.5 | 2805 | 53.8 | 276.6 | 10786V | 10786V | left |
| 746.4 | 1875.8 | 113.1 | 1197.5 | 1044.7 | 157.4 | 1570.9 | 158.3 | 159.2 | 10786W | 10786W | left |
| 587 | 1258.9 | 121.4 | 795.7 | 706.7 | 136.7 | 1048.4 | 46.6 | 167.9 | 10786X | 10786X | left |
| 696.3 | 2340.9 | 126.2 | 1406.2 | 1154.6 | 176.9 | 1914.8 | 47.7 | 183.4 | 10786Y | 10786Y | left |
| 1399.7 | 4317.8 | 255.4 | 2866.7 | 2182.4 | 379.9 | 5215.7 | 44.6 | 361.8 | 10786Z | 10786Z | left |
| 620.1 | 1391.4 | 103.9 | 852.9 | 748.3 | 149.3 | 1320.9 | 47.5 | 164.1 | 10787A | 10787A | left |
| 909.5 | 1276.5 | 143.5 | 802.7 | 731.8 | 193.9 | 1289.5 | 37.3 | 251.6 | 10787AA | 10787AA | right |
| 1164.4 | 2322.4 | 199.7 | 1333.5 | 1236.2 | 267 | 2761.8 | 51.1 | 340.4 | 10787AB | 10787AB | right |
| 979.2 | 1487.1 | 178.3 | 818.9 | 809.7 | 219.2 | 1134.9 | 34.4 | 289 | 10787AC | 10787AC | left |
| 892.2 | 2215.1 | 174.7 | 1328.1 | 1123.8 | 239 | 3188.3 | 46.4 | 266.8 | 10787AD | 10787AD | right |
| 1212.9 | 2916.1 | 207.6 | 1858.9 | 1691.4 | 311.3 | 4228.7 | 151.4 | 334.6 | 10787B | 10787B | right |
| 609.1 | 2219.6 | 86.6 | 1386.2 | 1152.8 | 161.7 | 2226.5 | 62.5 | 153.5 | 10787C | 10787C | right |
| 1650.8 | 2962.3 | 321.7 | 1712.1 | 1602.4 | 332 | 2933.3 | 141.1 | 477.7 | 10787D | 10787D | left |
| 1199.2 | 2677.9 | 231.2 | 1579.1 | 1374.7 | 302.8 | 2835 | 44.9 | 312.3 | 10787E | 10787E | left |
| 1274.8 | 3528.6 | 261.7 | 2122.4 | 1844.5 | 359.3 | 3625.3 | 48.1 | 358.6 | 10787F | 10787F | left |
| 1002.1 | 3369.9 | 190 | 1716.6 | 1558.3 | 205.6 | 2007.4 | 272.2 | 205.3 | 10787G | 10787G | left |
| 1256.3 | 2315.9 | 229 | 1392.9 | 1233.1 | 301.5 | 1836.6 | 117.7 | 321.5 | 10787H | 10787H | left |
| 730.2 | 1561.8 | 134.1 | 916.5 | 793.2 | 162.1 | 2234 | 26.8 | 202.4 | 10787I | 10787I | right |
| 690.8 | 1486 | 107.7 | 914.6 | 832 | 160.6 | 1129.1 | 43.3 | 186.3 | 10787J | 10787J | right |
| 786.1 | 2980.8 | 114 | 1447.4 | 1348.2 | 109.4 | 1643.8 | 266 | 106 | 10787K | 10787K | right |
| 1127.3 | 2116.1 | 211.9 | 1259.8 | 1094.2 | 258.6 | 2038.8 | 43.4 | 266.7 | 10787L | 10787L | left |
| 817.7 | 1132.2 | 177.1 | 657.5 | 580.9 | 198.2 | 1304.2 | 61.6 | 288.4 | 10787M | 10787M | right |
| 869.5 | 1625.3 | 154.1 | 1007.2 | 950.5 | 189 | 2670.7 | 36.5 | 242.5 | 10787N | 10787N | left |
| 1110.1 | 2906.1 | 154.1 | 1777.7 | 1648.7 | 224.1 | 3933.6 | 56.4 | 302.6 | 10787O | 10787O | right |
| 1368.5 | 3874.8 | 271.3 | 2462.7 | 2010.7 | 390.6 | 4276.9 | 95.4 | 326.7 | 10787P | 10787P | left |
| 1304.6 | 2667.1 | 254.1 | 1549.8 | 1451.9 | 288.8 | 2875 | 56.7 | 340.2 | 10787Q | 10787Q | left |
| 1305.7 | 2761.2 | 237.4 | 1503.8 | 1483.8 | 286.7 | 2369.5 | 56.6 | 332.6 | 10787R | 10787R | right |
| 956 | 3389.1 | 163.7 | 2184.5 | 1689.2 | 252.6 | 2966.7 | 45.7 | 233.8 | 10787S | 10787S | right |
| 716 | 1866.8 | 134.5 | 1047.6 | 907.1 | 163.1 | 1641.8 | 87.5 | 176.3 | 10787T | 10787T | left |
| 848 | 2986 | 129.5 | 1728.3 | 1544.1 | 162 | 2279.4 | 121.5 | 177 | 10787U | 10787U | left |
| 1125 | 2159.9 | 227.8 | 1329.3 | 1181.4 | 279.2 | 2200 | 48.2 | 301.4 | 10787V | 10787V | left |
| 1238.7 | 2983.8 | 229.4 | 1726.3 | 1637.8 | 298.7 | 3540.8 | 70.2 | 400.2 | 10787W | 10787W | right |
| 887.7 | 1744.4 | 145 | 1033.1 | 929.9 | 175.3 | 1370 | 106.3 | 205.1 | 10787X | 10787X | right |
| 1161.7 | 2422.4 | 204.3 | 1599.4 | 1342.2 | 311.7 | 2875.3 | 125.6 | 344 | 10787Y | 10787Y | left |
| 1099.2 | 2105.1 | 209.9 | 1166.6 | 1023.9 | 253.3 | 1936.5 | 43.7 | 309.9 | 10787Z | 10787Z | right |
| 861.7 | 2011 | 124.1 | 1341.2 | 1074.4 | 201.8 | 2018.6 | 55.2 | 218.9 | 10788A | 10788A | left |
| 1043.1 | 1749.8 | 182 | 1025.9 | 878.3 | 222.6 | 1453.3 | 35 | 250.1 | 10788AA | 10788AA | right |
| 871.9 | 2773.8 | 152 | 1943.8 | 1542.2 | 244.6 | 3148.5 | 47.4 | 234.1 | 10788AB | 10788AB | left |
| 1062.6 | 2239.9 | 172.7 | 1403.9 | 1173 | 245.7 | 1863.2 | 88.2 | 257.8 | 10788B | 10788B | right |
| 1242.8 | 2684 | 252.5 | 1827.2 | 1506.4 | 365.4 | 2535.8 | 46.4 | 293.4 | 10788C | 10788C | left |
| 1002.7 | 2028.3 | 220.7 | 1142.3 | 1048 | 249.6 | 1637.8 | 30.9 | 257.3 | 10788D | 10788D | right |
| 586.8 | 1972.5 | 97.6 | 1501.9 | 1147.2 | 174 | 1796.9 | 64.9 | 140.6 | 10788E | 10788E | right |
| 859.6 | 1657 | 134 | 1176 | 1019.3 | 202.8 | 2026.9 | 70.4 | 263.6 | 10788F | 10788F | right |
| 1194.6 | 4703.3 | 203.9 | 2697 | 2159.1 | 290.1 | 3289.6 | 125.9 | 232.4 | 10788G | 10788G | right |
| 1190.9 | 2686.2 | 205.2 | 1642.9 | 1415.2 | 290.6 | 3851.6 | 60 | 347.7 | 10788I | 10788I | right |
| 1245.2 | 2851.6 | 244 | 1817.5 | 1536.7 | 354.6 | 5663.8 | 64.4 | 365.2 | 10788J | 10788J | right |
| 825.6 | 1777 | 147.3 | 1022.5 | 904.3 | 194.4 | 1624.3 | 49.2 | 214.9 | 10788K | 10788K | left |
| 496.2 | 1566 | 66.1 | 1062.2 | 849.3 | 116.6 | 1423.1 | 40.4 | 77.9 | 10788L | 10788L | right |

| | | | | | | | | | | | |
|---|---|---|---|---|---|---|---|---|---|---|---|
| 638.8 | 1451.9 | 121.8 | 928.2 | 793.5 | 149.4 | 1224.5 | 48.6 | 137.4 | 10788M | 10788M | left |
| 731.4 | 1755.4 | 133.7 | 1118.9 | 976.6 | 175.3 | 1496.7 | 66.7 | 171.4 | 10788N | 10788N | left |
| 1122.7 | 2589.5 | 175.3 | 1599.6 | 1332.7 | 278.1 | 2248.8 | 57.6 | 383.6 | 10788O | 10788O | left |
| 749.4 | 2015.3 | 115 | 1235.8 | 1096.4 | 154.5 | 1946.8 | 24.3 | 204.9 | 10788P | 10788P | left |
| 1353.5 | 3287.6 | 266.9 | 1963.6 | 1716.1 | 324.1 | 2911.3 | 66.5 | 336 | 10788Q | 10788Q | left |
| 1118.8 | 1644 | 205.5 | 969 | 926.1 | 226.9 | 1507.5 | 30.2 | 310.3 | 10788R | 10788R | left |
| 581.9 | 1449.2 | 92.7 | 973.5 | 812.9 | 137.1 | 1195.9 | 46.2 | 138.5 | 10788S | 10788S | left |
| 675.8 | 2194.6 | 106.8 | 1244.1 | 1080.6 | 156.9 | 1768.6 | 106.9 | 178.4 | 10788T | 10788T | right |
| 916.9 | 1870.9 | 148.9 | 1140.1 | 1053.9 | 198.5 | 2284.7 | 56.9 | 263.1 | 10788U | 10788U | right |
| 1080.6 | 2757.8 | 149.8 | 1831.9 | 1452.4 | 268.8 | 3446 | 40.9 | 263.3 | 10788V | 10788V | left |
| 675.9 | 1740.2 | 91.5 | 1177.8 | 947.2 | 149.2 | 2406.1 | 52.3 | 198.4 | 10788W | 10788W | left |
| 1240.2 | 2033.7 | 208.4 | 1224.6 | 1097 | 270.9 | 2226.6 | 63.3 | 393.8 | 10788X | 10788X | right |
| 678.8 | 1855.8 | 117.8 | 1131.6 | 960.1 | 165.5 | 2256.2 | 27.1 | 215.9 | 10788Y | 10788Y | right |
| 1103.5 | 2575.7 | 242 | 1442.8 | 1273.9 | 256.3 | 2378.3 | 111.2 | 308.1 | 10788Z | 10788Z | right |
| 1362 | 2787.5 | 277 | 1634.6 | 1436.1 | 303.4 | 2989.7 | 39.6 | 315 | 10789A | 10789A | left |
| 1116.7 | 2084.3 | 199.7 | 1161.7 | 1023.4 | 247.7 | 2252.7 | 71.6 | 290.1 | 10789AA | 10789AA | left |
| 711.8 | 1822.6 | 106.5 | 1171.9 | 1012.7 | 150.4 | 2054.6 | 74.3 | 201.5 | 10789AB | 10789AB | left |
| 1027.5 | 3078.4 | 171.2 | 2098.7 | 1641.7 | 272.5 | 3425.5 | 43.7 | 309.7 | 10789AC | 10789AC | left |
| 772 | 1720.8 | 132.1 | 1138 | 945.5 | 193 | 2529.7 | 19.2 | 205.6 | 10789AD | 10789AD | right |
| 761.8 | 2222.1 | 128.8 | 1377.7 | 1110 | 175.7 | 2246.6 | 87.4 | 175.6 | 10789B | 10789B | right |
| 761.7 | 2547.9 | 120.5 | 1543.8 | 1245 | 212.4 | 3076.9 | 38.2 | 211.9 | 10789C | 10789C | right |
| 728.3 | 1894.2 | 142.1 | 1134.9 | 979.5 | 195.6 | 2706.6 | 28.3 | 194.6 | 10789D | 10789D | left |
| 1257.3 | 2609.9 | 221.5 | 1503.5 | 1290.2 | 281.2 | 2763.4 | 103.8 | 389 | 10789E | 10789E | right |
| 1239.6 | 3829.8 | 240.6 | 2471.5 | 1912.9 | 355 | 3953.3 | 54.6 | 340.5 | 10789F | 10789F | right |
| 897.1 | 1608.4 | 160.4 | 921.2 | 862.9 | 187.9 | 1474.6 | 35.5 | 272.4 | 10789G | 10789G | right |
| 1233.9 | 2354.7 | 244.5 | 1425.7 | 1255.3 | 300.3 | 2570.4 | 38.8 | 365 | 10789H | 10789H | right |
| 1087.5 | 4573.2 | 220 | 2733.6 | 2138.4 | 318.1 | 4227.9 | 48.3 | 232.8 | 10789I | 10789I | left |
| 699.5 | 5608.3 | 131.4 | 4612.1 | 3231.7 | 258.1 | 5175.3 | 85.1 | 296.1 | 10789J | 10789J | right |
| 1232.6 | 2509.3 | 226.8 | 1514.3 | 1332.6 | 271.8 | 2095.3 | 51.8 | 325 | 10789K | 10789K | left |
| 1112 | 3601.3 | 179 | 2298.9 | 2087.8 | 284.5 | 3694.7 | 47 | 312.2 | 10789L | 10789L | left |
| 743.2 | 2513 | 124.2 | 1459.9 | 1291.8 | 172.9 | 1898.1 | 95.2 | 197.7 | 10789M | 10789M | right |
| 736 | 1977.4 | 152.2 | 1166.9 | 1030.6 | 183.6 | 1511.3 | 153 | 278.1 | 10789N | 10789N | right |
| 1114.1 | 3148.5 | 203.8 | 2104.3 | 1812.4 | 298.6 | 3480.4 | 40 | 326.4 | 10789O | 10789O | right |
| 1291.7 | 3664.3 | 236.8 | 2403 | 1899.7 | 342.6 | 3905.7 | 73.4 | 347.6 | 10789P | 10789P | left |
| 757.6 | 1576.1 | 131.8 | 1026.9 | 956.6 | 169.8 | 1839.4 | 37.6 | 234.7 | 10789Q | 10789Q | left |
| 753.8 | 2473.6 | 121 | 1576.9 | 1267.9 | 216.4 | 3252.1 | 40.5 | 195 | 10789R | 10789R | left |
| 607.7 | 1419.8 | 142.4 | 19348.7 | 769.6 | 1257.7 | 1166.6 | 9967.3 | 991.7 | 10789S | 10789S | right |
| 1047.7 | 2503 | 196.2 | 1453.1 | 1292.2 | 275.9 | 4099.1 | 69.9 | 329.7 | 10789T | 10789T | right |
| 892.3 | 2743.3 | 155.2 | 1783.4 | 1460.7 | 253.3 | 2986.1 | 53.4 | 226.1 | 10789U | 10789U | left |
| 911.8 | 1893 | 162 | 1240.3 | 1050.4 | 224.1 | 2706.8 | 36.4 | 287.9 | 10789V | 10789V | right |
| 677.2 | 1570.8 | 111.1 | 948.4 | 842.9 | 150 | 1212.4 | 70.2 | 180.6 | 10789W | 10789W | left |
| 895.6 | 2250.9 | 144.5 | 1343.1 | 1096.4 | 202.6 | 2090.6 | 38.7 | 221.8 | 10789X | 10789X | left |
| 428 | 2747.5 | 61.8 | 2070 | 1505.3 | 136.9 | 2639.9 | 42.2 | 121.5 | 10789Y | 10789Y | left |
| 765.4 | 1385.8 | 127 | 830.7 | 724.5 | 157.6 | 1510.6 | 38.6 | 248.5 | 10789Z | 10789Z | right |
| 1062.4 | 3552.5 | 147.1 | 2130.6 | 1779.4 | 243.4 | 4067.7 | 126.8 | 224.6 | 10790A | 10790A | left |
| 812.7 | 4276 | 146.1 | 2049.1 | 1750.5 | 204.9 | 2346.4 | 135.4 | 327.2 | 10790AA | 10790AA | left |
| 601.4 | 1247.6 | 112.1 | 727.7 | 726.1 | 135.7 | 1043.5 | 30.8 | 198.4 | 10790AB | 10790AB | left |
| 375.9 | 1022 | 62.5 | 693.5 | 548.3 | 94.9 | 1145.1 | 23.2 | 104.8 | 10790AC | 10790AC | right |
| 606.7 | 3702.2 | 108.4 | 2625.2 | 1991.3 | 205 | 3543.3 | 95.9 | 177.7 | 10790AD | 10790AD | left |
| 728.3 | 4911 | 117.8 | 3677.5 | 2607.5 | 206.9 | 5098.3 | 631.5 | 166.3 | 10790B | 10790B | right |
| 787.1 | 2204.4 | 125 | 1227.9 | 1113.9 | 178.8 | 1756 | 120.7 | 163.5 | 10790C | 10790C | left |
| 593.9 | 1415.7 | 96.2 | 874.7 | 757.2 | 129.2 | 1296.9 | 76.9 | 141.8 | 10790D | 10790D | left |
| 668.6 | 1731.5 | 123 | 1317.6 | 1045 | 171.2 | 1894.8 | 131.9 | 211.5 | 10790E | 10790E | left |
| 1027.6 | 2784.7 | 189.9 | 1555.2 | 1297.8 | 248 | 2486.4 | 32.7 | 226.8 | 10790F | 10790F | left |
| 587.3 | 1998.5 | 105.4 | 1367.8 | 1110.3 | 156.7 | 1801 | 124.3 | 158.6 | 10790G | 10790G | right |
| 536.3 | 1376.1 | 81.4 | 830.8 | 779.5 | 105.5 | 1134.9 | 48.7 | 192.4 | 10790H | 10790H | right |

| | | | | | | | | | | | |
|---|---|---|---|---|---|---|---|---|---|---|---|
| 625.8 | 1563.1 | 114.6 | 1040.4 | 899 | 145.3 | 1276.3 | 50.4 | 179.1 | 10790I | 10790I | left |
| 1176 | 2401.3 | 216.6 | 1433.9 | 1269.2 | 294.8 | 3204.7 | 41.9 | 375.9 | 10790J | 10790J | right |
| 1020.9 | 2178.1 | 193.8 | 1284.4 | 1140.5 | 220.7 | 2034.9 | 67.9 | 260 | 10790K | 10790K | left |
| 712.7 | 2394.9 | 132.2 | 1265 | 1110.2 | 149.2 | 1792.3 | 106.2 | 153.6 | 10790L | 10790L | left |
| 589.1 | 1329.1 | 91.8 | 813.5 | 706.9 | 131.8 | 1013 | 52.3 | 179 | 10790M | 10790M | right |
| 1333.9 | 3960.7 | 254.4 | 2576.6 | 2079.2 | 390.5 | 4393.8 | 86.9 | 369.8 | 10790N | 10790N | right |
| 480.8 | 1618.6 | 90.1 | 1098.2 | 942.9 | 127.9 | 1598.8 | 99.9 | 148.6 | 10790O | 10790O | left |
| 758.7 | 2213.3 | 132.7 | 1362.9 | 1140.2 | 212.3 | 2080.7 | 106.4 | 238.8 | 10790P | 10790P | left |
| 559.4 | 1488.4 | 93.6 | 968.6 | 773.3 | 141.1 | 1320.4 | 46.4 | 144.8 | 10790Q | 10790Q | right |
| 630.4 | 1905 | 113.5 | 1300.4 | 1087 | 169.1 | 1900.7 | 148.9 | 184.2 | 10790R | 10790R | right |
| 580.3 | 2112.8 | 100.8 | 1522.9 | 1226.7 | 176.1 | 2105.3 | 46 | 149.9 | 10790S | 10790S | right |
| 661.6 | 3126.2 | 94.2 | 2417.8 | 2141.2 | 168.9 | 4382.5 | 100.6 | 219.5 | 10790T | 10790T | left |
| 943.4 | 2338.7 | 143.5 | 1431.5 | 1276 | 187 | 2103.7 | 66.8 | 289 | 10790U | 10790U | left |
| 622 | 1350.8 | 108.9 | 905.5 | 792.8 | 143.6 | 1439.4 | 77.3 | 168.3 | 10790V | 10790V | left |
| 765.4 | 1865.2 | 130.3 | 1187 | 969 | 161.4 | 2018.9 | 41.3 | 209.1 | 10790W | 10790W | left |
| 602.4 | 2663.3 | 93.7 | 1969.6 | 1547.6 | 159.8 | 2853.6 | 67.7 | 167.6 | 10790X | 10790X | right |
| 1287 | 2581.5 | 260.5 | 1557.3 | 1476.6 | 327.8 | 3972.5 | 44.7 | 417.8 | 10790Y | 10790Y | right |
| 712.1 | 1748.2 | 113.4 | 1104.5 | 984.6 | 137 | 1413.7 | 160.8 | 189 | 10790Z | 10790Z | left |
| 69 | 449 | 14 | 227 | 191 | 15 | 337 | 9 | 33 | 12893B | 12893B | left |
| 35.1 | 98.6 | 8.3 | 57.5 | 53.5 | 8 | 71.8 | 5.2 | 14.1 | 12899B | 12899B | left |
| 84 | 280 | 16 | 181 | 152 | 19 | 324 | 9 | 22 | 12902B | 12902B | left |
