# Supplementary material for: A decline in molluscan carbonate production driven by the loss of vegetated habitats encoded in the Holocene sedimentary record of the Gulf of Trieste
Source: Sedimentology. 2018 Aug 25;66(3):781–807. doi: 10.1111/sed.12516 (PMC6446828; doi:10.1111/sed.12516)
Supplement: Supplementary file 3 — Table S3. Amino acid racemization data and calibrated estimates of post‐mortem age of Corbula gibba and Piran 2. [file SED-66-781-s003.pdf]

Age	UAL_num	Injection	Hydrolysis	Notes	Station	specimen_no
10695.39	12610	2ul10	6		PiranII M53	PiranII M53, 65-70, 01
9866.533	12611	2ul10	6		PiranII M53	PiranII M53, 65-70, 02
6048.419	12612	2ul10	6		PiranII M53	PiranII M53, 65-70, 03
10095.88	12613	2ul10	6		PiranII M53	PiranII M53, 65-70, 04
8138.271	12614	2ul10	6		PiranII M53	PiranII M53, 65-70, 05
7012.75	12615	2ul10	6		PiranII M53	PiranII M53, 65-70, 06
	12616	2ul10	6		PiranII M53	PiranII M53, 65-70, 07
7244.357	12617	2ul10	6		PiranII M53	PiranII M53, 65-70, 08
7734.532	12618	2ul10	6		PiranII M53	PiranII M53, 65-70, 09
5739	12619	2ul10	6		PiranII M53	PiranII M53, 65-70, 10
6795.196	12620	2ul10	6		PiranII M53	PiranII M53, 65-70, 11
6050.761	12621	2ul10	6		PiranII M53	PiranII M53, 65-70, 12
8126.714	12622	2ul10	6		PiranII M53	PiranII M53, 65-70, 13
5211.78	12623	2ul10	6		PiranII M53	PiranII M53, 65-70, 14
7989.462	12624	2ul10	6		PiranII M53	PiranII M53, 65-70, 15
7325.941	12625	2ul10	6		PiranII M53	PiranII M53, 65-70, 16
	12626	2ul10	6		PiranII M53	PiranII M53, 65-70, 17
7864.956	12627	2ul10	6		PiranII M53	PiranII M53, 65-70, 18
7615.956	12628	2ul10	6		PiranII M53	PiranII M53, 65-70, 19
10103.65	12629	2ul10	6		PiranII M53	PiranII M53, 65-70, 20
10757.77	12630	2ul10	6		PiranII M53	PiranII M53, 65-70, 21
9472.635	12631	2ul10	6		PiranII M53	PiranII M53, 65-70, 22
7557.841	12632	2ul10	6		PiranII M53	PiranII M53, 65-70, 23
9208.798	12633	2ul10	6		PiranII M53	PiranII M53, 65-70, 24
9998.385	12634	2ul10	6		PiranII M53	PiranII M53, 65-70, 25
10355.29	12635	2ul10	6		PiranII M53	PiranII M53, 65-70, 26
7699.526	12636	2ul10	6		PiranII M53	PiranII M53, 65-70, 27
9891.28	12637	2ul10	6		PiranII M53	PiranII M53, 65-70, 28
5625.502	12638	2ul10	6		PiranII M53	PiranII M53, 65-70, 29
	12639	2ul10	6		PiranII M53	PiranII M53, 65-70, 30
8594	12640	2ul10	6		PiranII M53	PiranII M53, 85-90, 01
8554	12641	2ul10	6		PiranII M53	PiranII M53, 85-90, 02
7969.469	12642	2ul10	6		PiranII M53	PiranII M53, 85-90, 03
6445.818	12643	2ul10	6		PiranII M53	PiranII M53, 85-90, 04
8792.08	12644	2ul10	6		PiranII M53	PiranII M53, 85-90, 05
6706.991	12645	2ul10	6		PiranII M53	PiranII M53, 85-90, 06
8539.231	12646	2ul10	6		PiranII M53	PiranII M53, 85-90, 07
9707.627	12647	2ul10	6		PiranII M53	PiranII M53, 85-90, 08
5649.722	12648	2ul10	6		PiranII M53	PiranII M53, 85-90, 09
8248.926	12649	2ul10	6		PiranII M53	PiranII M53, 85-90, 10
9409.722	12650	2ul10	6		PiranII M53	PiranII M53, 85-90, 11
9748.153	12651	2ul10	6		PiranII M53	PiranII M53, 85-90, 12
7960.606	12652	2ul10	6		PiranII M53	PiranII M53, 85-90, 13
10158.74	12653	2ul10	6		PiranII M53	PiranII M53, 85-90, 14
10450.79	12654	2ul10	6		PiranII M53	PiranII M53, 85-90, 15
8384.319	12655	2ul10	6		PiranII M53	PiranII M53, 85-90, 16
7669.028	12656	2ul10	6		PiranII M53	PiranII M53, 85-90, 17
6782.948	12657	2ul10	6		PiranII M53	PiranII M53, 85-90, 19
8723.471	12658	2ul10	6		PiranII M53	PiranII M53, 85-90, 20
7390.475	12659	2ul10	6		PiranII M53	PiranII M53, 85-90, 21
6060.736	12660	2ul10	6		PiranII M53	PiranII M53, 85-90, 22
9147.286	12661	2ul10	6		PiranII M53	PiranII M53, 85-90, 23
6493.274	12662	2ul10	6		PiranII M53	PiranII M53, 85-90, 24

9385.268	12663	2ul10	6		PiranII M53	PiranII M53, 85-90, 25
8729.442	12664	2ul10	6		PiranII M53	PiranII M53, 85-90, 26
10738.35	12665	2ul10	6		PiranII M53	PiranII M53, 85-90, 27
6094.04	12666	2ul10	6		PiranII M53	PiranII M53, 85-90, 28
6280.595	12667	2ul10	6		PiranII M53	PiranII M53, 85-90, 29
9055.371	12668	2ul10	6		PiranII M53	PiranII M53, 85-90, 30
6064.341	12669	2ul10	6	1	PiranII M53	PiranII M53, 100-105, 01
8750	12670	2ul10	6	2	PiranII M53	PiranII M53, 100-105, 02
8594	12671	2ul10	6	3	PiranII M53	PiranII M53, 100-105, 03
3782.225	12672	2ul10	6	4	PiranII M53	PiranII M53, 100-105, 04
6575.722	12673	2ul10	6	5	PiranII M53	PiranII M53, 100-105, 05
5123.97	12674	2ul10	6	6	PiranII M53	PiranII M53, 100-105, 06
9838.048	12675	2ul10	6	7	PiranII M53	PiranII M53, 100-105, 07
3941.675	12676	2ul10	6	8	PiranII M53	PiranII M53, 100-105, 08
	12677	2ul10	6	9	PiranII M53	PiranII M53, 100-105, 09
8793.531	12678	2ul10	6	10	PiranII M53	PiranII M53, 100-105, 10
	12680	2ul10	6	12	PiranII M53	PiranII M53, 100-105, 12
7275.374	12681	2ul10	6	13	PiranII M53	PiranII M53, 100-105, 13
6119.362	12682	2ul10	6	14	PiranII M53	PiranII M53, 100-105, 14
4257.341	12684	2ul10	6	16	PiranII M53	PiranII M53, 100-105, 16
7756.893	12685	2ul10	6	17	PiranII M53	PiranII M53, 100-105, 17
5973.365	12686	2ul10	6	18	PiranII M53	PiranII M53, 100-105, 18
	12687	2ul10	6	19	PiranII M53	PiranII M53, 100-105, 19
	12688	2ul10	6	20	PiranII M53	PiranII M53, 100-105, 20
9905.249	12689	2ul10	6	21	PiranII M53	PiranII M53, 100-105, 21
8482.036	12690	2ul10	6	22	PiranII M53	PiranII M53, 100-105, 22
3968.812	12691	2ul10	6	23	PiranII M53	PiranII M53, 100-105, 23
9170.551	12692	2ul10	6	24	PiranII M53	PiranII M53, 100-105, 24
	12693	2ul10	6	25	PiranII M53	PiranII M53, 100-105, 25
6821.056	12694	2ul10	6	26	PiranII M53	PiranII M53, 100-105, 26
6348.593	12696	2ul10	6	28	PiranII M53	PiranII M53, 100-105, 28
	12697	2ul10	6	29	PiranII M53	PiranII M53, 100-105, 29
7753.583	12698	2ul10	6	30	PiranII M53	PiranII M53, 100-105, 30
6037.819	12699	2ul10	6		PiranII M53	PiranII M53, 125-130, 01
7750.931	12700	2ul10	6		PiranII M53	PiranII M53, 125-130, 02
9578.121	12701	2ul10	6		PiranII M53	PiranII M53, 125-130, 03
9459.292	12702	2ul10	6		PiranII M53	PiranII M53, 125-130, 04
8296.716	12706	2ul10	6		PiranII M53	PiranII M53, 125-130, 08
6230.276	12707	2ul10	6		PiranII M53	PiranII M53, 125-130, 09
7305.419	12712	2ul10	6		PiranII M53	PiranII M53, 125-130, 14
6760.07	12713	2ul10	6		PiranII M53	PiranII M53, 125-130, 15
6386.708	12714	2ul10	6		PiranII M53	PiranII M53, 125-130, 16
8154.368	12715	2ul10	6		PiranII M53	PiranII M53, 125-130, 17
9229.583	12716	2ul10	6		PiranII M53	PiranII M53, 125-130, 18
7978.728	12717	2ul10	6		PiranII M53	PiranII M53, 125-130, 19
7836.104	12719	2ul10	6		PiranII M53	PiranII M53, 125-130, 21
7662.466	12720	2ul10	6		PiranII M53	PiranII M53, 125-130, 22
6299.397	12721	2ul10	6		PiranII M53	PiranII M53, 125-130, 23
7800.392	12722	2ul10	6		PiranII M53	PiranII M53, 125-130, 24
6433.214	12723	2ul10	6		PiranII M53	PiranII M53, 125-130, 25
5705.955	12725	2ul10	6		PiranII M53	PiranII M53, 125-130, 27
7463.21	12726	2ul10	6		PiranII M53	PiranII M53, 125-130, 28
6402.124	12727	2ul10	6		PiranII M53	PiranII M53, 125-130, 29
7137.107	12728	2ul10	6		PiranII M53	PiranII M53, 125-130, 30
8997.147	12729	2ul10	6		PiranII M53	PiranII M53, 145-150, 01
	12730	2ul10	6		PiranII M53	PiranII M53, 145-150, 02

6420.347	12731	2ul10	6	PiranII M53	PiranII M53, 145-150, 03
8725.033	12732	2ul10	6	PiranII M53	PiranII M53, 145-150, 04
6896.428	12733	2ul10	6	PiranII M53	PiranII M53, 145-150, 05
8806.796	12734	2ul10	6	PiranII M53	PiranII M53, 145-150, 06
	12735	2ul10	6	PiranII M53	PiranII M53, 145-150, 07
8033.246	12736	2ul10	6	PiranII M53	PiranII M53, 145-150, 08
8140.71	12737	2ul10	6	PiranII M53	PiranII M53, 145-150, 09
5808.087	12738	2ul10	6	PiranII M53	PiranII M53, 145-150, 10
8674.054	12739	2ul10	6	PiranII M53	PiranII M53, 145-150, 11
4381.031	12740	2ul10	6	PiranII M53	PiranII M53, 145-150, 12
8689.159	12741	2ul10	6	PiranII M53	PiranII M53, 145-150, 13
7098.087	12742	2ul10	6	PiranII M53	PiranII M53, 145-150, 14
5818.793	12743	2ul10	6	PiranII M53	PiranII M53, 145-150, 15
8755.775	12744	2ul10	6	PiranII M53	PiranII M53, 145-150, 16
	12745	2ul10	6	PiranII M53	PiranII M53, 145-150, 17
6970.732	12746	2ul10	6	PiranII M53	PiranII M53, 145-150, 18
8614.313	12747	2ul10	6	PiranII M53	PiranII M53, 145-150, 19
9413.962	12748	2ul10	6	PiranII M53	PiranII M53, 145-150, 20
9291.201	12749	2ul10	6	PiranII M53	PiranII M53, 145-150, 21
6929.981	12750	2ul10	6	PiranII M53	PiranII M53, 145-150, 22
9381.065	12751	2ul10	6	PiranII M53	PiranII M53, 145-150, 23
7734.23	12752	2ul10	6	PiranII M53	PiranII M53, 145-150, 24
	12753	2ul10	6	PiranII M53	PiranII M53, 145-150, 25
8823.456	12754	2ul10	6	PiranII M53	PiranII M53, 145-150, 26
	12755	2ul10	6	PiranII M53	PiranII M53, 145-150, 27
4963.652	12756	2ul10	6	PiranII M53	PiranII M53, 145-150, 28
8395.972	12757	2ul10	6	PiranII M53	PiranII M53, 145-150, 29
9132.433	12758	2ul10	6	PiranII M53	PiranII M53, 145-150, 30
59.8161	14766	2ul10	6	PiranII M53	Pir2 M53 0-2 01
1015.504	14767	2ul10	6	PiranII M53	Pir2 M53 0-2 02
16.32641	14768	2ul10	6	PiranII M53	Pir2 M53 0-2 03
2183.843	14769	2ul10	6	PiranII M53	Pir2 M53 0-2 04
1108.204	14770	2ul10	6	PiranII M53	Pir2 M53 0-2 05
42.3674	14771	2ul10	6	PiranII M53	Pir2 M53 0-2 06
177.2344	14772	2ul10	6	PiranII M53	Pir2 M53 0-2 07
1303.015	14773	2ul10	6	PiranII M53	Pir2 M53 0-2 08
152.2273	14774	2ul10	6	PiranII M53	Pir2 M53 0-2 09
7167.898	14775	2ul10	6	PiranII M53	Pir2 M53 0-2 10
5245.562	14776	2ul10	6	PiranII M53	Pir2 M53 0-2 11
2797.544	14777	2ul10	6	PiranII M53	Pir2 M53 0-2 12
109.812	14778	2ul10	6	PiranII M53	Pir2 M53 0-2 13
1140.955	14779	2ul10	6	PiranII M53	Pir2 M53 0-2 14
1890.61	14780	2ul10	6	PiranII M53	Pir2 M53 0-2 15
715.42	14781	2ul10	6	PiranII M53	Pir2 M53 0-2 16
392.3679	14782	2ul10	6	PiranII M53	Pir2 M53 0-2 17
3134.802	14783	2ul10	6	PiranII M53	Pir2 M53 0-2 18
4297.687	14784	2ul10	6	PiranII M53	Pir2 M53 0-2 19
1354.503	14785	2ul10	6	PiranII M53	Pir2 M53 0-2 20
1463.763	14786	2ul10	6	PiranII M53	Pir2 M53 0-2 21
1087.802	14787	2ul10	6	PiranII M53	Pir2 M53 0-2 22
979.8556	14788	2ul10	6	PiranII M53	Pir2 M53 0-2 23
2145.484	14789	2ul10	6	PiranII M53	Pir2 M53 0-2 24
139.7003	14790	2ul10	6	PiranII M53	Pir2 M53 0-2 25
115.1938	14791	2ul10	6	PiranII M53	Pir2 M53 0-2 26
253.1204	14792	2ul10	6	PiranII M53	Pir2 M53 0-2 27
7341.875	14793	2ul10	6	PiranII M53	Pir2 M53 0-2 28

14.70915	14794	2ul10	6	PiranII M53	Pir2 M53 0-2 29
926.1485	14795	2ul10	6	PiranII M53	Pir2 M53 0-2 30
2597.074	14796	2ul10	6	PiranII M53	Pir2 M53 8-10 01
1264.259	14797	2ul10	6	PiranII M53	Pir2 M53 8-10 02
4185.513	14798	2ul10	6	PiranII M53	Pir2 M53 8-10 03
2081.428	14799	2ul10	6	PiranII M53	Pir2 M53 8-10 04
6796.327	14800	2ul10	6	PiranII M53	Pir2 M53 8-10 05
4420.144	14801	2ul10	6	PiranII M53	Pir2 M53 8-10 06
122.1341	14802	2ul10	6	PiranII M53	Pir2 M53 8-10 07
961.3259	14803	2ul10	6	PiranII M53	Pir2 M53 8-10 08
2168.635	14804	2ul10	6	PiranII M53	Pir2 M53 8-10 09
3005.242	14805	2ul10	6	PiranII M53	Pir2 M53 8-10 10
1143.534	14806	2ul10	6	PiranII M53	Pir2 M53 8-10 11
1215.226	14807	2ul10	6	PiranII M53	Pir2 M53 8-10 12
2000.02	14808	2ul10	6	PiranII M53	Pir2 M53 8-10 13
482.898	14809	2ul10	6	PiranII M53	Pir2 M53 8-10 14
3761.876	14810	2ul10	6	PiranII M53	Pir2 M53 8-10 15
6854.14	14811	2ul10	6	PiranII M53	Pir2 M53 8-10 16
447.669	14812	2ul10	6	PiranII M53	Pir2 M53 8-10 17
2275.434	14813	2ul10	6	PiranII M53	Pir2 M53 8-10 18
6761.282	14814	2ul10	6	PiranII M53	Pir2 M53 8-10 19
5848.819	14815	2ul10	6	PiranII M53	Pir2 M53 8-10 20
7291.757	14816	2ul10	6	PiranII M53	Pir2 M53 25-30 01
5672.275	14817	2ul10	6	PiranII M53	Pir2 M53 25-30 02
5521.768	14818	2ul10	6	PiranII M53	Pir2 M53 25-30 03
7916.849	14819	2ul10	6	PiranII M53	Pir2 M53 25-30 04
5015.043	14820	2ul10	6	PiranII M53	Pir2 M53 25-30 05
5606.647	14821	2ul10	6	PiranII M53	Pir2 M53 25-30 06
8769.594	14822	2ul10	6	PiranII M53	Pir2 M53 25-30 07
4567.071	14823	2ul10	6	PiranII M53	Pir2 M53 25-30 08
6227.304	14824	2ul10	6	PiranII M53	Pir2 M53 25-30 09
6912.838	14825	2ul10	6	PiranII M53	Pir2 M53 25-30 10
3381.408	14826	2ul10	6	PiranII M53	Pir2 M53 25-30 11
4787.305	14827	2ul10	6	PiranII M53	Pir2 M53 25-30 12
7886.465	14828	2ul10	6	PiranII M53	Pir2 M53 25-30 13
8181.936	14829	2ul10	6	PiranII M53	Pir2 M53 25-30 14
5240.301	14830	2ul10	6	PiranII M53	Pir2 M53 25-30 15
5758.063	14831	2ul10	6	PiranII M53	Pir2 M53 25-30 16
7469.013	14832	2ul10	6	PiranII M53	Pir2 M53 25-30 17
4376.389	14833	2ul10	6	PiranII M53	Pir2 M53 25-30 18
3559.96	14834	2ul10	6	PiranII M53	Pir2 M53 25-30 19
6186.865	14835	2ul10	6	PiranII M53	Pir2 M53 25-30 20
8106.575	14836	2ul10	6	PiranII M53	Pir2 M53 25-30 21
5256.536	14837	2ul10	6	PiranII M53	Pir2 M53 25-30 22
6234.729	14838	2ul10	6	PiranII M53	Pir2 M53 25-30 23
5402.607	14839	2ul10	6	PiranII M53	Pir2 M53 25-30 24
8980.337	14840	2ul10	6	PiranII M53	Pir2 M53 25-30 25
8149.285	14841	2ul10	6	PiranII M53	Pir2 M53 45-50 01
5455.256	14842	2ul10	6	PiranII M53	Pir2 M53 45-50 02
3245.536	14843	2ul10	6	PiranII M53	Pir2 M53 45-50 03
9079.311	14844	2ul10	6	PiranII M53	Pir2 M53 45-50 04
	14845	2ul10	6	PiranII M53	Pir2 M53 45-50 05
7092.152	14846	2ul10	6	PiranII M53	Pir2 M53 45-50 06
6080.111	14847	2ul10	6	PiranII M53	Pir2 M53 45-50 07
3092.444	14848	2ul10	6	PiranII M53	Pir2 M53 45-50 08
3970.149	14849	2ul10	6	PiranII M53	Pir2 M53 45-50 09

7114.185	14850	2ul10	6		PiranII M53	Pir2 M53 45-50 10
3841.326	14851	2ul10	6		PiranII M53	Pir2 M53 45-50 11
7639.447	14852	2ul10	6		PiranII M53	Pir2 M53 45-50 12
677.1841	14853	2ul10	6		PiranII M53	Pir2 M53 45-50 13
7298.774	14854	2ul10	6		PiranII M53	Pir2 M53 45-50 14
5965.253	14855	2ul10	6		PiranII M53	Pir2 M53 45-50 15
3217.367	14856	2ul10	6		PiranII M53	Pir2 M53 45-50 16
6831.879	14857	2ul10	6		PiranII M53	Pir2 M53 45-50 17
3206.528	14858	2ul10	6		PiranII M53	Pir2 M53 45-50 18
11023.76	14859	2ul10	6		PiranII M53	Pir2 M53 45-50 19
6674.37	14860	2ul10	6		PiranII M53	Pir2 M53 45-50 20
5574.553	14861	2ul10	6		PiranII M53	Pir2 M53 45-50 21
7569.519	14862	2ul10	6		PiranII M53	Pir2 M53 45-50 22
5705.568	14863	2ul10	6		PiranII M53	Pir2 M53 45-50 23
7271.924	14864	2ul10	6		PiranII M53	Pir2 M53 45-50 24
6867.587	14865	2ul10	6		PiranII M53	Pir2 M53 45-50 25
7123.079	10704r	2ul10-2ul12	6		PiranII M53	PiranII M53, 125-130, 06
5989.337	12679r	2ul10	6	11	PiranII M53	PiranII M53, 100-105, 11
7816.115	12683r	2ul10	6	15	PiranII M53	PiranII M53, 100-105, 15
9650.919	12695r	2ul10	6	27	PiranII M53	PiranII M53, 100-105, 27
9097.119	12703r	2ul10	6		PiranII M53	PiranII M53, 125-130, 05
9212.644	12705r	2ul10-2ul12	6		PiranII M53	PiranII M53, 125-130, 07
6211.7	12708r	2ul10	6		PiranII M53	PiranII M53, 125-130, 10
5023.36	12709r	2ul10-2ul12	6		PiranII M53	PiranII M53, 125-130, 11
4913.518	12711r	2ul10	6		PiranII M53	PiranII M53, 125-130, 13
7977.393	12724r	2ul10	6		PiranII M53	PiranII M53, 125-130, 26

depth (cm)	max. depth (cm)	5 cm group (cm)	10 cm group (cm)	Batch	taxon	Outlier	Contamination	Replicate
65-70	70	70	70		Corbula gibba			
65-70	70	70	70		Corbula gibba			
65-70	70	70	70		Corbula gibba			
65-70	70	70	70		Corbula gibba			
65-70	70	70	70		Corbula gibba			
65-70	70	70	70		Corbula gibba			
65-70	70	70	70		Corbula gibba	outlier	Asp DL-Glu DL	
65-70	70	70	70		Corbula gibba			
65-70	70	70	70		Corbula gibba			
65-70	70	70	70		Corbula gibba			
65-70	70	70	70		Corbula gibba			
65-70	70	70	70		Corbula gibba			
65-70	70	70	70		Corbula gibba			
65-70	70	70	70		Corbula gibba			
65-70	70	70	70		Corbula gibba			
65-70	70	70	70		Corbula gibba			
65-70	70	70	70		Corbula gibba	outlier	Asp DL-Glu DL	
65-70	70	70	70		Corbula gibba			
65-70	70	70	70		Corbula gibba			
65-70	70	70	70		Corbula gibba			
65-70	70	70	70		Corbula gibba			
65-70	70	70	70		Corbula gibba			
65-70	70	70	70		Corbula gibba			
65-70	70	70	70		Corbula gibba			
65-70	70	70	70		Corbula gibba			
65-70	70	70	70		Corbula gibba			
65-70	70	70	70		Corbula gibba			
65-70	70	70	70		Corbula gibba			
65-70	70	70	70		Corbula gibba			
65-70	70	70	70		Corbula gibba	outlier	Asp DL-Glu DL	
85-90	90	90	90		Corbula gibba			
85-90	90	90	90		Corbula gibba			
85-90	90	90	90		Corbula gibba			
85-90	90	90	90		Corbula gibba			
85-90	90	90	90		Corbula gibba			
85-90	90	90	90		Corbula gibba			
85-90	90	90	90		Corbula gibba			
85-90	90	90	90		Corbula gibba			
85-90	90	90	90		Corbula gibba			
85-90	90	90	90		Corbula gibba			
85-90	90	90	90		Corbula gibba			
85-90	90	90	90		Corbula gibba			
85-90	90	90	90		Corbula gibba			
85-90	90	90	90		Corbula gibba			
85-90	90	90	90		Corbula gibba			
85-90	90	90	90		Corbula gibba			
85-90	90	90	90		Corbula gibba			
85-90	90	90	90		Corbula gibba			
85-90	90	90	90		Corbula gibba			
85-90	90	90	90		Corbula gibba			
85-90	90	90	90		Corbula gibba			
85-90	90	90	90		Corbula gibba			
85-90	90	90	90		Corbula gibba			

85-90	90	90	90	Corbula gibba		
85-90	90	90	90	Corbula gibba		
85-90	90	90	90	Corbula gibba		
85-90	90	90	90	Corbula gibba		
85-90	90	90	90	Corbula gibba		
85-90	90	90	90	Corbula gibba		
100-105	105	105	110	Corbula gibba		
100-105	105	105	110	Corbula gibba		
100-105	105	105	110	Corbula gibba		
100-105	105	105	110	Corbula gibba		
100-105	105	105	110	Corbula gibba		
100-105	105	105	110	Corbula gibba		
100-105	105	105	110	Corbula gibba		
100-105	105	105	110	Corbula gibba		
100-105	105	105	110	Corbula gibba	outlier	Asp DL-Glu DL
100-105	105	105	110	Corbula gibba		
100-105	105	105	110	Corbula gibba	outlier	Asp DL-Glu DL
100-105	105	105	110	Corbula gibba		
100-105	105	105	110	Corbula gibba		
100-105	105	105	110	Corbula gibba		
100-105	105	105	110	Corbula gibba		
100-105	105	105	110	Corbula gibba		
100-105	105	105	110	Corbula gibba	outlier	Asp DL-Glu DL
100-105	105	105	110	Corbula gibba	outlier	Asp DL-Glu DL
100-105	105	105	110	Corbula gibba		
100-105	105	105	110	Corbula gibba		
100-105	105	105	110	Corbula gibba		
100-105	105	105	110	Corbula gibba		
100-105	105	105	110	Corbula gibba	outlier	Asp DL-Glu DL
100-105	105	105	110	Corbula gibba		
100-105	105	105	110	Corbula gibba		
100-105	105	105	110	Corbula gibba	outlier	High L-Ser/L-Asp
100-105	105	105	110	Corbula gibba		
125-130	130	130	130	Corbula gibba		
125-130	130	130	130	Corbula gibba		
125-130	130	130	130	Corbula gibba		
125-130	130	130	130	Corbula gibba		
125-130	130	130	130	Corbula gibba		
125-130	130	130	130	Corbula gibba		
125-130	130	130	130	Corbula gibba		
125-130	130	130	130	Corbula gibba		
125-130	130	130	130	Corbula gibba		
125-130	130	130	130	Corbula gibba		
125-130	130	130	130	Corbula gibba		
125-130	130	130	130	Corbula gibba		
125-130	130	130	130	Corbula gibba		
125-130	130	130	130	Corbula gibba		
125-130	130	130	130	Corbula gibba		
125-130	130	130	130	Corbula gibba		
125-130	130	130	130	Corbula gibba		
125-130	130	130	130	Corbula gibba		
125-130	130	130	130	Corbula gibba		
125-130	130	130	130	Corbula gibba		
125-130	130	130	130	Corbula gibba		
125-130	130	130	130	Corbula gibba		
145-150	150	150	150	Corbula gibba		
145-150	150	150	150	Corbula gibba	outlier	Asp DL-Glu DL

145-150	150	150	150	Corbula gibba		
145-150	150	150	150	Corbula gibba		
145-150	150	150	150	Corbula gibba		
145-150	150	150	150	Corbula gibba		
145-150	150	150	150	Corbula gibba	outlier	outlier in DL Asp - DL Glu
145-150	150	150	150	Corbula gibba		
145-150	150	150	150	Corbula gibba		
145-150	150	150	150	Corbula gibba		
145-150	150	150	150	Corbula gibba		
145-150	150	150	150	Corbula gibba		
145-150	150	150	150	Corbula gibba		
145-150	150	150	150	Corbula gibba		
145-150	150	150	150	Corbula gibba		
145-150	150	150	150	Corbula gibba		
145-150	150	150	150	Corbula gibba	outlier	Asp DL-Glu DL
145-150	150	150	150	Corbula gibba		
145-150	150	150	150	Corbula gibba		
145-150	150	150	150	Corbula gibba		
145-150	150	150	150	Corbula gibba		
145-150	150	150	150	Corbula gibba		
145-150	150	150	150	Corbula gibba		
145-150	150	150	150	Corbula gibba		
145-150	150	150	150	Corbula gibba	outlier	Asp DL-Glu DL
145-150	150	150	150	Corbula gibba		
145-150	150	150	150	Corbula gibba	outlier	Asp DL-Glu DL
145-150	150	150	150	Corbula gibba		
145-150	150	150	150	Corbula gibba		
145-150	150	150	150	Corbula gibba		
0-2	2	4	10	Corbula gibba		
0-2	2	4	10	Corbula gibba		
0-2	2	4	10	Corbula gibba		
0-2	2	4	10	Corbula gibba		
0-2	2	4	10	Corbula gibba		
0-2	2	4	10	Corbula gibba		
0-2	2	4	10	Corbula gibba		
0-2	2	4	10	Corbula gibba		
0-2	2	4	10	Corbula gibba		
0-2	2	4	10	Corbula gibba		
0-2	2	4	10	Corbula gibba		
0-2	2	4	10	Corbula gibba		
0-2	2	4	10	Corbula gibba		
0-2	2	4	10	Corbula gibba		
0-2	2	4	10	Corbula gibba		
0-2	2	4	10	Corbula gibba		
0-2	2	4	10	Corbula gibba		
0-2	2	4	10	Corbula gibba		
0-2	2	4	10	Corbula gibba		
0-2	2	4	10	Corbula gibba		
0-2	2	4	10	Corbula gibba		
0-2	2	4	10	Corbula gibba		
0-2	2	4	10	Corbula gibba		
0-2	2	4	10	Corbula gibba		
0-2	2	4	10	Corbula gibba		
0-2	2	4	10	Corbula gibba		
0-2	2	4	10	Corbula gibba		
0-2	2	4	10	Corbula gibba		

0-2	2	4	10	Corbula gibba	
0-2	2	4	10	Corbula gibba	
8-10	10	12	20	Corbula gibba	
8-10	10	12	20	Corbula gibba	
8-10	10	12	20	Corbula gibba	
8-10	10	12	20	Corbula gibba	
8-10	10	12	20	Corbula gibba	
8-10	10	12	20	Corbula gibba	
8-10	10	12	20	Corbula gibba	
8-10	10	12	20	Corbula gibba	
8-10	10	12	20	Corbula gibba	
8-10	10	12	20	Corbula gibba	
8-10	10	12	20	Corbula gibba	
8-10	10	12	20	Corbula gibba	
8-10	10	12	20	Corbula gibba	
8-10	10	12	20	Corbula gibba	
8-10	10	12	20	Corbula gibba	
8-10	10	12	20	Corbula gibba	
8-10	10	12	20	Corbula gibba	
8-10	10	12	20	Corbula gibba	
8-10	10	12	20	Corbula gibba	
8-10	10	12	20	Corbula gibba	
25-30	30	30	30	Corbula gibba	
25-30	30	30	30	Corbula gibba	
25-30	30	30	30	Corbula gibba	
25-30	30	30	30	Corbula gibba	
25-30	30	30	30	Corbula gibba	
25-30	30	30	30	Corbula gibba	
25-30	30	30	30	Corbula gibba	
25-30	30	30	30	Corbula gibba	
25-30	30	30	30	Corbula gibba	
25-30	30	30	30	Corbula gibba	
25-30	30	30	30	Corbula gibba	
25-30	30	30	30	Corbula gibba	
25-30	30	30	30	Corbula gibba	
25-30	30	30	30	Corbula gibba	
25-30	30	30	30	Corbula gibba	
25-30	30	30	30	Corbula gibba	
25-30	30	30	30	Corbula gibba	
25-30	30	30	30	Corbula gibba	
25-30	30	30	30	Corbula gibba	
25-30	30	30	30	Corbula gibba	
25-30	30	30	30	Corbula gibba	
25-30	30	30	30	Corbula gibba	
25-30	30	30	30	Corbula gibba	
25-30	30	30	30	Corbula gibba	
25-30	30	30	30	Corbula gibba	
45-50	50	50	50	Corbula gibba	
45-50	50	50	50	Corbula gibba	
45-50	50	50	50	Corbula gibba	
45-50	50	50	50	Corbula gibba	
45-50	50	50	50	Corbula gibba	outlier
45-50	50	50	50	Corbula gibba	
45-50	50	50	50	Corbula gibba	
45-50	50	50	50	Corbula gibba	
45-50	50	50	50	Corbula gibba	

45-50	50	50	50	Corbula gibba		
45-50	50	50	50	Corbula gibba		
45-50	50	50	50	Corbula gibba		
45-50	50	50	50	Corbula gibba		
45-50	50	50	50	Corbula gibba		
45-50	50	50	50	Corbula gibba		
45-50	50	50	50	Corbula gibba		
45-50	50	50	50	Corbula gibba		
45-50	50	50	50	Corbula gibba		
45-50	50	50	50	Corbula gibba		
45-50	50	50	50	Corbula gibba		
45-50	50	50	50	Corbula gibba		
45-50	50	50	50	Corbula gibba		
45-50	50	50	50	Corbula gibba		
45-50	50	50	50	Corbula gibba		
45-50	50	50	50	Corbula gibba		
125-130	130	130	130	Corbula gibba		
100-105	105	105	110	Corbula gibba	2 analyses,	average of r
100-105	105	105	110	Corbula gibba	2 analyses,	average of r
100-105	105	105	110	Corbula gibba		average of r
125-130	130	130	130	Corbula gibba		average of r
125-130	130	130	130	Corbula gibba		average of r
125-130	130	130	130	Corbula gibba		average of r
125-130	130	130	130	Corbula gibba		average of r
125-130	130	130	130	Corbula gibba		average of r
125-130	130	130	130	Corbula gibba	replicate	average of r

ageMedian	ageYng	ageOld	Asp_DL	Glu_DL	DL_Ser	DL_Ala	DL_Val	DL_Phe
NA	NA	NA	0.430	0.151	0.578	0.280	0.150	0.222
NA	NA	NA	0.420	0.157	0.394	0.272	0.128	0.170
NA	NA	NA	0.358	0.126	0.570	0.229	0.100	0.170
NA	NA	NA	0.422	0.139	0.562	0.269	0.119	0.204
NA	NA	NA	0.397	0.132	0.535	0.230	0.102	0.196
NA	NA	NA	0.376	0.135	0.512	0.290	0.111	0.174
NA	NA	NA	0.468	0.173	0.466	0.330	0.169	0.223
NA	NA	NA	0.382	0.146	0.519	0.265	0.121	0.226
NA	NA	NA	0.389	0.130	0.532	0.240	0.118	0.182
5739	5628	5917	0.335	0.143	0.279	0.207	0.097	0.169
NA	NA	NA	0.372	0.128	0.459	0.232	0.098	0.189
NA	NA	NA	0.357	0.128	0.546	0.221	0.092	0.174
NA	NA	NA	0.394	0.153	0.628	0.256	0.127	0.198
NA	NA	NA	0.341	0.103	0.581	0.206	0.072	0.125
NA	NA	NA	0.395	0.145	0.594	0.253	0.112	0.198
NA	NA	NA	0.382	0.143	0.646	0.274	0.123	0.228
NA	NA	NA	0.468	0.139	0.494	0.262	0.107	0.191
NA	NA	NA	0.390	0.124	0.420	0.238	0.097	0.158
NA	NA	NA	0.386	0.180	0.605	0.331	0.159	0.264
NA	NA	NA	0.422	0.161	0.611	0.299	0.142	0.196
NA	NA	NA	0.432	0.162	0.502	0.279	0.149	0.231
NA	NA	NA	0.420	0.131	0.569	0.250	0.108	0.165
NA	NA	NA	0.387	0.136	0.558	0.246	0.115	0.172
NA	NA	NA	0.410	0.155	0.542	0.274	0.127	0.230
NA	NA	NA	0.422	0.164	0.536	0.300	0.140	0.231
NA	NA	NA	0.429	0.134	0.526	0.243	0.106	0.194
NA	NA	NA	0.387	0.136	0.524	0.234	0.124	0.184
NA	NA	NA	0.421	0.144	0.560	0.229	0.124	0.199
NA	NA	NA	0.348	0.145	0.622	0.251	0.118	0.180
NA	NA	NA	0.511	0.166	0.532	0.304	0.157	0.220
8594	8438	8795	0.372	0.136	0.547	0.218	0.117	0.206
8554	8398	8753	0.447	0.155	0.618	0.294	0.143	0.205
NA	NA	NA	0.394	0.127	0.431	0.240	0.106	0.157
NA	NA	NA	0.366	0.128	0.530	0.263	0.095	0.173
NA	NA	NA	0.405	0.139	0.658	0.309	0.119	0.220
NA	NA	NA	0.371	0.139	0.604	0.267	0.130	0.182
NA	NA	NA	0.399	0.138	0.491	0.264	0.120	0.182
NA	NA	NA	0.419	0.141	0.609	0.277	0.134	0.202
NA	NA	NA	0.352	0.118	0.545	0.220	0.087	0.171
NA	NA	NA	0.396	0.158	0.568	0.297	0.136	0.210
NA	NA	NA	0.416	0.163	0.617	0.305	0.143	0.226
NA	NA	NA	0.417	0.137	0.547	0.241	0.111	0.194
NA	NA	NA	0.392	0.127	0.570	0.233	0.096	0.179
NA	NA	NA	0.423	0.156	0.512	0.267	0.143	0.182
NA	NA	NA	0.428	0.160	0.534	0.291	0.134	0.214
NA	NA	NA	0.398	0.136	0.573	0.246	0.118	0.202
NA	NA	NA	0.388	0.149	0.589	0.264	0.127	0.190
NA	NA	NA	0.372	0.133	0.566	0.240	0.110	0.182
NA	NA	NA	0.404	0.128	0.543	0.236	0.104	0.236
NA	NA	NA	0.382	0.117	0.521	0.197	0.069	0.139
NA	NA	NA	0.359	0.130	0.554	0.241	0.095	0.158
NA	NA	NA	0.408	0.148	0.577	0.271	0.118	0.229
NA	NA	NA	0.367	0.135	0.603	0.248	0.114	0.203

NA	NA	NA	0.412	0.146	0.565	0.248	0.131	0.205
NA	NA	NA	0.402	0.150	0.600	0.266	0.129	0.197
NA	NA	NA	0.432	0.155	0.555	0.296	0.145	0.223
NA	NA	NA	0.358	0.134	0.517	0.229	0.102	0.165
NA	NA	NA	0.361	0.114	0.596	0.196	0.080	0.167
NA	NA	NA	0.408	0.158	0.637	0.279	0.138	0.241
NA	NA	NA	0.358	0.116	0.579	0.182	0.093	0.165
8750	8566	9004	0.410	0.149	0.540	0.265	0.115	0.228
8594	8438	8795	0.393	0.131	0.598	0.237	0.109	0.191
NA	NA	NA	0.307	0.111	0.521	0.195	0.085	0.127
NA	NA	NA	0.367	0.172	0.567	0.259	0.146	0.169
NA	NA	NA	0.339	0.130	0.558	0.228	0.109	0.182
NA	NA	NA	0.415	0.174	0.543	0.312	0.168	0.243
NA	NA	NA	0.312	0.102	0.541	0.169	0.073	0.120
NA	NA	NA	0.479	0.179	0.513	0.309	0.158	0.224
NA	NA	NA	0.405	0.144	0.616	0.265	0.125	0.214
NA	NA	NA	0.473	0.145	0.537	0.266	0.123	0.202
NA	NA	NA	0.380	0.142	0.555	0.259	0.130	0.206
NA	NA	NA	0.359	0.137	0.604	0.236	0.117	0.189
NA	NA	NA	0.319	0.145	0.497	0.234	0.130	0.153
NA	NA	NA	0.390	0.138	0.531	0.266	0.119	0.193
NA	NA	NA	0.358	0.135	0.525	0.226	0.105	0.181
NA	NA	NA	0.461	0.169	0.570	0.300	0.158	0.210
NA	NA	NA	0.465	0.170	0.553	0.295	0.131	0.233
NA	NA	NA	0.420	0.151	0.445	0.259	0.133	0.194
NA	NA	NA	0.397	0.131	0.539	0.224	0.097	0.180
NA	NA	NA	0.311	0.113	0.194	0.286	0.139	0.160
NA	NA	NA	0.408	0.145	0.500	0.270	0.127	0.188
NA	NA	NA	0.478	0.162	0.494	0.273	0.130	0.226
NA	NA	NA	0.373	0.142	0.405	0.228	0.112	0.183
NA	NA	NA	0.363	0.128	0.539	0.194	0.106	0.167
NA	NA	NA	0.510	0.218	0.390	0.370	0.285	0.344
NA	NA	NA	0.389	0.123	0.535	0.233	0.098	0.173
NA	NA	NA	0.355	0.122	0.508	0.215	0.094	0.167
NA	NA	NA	0.388	0.139	0.569	0.280	0.146	0.197
NA	NA	NA	0.417	0.131	0.571	0.249	0.112	0.207
NA	NA	NA	0.415	0.152	0.521	0.288	0.132	0.200
NA	NA	NA	0.396	0.129	0.586	0.251	0.107	0.200
NA	NA	NA	0.363	0.134	0.543	0.233	0.132	0.194
NA	NA	NA	0.382	0.143	0.489	0.242	0.105	0.187
NA	NA	NA	0.370	0.127	0.560	0.226	0.115	0.193
NA	NA	NA	0.366	0.118	0.451	0.220	0.090	0.154
NA	NA	NA	0.397	0.139	0.570	0.235	0.113	0.208
NA	NA	NA	0.413	0.159	0.554	0.267	0.136	0.183
NA	NA	NA	0.393	0.147	0.573	0.267	0.116	0.201
NA	NA	NA	0.389	0.138	0.580	0.235	0.127	0.207
NA	NA	NA	0.385	0.141	0.529	0.213	0.120	0.174
NA	NA	NA	0.365	0.114	0.483	0.221	0.093	0.154
NA	NA	NA	0.388	0.140	0.485	0.239	0.126	0.189
NA	NA	NA	0.364	0.156	0.640	0.282	0.134	0.201
NA	NA	NA	0.352	0.114	0.495	0.213	0.082	0.139
NA	NA	NA	0.384	0.135	0.491	0.234	0.111	0.179
NA	NA	NA	0.366	0.134	0.590	0.243	0.110	0.183
NA	NA	NA	0.375	0.117	0.475	0.207	0.088	0.151
NA	NA	NA	0.406	0.135	0.496	0.239	0.101	0.192
NA	NA	NA	0.466	0.140	0.505	0.234	0.086	0.207

NA	NA	NA	0.364	0.162	0.550	0.304	0.149	0.208
NA	NA	NA	0.403	0.136	0.509	0.224	0.113	0.201
NA	NA	NA	0.375	0.150	0.560	0.269	0.126	0.183
NA	NA	NA	0.403	0.152	0.581	0.284	0.140	0.190
NA	NA	NA	0.151	0.041	0.236	0.055	0.014	0.033
NA	NA	NA	0.392	0.132	0.575	0.246	0.119	0.191
NA	NA	NA	0.396	0.135	0.430	0.243	0.112	0.182
NA	NA	NA	0.354	0.104	0.486	0.194	0.077	0.150
NA	NA	NA	0.405	0.147	0.556	0.256	0.126	0.193
NA	NA	NA	0.321	0.103	0.574	0.178	0.079	0.136
NA	NA	NA	0.403	0.162	0.561	0.277	0.134	0.209
NA	NA	NA	0.376	0.128	0.563	0.237	0.102	0.174
NA	NA	NA	0.351	0.141	0.608	0.241	0.114	0.158
NA	NA	NA	0.403	0.159	0.615	0.270	0.152	0.214
NA	NA	NA	0.457	0.175	0.549	0.271	0.144	0.217
NA	NA	NA	0.373	0.127	0.437	0.243	0.099	0.167
NA	NA	NA	0.402	0.132	0.511	0.247	0.109	0.200
NA	NA	NA	0.414	0.139	0.510	0.245	0.120	0.183
NA	NA	NA	0.411	0.143	0.471	0.269	0.122	0.206
NA	NA	NA	0.372	0.155	0.625	0.270	0.139	0.190
NA	NA	NA	0.414	0.138	0.514	0.251	0.118	0.187
NA	NA	NA	0.389	0.163	0.617	0.290	0.149	0.204
NA	NA	NA	0.360	0.184	0.613	0.309	0.149	0.216
NA	NA	NA	0.408	0.139	0.529	0.264	0.116	0.180
NA	NA	NA	0.473	0.153	0.493	0.282	0.110	0.225
NA	NA	NA	0.336	0.118	0.222	0.224	0.089	0.137
NA	NA	NA	0.396	0.142	0.526	0.240	0.123	0.194
NA	NA	NA	0.408	0.127	0.493	0.242	0.108	0.172
NA	NA	NA	0.084	0.027	0.110	0.028	0.008	0.025
NA	NA	NA	0.197	0.086	0.282	0.135	0.040	0.073
NA	NA	NA	0.062	0.025	0.060	0.044	0.011	0.046
NA	NA	NA	0.256	0.099	0.458	0.182	0.054	0.130
NA	NA	NA	0.203	0.098	0.260	0.137	0.047	0.073
NA	NA	NA	0.077	0.051	0.097	0.058	0.011	0.032
NA	NA	NA	0.114	0.033	0.139	0.053	0.012	0.048
NA	NA	NA	0.215	0.096	0.424	0.136	0.038	0.089
NA	NA	NA	0.109	0.038	0.118	0.066	0.014	0.026
NA	NA	NA	0.377	0.149	0.334	0.298	0.111	0.184
NA	NA	NA	0.341	0.172	0.565	0.389	0.129	0.281
NA	NA	NA	0.276	0.113	0.436	0.209	0.067	0.145
NA	NA	NA	0.099	0.031	0.076	0.055	0.013	0.037
NA	NA	NA	0.205	0.109	0.165	0.193	0.063	0.114
NA	NA	NA	0.242	0.093	0.434	0.145	0.043	0.106
NA	NA	NA	0.176	0.080	0.379	0.132	0.035	0.085
NA	NA	NA	0.145	0.047	0.236	0.092	0.018	0.050
NA	NA	NA	0.288	0.156	0.351	0.314	0.124	0.212
NA	NA	NA	0.320	0.158	0.315	0.341	0.126	0.225
NA	NA	NA	0.217	0.107	0.266	0.187	0.055	0.119
NA	NA	NA	0.224	0.105	0.330	0.177	0.062	0.119
NA	NA	NA	0.201	0.091	0.251	0.151	0.046	0.080
NA	NA	NA	0.194	0.052	0.278	0.082	0.021	0.055
NA	NA	NA	0.253	0.086	0.425	0.173	0.046	0.112
NA	NA	NA	0.107	0.044	0.103	0.069	0.021	0.110
NA	NA	NA	0.101	0.039	0.115	0.064	0.015	0.044
NA	NA	NA	0.127	0.044	0.182	0.073	0.016	0.031
NA	NA	NA	0.380	0.161	0.537	0.325	0.133	0.234

NA	NA	NA	0.060	0.025	0.055	0.043	0.012	0.033
NA	NA	NA	0.192	0.044	0.290	0.082	0.017	0.044
NA	NA	NA	0.269	0.086	0.271	0.096	0.022	0.075
NA	NA	NA	0.213	0.081	0.413	0.132	0.038	0.090
NA	NA	NA	0.317	0.121	0.507	0.230	0.082	0.186
NA	NA	NA	0.250	0.114	0.433	0.193	0.062	0.119
NA	NA	NA	0.373	0.154	0.412	0.277	0.104	0.204
NA	NA	NA	0.324	0.142	0.558	0.296	0.106	0.214
NA	NA	NA	0.102	0.025	0.129	0.038	0.008	0.030
NA	NA	NA	0.195	0.044	0.414	0.064	0.014	0.055
NA	NA	NA	0.253	0.104	0.428	0.158	0.041	0.086
NA	NA	NA	0.283	0.103	0.541	0.191	0.061	0.144
NA	NA	NA	0.206	0.081	0.389	0.123	0.032	0.079
NA	NA	NA	0.209	0.066	0.401	0.121	0.029	0.078
NA	NA	NA	0.247	0.078	0.469	0.147	0.037	0.093
NA	NA	NA	0.155	0.045	0.320	0.069	0.017	0.057
NA	NA	NA	0.303	0.102	0.477	0.201	0.057	0.146
NA	NA	NA	0.372	0.171	0.608	0.352	0.136	0.256
NA	NA	NA	0.151	0.042	0.317	0.061	0.016	0.070
NA	NA	NA	0.258	0.109	0.418	0.176	0.063	0.125
NA	NA	NA	0.371	0.163	0.519	0.345	0.128	0.253
NA	NA	NA	0.355	0.150	0.552	0.291	0.112	0.209
NA	NA	NA	0.380	0.132	0.547	0.279	0.094	0.198
NA	NA	NA	0.353	0.137	0.578	0.287	0.097	0.185
NA	NA	NA	0.348	0.140	0.593	0.289	0.100	0.201
NA	NA	NA	0.389	0.146	0.530	0.331	0.113	0.250
NA	NA	NA	0.337	0.119	0.601	0.250	0.081	0.194
NA	NA	NA	0.350	0.136	0.615	0.289	0.102	0.227
NA	NA	NA	0.404	0.151	0.669	0.338	0.126	0.284
NA	NA	NA	0.328	0.129	0.556	0.248	0.091	0.199
NA	NA	NA	0.361	0.156	0.642	0.324	0.114	0.238
NA	NA	NA	0.375	0.121	0.465	0.254	0.082	0.178
NA	NA	NA	0.295	0.115	0.523	0.208	0.071	0.153
NA	NA	NA	0.333	0.130	0.615	0.288	0.090	0.197
NA	NA	NA	0.390	0.166	0.593	0.360	0.126	0.251
NA	NA	NA	0.394	0.183	0.618	0.390	0.155	0.310
NA	NA	NA	0.342	0.141	0.658	0.259	0.095	0.194
NA	NA	NA	0.352	0.143	0.653	0.313	0.104	0.206
NA	NA	NA	0.383	0.133	0.596	0.282	0.099	0.237
NA	NA	NA	0.322	0.128	0.540	0.237	0.082	0.163
NA	NA	NA	0.301	0.104	0.412	0.201	0.065	0.176
NA	NA	NA	0.361	0.140	0.522	0.291	0.107	0.210
NA	NA	NA	0.395	0.172	0.660	0.356	0.144	0.278
NA	NA	NA	0.342	0.146	0.639	0.313	0.112	0.260
NA	NA	NA	0.360	0.132	0.547	0.277	0.097	0.188
NA	NA	NA	0.343	0.116	0.550	0.219	0.076	0.160
NA	NA	NA	0.408	0.179	0.642	0.396	0.148	0.310
NA	NA	NA	0.396	0.154	0.532	0.371	0.137	0.317
NA	NA	NA	0.348	0.124	0.517	0.287	0.090	0.212
NA	NA	NA	0.291	0.090	0.516	0.189	0.056	0.137
NA	NA	NA	0.408	0.150	0.542	0.321	0.118	0.254
NA	NA	NA	0.554	0.153	0.610	0.330	0.111	0.246
NA	NA	NA	0.377	0.176	0.544	0.349	0.133	0.268
NA	NA	NA	0.358	0.144	0.533	0.354	0.116	0.279
NA	NA	NA	0.286	0.097	0.388	0.199	0.058	0.134
NA	NA	NA	0.312	0.143	0.648	0.286	0.100	0.207

NA	NA	NA	0.377	0.162	0.633	0.340	0.125	0.257
NA	NA	NA	0.306	0.121	0.550	0.235	0.078	0.197
NA	NA	NA	0.388	0.147	0.640	0.330	0.114	0.252
NA	NA	NA	0.173	0.039	0.346	0.062	0.015	0.052
NA	NA	NA	0.383	0.149	0.654	0.304	0.119	0.269
NA	NA	NA	0.356	0.120	0.563	0.262	0.087	0.191
NA	NA	NA	0.291	0.091	0.479	0.164	0.051	0.125
NA	NA	NA	0.372	0.164	0.564	0.361	0.128	0.274
NA	NA	NA	0.290	0.079	0.550	0.154	0.043	0.124
NA	NA	NA	0.435	0.161	0.477	0.353	0.128	0.254
NA	NA	NA	0.368	0.121	0.494	0.260	0.082	0.172
NA	NA	NA	0.347	0.138	0.484	0.297	0.110	0.228
NA	NA	NA	0.385	0.182	0.530	0.382	0.141	0.346
NA	NA	NA	0.352	0.179	0.589	0.373	0.145	0.301
NA	NA	NA	0.380	0.141	0.644	0.305	0.118	0.231
NA	NA	NA	0.373	0.167	0.566	0.361	0.132	0.295
NA	NA	NA	0.379	0.138	0.564	0.265	0.130	0.201
NA	NA	NA	0.356	0.141	0.541	0.258	0.113	0.201
NA	NA	NA	0.391	0.144	0.522	0.274	0.119	0.191
NA	NA	NA	0.418	0.168	0.596	0.316	0.148	0.217
NA	NA	NA	0.411	0.136	0.562	0.244	0.120	0.198
NA	NA	NA	0.409	0.145	0.580	0.266	0.127	0.208
NA	NA	NA	0.362	0.118	0.439	0.208	0.094	0.152
NA	NA	NA	0.333	0.094	0.219	0.193	0.078	0.146
NA	NA	NA	0.335	0.111	0.548	0.196	0.084	0.141
NA	NA	NA	0.392	0.128	0.515	0.246	0.112	0.191

DL_Ile	DL_Leu	L-Ser/ Asp	L-	[Asp]	[Glu]	[Ser]	[Ala]	[Val]	[Phe]
0.153	0.203		0.1	1238	718	184	618	358	168
0.166	0.235		0.2	527	314	121	361	175	94
0.115	0.162		0.2	1143	682	265	725	385	210
0.158	0.182		0.2	1496	872	262	755	452	204
0.118	0.171		0.2	1276	754	244	753	427	208
0.126	0.178		0.2	1016	611	223	551	357	145
0.198	0.251		0.2	857	434	198	543	267	141
0.144	0.213		0.2	1308	749	229	772	434	202
0.127	0.131		0.2	1371	842	270	786	435	228
0.224	0.186		0.3	1343	714	375	658	352	155
0.106	0.156		0.2	1965	1123	398	989	575	249
0.112	0.162		0.2	996	531	198	480	268	116
0.165	0.210		0.2	1231	786	274	760	399	196
0.089	0.143		0.2	1149	673	267	572	308	138
0.129	0.208		0.2	1876	1083	348	906	542	241
0.144	0.182		0.2	1327	761	277	795	395	201
0.124	0.166		0.2	1930	1050	409	1086	722	306
0.106	0.136		0.2	1749	1003	375	877	654	288
0.202	0.438		0.1	581	336	97	383	168	78
0.159	0.241		0.3	1019	750	295	915	464	234
0.190	0.252		0.2	681	394	143	449	235	138
0.116	0.166		0.2	1882	1035	380	898	559	263
0.140	0.185		0.2	1194	737	265	686	394	179
0.145	0.229		0.2	1039	589	181	614	375	164
0.171	0.242		0.2	895	553	201	618	334	168
0.121	0.162		0.1	1494	809	212	593	413	166
0.140	0.180		0.2	1144	712	225	689	397	207
0.159	0.204		0.2	1094	620	196	620	347	155
0.143	0.175		0.2	1017	642	284	845	363	184
0.182	0.237		0.2	1600	703	293	774	439	225
0.148	0.172		0.2	1236	724	298	883	442	193
0.160	0.205		0.1	1103	607	177	558	324	145
0.113	0.171		0.3	1582	1013	433	933	638	270
0.111	0.165		0.2	1614	923	372	757	472	214
0.160	0.149		0.3	1614	1240	624	1611	772	435
0.134	0.192		0.2	1399	900	265	663	412	195
0.142	0.186		0.2	1068	686	266	642	407	195
0.149	0.183		0.2	1355	877	344	952	518	259
0.099	0.154		0.2	1657	1031	375	857	539	222
0.161	0.214		0.2	844	511	174	512	286	125
0.170	0.236		0.2	974	612	185	555	308	143
0.129	0.177		0.1	1383	753	214	621	407	172
0.103	0.151		0.2	1294	759	231	594	372	148
0.161	0.239		0.2	699	414	130	414	235	119
0.160	0.212		0.2	1212	715	256	708	457	195
0.118	0.187		0.2	1147	660	203	569	333	146
0.150	0.217		0.2	1098	671	210	533	339	139
0.126	0.179		0.2	1412	879	340	803	475	230
0.129	0.159		0.2	909	507	171	442	252	118
0.097	0.123		0.2	2418	1513	642	1412	873	442
0.106	0.165		0.2	1597	936	322	792	456	204
0.136	0.171		0.2	994	545	178	533	300	123
0.109	0.157		0.1	886	510	155	442	221	98

0.161	0.208	0.2	738	459	154	545	261	147
0.156	0.204	0.2	937	580	172	552	307	133
0.160	0.208	0.2	1158	682	224	662	386	190
0.122	0.162	0.2	1391	840	363	889	486	218
0.090	0.131	0.2	1889	1084	356	853	506	198
0.164	0.285	0.1	766	452	120	404	197	92
0.119	0.138	0.3	1894	1316	572	1859	810	462
0.164	0.192	0.2	1024	624	239	655	373	165
0.130	0.152	0.3	1989	1394	592	1817	922	477
0.100	0.155	0.3	1598	1015	498	916	565	288
0.176	0.237	0.1	910	502	146	499	256	116
0.142	0.204	0.2	1021	622	222	592	337	151
0.226	0.261	0.2	558	343	110	423	182	103
0.084	0.125	0.2	1468	877	363	714	411	190
0.189	0.246	0.2	887	437	179	518	281	152
0.143	0.192	0.2	959	574	187	570	322	156
0.141	0.203	0.2	1728	834	301	788	490	231
0.138	0.178	0.1	1547	888	253	814	473	228
0.139	0.210	0.2	1093	693	235	627	344	166
0.154	0.195	0.2	916	541	203	545	260	131
0.131	0.164	0.2	1541	1018	407	1020	613	298
0.132	0.176	0.2	1080	683	253	833	436	245
0.206	0.269	0.2	646	327	116	380	191	99
0.170	0.218	0.2	737	415	159	513	310	146
0.169	0.212	0.2	1024	565	241	656	362	186
0.108	0.171	0.2	1298	764	242	663	429	208
0.145	0.211	0.3	682	469	212	403	233	148
0.164	0.216	0.2	745	424	140	438	232	117
0.177	0.240	0.2	1985	1107	463	1288	745	345
0.140	0.197	0.2	807	477	186	556	277	141
0.144	0.152	0.2	1043	630	202	771	374	186
0.436	0.465	0.4	392	237	149	400	119	70
0.110	0.141	0.2	2269	1323	447	993	646	295
0.108	0.154	0.2	1458	834	342	810	475	230
0.147	0.180	0.2	965	548	205	526	304	149
0.129	0.162	0.1	1548	892	246	643	400	170
0.148	0.213	0.2	1252	644	253	663	396	196
0.129	0.146	0.3	2030	1202	610	1651	807	419
0.161	0.201	0.2	1163	785	233	767	401	191
0.126	0.192	0.2	914	532	190	572	394	195
0.137	0.186	0.2	1117	691	226	696	386	199
0.101	0.131	0.2	1891	1181	499	1122	753	321
0.132	0.189	0.2	1010	590	177	617	350	164
0.159	0.273	0.2	754	431	149	438	204	112
0.150	0.255	0.2	915	512	166	522	260	127
0.149	0.195	0.2	1050	613	201	674	378	179
0.121	0.180	0.2	1014	560	188	610	308	150
0.109	0.146	0.2	2041	1281	540	1233	802	346
0.144	0.201	0.2	893	535	181	579	302	145
0.142	0.179	0.2	1216	703	329	941	426	230
0.096	0.130	0.2	1954	1192	539	1124	723	348
0.130	0.144	0.2	1263	752	242	721	422	201
0.121	0.171	0.2	1309	798	291	776	452	206
0.106	0.129	0.2	2281	1450	590	1339	944	450
0.120	0.199	0.2	1043	576	216	611	392	180
0.115	0.158	0.2	1184	605	252	704	499	174

0.182	0.262	0.2	852	395	149	465	227	125
0.133	0.179	0.2	1142	614	189	516	326	134
0.142	0.192	0.2	1148	700	234	682	368	160
0.150	0.182	0.2	1236	751	252	652	400	184
0.024	0.036	0.4	3942	1933	1794	1929	1761	567
0.137	0.183	0.2	1038	616	192	507	294	151
0.134	0.186	0.2	1032	595	238	619	382	170
0.098	0.127	0.2	2058	1268	485	1042	682	313
0.147	0.209	0.2	942	572	217	634	345	184
0.082	0.110	0.2	1905	1126	472	924	517	236
0.187	0.239	0.2	907	523	175	618	297	147
0.121	0.156	0.2	1463	902	345	805	473	220
0.130	0.165	0.2	767	437	141	407	212	96
0.183	0.216	0.2	1642	1045	386	1134	601	315
0.194	0.262	0.2	535	269	105	391	156	98
0.114	0.162	0.2	1220	720	290	647	441	214
0.120	0.190	0.2	1231	688	252	654	416	188
0.150	0.191	0.2	734	435	138	446	245	125
0.151	0.242	0.2	944	609	209	566	352	164
0.162	0.212	0.2	1167	741	259	680	376	194
0.129	0.207	0.3	1126	686	328	890	440	242
0.176	0.208	0.2	815	507	159	501	264	126
0.187	0.243	0.2	920	448	177	638	266	142
0.136	0.185	0.2	771	432	162	481	252	139
0.131	0.201	0.2	878	506	208	584	360	152
0.154	0.393	0.4	879	560	289	634	319	192
0.148	0.204	0.2	795	493	166	527	276	143
0.120	0.139	0.2	2139	1145	482	1043	665	320
0.005	0.015	0.6	3656	2466	2350	2470	1619	648
0.045	0.076	0.4	2244	1497	880	1738	1067	563
0.009	0.030	0.4	4428	2078	1772	1713	2262	454
0.068	0.143	0.3	1079	600	315	584	354	145
0.057	0.094	0.3	1632	970	541	1091	658	323
0.007	0.032	0.4	3514	1822	1334	1358	1139	433
0.011	0.032	0.3	3813	1624	1211	1249	1821	316
0.051	0.094	0.3	1700	1025	648	1172	580	293
0.011	0.042	0.4	1688	1009	735	799	606	206
0.117	0.224	0.2	1159	593	232	604	339	157
0.138	0.285	0.2	722	435	165	478	240	113
0.079	0.160	0.2	807	476	176	411	232	100
0.010	0.035	0.3	4594	1864	1529	1612	2084	452
0.059	0.126	0.2	1135	615	273	673	362	184
0.052	0.120	0.3	1214	741	355	702	397	174
0.043	0.100	0.3	1345	696	481	801	426	195
0.014	0.055	0.3	2000	1109	702	832	529	217
0.146	0.273	0.2	538	322	116	350	171	89
0.166	0.305	0.2	463	269	83	264	132	59
0.060	0.138	0.2	1038	570	250	595	333	148
0.076	0.153	0.3	912	530	271	619	331	153
0.069	0.105	0.3	1275	792	424	901	549	257
0.019	0.062	0.3	1078	600	380	546	295	124
0.051	0.114	0.3	1256	727	418	659	414	170
0.020	0.074	0.4	504	269	193	207	123	56
0.009	0.046	0.4	1197	740	506	574	327	129
0.013	0.044	0.4	2121	1193	792	1062	692	287
0.165	0.259	0.2	972	652	236	679	374	192

0.011	0.029	0.5	1265	863	659	715	389	174
0.024	0.069	0.3	1451	876	498	700	373	144
0.016	0.059	0.2	4091	1527	996	1557	1048	490
0.046	0.097	0.3	1266	791	488	739	456	195
0.076	0.146	0.2	736	481	188	418	227	101
0.060	0.147	0.2	1121	645	284	630	385	169
0.106	0.209	0.2	1891	854	383	885	544	249
0.129	0.190	0.3	1675	1122	535	1524	749	392
0.009	0.026	0.4	3201	1917	1360	1133	796	264
0.015	0.047	0.3	1498	926	545	659	400	142
0.047	0.107	0.2	1766	876	469	869	520	240
0.072	0.158	0.3	1196	762	389	669	402	168
0.036	0.107	0.2	1356	770	376	605	387	147
0.032	0.077	0.3	2224	1225	789	1030	655	297
0.045	0.096	0.2	2001	1161	578	838	582	215
0.012	0.039	0.3	1374	809	485	637	347	137
0.081	0.128	0.2	1524	720	366	646	430	163
0.165	0.271	0.2	727	477	162	468	269	108
0.027	0.047	0.3	1184	696	414	447	288	94
0.063	0.147	0.3	1034	602	303	580	348	159
0.146	0.278	0.2	1076	671	214	690	362	168
0.150	0.248	0.2	937	610	225	533	334	140
0.107	0.183	0.2	1765	1047	333	760	547	209
0.109	0.195	0.2	1422	910	322	742	493	217
0.118	0.206	0.2	1493	946	323	759	489	226
0.126	0.230	0.2	1251	777	226	589	352	135
0.101	0.176	0.2	1379	791	409	805	451	200
0.126	0.229	0.2	896	565	167	447	271	106
0.148	0.255	0.1	1372	811	215	637	397	168
0.113	0.206	0.2	973	558	226	518	297	134
0.130	0.240	0.2	1129	673	233	757	394	180
0.098	0.169	0.2	2247	1325	535	1114	847	345
0.088	0.164	0.2	1216	727	330	668	400	174
0.107	0.181	0.2	1618	937	378	843	496	214
0.149	0.261	0.2	935	636	202	628	365	148
0.193	0.314	0.1	524	344	84	304	159	64
0.108	0.182	0.2	1035	682	312	837	412	195
0.128	0.218	0.2	1194	768	329	786	462	199
0.112	0.196	0.1	1456	896	243	590	407	148
0.098	0.179	0.2	1759	1029	434	885	578	270
0.065	0.184	0.2	1366	806	360	641	359	142
0.127	0.226	0.2	1297	828	318	793	482	196
0.153	0.272	0.2	752	502	142	499	261	125
0.122	0.250	0.2	966	574	197	491	298	130
0.096	0.194	0.2	1713	1066	454	962	658	264
0.091	0.174	0.2	1257	660	324	658	356	165
0.182	0.324	0.2	705	455	147	451	242	109
0.172	0.299	0.2	660	404	132	375	217	90
0.102	0.210	0.2	1192	677	231	536	367	161
0.067	0.125	0.3	1591	952	477	757	482	197
0.147	0.265	0.2	1054	629	209	558	365	155
0.140	0.233	0.1	2729	909	375	918	567	254
0.165	0.289	0.2	794	497	139	467	267	107
0.150	0.278	0.2	1095	593	239	550	341	138
0.061	0.133	0.3	1262	788	385	572	373	143
0.127	0.190	0.2	1189	664	333	753	360	181

0.157	0.265	0.2	712	473	155	491	267	116
0.090	0.195	0.2	888	474	186	383	206	86
0.133	0.249	0.1	1003	587	156	459	273	111
0.017	0.042	0.3	1731	1073	596	647	396	144
0.145	0.244	0.1	927	560	137	424	241	88
0.103	0.183	0.2	1687	1038	400	856	575	269
0.058	0.125	0.3	1729	1069	529	796	533	234
0.161	0.292	0.2	991	545	188	551	324	137
0.050	0.103	0.2	1547	891	438	612	417	142
0.159	0.271	0.2	1104	631	261	633	395	181
0.097	0.165	0.2	2080	1293	542	1084	784	313
0.126	0.222	0.2	947	595	202	480	284	122
0.196	0.360	0.1	561	249	88	313	132	70
0.187	0.342	0.1	509	262	79	287	139	64
0.153	0.227	0.2	1518	891	346	763	447	225
0.172	0.294	0.2	566	297	102	320	159	82
0.167	0.202	0.186	915.793	589.171	192.606	551.666	307.682	163.524
0.142	0.175	0.144	1108.885	536.887	181.345	549.925	311.177	143.974
0.137	0.183	0.165	1130.790	643.892	203.767	583.468	318.956	144.647
0.190	0.213	0.202	991.715	637.451	225.069	653.154	359.031	171.286
0.138	0.167	0.155	985.833	591.156	169.442	516.509	279.500	138.683
0.152	0.189	0.162	913.070	510.493	165.745	486.301	281.843	132.431
0.105	0.115	0.257	1737.834	1146.831	471.186	1022.815	709.684	319.490
0.095	0.114	0.465	1814.983	1323.452	770.832	1074.771	738.452	347.286
0.108	0.124	0.225	1498.538	944.203	390.612	821.554	469.043	236.874
0.131	0.158	0.191	1569.032	916.693	325.953	837.480	502.439	226.061

[Ile]	[Leu]	[total]	L Asp	D Asp	L Glu	D Glu	L Ser	D Ser
159	245	3687	970.7	417.8	699.7	105.8	130.9	75.6
75	124	1790	449.6	188.9	329.5	51.6	105.2	41.4
171	263	3845	957.2	343.1	689.3	86.7	192.3	109.7
229	308	4578	1199.9	506.1	872.5	121.5	191.2	107.5
193	287	4142	1114.7	442.1	812.2	107.6	194.1	103.9
159	249	3308	875.3	329.1	638.2	86.1	174.7	89.5
128	200	2770	684.6	320.6	433.7	75.1	158.7	74
186	277	4157	1069.5	408.7	738.7	107.6	170.3	88.4
222	341	4494	1195	464.3	902.2	117.3	213.5	113.5
188	255	4039	1134.9	380	704.7	100.8	330.5	92.2
265	409	5973	1674.7	622.9	1164.7	148.7	319.4	146.5
120	166	2875	865.3	308.6	555.4	71	151.2	82.6
189	330	4166	1037.1	408.5	801.1	122.5	197.9	124.2
135	208	3450	1036.4	353.8	738.4	76.3	204.3	118.6
254	397	5648	1673.7	661.3	1177.7	170.2	271.7	161.3
184	287	4229	1167.6	445.6	809.7	115.5	204.9	132.4
338	488	6328	1601.4	748.8	1121.9	156.3	333.1	164.4
326	439	5712	1522.1	593.5	1079.3	133.9	319.6	134.3
74	122	1840	720.5	278	490.2	88.2	104.1	63
191	321	4188	838.1	353.5	755.3	121.3	213.8	130.7
110	171	2321	533.4	230.5	380.8	61.8	106.7	53.6
264	417	5698	1533.2	644.5	1058.4	139.1	279.9	159.2
170	284	3910	1005.4	388.7	757.7	103.4	198.8	110.9
177	238	3378	821.6	337.1	568.8	88.2	131.1	71
160	263	3191	770.5	325.4	581.9	95.2	160.3	86
210	271	4169	1268.6	544.8	865.6	116.1	168.6	88.6
191	302	3867	934.1	361.8	710.8	96.4	167.3	87.6
157	237	3425	919.3	386.9	646.8	93.1	150.1	84
134	232	3701	855	297.6	635.5	92.1	198.4	123.5
212	324	4570	1257.9	643.3	716	119.2	227.1	120.9
188	286	4249	1052	391.1	744	101	224.7	123
145	198	3256	856.5	383	590.9	91.3	122.6	75.8
280	486	5637	1359.9	535.7	1076.8	137.2	362.9	156.4
243	354	4949	1358.2	496.7	939.5	120.6	279.7	148.2
309	534	7139	1504.9	609.6	1426.9	198.1	492.8	324.4
205	316	4356	1188.9	441.2	921.2	127.8	192.7	116.4
191	297	3751	915.9	365.9	723.2	99.9	214	105.1
223	343	4872	1117.9	468.3	899.4	126.6	250.2	152.4
239	378	5299	1489.1	524.7	1120.6	132.6	294.7	160.5
126	192	2769	703.5	278.7	514.1	81.1	129.1	73.3
138	226	3140	835	347.5	639.1	104	138.8	85.7
205	252	4007	1199.8	500.1	813.8	111.8	169.7	92.9
161	240	3799	1144.3	448	828.9	105.1	181.2	103.3
105	151	2266	574	242.8	418.3	65.4	100.6	51.5
219	318	4080	1029.6	440.3	747.3	119.4	202	107.9
145	214	3418	974.4	387.6	689.8	93.6	153.5	87.9
161	234	3385	928.9	360.7	685.3	102	155.3	91.4
230	378	4747	1261	469.5	950.4	126.3	266.1	150.7
128	172	2700	988.5	399	686.2	87.8	169.5	92.1
411	681	8392	2313.7	883.5	1791.1	209.4	558.1	290.5
200	326	4832	1456.6	522.9	1026.7	133	256.7	142.1
131	189	2993	934.4	380.9	627.7	93.1	149.3	86.2
100	140	2552	1920.8	704.2	1333.1	179.6	287.6	173.3

111	177	2591	683.2	281.6	523	76.1	128.7	72.7
143	206	3030	768.6	308.7	580.1	86.9	123.9	74.4
191	299	3792	992.9	428.7	724.2	112.5	176.8	98.2
215	371	4773	1209.4	432.9	874.6	116.9	282.2	145.8
213	327	5427	1660	599.2	1164.7	132.3	266.9	159.2
78	136	2245	641.6	261.9	460.1	72.7	86.4	55
286	518	7718	1582.3	566.6	1337.9	155.4	411.2	238.2
178	282	3540	968	396.5	723.6	107.5	206.6	111.6
356	623	8169	1655.6	650.8	1429.6	187.2	429.3	256.6
291	477	5648	1431.1	438.8	1069.4	118.4	382.8	199.5
104	166	2699	782.5	286.9	503.6	86.7	109.5	62.1
131	236	3312	884.8	300.1	639.4	83	165.7	92.5
73	129	1921	509	211.3	376.6	65.7	91.9	49.9
194	312	4528	1300.4	405.4	924.5	94.6	273.5	148
139	209	2802	756.1	362.4	467	83.7	149.5	76.7
163	225	3156	799.5	324	588.5	84.7	135.3	83.3
246	362	4981	1476.3	698.8	916.9	133	246.8	132.5
215	303	4720	1220.2	463.9	845.9	120.2	176.8	98.2
164	259	3580	937.5	336.6	710.6	97.6	170.5	103
114	171	2881	804.9	257.1	547.9	79.2	157	78.1
277	450	5624	1358.1	529.8	1095.8	150.9	326	173.1
176	290	3997	933.5	334.1	706.9	95.1	194.4	102.1
78	124	1962	543.3	250.5	343.7	58.1	91.1	51.9
136	188	2604	657.3	305.9	463.4	78.7	134.2	74.2
161	261	3456	639.1	268.3	434.6	65.6	147.7	65.7
205	288	4096	1129.8	448.3	821.9	107.3	191.1	103
114	191	2452	605.3	188.4	490.3	55.3	206.6	40.1
104	155	2356	641.4	261.6	449	65.2	113.1	56.6
371	546	6850	1453.3	694.1	1030.4	167.3	335.7	165.7
117	198	2759	709.3	264.9	503.8	71.7	159.5	64.6
161	237	3603	935.2	339.3	681.9	87.5	160.4	86.4
50	95	1511	300.4	153.2	225.2	49	124.2	48.4
342	534	6849	1964.8	763.7	1415.9	174.6	350	187.2
224	346	4720	1275	453.1	881.5	107.6	268.9	136.6
152	205	3055	853.6	331.6	590.8	82.4	160.5	91.4
178	277	4354	1300	541.8	938	122.5	186.2	106.3
186	304	3894	1029.8	427.5	650.4	99	194	101
319	524	7562	1743	689.9	1275.3	165	460.6	270
177	303	4020	930.4	337.7	755.1	101	164.7	89.5
194	230	3223	739.9	282.6	521.2	74.4	142.8	69.9
171	263	3749	962.1	356.2	723.7	92.1	171.2	95.8
337	547	6653	1645.8	603.1	1256.4	148.4	409	184.3
154	212	3275	830.2	329.2	594.1	82.8	129.5	73.8
92	135	2316	598.6	247	416.7	66.2	107.8	59.7
123	183	2807	747.1	293.7	508.4	74.7	120.3	68.9
166	243	3504	867.9	337.9	617.8	85.5	146.1	84.8
132	203	3164	858.4	330.2	575.3	81.2	144.2	76.3
368	591	7202	1699.8	619.8	1306.4	149.2	413.8	200
121	194	2950	743	288.2	541.6	75.9	140.5	68.1
174	269	4288	1084.1	394.6	739.4	115.6	243.9	156.2
337	565	6782	1741	612.2	1288.1	147.4	433.9	214.7
194	285	4080	1083.4	415.9	787.1	106.2	192.9	94.7
197	301	4330	1142.8	417.7	838.9	112.2	218	128.6
461	720	8235	670.3	251.1	524.5	61.5	161.5	76.7
178	238	3434	876.3	355.7	599.5	81.1	170.7	84.7
210	278	3906	1042.4	485.4	685.3	95.7	216.1	109.1

98	165	2475	728	265	396.1	64.2	112.1	61.6
159	217	3297	940.5	379.3	624.8	84.7	144.8	73.7
160	251	3704	983.1	368.6	717	107.4	176.9	99
188	281	3943	979.3	394.7	725.5	110.1	177.4	103
670	861	13458	4213.3	637.7	2285.7	93	1785.8	422.3
141	200	3138	917.4	359.4	669.1	88.5	150.2	86.3
173	284	3494	876.4	347	621.9	83.9	197.1	84.7
328	520	6694	1842	651.8	1391.8	144.7	395.2	192.1
167	247	3310	814.6	329.8	605.8	89.2	169.6	94.3
228	349	5757	1624	520.6	1149.7	118.7	337.7	193.8
126	210	3003	752.7	303.5	523.7	85.1	130.4	73.1
224	366	4799	1254.3	471.9	943.9	120.4	260.8	146.7
86	136	2283	691.8	242.7	466.1	65.6	107	65.1
265	444	5832	1209.5	488	932.1	148.4	247.2	152.1
63	105	1722	446.6	204.2	278.7	48.7	82.5	45.3
232	333	4099	1028.9	383.5	739.7	93.9	233.7	102.2
190	292	3911	1068.1	429.8	740.1	97.4	202.8	103.7
110	173	2407	619.9	256.6	456.3	63.2	109.4	55.8
177	273	3294	778.9	320.3	620.9	88.5	165.3	77.8
188	291	3895	978.8	364.5	739	114.4	183.3	114.5
205	329	4245	919.4	380.7	695.8	96	250	128.5
115	174	2661	688.6	268.1	511.9	83.3	115.1	71
101	143	2835	830.6	298.9	464.4	85.4	134.8	82.6
116	173	2526	629.8	257.2	436	60.7	121.8	64.4
159	248	3096	2209	1045.8	1628.1	249.2	516.5	254.4
180	312	3364	749.9	251.8	570.5	67.4	269.7	60
115	199	2713	646.9	256.4	490.6	69.6	123.8	65.1
335	501	6631	1879.2	767.6	1257.8	159.5	399.7	196.9
678	920	14807	32750.5	2759.3	23330.2	618.8	20562.7	2266
529	762	9280	18526.2	3658.4	13627.5	1173.3	6785.6	1914.7
910	959	14577	50747.3	3130.7	24654.4	627.9	20343.8	1218.9
133	217	3426	8578.9	2193.7	5452	540.7	2154.9	987.6
319	459	5993	12381.3	2519.5	8061.7	793.9	3920.8	1018.7
542	717	10860	35044.4	2711.2	18616	958.1	13070.8	1265
699	692	11424	38169.9	4340.6	17520.4	584.6	11850.3	1648.1
257	403	6077	13371.1	2871.8	8935.9	857.2	4348.4	1845.2
257	365	5665	16486.3	1791.8	10526.7	403.3	7118.5	839.4
158	244	3486	8081.9	3045.3	4955.8	740.3	1670.9	558.7
104	174	2431	5172.6	1762.8	3566.3	612.5	1014.9	573.9
100	149	2451	6066.8	1671.5	4102.7	462.8	1175.6	513.1
895	905	13934	48923.9	4834.1	21147	663.4	16620.3	1266.6
172	266	3681	9789.8	2011.6	5771.4	629.2	2439.8	402.1
155	248	3985	10694.3	2584.8	7418.5	691.5	2705.4	1175.1
174	282	4401	12327.9	2171.4	6952.6	557.8	3760.7	1423.9
249	359	5997	20172.6	2927.3	12242.2	572.8	6557.8	1548.6
84	128	1798	4110.9	1182.1	2740.7	427.9	843.3	295.8
65	97	1433	3372.3	1080.4	2230	353.3	609.9	192.2
151	218	3303	9079.4	1970.9	5484.9	588.9	2106.4	559.7
143	217	3175	7048.8	1577.9	4533.1	478.2	1923	635.2
248	375	4821	10148.3	2035.5	6932.3	631.9	3240.5	813
134	210	3366	9300.5	1800.1	5866.1	307.8	3059.4	850.4
187	294	4125	11458	2901.9	7652.5	659.7	3355.9	1427.3
59	81	1492	5204	556.3	2949.7	130.1	1997.4	205
137	218	3827	11203	1128.5	7337.5	286.8	4673.8	536.1
319	493	6959	18692.5	2375.7	11343.2	502.5	6659	1208.7
171	308	3584	7176.4	2724.9	5718.2	920.3	1560.9	838.5

171	277	4514	11961.6	723.6	8440.1	212	6262.8	344.4
156	263	4461	12403.5	2384.9	8550.1	376.1	3937.1	1141.8
469	657	10836	29891.1	8052.8	13041.7	1121.2	7268.1	1966.1
197	322	4454	10638.1	2264.3	7455.6	604.7	3516	1452.8
93	151	2395	5872.3	1861.5	4516	545.1	1309.3	663.6
165	243	3642	8882.6	2222.4	5742.6	652.1	1962	850.1
247	367	5419	14538.4	5423.6	7814.6	1205.7	2860	1179.6
289	480	6766	13312.9	4309.4	10335.5	1470.8	3615.7	2017.1
381	535	9587	32817.3	3354.4	21144.3	520.9	13612.2	1755.2
182	255	4608	13441.6	2625	9510	422.8	4128.9	1711.3
227	337	5306	14021.7	3547.6	7895.9	823.4	3269.7	1400.1
166	301	4054	9302.1	2631.8	6896.6	710.7	2519.7	1364.3
158	253	4051	11631.5	2399.9	7366.5	597.5	2802.7	1090.8
298	472	6992	19061.3	3988.8	11908.3	788.7	5839.6	2341.7
264	405	6043	16202.2	4009.1	10877.5	844	3972.3	1862.9
155	221	4166	12657.4	1964.5	8229.6	371.5	3912.9	1251.2
176	273	4297	12165.8	3689.6	6795.7	693.7	2574.5	1229.2
110	190	2511	5639.3	2097.5	4332.6	740.1	1071.8	651.8
122	179	3425	10679.1	1616.1	6936.8	293.8	3266.9	1034
146	238	3411	8102	2089.2	5354.9	582.3	2108	880.9
144	244	3569	8446.2	3131.1	6208.7	1011.3	1516.3	786.9
144	243	3168	7150.5	2541.9	5489.8	821.6	1499.7	828.3
266	412	5340	13606.4	5166.4	9836.7	1298.2	2291.3	1254.2
229	384	4719	11169.5	3944.2	8506.5	1167	2169.5	1254.2
237	372	4846	11978.5	4172.2	8980	1254.3	2194.7	1302.3
154	249	3731	9765.8	3798.7	7346.3	1075.2	1598.8	847.3
195	320	4550	11217.4	3784.9	7688.6	913.2	2775.7	1667.9
112	184	2747	6920.6	2422.1	5189	706.9	1075.6	661.5
181	273	4054	9865.5	3989.9	7122.5	1073.8	1303	871.7
139	219	3063	7429.5	2438.9	5009.5	647.7	1471.1	817.3
147	256	3767	8713.5	3141.9	6114.7	950.9	1487.3	955.2
400	647	7460	16630	6233	12022.1	1456.9	3716.8	1727.8
175	287	3978	9638.5	2844.4	6696	771.7	2224.5	1163.7
205	344	5035	12727.6	4238.7	8692.2	1131.4	2455.2	1511
145	252	3311	7139.9	2783.7	5795	960.2	1347.4	799.1
68	110	1657	4004.4	1579.5	3092.5	567.3	554.2	342.5
155	259	3888	7832.4	2680.2	6076.3	857.3	1914.5	1259.4
184	333	4255	9345.4	3293	7115.9	1014.1	2109.6	1377
191	298	4229	11482.4	4402	8629.6	1148.7	1662.7	990.2
279	466	5700	14314.8	4613.1	9819.2	1254.4	3033.3	1637.3
152	248	4073	11078.6	3339.1	7705.9	801.9	2694.1	1110.4
205	337	4454	10324.9	3727	7867.9	1100	2261.4	1180.7
116	191	2589	5946	2348.6	4719.3	810.5	945.6	624
125	213	2994	7980.8	2727.3	5547.7	809.1	1330.3	850
288	492	5898	13699.1	4926.8	10239.6	1349.2	3188.6	1745.3
154	265	3839	10144.6	3483.8	6409.7	741.5	2263.8	1245.1
109	183	2399	5768.8	2354.1	4451.5	795.6	1031.6	662
91	151	2121	4902.1	1942.7	3631	559.5	891.2	474
163	260	3587	9279.7	3231.5	6322	785.9	1599.5	826.8
200	339	4994	12917.8	3753.7	9152.2	827.3	3298.6	1701.9
164	281	3416	7770.1	3168.1	5679.1	849.6	1408.6	763.9
263	417	6431	18085	10021.4	8115.8	1243.4	2397	1462
106	180	2557	6250	2353.5	4581.8	805.8	978.8	532.1
138	233	3327	8780.9	3140.3	5646.1	811.2	1699	905.5
161	279	3963	10436.2	2981.2	7632.4	742.7	2946.1	1143.3
150	257	3887	9188.9	2868.7	5896.7	840.3	2048.6	1327.2

105	182	2502	5559.7	2097.9	4380.5	709.7	1023	647.4
85	145	2453	7146.5	2185	4445.1	538.9	1263.7	695.5
126	191	2907	8077.6	3133.9	5716.5	842.4	1062.4	679.8
196	277	5060	14643.3	2529.5	10252.6	395.9	4395.7	1521.4
95	158	2629	6749.8	2585.4	4915	730.3	835.9	546.4
291	463	5578	13886.7	4950.1	10343.6	1242.3	2860.3	1610
250	392	5532	13516.2	3931.5	9889.2	900.4	3607.9	1726.5
145	258	3140	7924.4	2945.8	5136.6	844.7	1321.4	745.4
194	280	4520	12796.7	3713.2	8810.7	699.6	3017.3	1659.2
182	336	3723	8276.7	3604.1	5847.2	940.1	1898.9	905.5
362	578	7036	15336.6	5639.2	11635.3	1407.3	3660.3	1807.8
126	211	2968	7617.7	2646.4	5662.6	781.9	1477	715.5
58	102	1572	2782.6	1072.3	1445.4	263.1	397.6	210.6
59	102	1502	3876.7	1362.7	2290	410.7	515.1	303.3
226	359	4774	11596.2	4403.7	8231	1163.5	2217.6	1428.4
74	124	1723	4312.5	1610.6	2660.8	444.2	678.5	384
142.204	219.345	3081.992	1684.867	637.367	1306.733	181.367	309.033	176.233
140.907	213.776	3186.876	1134.550	404.400	652.850	91.900	163.250	88.350
148.661	220.292	3394.474	971.150	380.150	672.850	96.600	159.950	83.550
152.813	247.570	3438.090	833.150	347.900	650.250	108.950	167.900	100.150
126.320	195.725	3003.169	833.350	342.850	620.900	84.350	129.450	72.700
131.960	186.033	2807.876	1663.133	680.867	1139.467	166.867	267.100	156.900
338.566	507.676	6254.083	1568.600	567.350	1260.800	148.650	402.350	176.750
359.222	626.168	7055.166	4327.100	1455.100	3842.900	370.500	2008.800	440.100
222.483	370.418	4953.724	1305.550	437.700	988.350	110.200	293.600	160.850
225.480	366.376	4969.514	1344.800	527.500	969.750	124.300	256.800	132.200

Gly	L Ala	L hArg	D Ala	L Val	D Val	L Phe	L Ile	D Phe
488	541.5	224.3	151.6	348.6	52.4	153.9	154.5	34.1
394.9	344.3	242.5	93.6	187.7	24	97.8	77.8	16.6
631.6	671.7	227.6	153.5	398.7	39.8	204.4	175	34.8
708.9	678.6	228.1	182.5	460.7	54.9	193.4	225.4	39.4
644.2	747	244	171.8	472.8	48.1	211.7	211	41.5
469.5	506.3	237.2	146.6	380.5	42.4	146	167	25.4
622.5	478.9	234.5	158	267.7	45.3	135.7	125.5	30.2
690.6	688.9	226	182.9	437.4	53.1	186.1	183.4	42.1
832.6	767.2	242.1	183.8	471	55.5	233.3	238.3	42.4
620.4	614.9	225.6	127.1	362.1	35.3	149.5	173	25.2
788	939.3	233.9	217.6	612.2	59.8	245.1	280.2	46.3
498.4	463.4	235.8	102.2	288.7	26.7	117	127.4	20.3
581.8	711.4	234.9	181.8	415.3	52.9	192.2	190.7	38
580.8	574.5	242	118.1	347	25.1	147.9	150.2	18.5
987.6	899.7	248.9	227.4	607	67.7	250.6	280.1	49.6
757.7	758.1	243.1	207.9	428	52.7	199.1	195.6	45.4
1295.1	1047.4	243.5	274.8	794.2	85.2	312.3	365.6	59.8
1064.2	856.6	241.9	203.6	721.6	69.9	301.3	356.6	47.6
449.6	495.2	344	163.7	249.8	39.6	106.8	106.5	28.2
807.5	823.4	233.9	246.3	475	67.3	229.3	192.6	44.9
449.7	393.8	224.5	109.7	229.5	34.2	126	104.2	29.1
944.7	831.2	231.4	207.9	583.4	63	261.1	274	43
634.8	643.4	233.6	158.4	413.1	47.6	178.6	174	30.8
509.4	537.6	223	147.3	370.7	46.9	148.4	172.7	34.2
627.5	582.6	245	174.5	359	50.3	166.9	167.4	38.5
624.2	578.7	242.7	140.8	453.7	47.9	168.5	227.3	32.7
656.6	632.7	226.6	147.8	400.6	49.6	197.9	189.8	36.4
556.6	601.9	238.8	137.9	368.8	45.9	154.2	162.1	30.7
672.3	765	226.7	192.3	368.2	43.6	177	133.2	31.9
1011.3	704.8	237.6	214.4	450.3	70.7	219.2	212.9	48.3
523.2	845.8	233.5	184.7	462.3	53.9	186.8	191	38.4
510.4	484.5	224.7	142.4	318.2	45.4	135	140.4	27.7
844.4	901.1	239.6	216.4	691.2	73.5	279.9	301.9	43.9
741.7	688.4	229.8	181.1	495.6	47	209.3	251.2	36.3
1708.1	1613.6	262.1	498	904.1	107.6	467.5	349.3	102.9
659.8	609.5	233	163	425.2	55.1	191.9	210.2	34.9
633.8	609.8	240.1	160.9	436.1	52.3	197.6	201	35.9
878.8	873.4	234.1	241.5	534.4	71.5	252.6	227.2	51.1
822.6	853.8	243	187.5	603.1	52.2	230.8	263.9	39.4
453.9	459	232.8	136.4	292.8	39.9	120.1	126.1	25.2
547.4	516.4	242.9	157.4	327	46.8	141.4	143.2	31.9
638.1	615	245.8	148.3	450.7	49.9	177.5	222.9	34.4
541.4	593.2	246.2	138	417.7	40.2	154.6	180.3	27.6
377.4	382.2	233.8	102.2	240	34.4	117.3	105.8	21.3
741.9	664.7	242.5	193.3	488.5	65.4	194.9	229.2	41.7
523.5	541.9	237.4	133.4	353.1	41.6	144.2	154.4	29.1
517.6	495.3	234.8	130.6	352.9	44.9	137.6	164.2	26.1
765.4	793.6	245.1	190.7	524.3	57.7	238.6	249.8	43.4
566.7	546.5	305.4	129	348.9	36.3	145.8	173.3	34.4
1452.4	1559.5	264.4	307.1	1079.3	74.5	513	495	71.4
690.6	790.8	247.9	190.5	516.5	49.2	218	223.8	34.4
498.1	554.9	264.7	150.3	355.5	42	133	152.6	30.4
630.9	1049.7	592.8	260	588.1	66.9	242.2	267	49.2

500.3	570.4	261.3	141.6	301	39.4	159.3	124.4	32.7
396.9	501.1	230	133.4	313	40.4	127.3	142.2	25.1
729.8	626.4	245.5	185.7	413.4	60.1	191.2	202.3	42.6
708.2	854.4	236.1	195.5	520.1	53.1	221	226.5	36.5
740.7	853.3	239.2	167.4	560.8	44.9	202.9	233.3	33.8
348.2	372.6	235.9	103.9	204	28.2	87.6	79.1	21.1
1317.8	1784.3	226.9	324.7	841.2	78.2	450.3	289.6	74.4
705.5	689.4	266.5	182.8	446.3	51.2	179.4	204.1	40.9
1604	1702.5	231.9	404	964.6	104.9	464.4	365	88.9
769.8	896.8	234	175.1	609.1	51.7	299.2	309.4	38.1
374.9	465.6	235.1	120.5	263	38.3	116.7	103.8	19.7
475.1	559.9	232.2	127.9	352.4	38.5	147.9	132.8	26.9
373.9	416.2	258.1	130	201.1	33.8	106.5	77.3	25.9
629.1	709.8	232.4	119.8	444.7	32.4	196.6	207.8	23.6
743.9	499	252.1	154.4	305.6	48.2	156	146.9	35
675.6	527.8	234.4	139.9	335.6	42.1	151	166.7	32.3
1096.5	783.5	251.8	208.7	549.5	67.5	242.3	271.8	49
658.2	703.2	217.7	182.4	455.4	59.1	206.1	205.5	42.4
549.5	591.6	233.2	139.7	359	42.1	162.6	168.2	30.7
489.3	512.2	231.9	120	266.9	34.6	131.7	114.3	20.1
935.6	986.5	245	262.4	670.9	80.1	306.4	299.8	59.1
664.6	797.4	234.7	180.4	463.2	48.7	243.1	182.8	44.1
377.1	358.7	245.6	107.6	203	32	100.4	79.3	21.1
584.5	517.3	261.4	152.6	357.7	47	155.2	151.9	36.2
568.1	461.6	177.2	119.6	282.8	37.7	137.7	122.3	26.7
702.2	658	243.2	147.6	475.3	46.1	214.9	224.9	38.6
419.7	365	232.7	104.4	238	33	148.3	116.1	23.7
381	418	242.3	112.7	249.4	31.7	119.6	107.9	22.5
1094	1094.7	216.4	298.8	713.3	92.8	304.9	341.4	68.9
402.5	546.5	241.3	124.4	300.6	33.8	143.4	124	26.3
533.2	788.5	244.4	153.1	413.5	43.8	194.8	172	32.5
359	337.5	231.5	125	107.5	30.6	60.5	39.9	20.8
1158	969.1	240.5	225.5	707.4	69.3	302.5	370.5	52.3
634.2	790.3	237.1	170.3	514.9	48.6	234.2	239.7	39
585.9	504.5	245.6	141.1	326.1	47.7	152.7	162.5	30.1
667.4	612.1	237.9	152.2	428	47.9	167.6	187.9	34.7
733.9	598.9	232.8	172.6	407.5	53.6	190.2	188.9	38.1
1676	1582.7	239.7	396.6	873.4	93.2	418.9	338.4	83.8
528.7	677.9	218	158.2	385.7	51	174.6	165.8	33.9
515.6	515.1	223.7	124.9	399.2	41.9	183.8	192.9	34.3
637.1	669.6	236.1	151.5	409	46.9	197.4	177.1	38
1027	1094.1	237.8	240.2	821.4	73.8	330.9	364.4	51
465.2	573.7	229.5	134.6	360.6	40.9	156.1	155.7	32.5
418	387.8	224.2	103.7	201.7	27.5	105.7	88.9	19.3
459	468.6	227.6	125	265	30.7	119.9	121.9	24.1
580.6	626	229.6	147.3	385.7	48.8	170	166.3	35.2
477	589.4	234.5	125.8	322.1	38.5	149.7	137.5	26
1091.1	1148	227.3	253.7	833.7	77.7	340.6	377.3	52.4
452.4	540	230.9	128.9	309.3	39	140.8	122.1	26.6
963.8	892.7	243.2	251.3	457.3	61.1	232.4	185.3	46.8
1068.1	1115.8	240.8	237.6	805.1	65.8	367.7	369.9	51.1
639.3	694.3	237.5	162.3	451.6	50	202	203.6	36.2
668.5	744.1	238.4	180.6	485	53.3	207.8	209.8	38.1
456.8	448	80.8	92.9	350.4	30.9	158.1	168.5	23.9
696.9	581.9	236.2	139.2	420.2	42.6	178.7	187.7	34.3
779	735.6	258	172.4	592.6	50.9	185.8	243	38.4

425.9	415.9	233.2	126.4	230.3	34.2	121.1	96.3	25.2
452.5	487.6	231.2	109.3	338.5	38.2	128.8	162.1	25.9
562.8	632.5	235.4	170.1	384.8	48.5	158.9	164.7	29
642.5	564.1	222.4	160.4	389.8	54.6	171.7	181.6	32.6
4364.7	2249.6	246.1	123.8	2137.3	30.2	675.5	805	22.3
582.4	500.3	246.1	123.2	323.2	38.3	156	152.4	29.8
650	590.6	237.1	143.6	407.5	45.7	170.4	180.4	31
1101.9	1058	242.4	204.9	767.8	58.9	329.4	361.5	49.5
639.1	613.6	242.9	156.9	372.5	47	187.2	176.3	36.2
879.1	883.3	225.2	157.1	539.8	42.5	234.1	237.1	31.9
514.5	563.1	232.8	155.7	304.5	40.9	141.6	123.5	29.6
770.9	767.7	236	182.2	506.8	51.8	221.3	236.3	38.4
382.1	399.8	243.6	96.2	232	26.5	101.2	93	16
889.1	923.1	206.8	249.5	539	82	268.1	231.6	57.3
368	373.9	243.2	101.5	166	23.9	98	63.8	21.3
781.6	602.5	231.5	146.7	465	45.9	212	241.3	35.4
736.7	638.5	243.4	157.7	456.4	49.9	191.1	206	38.2
467.9	427.5	238.7	104.6	260.8	31.3	126.2	114.4	23.1
623	518.9	232.8	139.6	365.6	44.6	158.1	179.4	32.5
652.7	616.1	230.3	166.4	380.2	53	187.6	186.4	35.6
908	821.1	230.9	206	454.8	53.5	235.1	209.9	44
425.2	455.9	234.8	132.3	270	40.2	123.3	114.6	25.1
574.9	598.6	245.5	185.1	284	42.4	143	104	30.9
465.3	437.9	230.1	115.7	260.1	30.2	135.3	118	24.3
1139	1688.6	741.3	475.8	1200.9	132.5	461.3	522.4	103.6
677.9	590.4	228	132	333.9	29.6	192.6	178	26.4
468.4	482.6	227.2	115.9	278.9	34.4	135.7	113.8	26.3
1463.9	1039.4	247.5	251.4	743.2	80	337.9	370	58.2
36251.2	23334.7	1942.7	660.2	15595.4	132.2	6134.6	6559.4	155.3
12321.8	15142.8	1977.3	2037.7	10142.8	410.2	5190.9	5000.1	376.9
57433	19971.8	2433.3	873	27228.3	294.9	5282.5	10972	245
4592.7	4935	1997.4	898.4	3353	179.5	1279.9	1242.2	166.1
7488.4	8767.1	1826.3	1198.1	5738.6	267.6	2749.9	2755.9	201.6
23161.5	13787.1	2148.8	804.2	12103.5	137.9	4501.4	5790.1	146.2
43074.1	13222.9	2230	702.1	20059.6	249.2	3357.1	7707.1	161.9
8885.2	9859.9	1911.3	1338.5	5334.1	204.2	2569	2338.2	227.6
12064.1	8108.6	2165.5	538.8	6473.7	88.3	2171.6	2753.8	55.7
4934.3	4468.6	1920.8	1332	2932.6	324.6	1272.4	1357.8	234.5
3309.6	3307.3	1922.4	1285.3	2045.4	263.3	846.1	880.1	237.5
2291.5	3262.1	1917.8	681.8	2082.2	139.9	838.4	885	121.8
55792.6	17876.3	2340.3	987.6	24080.1	304.3	5099.4	10370.3	190.3
4648.7	5868	2080.1	1129.7	3545.5	222.5	1717.2	1691.9	195.8
4893.4	6700	2187.7	974.4	4165.2	178.4	1719.2	1607	181.6
6526.3	7635.1	2156.7	1005	4441.5	156.6	1943.2	1802	164.9
8574.6	8803.7	2310.5	810.4	6002.6	109.4	2389	2832.1	118.6
2006.7	2618.1	1966.6	822.8	1492.7	185.2	719.1	718.6	152.7
1411.1	1895.4	1923.1	647.1	1125.7	141.9	466.2	532.8	104.9
3531.4	5337.5	2129.3	997.2	3361.6	185.8	1406.3	1512.4	167.9
4500.1	4971.1	1891.4	879.5	2947.4	182	1291.7	1254.7	153.3
5276.9	7475.3	1910.9	1129.8	5016.2	232.8	2271.3	2218.1	182.8
5239.9	5200.2	2059.6	424.7	2979.4	62	1208.7	1349.8	66.6
6177	6427.2	2287.5	1109.9	4533.4	206.7	1750.1	2034.8	196.4
2701.7	2213.3	2285.6	152.8	1376.3	29.2	578.5	658.9	63.4
6030.4	5555.5	2061.2	355.8	3326.1	48.7	1276.7	1396.1	56.5
9172.1	9835	1986.7	719.3	6767.4	109.4	2766.7	3126.9	84.7
4561	5216.1	2036.6	1697.3	3361.4	448.1	1583.2	1498.3	370.5

7414.3	6873.4	2005	298.8	3850.5	44.9	1692.1	1699	56.4
6337.9	6592.5	2039	543.3	3742.2	64.4	1404.7	1549.3	62.1
18787.2	13177.6	1854.8	1265.4	9510.8	211.5	4227.3	4284.1	316
8328.2	6652.7	2037.5	879.1	4475	171	1823.3	1914.6	163.9
3078.2	3575.9	2102.6	821.8	2202.8	181.1	893	911.8	166.1
4063.2	5234.8	1982.1	1010.6	3594.3	222.1	1496.9	1546.2	178.6
10128.8	7315.3	2111.7	2027.9	5204.8	541.7	2181.1	2355.5	444.7
11653.3	12382.6	2104.6	3659.3	7125.3	755.9	3397.2	2691.6	726.1
13838.5	12336.4	2260	470.2	8916.4	73.7	2894.2	4271.6	86.9
6127.9	6644.9	2144.7	424.7	4235.9	58.1	1439.7	1924.3	78.5
7776.4	7462.8	1989.6	1178.5	4974.7	203	2199.9	2161.2	189.9
5606.3	5609.3	1995.7	1069.3	3784.2	231.4	1463.2	1541.2	211.1
3952.4	5575.8	2069.6	685.5	3876.3	125.4	1411.8	1574.2	111.1
9952.5	9526.3	2072.7	1151.8	6596.4	191.4	2858.3	2991.9	222.5
6280.8	7375.9	2019.9	1082.9	5666.5	210.2	1989	2552.8	184.6
5664.4	6337.9	2127.6	440	3633.1	62.1	1381.9	1624	78.3
6594.2	5600.7	2081	1123.1	4236.9	239.5	1480.3	1690.6	216.2
3417	3678.6	2127.1	1294.5	2519	342.3	912.7	1004.4	233.2
4368.4	4376.5	2076.9	267.7	2945.6	46.5	910.5	1237.5	63.9
4194.5	4858.6	1971.3	855.5	3225.9	203.7	1394.2	1352.4	174.8
5238.4	5517	2151.3	1905.5	3455	441.4	1439.8	1348.9	364.2
3943.1	4269.8	2068.4	1242.4	3109.5	348.5	1200.9	1298.8	250.6
6481.5	6317	2126.7	1761.6	5318.8	498.9	1853.7	2558.8	367.6
6496.4	6131.3	2126.1	1758.8	4775.5	461.3	1950.6	2197.6	361.4
6130.4	6371.3	2163.4	1840.8	4808.5	480.1	2039	2291.2	409
5185	4801.8	2169	1587.5	3425.6	388	1171	1479.9	292.7
7500.7	6998.2	2175.4	1752.4	4544.6	366.3	1818.8	1932	352.1
2701.6	3619.2	2085.6	1046.2	2561.5	260.5	897.2	1039.4	203.7
4833.5	4806.5	2020.1	1627	3558.7	447	1320	1591.6	374.7
4950.5	4212.5	2029.2	1045.2	2763.6	251.5	1134.5	1267.1	225.3
5565.3	6006.8	2100.4	1944.7	3712.5	424.8	1522.9	1362.8	362.4
9830.9	9033.4	2034.7	2296.6	7962	652.4	2982.5	3708.9	530.8
4710	5677.8	2053.2	1179.8	3833.1	271.6	1549.4	1654.6	237.3
6781.7	6860.9	2097.1	1979	4770.7	430.7	1872.2	1945.2	368.4
3873.9	4905.9	2123.5	1764.8	3437.3	432.9	1255.2	1340.6	315.1
2189.8	2325.2	2129.6	907.1	1464	226.9	519.2	607.3	160.7
5011.4	6758.5	2031.9	1748.5	3824.5	364.3	1659.8	1420.5	322.7
4887.1	6337.9	2117	1980.6	4429.4	460.4	1745	1726.3	359.8
4811.6	5019.1	2182.6	1417.2	4042.6	400.3	1303.8	1877.7	309
7254.5	7705.8	2152.7	1824.6	5753.6	470.4	2497	2737.9	406.7
5228.1	5632.1	2111.6	1131.4	3556	231.6	1277.9	1506.3	225
5620.3	6656.1	2167.3	1938.7	4718.4	506	1752.8	1966.1	368
3970.9	4061.7	2204.6	1444	2511.2	360.8	1075.9	1112.9	299.4
3935.6	4141.4	2216.3	1296.3	2973.5	332.9	1144.3	1234.5	297.8
8292.4	8193.3	2174.2	2268.3	6524.3	630	2416.2	2855.4	455.2
6741.1	5854.6	2168.2	1281.3	3584.7	272.7	1544.6	1532.6	246.7
5913.6	3723.6	2305.8	1475.6	2428.5	358.4	957	1058.4	296.3
2848.5	2836.2	2073.2	1052.7	1981.4	272.4	712.2	803	225.7
4018.6	4372	2099.6	1253.5	3534.3	318.1	1394.7	1553.8	295
7034.2	6672	2096.2	1259.5	4783.2	270.2	1812.1	1963.9	248
4539	4385.2	2075.3	1406.4	3389.4	399.3	1284.6	1482.1	325.9
17164	7108.1	2059.9	2344.6	5254.1	582.6	2101.7	2372.9	516.8
3111.3	3748.5	2166.2	1309.1	2549.8	339.2	913.1	983.5	244.6
4047.7	4423.7	2177.5	1565.9	3326.6	386.8	1171	1306.5	327.2
5523.4	5076.7	2125.9	1008	3748.4	216.9	1336.4	1615.5	179.7
7029.1	5933.2	2028.5	1699.8	3322.7	331.6	1521.6	1352.4	314.4

3407.1	3942.6	2150.6	1341.7	2550.2	319.3	996.2	978.6	256.3
3688.2	3261.6	2101.7	765.3	2005.7	155.5	753.1	818.9	148.5
3436.8	3853.3	2234.6	1270.8	2740.6	311.8	994.1	1244.1	250.6
7384.5	6044.9	1984.5	377.8	3869.7	57.3	1353.9	1907.5	70.3
3252.3	3273.8	2014.9	995.7	2171.6	258.3	697.5	832.3	187.4
7227.7	7577.2	2233.4	1985.6	5904.2	511.4	2522.1	2942.7	481.4
7379.6	6899	2018.2	1130.9	5118	260.9	2101	2385.6	262.4
4358.1	4437.5	2193.5	1601	3151.1	403.1	1182.2	1370.6	324.1
6020.2	5665.4	2134.9	869.8	4266.9	182.4	1346.1	1968.9	166.8
5862.2	5034.1	2152.8	1777.7	3774.9	482.2	1554.6	1693.4	395.2
9423.5	8673.1	2017.1	2257.1	7307.8	598	2696.6	3325.5	463.5
3740.9	4008.5	2167.2	1192.3	2773.1	304.7	1079.7	1208.5	246.3
1790.4	1558.1	1374.7	594.6	796.5	112.3	357.5	330.7	123.6
2239.2	2152.3	2060.1	801.8	1252.1	181.7	505.6	516.2	152.1
7847.1	6157.9	2108.3	1880.5	4218	497.3	1927.9	2061.3	445.6
8289.1	2456.2	2091.4	887.2	1464.7	193.4	658.9	656.7	194.5
1206.133	1101.200	503.933	294.800	689.900	86.400	336.967	306.000	73.333
584.000	606.600	277.200	156.300	388.000	43.750	165.850	171.150	33.350
519.200	547.450	239.000	149.800	340.500	40.650	145.100	156.250	27.750
589.150	591.300	238.200	186.600	372.350	55.250	167.600	152.900	36.400
470.550	495.250	238.600	120.950	297.750	35.700	138.150	132.450	27.300
995.733	980.733	510.867	262.367	638.933	79.733	277.867	295.200	61.867
1004.900	1040.750	245.800	216.300	797.600	74.600	340.950	376.650	51.700
3236.600	2872.150	642.050	561.700	2190.950	166.200	971.000	1043.700	147.650
806.500	799.300	232.700	156.550	503.400	42.300	241.600	233.550	34.000
777.850	802.000	238.700	197.550	539.450	60.200	226.600	237.950	43.200

L Leu	D alle	D Leu	**Valve**
228.5	23.6	46.3	right
121.5	12.9	28.5	right
257.9	20.1	41.7	right
297.6	35.6	54.1	right
298.6	25	51.2	right
250.6	21.1	44.6	right
187.7	24.9	47.2	right
258.2	26.5	55.1	right
364.5	30.2	47.9	right
242.3	38.7	45.1	right
414	29.7	64.6	right
168.5	14.3	27.3	right
320.6	31.4	67.2	right
220	13.4	31.5	right
409.1	36.1	85.2	right
295.6	28.1	53.7	right
509.7	45.5	84.4	right
466.9	37.9	63.5	right
145.7	21.5	63.8	right
302.5	30.7	72.8	right
153.8	19.8	38.7	right
414	31.9	68.7	right
280.2	24.4	51.8	right
215.8	25.1	49.5	right
259.6	28.7	62.7	right
283.5	27.5	45.8	right
289.4	26.5	52.2	right
234.6	25.7	47.9	right
223.5	19	39.1	right
311.3	38.8	73.9	right
284.7	28.3	48.9	right
184.2	22.4	37.7	right
496.8	34	85.2	right
349.5	28	57.5	right
608.7	55.8	90.9	right
309.1	28.2	59.3	right
300.9	28.6	55.9	right
339.7	33.9	62	right
398.5	26.1	61.3	right
184.2	20.3	39.4	right
222.4	24.4	52.5	right
262.6	28.7	46.5	right
256.7	18.5	38.7	right
142	17	34	right
318.5	36.6	67.4	right
214.2	18.2	40.1	right
225.4	24.7	48.8	right
393.4	31.5	70.3	right
227	22.4	36	right
801.6	47.9	98.7	right
346.6	23.7	57.3	right
213.5	20.7	36.6	right
358.1	29.2	56.1	right

191.3	20	39.7	right
196.8	22.2	40.1	right
304.4	32.3	63.2	right
377.1	27.6	61.2	right
345.4	20.9	45.3	right
125	13	35.6	right
516.3	34.4	71.2	right
314.7	33.4	60.5	right
626.5	47.3	95.3	right
483.2	30.8	74.8	right
157.6	18.3	37.4	right
227.9	18.8	46.4	right
132	17.5	34.5	right
322.6	17.4	40.4	right
211.3	27.8	52	right
221.5	23.9	42.5	right
379	38.3	76.9	right
279.6	28.4	49.9	right
249.3	23.4	52.4	right
166.1	17.6	32.4	right
473.4	39.3	77.5	right
289.8	24.2	50.9	right
119.9	16.3	32.2	right
201.7	25.8	43.9	right
191	20.7	40.5	right
298.6	24.4	51	right
183.2	16.8	38.7	right
154.9	17.7	33.4	right
476	60.3	114.3	right
199.1	17.3	39.2	right
251.5	24.8	38.2	right
74.9	17.4	34.8	right
562.7	40.6	79.5	double
355	25.8	54.6	right
213.8	23.9	38.5	right
284.1	24.2	45.9	right
291.7	27.9	62	right
548	43.6	80.1	right
274.7	26.7	55.1	right
215.8	24.4	41.4	right
261.8	24.3	48.7	right
575.1	36.8	75.6	right
204.5	20.6	38.7	right
119	14.1	32.5	right
165.8	18.3	42.2	right
233.2	24.7	45.5	right
202.2	16.7	36.4	right
585.7	41.3	85.7	right
186.6	17.6	37.5	right
277	26.4	49.6	right
602	35.5	78.3	right
295.3	26.4	42.6	right
306.5	25.3	52.4	right
257.7	17.8	33.2	right
234	22.5	46.6	right
309.6	27.9	48.9	right

152.2	17.5	39.9	right
213	21.6	38.1	right
248.4	23.4	47.6	right
264.2	27.3	48.2	right
1023.1	19	36.4	left
207.8	20.9	38	right
284.2	24.2	53	right
559.3	35.6	71	right
248.5	26	51.9	right
354	19.5	39	right
197.5	23.1	47.3	right
373.2	28.6	58.3	right
142.1	12.1	23.5	right
378	42.3	81.6	right
100.8	12.4	26.4	right
332.2	27.6	53.8	right
298.5	24.7	56.7	right
173.4	17.2	33.2	right
255.3	27	61.9	right
276.2	30.2	58.5	right
314.4	27.1	65.1	right
169	20.2	35.1	right
141.2	19.4	34.3	right
167.8	16	31	right
764.6	68.6	154	right
255.4	27.4	100.3	right
187.3	16.8	38.2	right
544.5	44.5	75.9	right
8804.7	30.6	135.1	right
7000.7	227.5	531.4	right
11321.8	94.3	341.4	right
1895.8	84.5	271.5	right
3835.9	157.6	359	right
7461.1	38.4	241.8	right
7473.7	85.8	241.7	right
3519.4	120.1	331.8	right
3788.3	30.5	160.9	right
1914.3	158.7	429.3	right
1302.6	121.6	371.3	right
1229	69.5	197.2	right
10231	99.9	357.9	right
2457.6	100.2	310.3	right
2418.3	84	290.2	right
2761.3	77.8	277.1	right
3929.9	39.9	217.8	right
991.7	105.1	271	right
717.9	88.5	219.1	right
2036.6	90.4	281.3	right
1783.4	95.1	272.9	right
3241.2	152.9	339.3	right
2037.1	25.1	127.3	right
3024.1	102.8	343.7	right
861.8	13.1	63.6	right
2147.9	12.3	98.2	right
4687	40	205.7	right
2489.9	246.6	645.2	right

2696.8	18.1	77.8	right
2512.1	37.6	173.3	right
5754.7	67	338.4	right
2989.4	88.9	288.5	right
1384.9	68.9	201.7	right
2098	92.5	308	right
3206.9	249.3	668.8	right
4246.5	346.7	807.2	right
5890	39	150.2	right
2616.7	29.8	122.6	right
3029.2	100.9	322.7	right
2596.7	110.9	409.8	right
2362.4	56.5	252.1	right
4545.2	95.2	351.2	right
3733.2	115	357.5	right
2261.4	20.1	89.2	right
2519.4	136.7	321.9	right
1593.8	165.4	431.8	right
1776.1	33.8	83.3	right
2047	85.8	301.6	right
2054.3	197.2	570.8	right
2013.3	194.8	498.9	right
3706.1	273.2	676.7	right
3413.5	240.1	666.8	right
3338.1	270.2	687.2	right
2192.9	185.9	505.3	right
2963.8	194.4	521	right
1558.1	131.1	356.2	right
2195.1	236	560.3	right
1839.5	143.8	379.2	right
2168.7	177.2	519.5	right
5625.6	362	951.8	right
2534.2	145.9	416	right
3051.9	207.3	550.9	right
2120.2	200.3	554.2	right
894.1	117.3	280.8	right
2225.1	153	404.4	right
2893.6	220.8	631.8	right
2723.1	209.7	534.1	right
4258.6	268.1	762.3	right
2210.6	98.4	407.7	right
2977.2	250	671.4	right
1656.4	170.3	449.9	right
1890.5	151	471.9	right
4481.3	273.6	867.2	right
2448.8	139.1	426.6	right
1590.1	192.5	514.4	right
1206.9	138.5	361.1	right
2253.9	158.3	472.7	right
3160.2	132.5	393.7	right
2307.3	217.4	610.5	right
3485.6	332.4	813.1	right
1508.3	161.9	436.4	right
1987.9	195.6	552.7	right
2616.6	99.3	346.9	right
2188.2	171.7	416.2	right

1543	153.7	408.8	right
1275.4	73.3	248.3	right
1712.2	165.8	426.1	right
2642	32.4	110.1	right
1276.7	120.6	311.6	right
4372.1	301.8	800.3	right
3517.1	138.1	439.8	right
2193.4	221	640.6	right
2704.5	98.7	279.5	right
2850.1	268.9	771.6	right
5005.9	321.1	827.2	right
1873.9	152.8	415.4	right
513.3	64.9	184.6	right
782.6	96.5	268	right
3080.9	315.9	700.3	right
1004.4	113	295.7	right
460.833	52.567	99.600	right
252.150	24.300	44.050	right
222.600	21.400	40.650	right
243.100	29.100	51.750	right
200.100	18.250	33.400	right
400.333	46.000	83.100	right
559.550	39.450	64.400	right
1784.300	101.450	214.550	right
383.250	25.300	47.700	right
377.600	31.150	59.650	right
