# Supplementary material for: A decline in molluscan carbonate production driven by the loss of vegetated habitats encoded in the Holocene sedimentary record of the Gulf of Trieste
Source: Sedimentology. 2018 Aug 25;66(3):781–807. doi: 10.1111/sed.12516 (PMC6446828; doi:10.1111/sed.12516)
Supplement: Supplementary file 4 — Table S4. Absolute abundances of molluscs in living assemblages collected by Van Veen grabs at 11 stations in 2011. [file SED-66-781-s004.pdf]

| **Species** | MSN01 | MSN02 | MSN03 | MSN04 | MSN05 | MSN06 | MSN07 | MSN08 |
|---|---|---|---|---|---|---|---|---|
| Abra alba | 1 | 2 | 0 | 1 | 2 | 0 | 2 | 0 |
| Abra prismatica | 1 | 0 | 0 | 1 | 1 | 1 | 1 | 4 |
| Abra sp. | 0 | 0 | 0 | 0 | 0 | 0 | 0 | 0 |
| Acanthocardia paucicostata | 0 | 0 | 0 | 0 | 0 | 0 | 0 | 0 |
| Acanthocardia tuberculata | 0 | 0 | 0 | 0 | 0 | 0 | 0 | 0 |
| Akera bullata | 0 | 0 | 0 | 1 | 0 | 0 | 0 | 0 |
| Anomia ephippium | 0 | 0 | 0 | 0 | 1 | 0 | 0 | 0 |
| Antalis inaequicosta | 5 | 0 | 0 | 4 | 6 | 1 | 9 | 20 |
| Aporrhais pespelecani | 0 | 1 | 0 | 0 | 0 | 1 | 2 | 0 |
| Atrina pectinata | 0 | 0 | 0 | 0 | 0 | 1 | 0 | 0 |
| Azorinus chamasolen | 1 | 0 | 1 | 0 | 0 | 0 | 0 | 0 |
| Bela nebula | 0 | 1 | 0 | 0 | 0 | 0 | 0 | 0 |
| Calyptraea chinensis | 1 | 1 | 2 | 0 | 2 | 0 | 1 | 1 |
| Corbula gibba | 1 | 0 | 0 | 0 | 19 | 5 | 7 | 60 |
| Cylichna cylindracea | 0 | 0 | 0 | 0 | 1 | 0 | 0 | 0 |
| Epitonium pulchellum | 0 | 0 | 0 | 0 | 0 | 1 | 0 | 0 |
| Eulima glabra | 2 | 0 | 0 | 0 | 1 | 1 | 6 | 3 |
| Eulimella scillae | 0 | 0 | 0 | 0 | 1 | 0 | 0 | 0 |
| Euspira guilleminii | 0 | 1 | 0 | 1 | 3 | 0 | 0 | 0 |
| Euspira pulchella | 1 | 0 | 0 | 1 | 0 | 0 | 0 | 0 |
| Fusinus rudis | 0 | 0 | 0 | 0 | 0 | 0 | 0 | 0 |
| Gari fervensis | 0 | 1 | 0 | 0 | 0 | 0 | 0 | 2 |
| Gastrochaena dubia | 0 | 0 | 0 | 0 | 0 | 0 | 0 | 0 |
| Hexaplex trunculus | 0 | 0 | 1 | 0 | 0 | 0 | 0 | 0 |
| Hiatella arctica | 0 | 0 | 2 | 1 | 1 | 0 | 0 | 2 |
| Ischnochiton rissoi | 0 | 0 | 0 | 0 | 0 | 0 | 0 | 0 |
| Kellia suborbicularis | 0 | 0 | 0 | 0 | 0 | 0 | 0 | 0 |
| Kurtiella bidentata | 1 | 1 | 1 | 3 | 0 | 50 | 0 | 2 |
| Laevicardium crassum | 0 | 0 | 1 | 0 | 0 | 0 | 0 | 0 |
| Laevicardium oblongum | 0 | 0 | 1 | 0 | 0 | 0 | 0 | 0 |
| Loripes lacteus | 1 | 0 | 1 | 0 | 0 | 0 | 0 | 0 |
| Lucinella divaricata | 0 | 0 | 1 | 0 | 0 | 0 | 0 | 0 |
| Melanella polita | 0 | 1 | 0 | 0 | 0 | 0 | 0 | 0 |
| Mimachlamys varia | 0 | 0 | 1 | 0 | 0 | 0 | 0 | 0 |
| Modiolarca subpicta | 0 | 0 | 1 | 0 | 0 | 0 | 1 | 0 |
| Myrtea spinifera | 0 | 0 | 0 | 1 | 1 | 1 | 0 | 1 |
| Nassarius incrassatus | 0 | 0 | 0 | 0 | 0 | 0 | 0 | 0 |
| Nassarius pygmaeus | 0 | 0 | 0 | 0 | 0 | 0 | 0 | 0 |
| Nassarius reticulatus | 1 | 0 | 1 | 0 | 0 | 1 | 1 | 1 |
| Nucula nucleus | 2 | 0 | 5 | 0 | 0 | 0 | 1 | 4 |
| Nuculana commutata | 0 | 0 | 0 | 0 | 0 | 0 | 0 | 0 |
| Nuculana pella | 0 | 1 | 0 | 0 | 0 | 0 | 0 | 0 |
| Parvicardium minimum | 0 | 0 | 0 | 0 | 0 | 0 | 0 | 0 |
| Parvicardium scabrum | 0 | 0 | 20 | 0 | 0 | 0 | 0 | 0 |
| Phaxas adriaticus | 2 | 4 | 7 | 3 | 2 | 0 | 0 | 0 |
| Philine aperta | 3 | 0 | 0 | 0 | 0 | 0 | 2 | 0 |
| Pitar rudis | 0 | 0 | 0 | 0 | 0 | 0 | 1 | 1 |
| Plagiocardium papillosum | 0 | 0 | 0 | 0 | 0 | 0 | 0 | 1 |
| Saxicavella jeffreysi | 0 | 0 | 0 | 0 | 0 | 1 | 5 | 0 |
| Scapharca inaequivalvis | 0 | 0 | 0 | 0 | 0 | 0 | 0 | 1 |
| Smithiella costulata | 0 | 1 | 0 | 0 | 0 | 1 | 0 | 2 |
| Solecurtus scopula | 1 | 0 | 0 | 0 | 1 | 0 | 0 | 0 |

| | | | | | | | | |
|---|---|---|---|---|---|---|---|---|
| Striarca lactea | 0 | 0 | 0 | 0 | 0 | 0 | 0 | 0 |
| Tellina distorta | 3 | 3 | 2 | 0 | 1 | 1 | 0 | 1 |
| Tellina serrata | 0 | 1 | 0 | 0 | 0 | 0 | 0 | 1 |
| Timoclea ovata | 0 | 0 | 0 | 0 | 0 | 0 | 0 | 0 |
| Thyasira biplicata | 0 | 0 | 0 | 0 | 0 | 0 | 0 | 0 |
| Turritella communis | 2 | 2 | 0 | 1 | 16 | 55 | 9 | 32 |
| Venerupis aurea | 0 | 0 | 0 | 0 | 0 | 0 | 0 | 0 |

| MSN09 | MSN10 | MSN11 | MSN12 |
|---|---|---|---|
| 0 | 9 | 0 | 1 |
| 0 | 0 | 0 | 0 |
| 1 | 0 | 0 | 0 |
| 1 | 0 | 0 | 0 |
| 2 | 0 | 0 | 0 |
| 0 | 0 | 0 | 0 |
| 0 | 1 | 0 | 0 |
| 1 | 2 | 0 | 0 |
| 3 | 0 | 0 | 0 |
| 0 | 0 | 0 | 0 |
| 0 | 0 | 0 | 0 |
| 0 | 2 | 0 | 1 |
| 1 | 0 | 0 | 0 |
| 1 | 2 | 0 | 0 |
| 0 | 1 | 2 | 1 |
| 0 | 0 | 0 | 0 |
| 0 | 0 | 0 | 0 |
| 0 | 2 | 0 | 0 |
| 0 | 0 | 0 | 0 |
| 1 | 0 | 0 | 0 |
| 0 | 0 | 1 | 0 |
| 0 | 0 | 0 | 1 |
| 0 | 0 | 0 | 5 |
| 0 | 0 | 0 | 0 |
| 0 | 0 | 0 | 0 |
| 0 | 0 | 1 | 0 |
| 0 | 2 | 0 | 0 |
| 0 | 0 | 0 | 0 |
| 1 | 1 | 1 | 0 |
| 0 | 0 | 0 | 0 |
| 1 | 1 | 1 | 2 |
| 0 | 0 | 0 | 0 |
| 0 | 0 | 0 | 0 |
| 0 | 0 | 0 | 0 |
| 0 | 0 | 0 | 0 |
| 1 | 0 | 0 | 0 |
| 0 | 0 | 2 | 0 |
| 1 | 0 | 0 | 0 |
| 0 | 1 | 0 | 0 |
| 1 | 4 | 0 | 2 |
| 0 | 1 | 0 | 0 |
| 2 | 1 | 0 | 0 |
| 0 | 0 | 0 | 2 |
| 1 | 1 | 2 | 0 |
| 6 | 10 | 1 | 9 |
| 0 | 0 | 0 | 0 |
| 0 | 2 | 0 | 1 |
| 1 | 1 | 1 | 0 |
| 0 | 0 | 0 | 0 |
| 0 | 0 | 0 | 0 |
| 0 | 1 | 0 | 0 |
| 0 | 0 | 0 | 0 |

| | | | |
|---|---|---|---|
| 1 | 0 | 0 | 0 |
| 1 | 11 | 0 | 7 |
| 3 | 2 | 0 | 0 |
| 0 | 0 | 1 | 0 |
| 0 | 1 | 0 | 0 |
| 3 | 4 | 0 | 0 |
| 0 | 1 | 0 | 0 |
